# Supplementary material for: Population genomics uncovers loci for trait improvement in the indigenous African cereal tef (Eragrostis tef)
Source: Commun Biol. 2025 May 26;8:807. doi: 10.1038/s42003-025-08206-5 (PMC12106829; doi:10.1038/s42003-025-08206-5)
Supplement: Supplementary file 2 — Supplementary Information [file 42003_2025_8206_MOESM2_ESM.pdf]

Supplementary Information

Population genomics uncovers loci for trait improvement in the indigenous African cereal tef (*Eragrostis tef*).

Maximillian R. W. Jones[†], Worku Kebede[†], Abel Teshome[†], Aiswarya Girija, Adanech Teshome, Dejene Girma, James K. M. Brown, Jesus Quiroz-Chavez, Chris Jones, Brande B. H. Wulff, Kebebew Assefa, Zerihun Tadele, Luis A. J. Mur, Solomon Chanyalew[*], Cristobal Uauy[*], Oluwaseyi Shorinola[*]

Supplementary Note 1: Additional Information on genotyping of the core collection with a minimal SNP set.
We called the minimal SNPs on a VCF file derived from pooled, subsampled FASTQ files. We therefore checked that the individual constituents of the pools didn't disagree with the "consensus" calls for the 28 selected SNPs.

There were eight cases where the VCF file for the 220 individual accessions differed from the pool calls for the 28 SNPs. In six of these cases, one member of a group was called heterozygous where the group itself was called homozygous. However, in all cases these heterozygous calls were based on a total read depth < 10 and/or received a GQ score < 15. This implies that the calls were not very high confidence - indeed, the empirical filters derived for the 150 accession and 220 accession VCFs would have converted such calls to missing data.

Additionally, in each of these cases, the heterozygous calls for the group members trended towards the genotype assigned to their respective group. For example, DZ-01-348 received a heterozygous call for SNP 17, but the reads supporting this call were actually 1 read for the reference allele and 8 for the alternate allele. Given that the group that DZ-01-348 is part of (DZ-01-136 group) was called homozygous for the alternate allele, this suggests that a higher read depth might have led to the correct call for DZ-01-348 for this SNP.

Lastly, there were two cases where different group members had different types of homozygous calls for a SNP. However, these groups have missing data for those particular SNPs, so do not use them for discrimination from other accession groups and singlets. These discrepancies therefore do not change which of the 150 accession groups and singlets the group members would be assigned to.

For the new DZ-01 group, DZ-01-383 is heterozygous for SNP 13. In the DZ-01-136 group, DZ-01-348 is heterozygous for SNP 1 and SNP 17. In the DZ-01-647 group, DZ-01-647 is heterozygous for SNP 7. In the DZ-01-1556_1 group, DZ-01-1556_2 is heterozygous for SNP 6. Also, SNP 6 has member accessions homozygous for both alleles, however, the DZ-01-1556_1 group has missing data here. In DZ-01-667 group, DZ-01-667 is heterozygous for SNP 7. In the DZ-01-1100 group, SNP 11 has member accessions homozygous for both alleles, however, the DZ-01-1100 group has missing data here.

Supplementary Figures

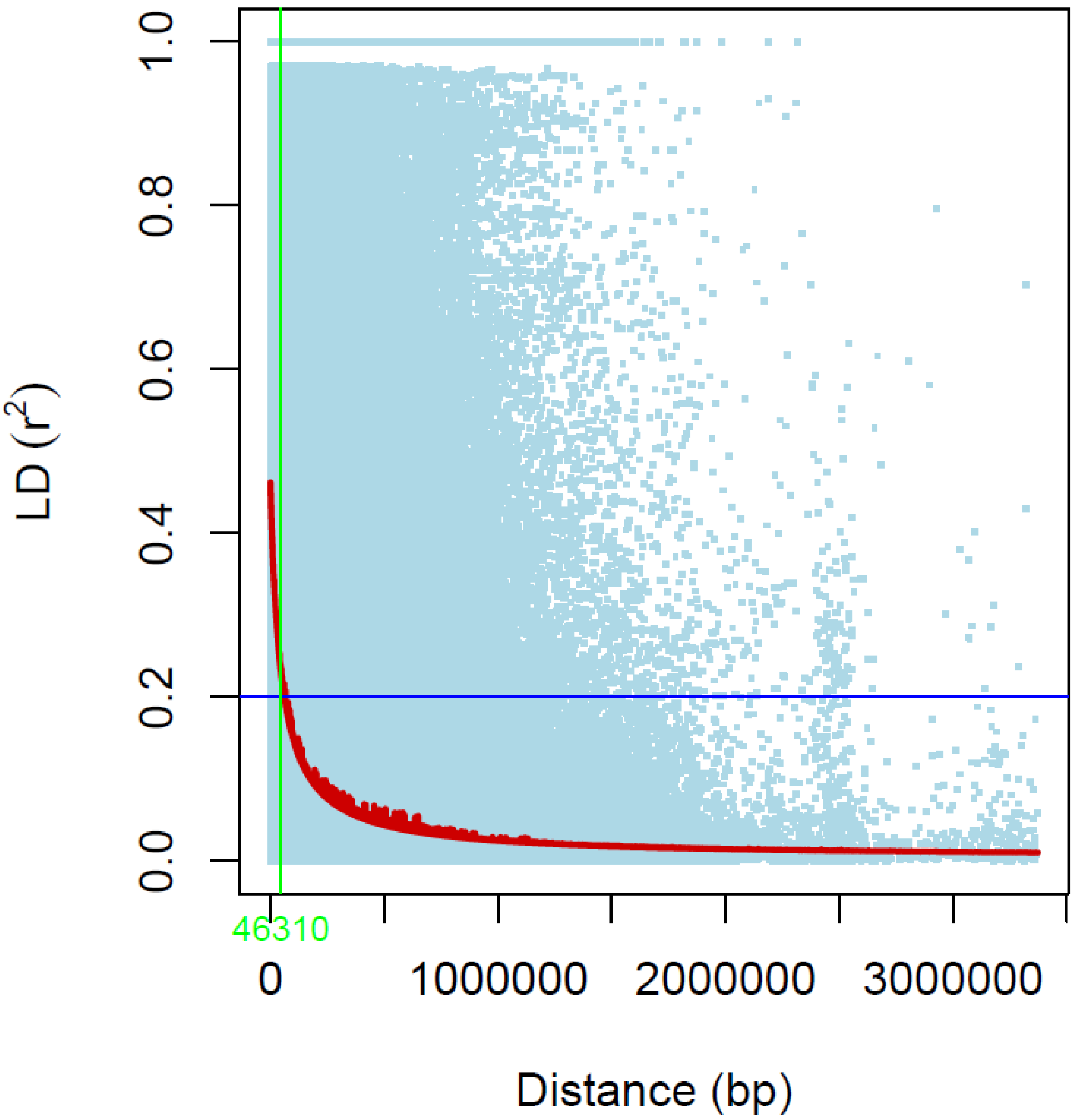

Supplementary Figure 1: A genome-wide Linkage disequilibrium (LD; r^2) decay plots. the average genome-wide LD-decay against the genomic distance. The x-axis represents the physical distance between pairs of SNPs in base pairs (bp). The y-axis represents the LD between SNPs, computed as r^2.

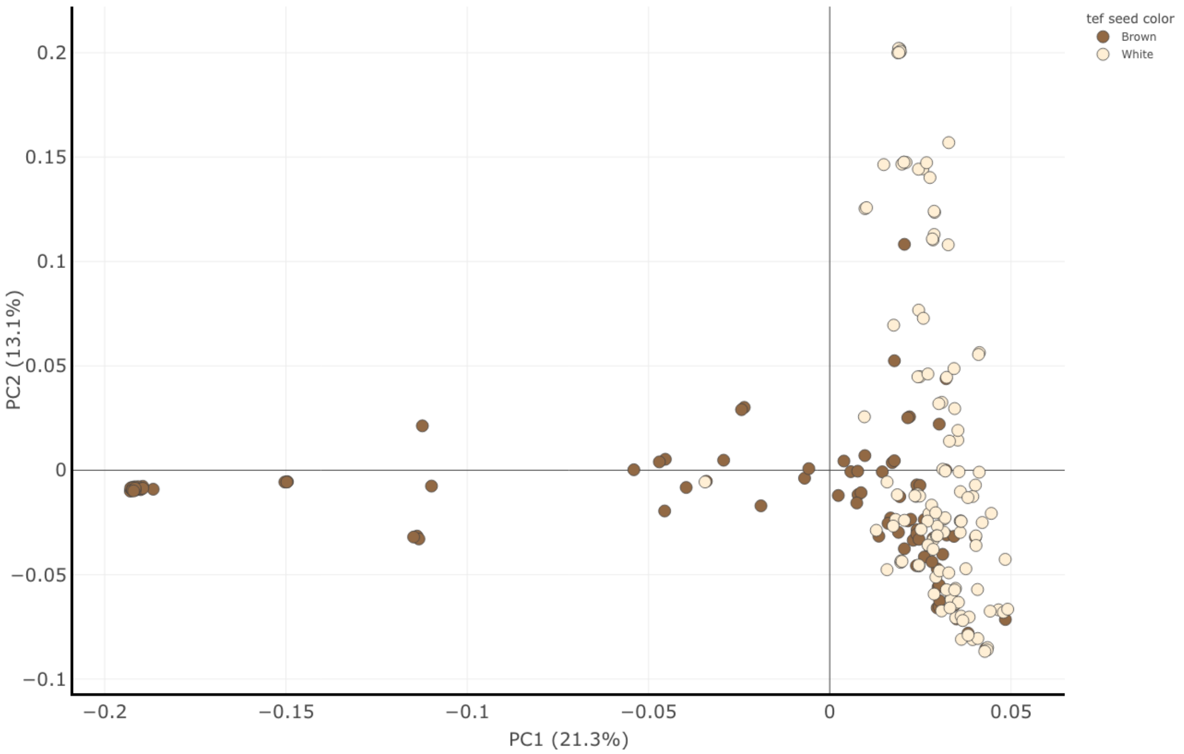

Supplementary Figure 2 – Principal component analysis (PCA) based on ca. 40K SNPs for 230 tef genotypes. A scatter plot of PC1 (explaining 21.3% of the variance) versus PC2 (explaining 13.1% of the variance). Binary colour codes represent seed coat colour of the individual genotypes

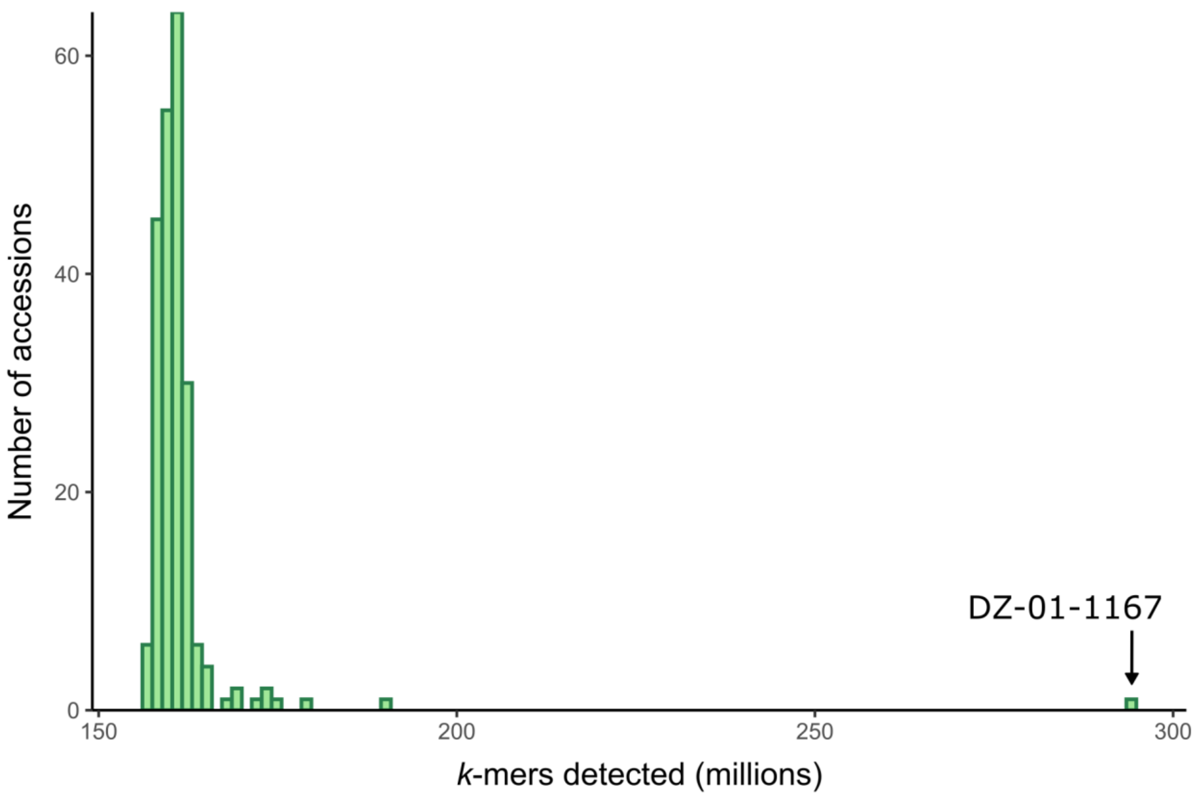

Supplementary Figure 3 - Accession 'DZ-01-1167' contained a uniquely high number of distinct k-mers. Histogram showing number of distinct k-mers per sequenced tef accession (before collapsing redundancy groups). Accession DZ-01-1167 displayed a significantly higher number of distinct k-mers than any other accession (84% higher than the mean of other accessions).

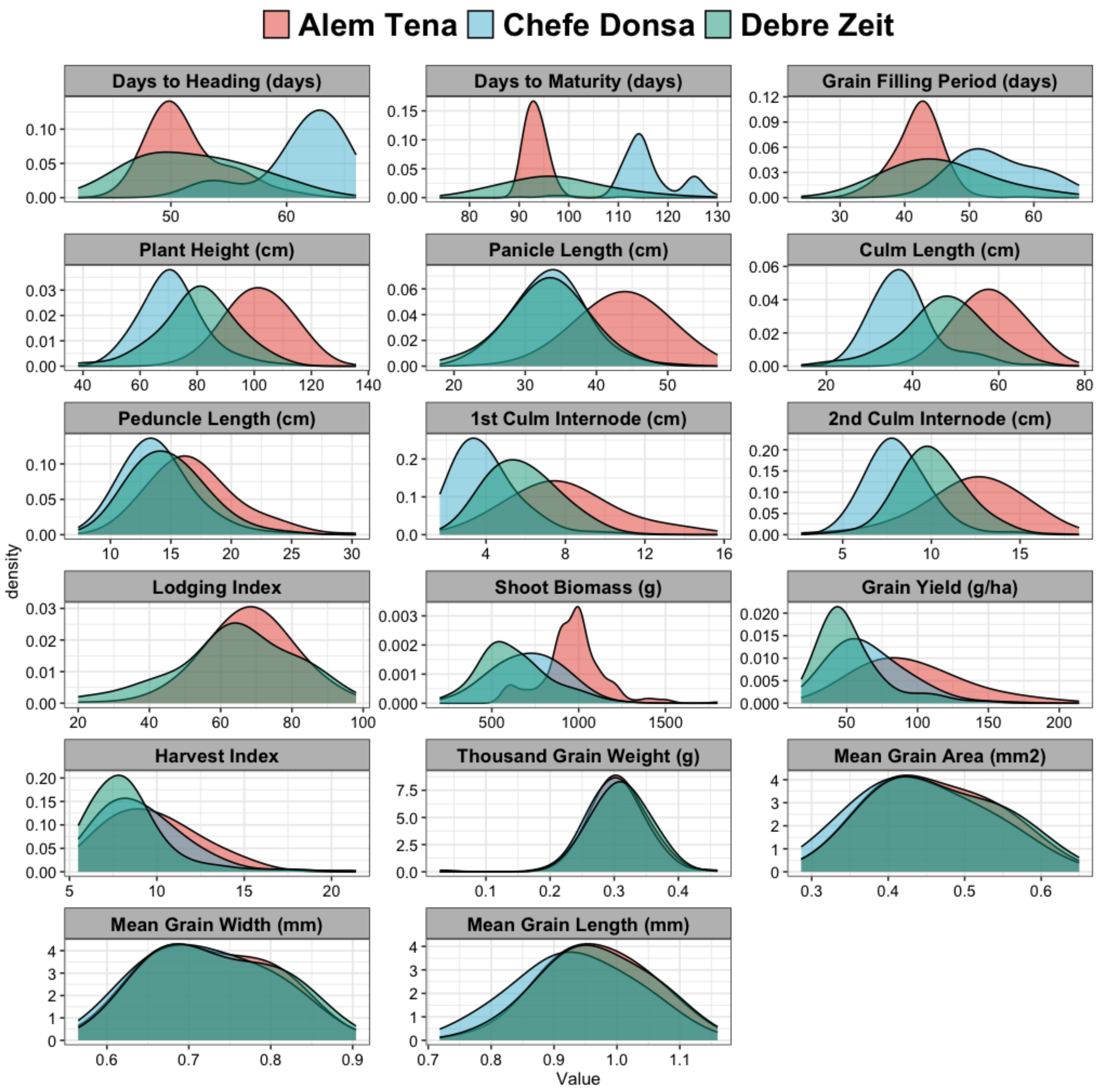

Supplementary Figure 4 - Distribution of quantitative agronomic traits. Density plots showing the raw distributions of quantitative agronomic traits for each location.

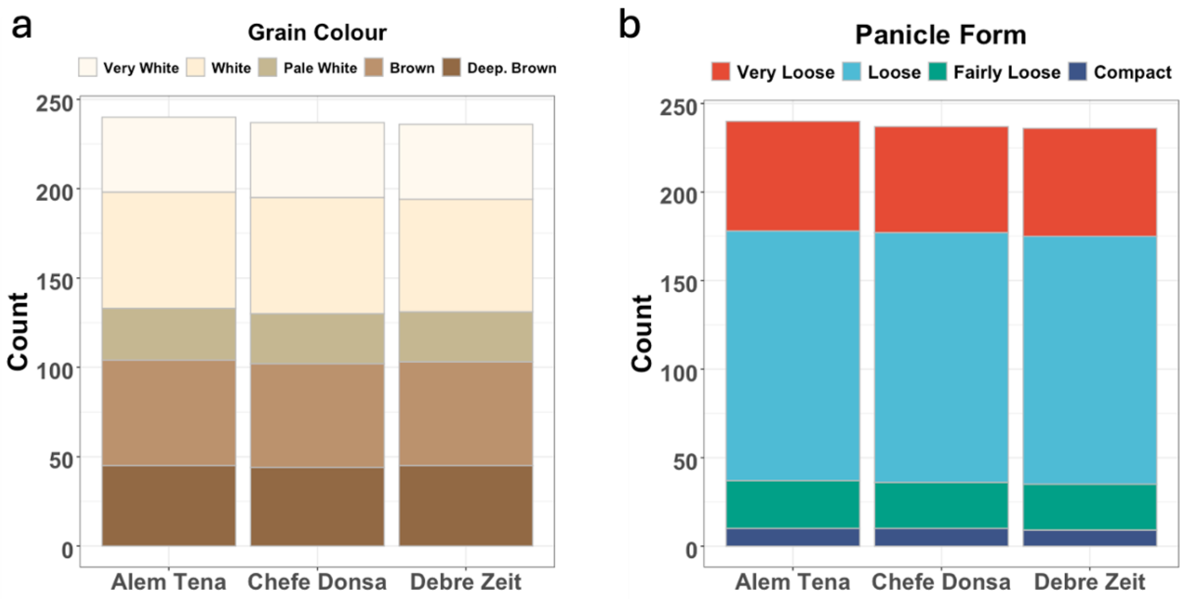

Supplementary Figure 5 - Distribution of qualitative traits. Stacked bar plots showing the distribution of grain colour (a) and panicle form (b) at each location.

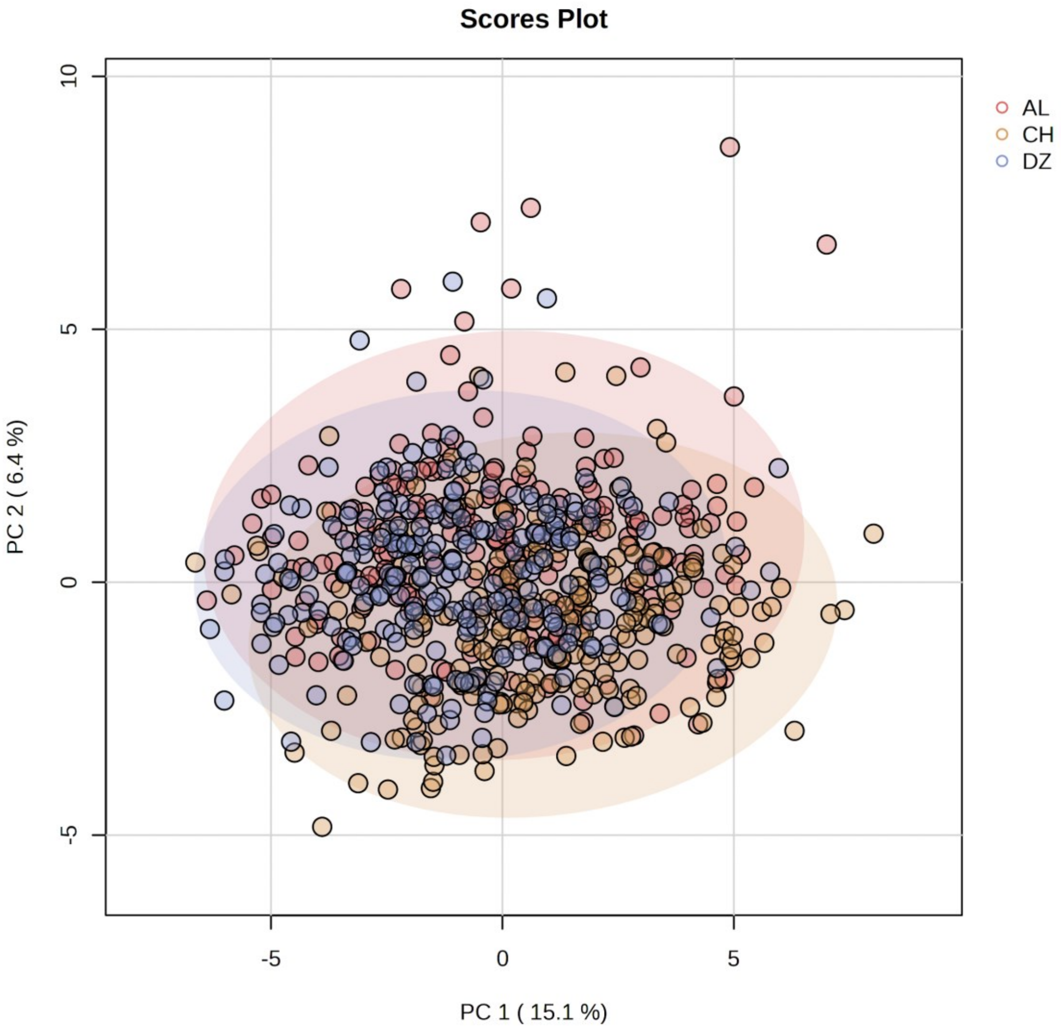

Supplementary Figure 6 – Differentially accumulated metabolites between brown and white-grained accessions did not differ between locations. Principal component analysis of trial plots from all three locations based on the 183 annotated metabolites that were differentially accumulated between brown and white-grained accessions. AL, CH and DZ represent Alem Tena, Chefe Donsa and Debre Zeit, respectively. Points from the three locations were not well-separated, indicating little effect of location on the grain accumulation of these metabolites. Ellipses represent 95% confidence intervals around each group.

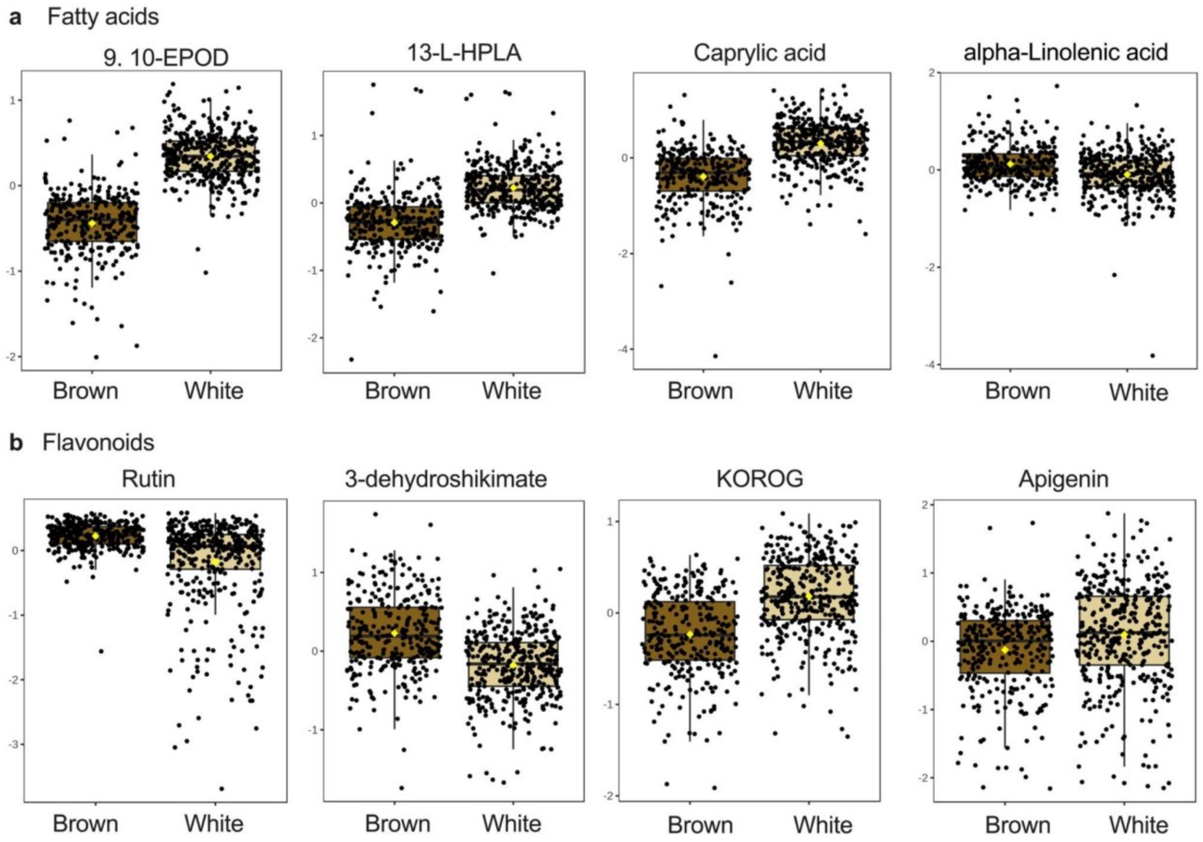

Supplementary Figure 7 – Grain samples from brown and white-grained accessions differed in their accumulation of several fatty acids and flavonoids. Box plots for each metabolite were generated using Metaboanalyst and the plots show the accumulation of selected metabolites in brown and white-grained tef accessions. The metabolites in row **a** are fatty acids while those in row **b** are flavonoids. Relative metabolite concentrations are shown as $\log_2$ transformed values and each dot represents biologically independent samples (brown n=303 and white n=389). The box ranges from 25% and the 75% percentiles; the 5% and 95% percentiles are indicated as error bars. Medians are indicated by horizontal lines within each box.

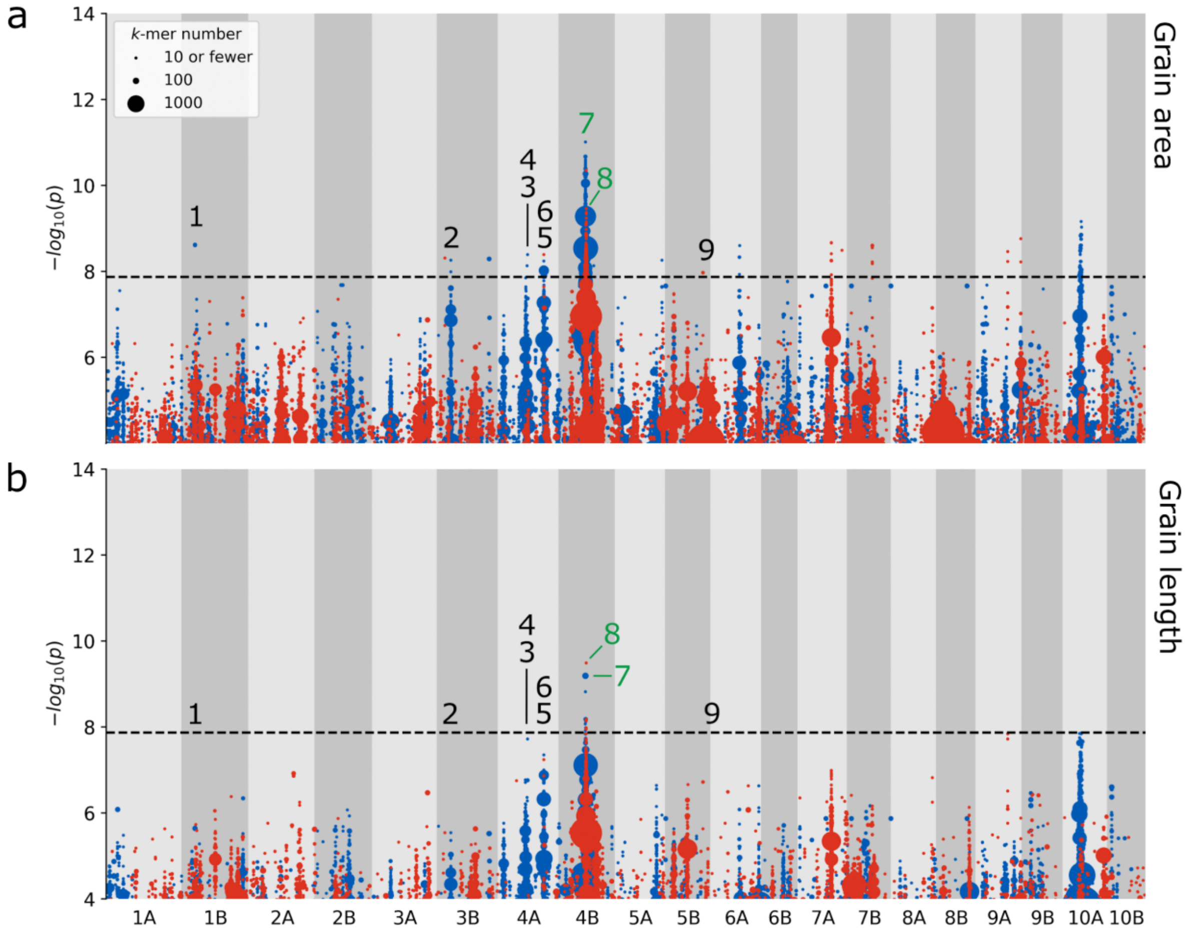

Supplementary Figure 8 - k-mer-based GWAS identifies marker-trait associations for grain area, but not grain length. Plots of *k*-mers associated with **a**, grain area and **b**, grain length. *k*-mers are grouped according to their association level and genomic coordinates (10 kb bins) and coloured according to the direction of association; red for association with lower trait values, and blue for association with higher trait values. Point size is proportional to the number of *k*-mers rounded upwards to the nearest 10. The nine highlighted regions from Figure 7 are labelled with black and green numbers, denoting whether the region is significant or not-significant for the plotted trait, respectively.

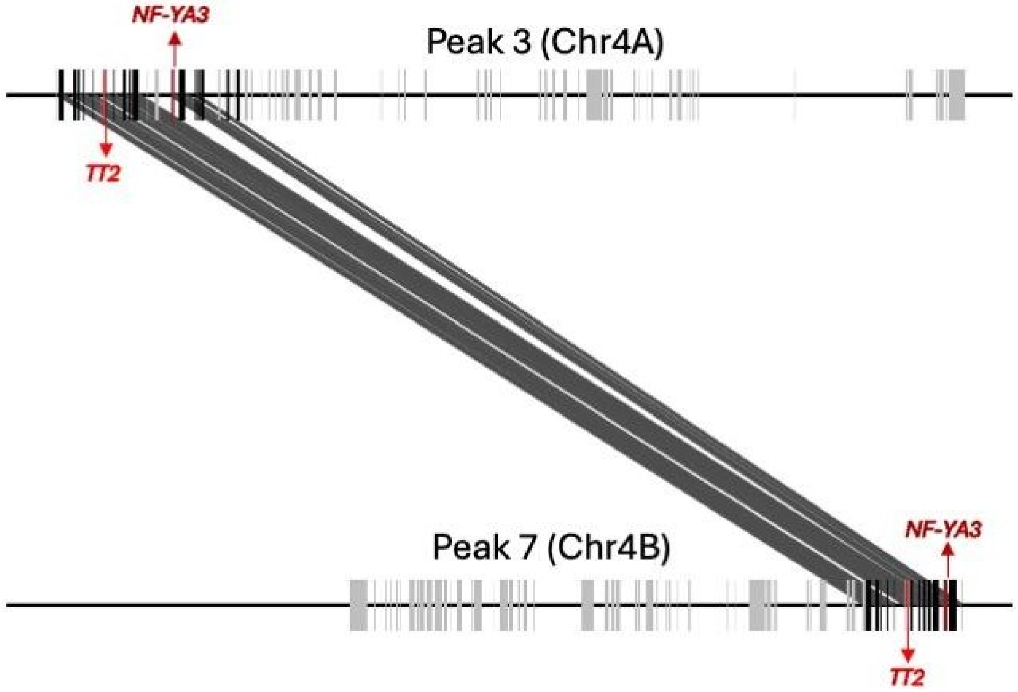

Supplementary Figure 9 - Homoeology in associated regions for grain size, colour and metabolite on Chr4A and Chr4B. Homoeologous relationship between genes in peak 3 and peak 7. The vertical bar represents individual genes, and the diagonal lines connect genes with homoeologous relationships. The candidate genes in the interval are shown in red.

Supplementary Table 1: List of redundancy groups and their constituent accessions

Group name	Mixed colour	Accessions
**DZ-01-New**	No	DZ-01-New, DZ-01-D9, DZ-01-86, DZ-01-2138, DZ-01-383, DZ-01-1481, DZ-01-1593A, DZ-01-385, DZ-01-424, DZ-01-478, DZ-01-1681A, DZ-01-1726, DZ-01-1741, DZ-01-581, DZ-01-634B, DZ-01-1994B, DZ-01-951, Keyagacham-T-6, Shewagemira-T-140
**DZ-01-14**	No	DZ-01-14, DZ-01-104
**DZ-01-36**	No	DZ-01-36, DZ-01-58, DZ-01-71B
**DZ-01-91**	Yes	DZ-01-91, DZ-01-101
**DZ-01-102**	No	DZ-01-102, DZ-01-179, DZ-01-295, DZ-01-886
**DZ-01-1108**	No	DZ-01-1108, DZ-01-1020
**DZ-01-1225**	No	DZ-01-1225, DZ-01-1728
**DZ-01-1233**	No	DZ-01-1233, DZ-01-1288, DZ-01-1292, DZ-01-296, DZ-01-1717
**DZ-01-1277**	Yes	DZ-01-1277, DZ-01-328
**DZ-01-1310**	No	DZ-01-1310, DZ-01-1057
**DZ-01-1312**	Yes	DZ-01-1312, Karadebi-T-3
**DZ-01-1336**	No	DZ-01-1336, DZ-01-1986, DZ-01-1091
**DZ-01-136**	Yes	DZ-01-136, DZ-01-348, DZ-01-1670, DZ-01-1671B
**DZ-01-192**	No	DZ-01-192, DZ-01-485, DZ-01-272
**DZ-01-252**	Yes	DZ-01-252, Adonis-T-9, Balemi-T-49, Rubicunda-T-17
**DZ-01-979**	No	DZ-01-979, DZ-01-1953
**DZ-01-392**	Yes	DZ-01-392, DZ-01-974, DZ-01-1014
**DZ-01-527**	No	DZ-01-527, DZ-01-1403A, DZ-01-530
**DZ-01-647**	No	DZ-01-647, Viridis-T-109
**DZ-01-2142**	No	DZ-01-2142, DZ-01-2054
**DZ-01-1556_1**	No	DZ-01-1556_1, DZ-01-1496, DZ-01-1556_2
**DZ-01-1313**	Yes	DZ-01-1313, DZ-01-576
**DZ-01-1580**	Yes	DZ-01-1580, DZ-01-146, DZ-01-165, DZ-01-855, DZ-01-306, DZ-01-759, DZ-01-866, DZ-01-1868
**DZ-01-297**	No	DZ-01-297, DZ-01-1551
**DZ-01-1512**	No	DZ-01-1512, DZ-01-1640A
**DZ-01-1602**	No	DZ-01-1602, DZ-01-300A
**DZ-01-320**	No	DZ-01-320, DZ-01-1676B
**DZ-01-504**	No	DZ-01-504, DZ-01-553
**DZ-01-1687**	Yes	DZ-01-1687, Magna-T-24
**DZ-01-558**	No	DZ-01-558, DZ-01-2044
**DZ-01-667**	No	DZ-01-667, DZ-01-691
**DZ-01-1100**	No	DZ-01-1100, DZ-01-1102

Supplementary Table 2: Heritability estimates for agronomic and grain morphometric traits

Category	Trait	Transformation	Cullis heritability	BLUP-BLUE regression heritability	Additional datapoints removed
Field	Days to heading	none	0.55	0.53	All Alem Tena data removed
Field	Days to maturity	none	0.23	0.19	All Alem Tena data removed
Field	Grain filling period*	NA	0.17	0.14	All Alem Tena data removed
Field	Plant height	none	0.66	0.64	
Field	Culm length	none	0.55	0.52	
Field	Panicle length	none	0.73	0.71	
Field	Peduncle length	natural log	0.43	0.38	
Field	First culm internode length	natural log	0.31	0.29	
Field	Second culm internode length	none	0.26	0.22	
Field	Shoot biomass	none	0.33	0.29	
Field	Grain yield	natural log	0.46	0.42	
Field	Harvest index**	NA	0.45	0.42	
Field	Lodging index	none	0.36	0.28	No data recorded for Chefe Donsa
Field	Panicle form	NA (ordinal)	0.92	0.92	
Field	Grain colour	NA (binary)	0.97	0.97	
MARVIN	Thousand Grain Weight***	none	0.80	0.79	Chefe Donsa plot 35, Chefe Donsa plot 146
MARVIN	Mean grain area	none	0.89	0.89	
MARVIN	Mean grain width	none	0.90	0.90	
MARVIN	Mean grain length	none	0.87	0.86	

*GFP is calculated as DTM - DTH, so BLUPs for GFP were calculated as:DTM ~ DTH + location + (1 | block_by_location) + (1 | redundancy_group)

**Harvest index is calculated as grain yield / shoot biomass, so BLUPs for harvest index were calculated as: log(grain_yield) ~ log(shoot_biomass) + location + (1 | block_by_location) + (1 | redundancy_group)

***Two anonamously low datapoints removed. These were sufficient to generate a spurious association

Supplementary Table 3: Significant regions from k-mer-based GWAS

Trait	Chromosome	Region start	Region end	Size (kb)	Correlation of ref kmers	Number sig kmers	Number tef genes	Figure 6 label
grain_colour	1B	7670000	8460000	790	positive (brown)	2734	114	1
riboflavin	1B	20890000	22620000	1730	negative	1726	174	NA
riboflavin	2A	3710000	4740000	1030	negative	12351	126	NA
grain_width	3B	7170000	7200000	30	positive	1953	4	2
EPOD	3B	7180000	7200000	20	negative	1865	2	2
grain_colour	3B	7180000	7200000	20	positive (brown)	2407	2	2
panicle_morphology	3B	28030000	28100000	70	negative	921	13	NA
grain_colour	4A	14480000	15790000	1310	positive (brown)	8869	135	3
grain_width	4A	14600000	14830000	230	positive	3260	24	3
EPOD	4A	14640000	15790000	1150	negative	24830	118	3
EPOD	4A	15570000	17570000	2000	positive	26134	162	4
PFCC	4A	24250000	24370000	120	positive	9529	19	NA
grain_width	4A	24290000	24370000	80	positive	6521	10	5
grain_colour	4A	25950000	26030000	80	positive (brown)	1066	5	6
grain_width	4A	25960000	26030000	70	positive	768	4	6
succinic_acid	4A	31860000	31900000	40	negative	2417	11	NA
riboflavin	4B	4590000	5010000	420	negative	3288	75	NA
EPOD	4B	13340000	14030000	690	negative	60639	81	7
grain_colour	4B	13340000	14030000	690	positive (brown)	64749	81	7
grain_width	4B	13340000	14050000	710	positive	29576	82	7
grain_area	4B	13600000	14030000	430	positive	23978	54	7
caprylic_acid	4B	13700000	13930000	230	negative	1325	21	NA
grain_width	4B	14060000	14320000	260	negative	1751	25	8
grain_area	4B	14130000	14320000	190	negative	2324	17	8
grain_colour	6A	1160000	1200000	40	negative (white)	2492	6	9
KOROG	7A	25780000	26140000	360	positive	9519	26	NA

Supplementary Table 4: Significant regions from SNP-based GWAS

Chr	Trait	Trait Type	Coordinate	Model	p-value	Minor allele frequency	p-value	kGWAS overlap
1B	Rutin	Metabolite	18755483	Blink, CMLM, ECMLM, FarmCPU, MLMM, SUPER	5.07E-11	0.21	0	No
2A	Lodging index	Agronomy	24309274	Blink, MLMM	1.37E-08	0.49	0	No
2A	Panicle morphology	Agronomy	25784710	Blink, MLMM, SUPER	3.64E-07	0.09	0.01	No
2A	Panicle morphology	Agronomy	28192436	Blink, MLMM	1.39E-11	0.09	0	No
4A	EPOD	Metabolite	14640289, 14653651	FarmCPU, SUPER	2.01E-07, 8.03E-07	0.42	0	Yes
4A	EPOD	Metabolite	15580200	Blink, SUPER	9.33E-07	0.36	0.02	Yes
4A	PFCC	Metabolite	24289851	Blink, MLMM, SUPER	6.76E-08	0.27	0	Yes
6A	Rutin	Metabolite	18730097	Blink, FarmCPU	2.16E-07	0.37	0	No
7A	KOROG	Metabolite	25918963	Blink, CMLM, ECMLM, FarmCPU, MLMM, SUPER	2.59E-07	0.37	0.01	Yes
7A	KOROG	Metabolite	26131606	CMLM, ECMLM	5.69E-07	0.43	0.01	Yes
7A	Rutin	Metabolite	26448021	Blink, FarmCPU	1.04E-08	0.18	0	No
9A	DHS	Metabolite	20029232	Blink, FarmCPU, MLMM	8.87E-07	0.13	0.04	No
10A	Grain length	Agronomy	8599074	Blink, SUPER	1.50E-14	0.39	0	No

Supplementary Table 5: Heritability estimates for selected metabolite traits.

Category	Trait	Transformation	Cullis heritability	BLUP-BLUE regression heritability	Additional datapoints removed
Metabolite	HPLA	natural log	0.82	0.81	
Metabolite	DHS	natural log	0.71	0.67	
Metabolite	ALA	natural log	0.65	0.62	Alem Tena plot 210, Chefe Donsa plot 92
Metabolite	apigenin	natural log	0.48	0.40	
Metabolite	arachidic_acid	natural log	0.45	0.40	
Metabolite	ascorbic_acid	none	0.29	0.28	
Metabolite	heme	square root	0.61	0.60	
Metabolite	jasmonic_acid	natural log	0.56	0.51	
Metabolite	KOROG	square root	0.94	0.94	
Metabolite	OG	none	0.83	0.82	
Metabolite	PFCC	square root	0.71	0.70	
Metabolite	quercitrin	none	0.84	0.84	
Metabolite	riboflavin	square root	0.60	0.60	Debre Zeit plot 15
Metabolite	rutin	none	0.87	0.87	
Metabolite	succinic_acid	natural log	0.84	0.84	
Metabolite	EPOD	natural log	0.88	0.87	
Metabolite	glutathione	square root	0.52	0.50	
Metabolite	caprylic_acid	natural log	0.74	0.73	
Metabolite	mevalonic_acid	square root	0.70	0.68	
Metabolite	ESA	square root	0.66	0.63	
Metabolite	propionic_acid	natural log	0.82	0.82	

Supplementary Table 6: Details of field sites used for field phenotyping in Ethiopia.

Location and Climatic Features	Field sites		
	Alem Tena	Debre Zeit	Chefe Donsa
**Latitude**	8° 20' N	8° 44’ N	8° 57’
**Longitude**	38° 57' E	38° 58’E	39° 16’E
**Altitude (m.a.s.l.)**	1575	1900	2435
**Rain falls (mm)**	689	984	1020
**Soil type**	Light soil	Black vertisol	Black vertisol
**Maximum mean daily temperature (°C)**	29.46	26.84	20
**Minimum mean daily temperature (°C)**	15.29	11.39	8
**Agroecology type**	Lowland	Mid altitude	Highland

Supplementary Table 7: Phenotype definitions and methodologies

Category	Trait	Description
**Qualitative**	Basal stalk colour	Colour of the base of the seedling stalk. Scored as red (R), purple (P), greenish (G) and yellow-white (Y.w).
	Grain colour	Visual classification of grain colour after threshing. Scored as brown or white.
	Panicle colour	Visual classification of panicle colour (largely attributable to lemma colouration). Recorded at anthesis and scored as yellow-white (Y.w), greenish (G), red (R), purple (P), or variegated (V; colours as before, denoted by a primary colour plus a secondary colour either beginning from the middle of the panicle (m) or just present at the tip (t)).<br>e.g. V(Y.w+P(m)) denotes a panicle that is primarily yellow-white but with purple tints beginning from the middle of the panicle.
	Panicle form	Visual scoring of panicle morphology. Recorded at anthesis and scored as very loose (1), loose (2), semi-compact (3), or compact (4).
**Phenological**	Days to heading (days)	The number of days from seedling emergence until 50% of the plants in a row have ~5 cm of panicle emerged on the main tiller.
	Days to maturity (days)	The number of days from seedling emergence until 90% of the plants in a row attain physiological maturity. Maturity is judged as the point at which aerial biomass has dried and changed to a yellow, straw-like colour.
	Grain filling period (days)	Days to maturity minus days to heading.
**Agro-morphology (five randomly sampled plants)**	Plant height (cm)	Length of the plant from ground level to the tip of the panicle.
	Culm length (cm)	Length from ground level to the node where the first panicle branch starts.
	Peduncle length (cm)	Length from the flag leaf node to the node where the first panicle branch starts.
	Panicle length (cm)	Length from the node where the first panicle branch starts to the tip of the panicle.

	First culm internode (cm)	Length of the first internode, from the ground to the first node.
	Second culm internode (cm)	Length of the second internode, from the first to the second node.
**Agro-morphology (whole plot)**	Shoot biomass (g/row)	The dry weight of the above ground biomass per row before threshing .
	Grain yield (g/row)	The dry weight of threshed grains per row.
	Harvest index	The ratio of grain yield to total biomass.
	Lodging index	Calculated according to Caldicott and Nuttall (1979) who defined lodging index as:<br>*Lodging index = ∑ ((lodging degree * percent of plot affected) / 5)*<br>Where 'lodging degree' varies from 0 for completely upright to 5 for completely lodged (flat against the ground). The calculated values for lodging index therefore vary between 0 (no lodging) and 100 (complete lodging).
**Grain morphology**	Grain area (mm^2)	The average grain area, calculated by MARViN grain analyser from a sample of 0.075-0.085 g grain.
	Grain width (mm)	The average grain width, calculated by MARViN grain analyser from a sample of 0.075-0.085 g grain.
	Grain length (mm)	The average grain length, calculated by MARViN grain analyser for a sample of 0.075-0.085 g grain.
	Thousand grain weight (g)	Extrapolated mass of 1000 grains, calculated by MARViN grain analyser for a sample of 0.075-0.085 g grain (mean of 262 grains).
