# Supplementary material for: Population genomics uncovers loci for trait improvement in the indigenous African cereal tef (Eragrostis tef)
Source: Commun Biol. 2025 May 26;8:807. doi: 10.1038/s42003-025-08206-5 (PMC12106829; doi:10.1038/s42003-025-08206-5)
Supplement: Supplementary file 3 — Description of Additional Supplementary Materials [file 42003_2025_8206_MOESM3_ESM.pdf]

Description of Additional Supplementary Files

File name: Supplementary Data 1

Description: Matrix of shared kmer state rates between all pairs of accessions

File name: Supplementary Data 2

Description: Loci for minimal SNP panel plus observed genotypes for 150 non-redundant accessions

File name: Supplementary Data 3

Description: Details of 183 selected metabolites

File name: Supplementary Data 4

Description: Scaffold from the Tsedey genome assembly by Cannarozzi *et al.* (2014) containing the A genome copy of the tef *TT2* orthologue (Et_4A_032842). The gene model is highlighted with background colours in the sequence. The LTR insertion is shown in the green text.

File name: Supplementary Data 5

Description: Scaffold from the Tsedey genome assembly Cannarozzi *et al.* (2014) containing the B genome copy of the tef *TT2* orthologue (Et_4B_039404). The gene model is highlighted with background colours in the sequence. The LTR insertion is shown in the green text.

File name: Supplementary Data 6

Description: Details of accessions used in the study

File name: Supplementary Data 7

Description: Original field trial design; Updated field trial design based on definition of redundant accession groups

File name: Supplementary Data 8

Description: Raw field and grain phenotypic data. The main trait columns are highlighted in blue
