# Supplementary material for: Population genomics uncovers loci for trait improvement in the indigenous African cereal tef (Eragrostis tef)
Source: Commun Biol. 2025 May 26;8:807. doi: 10.1038/s42003-025-08206-5 (PMC12106829; doi:10.1038/s42003-025-08206-5)
Supplement: Supplementary file 8 — Supplementary Data 5 [file 42003_2025_8206_MOESM8_ESM.docx]

## >lcl|scaffold2714_Reverse_complement

TGCTTAATTTCACAGCCTGTAAATTCGAGAAGGAAGAAATTAGAACAATATTATTCAAGTGAGCAGGGGCATAGAAAAGGAATAAAGAATCGGATGTATCATGCTTTTGAGATAATATTTACGAAATGGGGCTTAATTTCATGCTGCAACTGCATTTGTCTGCAGAAACCCTATAGGAAGAAAATACCCATGACCCCATGATGCATGGAAAGGATCTAGTTGTAATGCAGTGTTAACCTAATTTCACTCTTGACAAGAATGACAGAGCAACGTATGAAACAAATTATCAGCATAGTAGTTGCAAATCAGCAACAGCACAAATGTGATTGTAAGGAACCATATGAATTGGAAGTGAGCACAATACGAAACAACCCTTTATTTCTTCCTCAGTGTTTATGCAGCATATGTGTTTGCAAGGTAACGTGAAGCTTGTTAAACTACTAACTAAACAAATACAACTGTTATTGTTTTTTATCCCTTTTTCACAGTTCATAATAAACTTGTGTTTCCAGCTAGCAGAGCTTAATTCAAGGGCCTTCTTTATTTCATTAAATTTATGACCTTCAGGCACTAGCTGTTGTGATCTAGCATTAGCCTGAGACGGGCAATACCTTTCTCTCAAGCTGCTTGTCTTCAGAAAGCCATGCTTTTGCTGTGAGAAGATCCACCTGTTGCTTTGCCAATACCTGAAAAAGGGGACAAATTTCTTATATGCCATCAAAAGGTTATATGGCACGACATCCATGATCGATTGTGCCACTTGGAGCCACAATGTCAGTGGTTAAGTGAGGAGTTGCAGAAGTGATGCCGCATGTGAGTATTGAAACTTTAGATCGATGGCCATATGCTATTTTATTAATGATCATATCACATCAATCAGTTTTCGACTCAATAATGGTGCAATGCTGGCATTCATAGCAAGAAAAAACACATTTGCTCACTTATGTGGCCAATATTTTGTGACTTGCCTATCAAATAACTACAAATAGGCCAGAGGCCAATGTTTGCCACAGCTTTGCTCAATATATTTGAGTCTTTCTCATGGTCTATATTGTGAGCTGATGGAAGTATGTCTTCTTACATGCAAAGCACATGTGCAGTTAGGCTTTTTTTCCCCTGCACAATATGTGCAAAATAGAAACAAACCAACGCTATGCCGCTATCCATAAGACCCTAAAAAAATTATTCAAACAGAAAAACAACAAATTGGTACTATCATCTTGTTTACCAGTGATATGGTTCTAATAATGTAAATCGCAGAACTTGTTTAAGTGTGCCAGGACAGGGCTGGATTCATCAAACAAATGAGTAAGTGCAGCCATTATATCTGGTTGGGCTAGAAATCAATTCTGGTCAGGAACCAACTTCAAATTGGCATGCAATAAGGTGGACAGAACCTTGTTATTAGTTTGAACAGCGGTATATGTACCGGTAATCAAAACAACTGACCATCAAAGTACTTCTGCTACCTTTTTTTGTTTTGTTTATTTGATTGACTACTGTAAGTCAAAAGCTCCAAGCGATATTAACAAAAGCCAGTAAACAAAAATAACAAAGAAATGAGTATATTACTTTGTCAAGAGGTATCTCCTTTATATGTTTGACTTTCAATTCAACAGATATTTCGCTCATGTCAGCCAAGTGACCCTTGACGGTGAGTGTCTCCGGAAGCTTGCCATGGTACCTTCCAGATACACATAATGGATATTTCGCCGAAATGTCTGGAATATACTCAGAATCCACCTAATTGGAAAAGGAAAAGCTTGACTGTAAGAAACATAGCTGTTCGGATGCAGGAGCTAACTGCAAAGATATACCTTGACAATGGTCTAATAATATATTCATCGGCAAGGTGCTCCTGTCTAATAATATGGTACTCATGGTTTCAGAATGGTTTAATATCACTCTTTGCTATCCTCTACTCCTAGTGACAGAAGCTGACTGATGAGTAGTTAAAATATATGATTCAACAGGGATGAATGACTCAATTACAAATTGTTGTGGATAGTTTGTGCCTCGTTGGTCAGTTAAAAAGGTAGTAATTCTGAAATGAGGCAGTGTCACATTGCATTTAGTAAATCTAATTGTAACACAAAAAAACTAAAGAAAAAGGTGAATGATCTCTAAAAGGCAGCAACAGAAAGTGAAAGTAAAAAACTCACTTCAAATTCTTGTATATATTTCATGCCATCAATGGAAATATTTGTCACTATTGTGCTCAAAGCTTTCNNNNNNNNNNNNNNNNNNNNNNNNNNNNNNNNNNNNNNNNNNNNNNNNNNNNNNNNNNNNNNNNNNNNNNNNNNNNNNNNNNNNNNNNNNNNNNNNNNNNNNNNNNNNNNNNNNNNNNNNNNNNNNNNNNNNNNNNNNNNNNNNNNNNNNNNNNNNNNNNNNNNNNNNNNNNNNNNNNNNNNNNNNNNNNNNNNNNNNNNNNNNNNNNNNNNNNNNNNNNNNNNNNNNNNNNNNNNNNNNNNNNNNNNNNNNNNNGGTTAAAAAAAAAAAAAAAGAAGAAGAAGAAACTTCAGCAATAAAATTTTGACTAAGTTTAACATGCTCAAAATTCTGTATAATCCATTTAAGTCAAGACAATCAATAAGTGTTTAATTTAAATGAACCGGATTTGAGTATTTTATTATTATCCCTTTGACCCAAAGAGGAGGATTGAACGAAAATTAAAATAGATATTGTCCATTGTCTATACGTAAAATATGGCAATCTATTAATTTAAATGCCTATTTTTTTGTTCACCCAACTGATAGATTTTCTTCATCAACGGAACGAAGTTACTCTGACTTTTGCTCTACACATGAGCTGGATCATCTAGGCAACACCCCCCCCCCCAAAAAAAAAAAAAACTATAATTAAGAAGACAAATATTAGACCATAACGCTTAAGTGAAATTCCAAATTCCATCAAGAGATACAGTATAGAACAGGAGAGAGGCCCCATCAATTTTTCAAGACAAAAGGAAGAAACATGTTAATACCTGTATCAAACGTAGCATCGTAGTGACCCTTCCCAATTGATGCCAACATCCTCAAGAAATAGTGGTTGCAAAATGAACCTAACATGTTGTTCCAGAACAAATACAAACAATCCGATAATGCATTCAACTCATAAAACAGCAAAATCAAATTTATGCCCCATCACATCCAAAAAAGGTTAAACTATATTGTTGCTCTTCAGTCCATTCACAAAATATTTAAAGAAATTAGCTTAAGAACAGATATTCACAGAGTGAGATACATAAGTCAATGATTCAAAAGAAATTAATTTAACAACAGATATTTGCAGTGAGGTACATAAGTCAGTATGTACAAATGCCGAAATTATTTACTGAGAAATCTCAGACTTCAGGAACAATTTCGTTAACTTAAGAATGGTACATGACTCTTAAAAATTAGTGGAGAATTGGCTTGAGACAAGGACTTAGTTTGATCAGTATGGTACATGACTCTCATGATACATAACCAGATATTCTATGAGATATTACCTAGTCCAAAAGTAGAAATTCGAGGAGCCTTAGATCCTCTACTCATGAGCTGATTTTTCATGGAATGGCAGATATTCCGTTCATCGTCAACTGAGCCATCGGTAACAAGATAGATTTGTGGCAGAGCATCATGAGAGTTTGACAACAAAGCCATTGCCTTACACCCAAAAAAGAGGGGAATACCATGAGTGACGATATTCCTATGACAATGACAACTAATATAATCTGCTAAATACCAAAATACCACACCTCACTCAAAGGATGCATGATGTCAGTACCACCCTTCGCAACAAAATTTAAATTCATCCATTCAATAGCACTTTCCACTGTTTTCTCGTTTACTTGCTCCAAAGATGATGAGAAAGAGTAAAGCTCATCATTGAATGATATTATGTTGAAGTAATCTCCTTGCACAAGATCAGAGAGAGCAGTGGACATGGCGTTCTTCACATTCTCAAGAGGCTTTCCTTGCATGCTTCCACTTGAATCAACAACAAACACAACTGCTTTTCTGAAGACCTAACAGAGAACTCAAATGAGATGAATTACCACAAAAGGAAAAAGTTATGAAACAAACAAGGAAGGGGTAACAAACGGATCTTATTTCAGGTTGAGTCAATATTGATAACATCATTATTAGAAAAGACTCACAATATGCCCAACACTGGTCAAGGGGCAAACGATAAACATACAGTTACAAAGATACCAACTAGTCTGAGAATAATCTGACAGTCAGAAAGTTAGAATTGTTTATAATTAAAAAGGAATAACTTGTTTTAGTGACAACAGTTTTCAATCACCAGAAGATTATCCATTGACATAACTTTTTACTCATAATGCTAAGACACTAAGAAGATAACCAAAACAGAGCAACTACCTTTCTGTTAGCATTATTTCCAGGTAAAAGAAAAATGCTGAACATGTCTCTGTCATCATAATCACGCAATGTTGAGGGCTGCACTAGAACTCCACCAGACAAGTCGCCTGAGTAAACCTATAGGCATGAACAAACAAAATCAGGAGAAGTGGATGGAACGGTAAGTCATAAATGAACTCCATAAACCCTCCATTTCCTTCAATTATTTTTAAATGCTAATCATTAAAGAGTATAATGAAAGCTTTCACACTGTAGTAAACTTAAAGACGTCAAATAAACCTACAAGTTGTCATAACTACTTTTTATATAAAAAAGGGAGATATCTTGTATGTACATGGCATGCTAAAGGCATTATTGTCCCTATGAGAGAACGAGTTAACAGCTATCACTACAGAGAATAAAAATGCTCAGCTATACTGGAACTGGACAGGCATAGCATAGCTTTTCTTCACTGTGTTCAAGTAAAATGGTAGTATGATTATCGCTAGCATAGTTGTTACACACAAATCCCACAGGCCAGTACATGCGACAATAATCATTCATGAGAAGTTTATCAGTAGTATATAAGTACTTCCAACCTGGCATTTTATGTAAACAAAACCCACTATGCTTTTGATGTTTTGAATGGTCGAAAAAGTAGTGCTGACAACAAGCACGAGCAATCGAGCACAAACGGAAACATAAAAAAATAGTTAAAGGCTATAAAAAATGTTGGACAGCTTACACTGTATGCGAAGGTAAAATCTTTGATAGACCAATTCTCAACAACTGCATCGTGCAGGAATGACAATTTCTCATCTTGCCTACCTTTTTCCTGAAATCGTAACAGAAGATCATGCTATAAATATATGGAGCAGAGGTTCTCCTAAAAGAACTAACAAAAGCAGTGAATACTTTATTACCTTCAAGGGATGGCTTGTCCCCTGCAATAAGACCTCTTTACTCACACCAGTATTCACAGTCAACCGTATTTTCTCCTTCTTCGTGAAAACTTTTGGCAAAGGGTTCACAAACGATGGGAATCGAAAAGGTACTTCGACAGAAAACTGCCCGTTGTCATATAGTAAATTCTGTGACCATCTAATAGTGGCAAAAATGTCTTCTCCACCTCCAACCTTGATGGTGTAAAAGAAATTAGTTAAAAAAGACATGCAGATGACGAAATCTTGAAACATTGCAAGTAGCAATTGGCACCAATTAAGAGTATGTGACAACCACCTGTGGTATTGTGAGGGAAAACAATTCCTGTTTTAAAAGACCCCCACTCTCAGGCTTCGAAATCTTCATTGCACTGTCATCCTCCACATCAATTACATGAGTACTGTATGATTTTTTTCCAACAGTAACCTCAGCGCCTAGAATTGAACCCTATTAGTGAAATTATTCTTAGATTTAGGCAGGCGATAAATTTCAGGATGAATTGTTTCAGAAAATGTAAGCATTGAAAATTACTGCCGCATAAAAATACAGAGTGACAACAGTTTTCAATCACCAGAAGATTATCCATTGACATAACTTTTTACTCATAATGCTAAGACACTAAGAAGATAACCAAAACAGAGCAACTACCTTTCTGTTAGCATTATTTCCAGGTAAAAGAAAAATGCTGAACATGTCTCTGTCATCATAATCACGCAATGTTGAAGGCTGCACTAGAACTCCACCAGACAGGTCGCCTGAGTAAACCTATAGGCATGAACAAACAAAATCAGGAGAAGTGGATGGAACGATAAAACCTCCATTTCCTTCAATTATTTTTAAATGATAATCATTAAAGAGTATAATGAAAGCTTTCACACTGTAGTAAACTTAAAGACGTCAAATAAACCTACAAGTTGTCATAACTACTTTTTATATAAAAAAGGGAGATATCTTGTATGTAAATGGCATGCTAAAGGCATTCTTGTCCCTATGAGAGAACGAGTTAACAGCTATCACTACAGAGAATAAAAATGCTCCGCTATACTGGAACTGGACAGGCATAGCATAGCTTTTCTTCATTGTGTTCAAGTAAAATGGTAGTATGATTATCGCTAGCATAGTTGTTACACACAAATACCACAGGCCAGTACATGCGAAAATAATCATTCATGAGAAGTTCATCAGTAGTATATAAGTACTTCCAACTTGGAATTTTATGTAAACAAAACCCACTATGCTTTTGATATTTTGAATGGTCGAAAAAGTAGTGCTGACAACAACCACGAGCAATCGGGCACAAACGGAAACATAAAAGAATAGTTAAAGACTATAAAAGAATGTTGGACAGCTTACACGGTATGTGAAGGTAAAATCTTTGATAGACCAATTCTCAACAACTGCATCGTGCAGGAATGACAATTTCTCATCTTGCCTACCTTTTTCCTGAAATCGTAACAGAAGATCATGCTATAAATATATGGAGCAGAGGTTCTCCTAAAAGAACTAACAAAAGCAGTGAATACTTTATTACCTTCAAGGGATGGCTTGTCCCCTGCAATAAGACCTCTTTACTCACACTAGTATTCACAGTCAACCGTATTTTCTCCTTCTTCGTGAAAACTTTTGGCAAAGGGTTCACAAACGATGGGAATCGAAAAGGTACTTCGACAGAAAACTGCCCGTTGTCATATAGTAAATTCTGTGACCATCTAATAGTGGCAAAAATGTCTTCTCCACCTCCAACCTTGATGGTGTAAAAGAAATTAGTTAAAAAAGACATGCAGATGACGAAATCTTGAAACATTGCAAGTAGCAATTGGCACCAATTAAGAGTATGTGACAACCACCTGTGGTATTGTGAGGGAAAACAATTCCTGTTTTAAAAGACCCCCACTCTCAGGCTTCGAAATCTTCACTGCACTGTCATCCTCCACATCAATTACATGAGTACTGTATGATTTTTTTCCAACAGTAACCTCAGCGCCTAGAATTGAACCCTATTAGTGAAATTATTCTTAGATTTAGGCAGGCGATAAATTGCAGGATGAATTGTTTCAGAAAATGTAAGCATTGAAAATTACTGCCACGTAAAAATACAGTAGCAAATATACATTTAACAGACATCATAATGGCATGCACAACTCTAGATAGTGAAGAAGTGGTAAACCATGTGCATTAATGGCTACCTAATTCAGACTGATCCTCTTATAGTTGTTAACATCAAGTGGCCAAACTTAGACCAAAAAAAAAAAAGAGTTGCTATTTTCAAGATCAAAAGAAACAGATCTTGCTCGTGCACAGCTCTGAGGTTCCCTCTCTTAAGGCCATTTGAACCAACACCAGAATGAAAAAGGGGATCACATCAAACCCAACAAATTTTGGCACTCCTCTAGCATCAAAATCGTAATACTTTGCACAGCTTACATTTCTCCAAACAGCATTTTTGGATGTATATTCTATTTGTACAATTAACAGATAATACTACTGTGCACCGGTAGAAGGAAAGGAGAATGAAAGACCCAAATGCTAAAGCCTTTACTGGACAAGCGTACAAAGGCAGCGAACCTTTTGCAAGTCAAAAGTTCAATGTTTCTTTCGTGCTGCTTATCTTTTAGCATGGAAAGCAAGGAATTGGACTTGCTTTGTATCGAAACTATGCTGGAATCCACTGCAAGCGGAGGCAGAGCTGTGCCCTTCACCGCCGGACAAGAACAAATGAGCATTGTCGTGCAGCACCAGTGCTCCAACCAAATGCAGGTGGGGAATAATTGCATAATGACAAAGTAGTATGTCCTGATTTGGGCAGTGCAAAGCGGACGACAATGCACAGCGAATTCATGGGCCCCAACTACCAAACAGAAATCAAGTTGCCGGGGGAACAATGGCAAATCAATCGTACCGTGGCCTACCATGCCAAATTCAGCACAACACCGGAAGCAGCACGCTAAAGAGTAAGGAAATTGAATTGAGATCGCACGGAAACAGCAGGGGGACGGAGAGAGGGGTGCGACCTGGTACCCCATGGGCACGACGACGCGGCAGTCGCAGGCGCGGCTGCGCGTGACGCAGTGCACCCACCACCGCGCGCGGAGGGTGACCTCCGCGCGCGCANNNNNNNNNNNNNNNNNNNNNNNNNNNNNNNNNNNNNNNNNNNNNNNNNNNNNNNNNNNNNNNNNNNNNNNNNNNNNNNNNNNNNNNNNNNNNNNNNNNNNNNNNNNNNNNNNNNNNNNNNNNNNNNNNNNNNNNNNNNNNNNNNNNNNNNNNNNNNNNNNNNNNNNNNNNNNNNNNNNNNNNNNNNNNNNNNNNNNNNNNNNNNNNNNNNNNNNNNNNNNNNNNNNNNNNNNNNNNNNNNNNNNNNNNNNNNNNNNNNNNNNNNNNNNNNNNNNNNNNNNNNNNNNNNNNNNNNNNNNNNNNNNNNNNNNNNNNNNNNNNNNNNNNNNNNNNNNNNNNNNNNNNNNNNNNNNNNNNNNNNNNNNNNNNNNNNNNNNNNNNNNNNNNNNNNNNNNNNNNNNNNNNNNNNNNNNNNNNNNNNNNNNNNNNNNNNNNNNNNNNNNNNNNNNNNNNNNNNNNNNNNNNNNNNNNNNNNNNNNNNNNNNNNNNNNNNNNNNNNNNNNNNNNNNNNNNNNNNNNNNNNNNNNNNNNNNNNNNNNNNNNNNNNNNNNNNNNNNNNNNNNNNNNNNNNNNNNNNNNNNNNNNNNNNNNNNNNNNNNNNNNNNNNNNNNNNNNNNNNNNNNNNNNNNNNNNNNNNNNNNNNNNNNNNNNNNNNNNNNNNNNNNNNNNNNNNNNNNNNNNNNNNNNNNNNNNNNNNNNNNNNNNNNNNNNNNNNNNNNNNNNNNNNNNNNNNNNNNNNNNNNNNNNNNNNNNNNNNNNNNNNNNNNNNNNNNNNNNNNNNNNNNNNNNNNNNNNNNNNNNNNNNNNNNNNNNNNNNNNNNNNNNNNNNNNNNNNNNNNNNNNNNNNNNNNNNNNNNNNNNNNNNNNNNNNNNNNNNNNNNNNNNNNNNNNNNNNNNNNNNNNNNNNNNNNNNNNNNNNNNNNNNNNNNNNNNNNNNNNNNNNNNNNNNNNNNNNNNNNNNNNNNNNNNNNNNNNNNNNNNNNNNNNNNNNNNNNNNNNNNNNNNNNNNNNNNNNNNNNNNNNNNNNNNNNNNNNNNNNNNNNNNNNNNNNNNNNNNNNNNNNNNNNNNNNNNNNNNNNNNNNNNNNNNNNNNNNNNNNNNNNNNNNNNNNNNNNNNNNNNNNNNNNNNNNNNNNNNNNNNNNNNNNNNNNNNNNNNNNNNNNNNNNNNNNNNNNNNNNNNNNNNNNNNNNNNNNNNNNNNNNNNNNNNNNNNNNNNNNNNNNNNNNNNNNNNNNNNNNNNNNNNNNNNNNNNNNNNNNNNNNNNNNNNNNNNNNNNNNNNNNNNNNNNNNNNNNNNNNNNNNNNNNNNNNNNNNNNNNNNNNNNNNNNNNNNNNNNNNNNNNNNNNNNNNNNNNNNNNNNNNNNNNNNNNNNNNNNNNNNNNNNNNNNNNNNNNNNNNNNNNNNNNNNNNNNNNNNNNNNNNNNNNNNNNNNNNNNNNNNNNNNNNNNNNNNNNNNNNNNNNNNNNNNNNNNNNNNNNNNNNNNNNNNNNNNNNNNNNNNNNNNNNNNNNNNNNNNNNNNNNNNNNNNNNNNNNNNNNNNNNNNNNNNNNNNNNNNNNNNNNNNNNNNNNNNNNNNNNNNNNNNNNNNNNNNNNNNNNNNNNNNNNNNNNNNNNNNNNNNNNNNNNNNNNNNNNNNNNNNNNNNNNNNNNNNNNNNNNNNNNNNNNNNNNNNNNNNNNNNNNNNNNNNNNNNNNNNNNNNNNNNNNNNNNNNNNNNNNNNNNNNNNNNNNNNNNNNNNNNNNNNNNNNNNNNNNNNNNNNNNNNNNNNNNNNNNNNNNNNNNNNNNNNNNNNNNNNNNNNNNNNNNNNNNNNNNNNNNNNNNNNNNNNNNNNNNNNNNNNNNNNNNNNNNNNNNNNNNNNNNNNNNNNNNNNNNNNNNNNNNNNNNNNNNNNNNNNNNNNNNNNNNNNNNNNNNNNNNNNNNNNNNNNNNNNNNNNNNNNNNNNNNNNNNNNNNNNNNNNNNNNNNNNNNNNNNNNNNNNNNNNNNNNNNNNNNNNNNNNNNNNNNNNNNNNNNNNNNNNNNNNNNNNNNNNNNNNNNNNNNNNNNNNNNNNNNNNNNNNNNNNNNNNNNNNNNNNNNNNNNNNNNNNNNNNNNNNNNNNNNNNNNNNNNNNNNNNNNNNNNNNNNNNNNNNNNNNNNNNNNNNNNNNNNNNNNNNNNNNNNNNNNNNNNNNNNNNNNNNNNNNNNNNNNNNNNNNNNNNNNNNNNNNNNNNNNNNNNNNNNNNNNNNNNNNNNNNNNNNNNNNNNNNNNNNNNNNNNNNNNNNNNNNNNNNNNNNNNNNNNNNNNNNNNNNNNNNNNNNNNNNNNNNNNNNNNNNNNNNNNNNNNNNNNNNNNNNNNNNNNNNNNNNNNNNNNNNNNNNNNNNNNNNNNNNNNNNNNNNNNNNNNNNNNNNNNNNNNCACCGGGAGGTGAGGTTGTTCTTCGAGGGCGCTGCCGCCGATGGACGTGGTGGGCAGGAATTCGGACTAGTCCACGCGAGAGGCATGTTGCCCTGTCTCTGCAGGAATTCTGATGGTCCACGCGAGAGGCATGTTTGCTGTCTTTGCAGTTTGCATTCAAAGAGTAAACCCTGGGTTTAATAGTGTTTGTCTCACAAAATTATAGTTATTTTATCTTATACTATCATTTAATATTATATGTTATTGTTATATTGTTTTATAAAACTCCACCTTTTTTAGCAATGGCCATAAATATGACATGTGAATATATCTTTGTGCTTGTCTTGTGTGATACAATTTTTGTAATAATTTGATCAACGAAATCATTTAGTTGGATGCATGGCTATCAAACCTCTAAAGTAGGTAACCTTCGGCGAAGGAGAGAACGCAAGAACAGAAGACATCACCTTCGACGTGGTCGATATTGTTTATCCTTACAATGCGATCTTCGGAAGAGGATCCATCAACAAGTTCGAAGCCGCCATACATCAAGGCTATCTTTGCATGAAGATTCCGGGACCTATCTCTGTGATCAGCGTCTTCGGAGACCAGCTTCAAGCAAGAAACATTGAAAAAGGCGAAACGCCCGGAAGAAAGAACATCCACCTCGTCCGAAGCGAGTTCGAAGCCCAATCGTTCAAGATCTTCGGCTTGAAAAATTCGGCCATGGCATACCAAAGATGCATGAGCCATGTCTACCAAGGGCATTTGCTTGGTAGCATGAGTGATTCATATTTGTAAAAACATAAAGAACTTGCAGGGGGTGTGCCCTTTTTCCTTACGAGGGTAACCCTGCGAAGGGTGTGAGGTTTTTGAACGAGGCACCTCCCAAGATTGTAATAGTTTTTAATCATTTTTATCCTTAAAAAATTCCAGAGCAATTTCCTGGAGGGCGAAGTGAAGCCTAGGCTCAAAAACAAAGTCTAGTCAAAATCGATATCAGGCAAAAGCCTAGAAACGAAGCCTCGAGCTCAAACTCGATATCAGGCAAAAGCCTAGAAAAATCGAAGGTTACACCAATAAAGCATTTGGTGCGACCTCAGTCTGAGTCAAAGTATCATGAGAAATGATACAAAGCCTACAGACAAGAGTCGAAGGGAAAGAAAAGCTAAGAAAGCTTAATTTACCCGACCATCGACAAACAAAAACCTCCGCAAGAGGAAGAAAGTGCGAGCCTTCGCATAAAAACGATTTCGATACATCAAATACGAATGCACGAAAACTTGTGATCCTCGAAGGAAACACAAAGACATCGCGTAGGAAGCATCAAAAGCTTCGAGAGATATTGAAGAAATCTTCAGTTCGGCAAAGACCTTCGAAAGATCACTCCAAAAGAAAAGCTTTATTAAGCAAACAACACTGTTTAAGAGGAACCATCATCAGGTTCCTGCTTTATAATAAGACCTAGTTCTAAAAGTATTTGACAAACAATGCTATACTCATCAAAATTATATCCACTCGAAGGCGAGTAGGGTCTCTGAAAAACTGGGTCAATAACCCCGGTCGAAGTCATTCGACCAACATGGGAAGCAGCATCAAACATATGAGGAGAATCTTCGCGCTCCACCGAAGGATTGTGACGACCGCGTTTTTCGCGTTTCTTGGCTGTGGTTTTTCGCCCAGAAGAAGAAGAAGAAGAACCACGATACTTACGCCCTCGAGAACCTCGCCTACCTCGACCACGGCGACCTTCGGAACCCTCAGATCTTGGAGAACCAAGTCTATACACACCAACAAAACTACCAGGAGGAAAAACATAGCCTTCGGTCCCTTGAACGGGGGACAAAGGGTCAGTCACTTGACAAAACAAACTCCAAAGAAGACGAACTCAAAATCTCAATAGCACCCTCGATGGGAGCCATCTGAGAAACCTCCGGAGTAGCAAACTCGCTCAGACAATAGTCAGATGAATCCTAAAGTGCATGAATCAAACAAAGATAAATCAAAGTAGCAACGAAGCATGCAAAGCATAAAATTACAGAAGTTCTCAACAAATACATGTCCGAAGGGAAAACATAGTCCCTATCCTGAAACAAAACAAAAATTGTTTATACCTCTTTTTGAGGTAGATGACCGACCGACTGCACTCGGCAACTTTCCGGAGGGCTTCGACAACAGGACGAGGTGTAGAATCGACAACAGAAAACTTGAAGCCGTCAGAAGCAATCTTCGCCCAAGCGTCGCTCCCTTCGACGTTCAAGGAATTGTAGCACCCCACCTCAAATTAATGAGGGGTTTGGCTAACCAGGATCTTGATCGGGGGATACTTGACGAAGCAAGCATTTCAAAGGACCACACACATAAGCATAAGTAAAATAATTTTACAACAAGTTTAGTTATTACATTGGTCCGACAATAAACATAGGGTACTCGGTTCGAAAGGTGGTTCTTAATACATAAAAAGAGTTTGAAATTCGATTACATGAAAACGAGCGGAAGCGAACACACGACGATATAGCTAAAGTGAATTCATGTCAGGCCATAGACACGATTTCACTCTTCATGCTCCGAAGATGGAGAAACAGGAACCACTTCAAAAGACCACTCACCTGCATAAACATTTTCAACAAAAACCCTGAGCAACTAATACTCAGCAAGTCTTACCCGTCAAATTGGCCTCNNNNNNNNNNNNNNNNNNNNNNNNNNNNNNNNNNNNNNNNNNNNNNNNNNNNNNNNNNNNNNNNNNNNNNNNNNNNNNNNNNNNNNNNNNNNNNNNNNNNNNNNNNNNNNNNNNNNNNNNNNNNNNNNNNNNNNNNNNNNNNNNNNNNNNNNNNNNNNNNNNNNNNNNNNNNNNNNNNNNNNNNNNNNNNNNNNNNNNNNNNNNNNNNNNNNNNNNNNNNNNNNNNNNNNNNNNNNNNNNNNNNNNNNNNNNNNNNNNNNNNNNNNNNNNNNNNNNNNNNNNNNNNNNNNNNNNNNNNNNNNNNNNNNNNNNNNNNNNNNNNNNNNNNNNNNNNNNNNNNNNNNNNNNNNNNNNNNNNNNNNNNNNNNNNNNNNNNNNNNNNNNNNNNNNNNNNNNNNNNNNNNNNNNNNNNNNNNNNNNNNNNNNNNNNNNNNNNNNNNNNNNNNNNNNNNNNNNNNNNNNNNNNNNNNNNNNNNNNNNNNNNNNNNNNNNNNNNNNNNNNNNNNNNNNNNNNNNNNNNNNNNNNNNNNNNNNNNNNNNNNNNNNNNNNNNNNNNNNNNNNNNNNNNNNNNNNNNNNNNNNNNNNNNNNNNNNNNNNNNNNNNNNNNNNNNNNNNNNNNNNNNNNNNNNNNNNNNNNNNNNNNNNNNNNNNNNNNNNNNNNNNNNNNNNNNNNNNNNNNNNNNNNNNNNNNNNNNNNNNNNNNNNNNNNNNNNNNNNNNNNNNNNNNNNNNNNNNNNNNNNNNNNNNNNNNNNNNNNNNNNNNNNNNNNNNNNNNNNNNNNNNNNNNNNNNNNNNNNNNNNNNNNNNNNNNNNNNNNNNNNNNNNNNNNNNNNNNNNNNNNNNNNNNNNNNNNNNNNNNNNNNNNNNNNNNNNNNNNNNNNNNNNNNNNNNNNNNNNNNNNNNNNNNNNNNNNNNNNNNNNNNNNNNNNNNNNNNNNNNNNNNNNNNNNNNNNNNNNNNNNNNNNNNNNNNNNNNNNNNNNNNNNNNNNNNNNNNNNNNNNNNNNNNNNNNNNNNNNNNNNNNNNNNNNNNNNNNNNNNNNNNNNNNNNNNNNNNNNNNNNNNNNNNNNNNNNNNNNNNNNNNNNNNNNNNNNNNNNNNNNNNNNNNNNNNNNNNNNNNNNNNNNNNNNNNNNNNNNNNNNNNNNNNNNNNNNNNNNNNNNNNNNNNNNNNNNNNNNNNNNNNNNNNNNNNNNNNNNNNNNNNNNNNNNNNNNNNNNNNNNNNNNNNNNNNNNNNNNNNNNNNNNNNNNNNNNNNNNNNNNNNNNNNNNNNNNNNNNNNNNNNNNNNNNNNNNNNNNNNNNNNNNNNNNNNNNNNNNNNNNNNNNNNNNNNNNNNNNNNNNNNNNNNNNNNNNNNNNNNNNNNNNNNNNNNNNNNNNNNNNNNNNNNNNNNNNNNNNNNNNNNNNNNNNNNNNNNNNNNNNNNNNNNNNNNNNNNNNNNNNNNNNNNNNNNNNNNNNNNNNNNNNNNNNNNNNNNNNNNNNNNNNNNNNNNNNNNNNNNNNNNNNNNNNNNNNNNNNNNNNNNNNNNNNNNNNNNNNNNNNNNNNNNNNNNNNNNNNNNNNNNNNNNNNNNNNNNNNNNNNNNNNNNNNNNNNNNNNNNNNNNNNNNNNNNNNNNNNNNNNNNNNNNNNNNNNNNNNNNNNNNNNNNNNNNNNNNNNNNNNNNNNNNNNNNNNNNNNNNNNNNNNNNNNNNNNNNNNNNNNNNNNNNNNNNNNNNNNNNNNNNNNNNNNNNNNNNNNNNNNNNNNNNNNNNNNNNNNNNNNNNNNNNNNNNNNNNNNNNNNNNNNNNNNNNNNNNNNNNNNNNNNNNNNNNNNNNNNNNNNNNNNNNNNNNNNNNNNNNNNNNNNNNNNNNNNNNNNNNNNNNNNNNNNNNNNNNNNNNNNNNNNNNNNNNNNNNNNNNNNNNNNNNNNNNNNNNNNNNNNNNNNNNNNNNNNNNNNNNNNNNNNNNNNNNNNNNNNNNNNNNNNNNNNNNNNNNNNNNNNNNNNNNNNNNNNNNNNNNNNNNNNNNNNNNNNNNNNNNNNNNNNNNNNNNNNNNNNNNNNNNNNNNNNNNNNNNNNNNNNNNNNNNNNNNNNNNNNNNNNNNNNNNNNNNNNNNNNNNNNNNNNNNNNNNNNNNNNNNNNNNNNNNNNNNNNNNNNNNNNNNNNNNNNNNNNNNNNNNNNNNNNNNNNNNNNNNNNNNNNNNNNNNNNNNNNNNNNNNNNNNNNNNNNNNNNNNNNNNNNNNNNNNNNNNNNNTCGAAATTCATTTAACCTTATTCTGGAAGATGAATATTTTATATCAAGTCTTCAGTAGAACACTATAACCCTGTACAGTAACATATCCAAAGTTTGGTGCCAAAATATGCAGGATTAAAATATGAAAAATGACTTAAGTAGAAACTTGAAATTGATATTACTCCAGAAATAGGGTGACTTACAAGAAACTGTTTGGTGAGAGGTTCAGTTAACCTATAAGGAGTGTGTGTGCCAACATTCAGAATTTTTGCATGTGTAGATCTTTTTATAAAAACATTTTTCAGTTTCTCTTCTCTGAATTTTAGAAAATTAAACAAGATGATTTCAGAAGTTCATGTTATGCCAAAGGTTAGATCTGCTCACAGAGAATCTAACAAAATTTGTTTCACAATTTTCCGACTTATATTTTAATTTCTATCAAATTTACAAAGTCACTAGATTTCTGGAAAAAGAAAAAGAAAAGACTGGGCGACTGTAGCAATGTCAGCGGTTCACCGGGAACGCGACAATGACAAGGTCGCCGTTTCGGCTAAACGGCGGCGAACCGAGGGCAATTGGGGCCATCACAGCGACCAGGGCATCAAGGCGGCTCTAGTACACGGTTGAGGGCACGCTCGCGCACAACGGGGCAGCGTTGGTGGCGCGGGTAGGGNNNNNNNNNNNNNNNNNNNNNNNNNNNNNNNNNNNNNNNNNNNNNNNNNNNNNNNNNNNNNNNNNNNNNNNNNNNNNNNNNNNNNNNNNNNNNNNNNNNNNNNNNNNNNNNNNNNNNNNNNNNNNNNNNNNNNNNNNNNNNNNNNNNNNNNNNNNNNNNNNNNNNNNNNNNNNNNNNNNNNNNNNNNNNNNNNNNNNNNNNNNNNNNNNNNNNNNNNNNNNNNNNNNNNNNNNNNNNNNNNNNNNNNNNNNNNNNNNNNNNNNNNNNNNNNNNNNNNNNNNNNNNNNNNNNNNNNNNNNNNNNNNNNNNNNNNNNNNNNNNNNNNNNNNNNNNNNNNNNNNNNNNNNNNNNNNNNNNNNNNNNNNNNNNNNNNNNNNNNNNNNNNNNNNNNNNNNNNNNNNNNNNNNNNNNNNNNNNNNNNNNNNNNNNNNNNNNNNNNNNNNNNNNNNNNNNNNNNNNNNNNNNNNNNNNNNNNNNNNNNNNNNNNNNNNNNNNNNNNNNNNNNNNNNNNNNNNNNNNNNNNNNNNNNNNNNNNNNNNNNNNNNNNNNNNNNNNNNNNNNNNNNNNNNNNNNNNNNNNNNNNNNNNNNNNNNNNNNNNNNNNNNNNNNNNNNNNNNNNNNNNNNNNNNNNNNNNNNNNNNNNNNNNNNNNNNNNNNNNNNNNNNNNNNNNNNNNNNNNNNNNNNNNNNNNNNNNNNNNNNNNNNNNNNNNNNNNNNNNNNNNNNNNNNNNNNNNNNNNNNNNNNNNNNNNNNNNNNNNNNNNNNNNNNNNNNNNNNNNNNNNNNNNNNNNNNNNNNNNNNNNNNNNNNNNNNNNNNNNNNNNNNNNNNNNNNNNNNNNNNNNNNNNNNNNNNNNNNNNNNNNNNNNNNNNNNNNNNNNNNNNNNNNNNNNNNNNNNNNNNNNNNNNNNNNNNNNNNNNNNNNNNNNNNNNNNNNNNNNNNNNNNNNNNNNNNNNNNNNNNNNNNNNNNNNNNNNNNNNNNNNNNNNNNNNNNNNNNNNNNNNNNNNNNNNNNNNNNNNNNNNNNNNNNNNNNNNNNNNNNNNNNNNNNNNNNNNNNNNNNNNNNNNNNNNNNNNNNNNNNNNNNNNNNNNNNNNNNNNNNNNNNNNNNNNNNNNNNNNNNNNNNNNNNNNNNNNNNNNNNNNNNNNNNNNNNNNNNNNNNNNNNNNNNNNNNNNNNNNNNNNNNNNNNNNNNNNNNNNNNNNNNNNNNNNNNNNNNNNNNNNNNNNNNNNNNNNNNNNNNNNNNNNNNNNNNNNNNNNNNNNNNNNNNNNNNNNNNNNNNNNNNNNNNNNNNNNNNNNNNNNNNNNNNNNNNNNNNNNNNNNNNNNNNNNNNNNNNNNNNNNNNNNNNNNNNNNNNNNNNNNNNNNNNNNNNNNNNNNNNNNNNNNNNNNNNNNNNNNNNNNNNNNNNNNNNNNNNNNNNNNNNNNNNNNNNNNNNNNNNNNNNNNNNNNNNNNNNNNNNNNNNNNNNNNNNNNNNNNNNNNNNNNNNNNNNNNNNNNNNNNNNNNNNNNNNNNNNNNNNNNNNNNNNNNNNNNNNNNNNNNNNNNNNNNNNNNNNNNNNNNNNNNNNNNNNNNNNNNNNNNNNNNNNNNNNNNNNNNNNNNNNNNNNNNNNNNNNNNNNNNNNNNNNNNNNNNNNNNNNNNNNNNNNNNNNNNNNNNNNNNNNNNNNNNNNNNNNNNNNNNNNNNNNNNNNNNNNNNNNNNNNNNNNNNNNNNNNNNNNNNNNNNNNNNNNNNNNNNNNNNNNNNNNNNNNNNNNNNNNNNNNNNNNNNNNNNNNNNNNNNNNNNNNNNNNNNNNNNNNNNNNNNNNNNNNNNNNNNNNNNNNNNNNNNNNNNNNNNNNNNNNNNNNNNNNNNNNNNNNNNNNNNNNNNNNNNNNNNNNNNNNNNNNNNNNNNNNNNNNNNNNNNNNNNNNNNNNNNNNNNNNNNNNNNNNNNNNNNNNNNNNNNNNNNNNNNNNNNNNNNNNNNNNNNNNNNNNNNNNNNNNNNNNNCGCTATTCGGCGGCAATGGCGGCTAGGGTTGCGTGCGTACGCGCGAAGGAATGGGAAGAAGCGAGAAGGGAAGTCGATGGGGGAGTGGTTGCGCGTCTTGAAGCCGTCGTCGTCCTGCCGGCGCTGGCACTGAGGCGACGGAGCGGCGAGCACAGCGGCCGGCATGGGCGAGCGGAGGTGGGAGACGATGACAGGCGGGTCCCGCTCGATTTTCTGATTTTTCAAAAGAATTTCTCCACGTTTTCCTTTGACTCAATTTGTATCTGGTCTTCCCGTGCTCCAAAAATTCTGAAAAATTTATCTCTAAACGACTTCACTTGGGGGAACAGTTTTCTCATTGCAAGTTTTCAAATTTCCGTGTGGCTTTTGAATCAAAAATTCTGTCAATTTAGAAATGGCATAATTGAACTTTAAAATATTTGAGCACCTTTTTACTCATCAGAACATCTGGACCCAAAATTCTATCTTGGCACATGCAGACCAAAGAAAGTTTTTCCAATGGACCCACTGTTAAGAATTTATTATATAATTTTTTGTTTTTATTTTGTCAAATTAATTATGACCTTGGTTTGACTTAATTAAAGTTTTGTTCATTTATTCCTTTTGATTATTTTGTCTAAACCCATACCAATCGCATTAATTAGTTTGATTAACAGTGAATTTAAACTGGGGTGTTACAGGAATGGAGCAATCTCTAATAGCAAGGTGAACAACATTAGTGGAGAAGGCCCTACTAGCTTTCACCACATAAGGAATATTCACCGAAAGCCAAGACTGGCACTGCTCAGTCGAAGCACCCTTGGGAATCGGATCAGGAACCGTACCCACTGAGATAAGAGCATCCTGGATATAGCCGCAAAAAGACGACAGGTGAAGCCCCTCAGTCTCAGCGAAGGAAGCCGCGTCCTTCTCCGCAGCCTCAACCTTCCCGCGAAGATCTTTGTTAACCTCCAAGACCTTCGAAAGGGACGAGTCAAGATCCTTAAGCTCTCTCTCCAAAGAAGCCACCTTCGCCTTTAAATCCTCAGTGGACATAGCAAGGGTCTCCTTCTCAGTGGAAAGATTGCTTACACGCTTCTCGCTGAGAGCAAGAGCTTCCTCCTTCTCTTTGACCTTCGTCTCAAGGACCGAAAGGTCCTTGCGAAGCCGGTCCCGGTCAGCCTTCGCCAGAGTCGGCTCATGGAACTGACGAAGAAAGGCATGGGTAATAGCGAGAGTCTAACAAAAATGAAGACAAAATAAGAAAAACAAAACAATCGAGTAAAAACAAAGAATGACAAATCATTTACCTTCATCTGCGAAGACAATAAAGACTTGTACAGGTATCTCCACCAGAAGTACAAGATCACTCTCCACTTGGGGAAAGCGAACTCTTTCGGCAGCAGTAACAAAAGGTGCAGCGGCCTGAGCACTCATGACGAAGGTGTTCGAAGGCACCTCGTCACAAAGAGCTAAAGAACTCCCAATGGCTTCGACTGAATGAAAAGTGATGTTAAAAGGGAAAAACTCTCCTGTCGTCGCTTCATAGACTGAGTAAAAGAAAAGTTCACCACCTGCTGAAAGGTTAAGGGGCTCACGAGGCCCGACACCTGGCTCCGAAGGCAGCTGAAGCTGAGAATGATCTGTAAGAGGTCTTAAATCATCAGCAATTCGACGAAGATCATCTTTACTCATAGATGAAAAACGACCTTCAGCAGGGGCTTCGGCCGCCGCTTCACCCAGAGCTGGGGCAACAGGAGGGGCACCAGAGCCTTCATTGTTGGGATTCTCCTAGATCTTAGCCTTCGATCCGGACCTAGTGAGAACAGAATCAGCATGCTTCGAAGCCTTGTCCTGAATCTTGGCCTTCTCAGCAACACCAACATCATCAACATCAACTGTAGAAGCAGGAACATCCTCCGCAGAGGAAGAAGAACTCTTCGAGGCAACTCTACCGGGGGTAGACTGAACCTTCACCCCAGCAGAAGTGTTATCCTCATCCTCCGAAATAGAGAACAAAGACAATGCGGAACGAATCTTTGGAGGCTTAAAATCAAAAGTTGGTTTGGCCGCACATAAAGTCTTCGGTTGATTTGCCGAAGCACCGACAGCAATGGAAGCTGCAGCATTAGCTTCGGCGAAAGAACTCCCAATGGCTTCGACTGAATGAAAAGTGATGTTAAAAGGGAAAAACTCTCCTGTCGTCGCTTCATAGACTGAGTAAAAGAAAAGTTCACCACCTGCTGAAAGGTTAAGGGGCTCACGAGGCCCGACACCTGGCTCCGAAGGCAGCTGAAGCTGAGAATGATCTGTAAGAGGTCTTAAATCATCAGCAATTCGACGAAGATCATCTTTACTCATAGATGAAAAACGACCTTCAGCAGGGGCTTCGGCCGCCGCTTCACCCAGAGCTGGGGCAACAGGAGGGGCACCAGAGCCTTCATTGTTGGGATTCTCCTAGATCTTAGCCTTCGATCCGGACCTAGTGAGAACAGAATCAGCATGCTTCGAAGCCTTGTCCTGAATCTTGGCCTTCTCAGCAACACCAACATCATCAACATCAACTGTAGAAGCAGGAACATCCTCCGCAGAGGAAGAAGAACTCTTCGAGGCAACTCTACCGGGGGTAGACTGAACCTTCACCCCAGCAGAAGTGTTATCCTCATCCTCCGAAATAGAGAACAAAGACAATGCGGAACGAATCTTTGGAGGCTTAAAATCAAAAGTTGGTTTGGCCGCACATAAAGTCTTCGGTTGATTTGCCGAAGCACCGACAGCAATGGAAGCTGCAGCATTAGCTTCGGCGATGGCCTTATCAGAACCATCCTTCGAAGAAGACTTATGAGTTTTCAATTTCTTCTTGGCATGATCTTTGTCAGACCTTTGATTACCAGACATCTTGAAACAATAACGAAAGAGTTTTCTTTCACGAAAGAGATTGGAACGACCAAGGGAGTGAGAATGCGCAAGACAGACAGTTGAAGAAACAACGCACCCGCAAGGATAATATATAGAGAAGAGGAACACATTGGGAACAGAAAAGAAGTACGGGACAAAAATGACAAGACACGTGTGTGGATAAGAAAGACGCACGGCGTAATCTTAATAAATAAATAAATTGTCTCCGGTTTCAGTTACACTAAAAAGCATCGTCAATTGAGGGCTAATGTTGGAGACCACACCGAAGCCACTTCCTAACCCACACGAAGACCAACTATCACCTGCGGAGAAGGAATGGAAGTGCAGTCAACACAGGCCAACGTTCGGCTCAAGACACTAAGCTAAGAGCCGTGCCGAGTTAGCTAATGGCACAGTTGTAAATATGATCTTTCTCCCCCCTATAAATATAGAGGGAGATGGTCATGTACGATCATCTGTTTTTCGGGCCACGAGCCAAAGAACAAGCATTCGAACCGCACTTAATTCTCTCTAAACACTCAACCCCTTTGTCCGGCACCATGGGCAAAGGAGGGCTTCGCGTAACCAAGAACTGCAGGGAACCTGAACGTGGACACCTTCGTACGGGACTTCGGGACTAGGAGGAGGGCTTCGCACACATCGGTCAACGACCCGGACTACATCAACGACCTTCGATCGCCTTCAAACGAGCAAGGACTACATCAACTCTTCGTTGGAGGCCTTCGACGTCACATCAAGACCTTCGATCCCCCAACATTACTCATAAGACATGCAGCCCTGTGAGTTGAGCATATAAATTGTTGATACATACTCACTACTTAATCACTAGATAAGTTAAGACTCGAACATCAGGTATTATCGATGGTGTCAAGTTAGGTTGCTATGTTATATCATCACACATGGATTTAGAAGAATTCTTTGGTGTCCACGGCTTCTTTTTTGTGCAATTCTAAGGTATTGAATAGACTCTTTCTCCTCCATGCCATAAAACAACAATCATGGCACATTACATGAGTCAAATATGAGAGAGTTATGGTATGACACATTGTGCTTACTCTTCACCCTCTGTATAACATTGTGTGCCATCCTATTATATCGAGAATGACAATCTACACAACTAATTAGTGGGATGACACAATAACTCTACACTTTTGATTTGGGTTGATATTATGAAATGTTCTTTTGGAGTCATGAATAAACATTGCCCAGTAGAACAGGTACCTTAACTTTTTGTGTGTGTGAAACAGAACAGGTAGCATAACCAGTGGCGGAGGACGGAACAAATTCAAGGTATGGCAAAATCAGAGTAGTTGGCCCATTAAATAAATAAATAAAAAAGCTTATGAGGTGGCCTAGCTCTCACAAAAACGGTTTGACTTCATCGATTAAGCCTGTCGCAGAAACTTTTTTCACCTCTTTGCATTCTCATGACGGATAATACTATAATAGACAGTGTGATCAAAGATTTGAGAGTTCATGCAGCTAAGTCAAGGTATGGCGGGCGGCCNNNNNNNNNNNNNNNNNNNNNNNNNNNNNNNNNNNNNNNNNNNNNNNNNNNNNNNNNNNNNNNNNNNNNNNNNNNNNNNNNNNNNNNNNNNNNNNNNNNNNNNNNNNNNNNNNATCCCCCCCCCTTTCTTTGGCCATACCGGGTCCTCCGCCCCTGAGCATAACTAATTTTAATAATTTTCAAAATGAGAATATAAAGTGTAAATATCAGTTAATATCACTGCCGAGGTTTGAAATTCTCATAGCATGAACGTAGTAGTATATCTTTGAAATGCTTGCGTGCGATTCTCTACGGCCTCATAGTATTCCATCACATACAGCCCGTTCCCCCGTACACGTACACACACGTGGTGACGCGGAGACGCATTGCTCCAGCGAAAATATCAATCAAACCTTCAAGTCGAAAGCAAAATGCATCTACAAGCGAAAGCTGATGGATTCGGCGGGCGGTAAGCGTACCCCAGGTCCCCAGCGCTTCTCATTCCCGTCGTCTGCATCGTAGATGGGCAGTGGGGCCCGCTCTCCGAATGACGCATCGCTTCGGATGCTCTCGCCGGACAGCGACCGCGCCCGGCAACCGTCGGCAAGAGCCGCAACGCGGAAATGGCGCGCATCGCACTCGTCGCGCCACGCGGGGTAAAATTCGTTAGATCACCATCATCTCGACTCGATCGCGTCGGTTCCTTGGATATGGCCCGCAGATGCAGACGAAATCAGATTTATCACAGAGAATCGGTAGGGGGCAACGATGATTATTGCATCCTTAGTTTGGTTTCAAAAAGTCCTAAGAGGAGTGTTAGACTAACTTTGGATCCTTTAGCCAATCTGTTTGTATCCTTAACTAAGGTCTCTGACTTCTGTACTTTTGTTGCTTTTTTATATGTTTAGTCGATTTTAGTAGTTTTGAAGTGATTAGAGGCTAAAAGTTTAAGAGAGAGTGGACTAAAAGTTTAGAAGATTTTAAGATGTCATGTTTAGATACAAATTGACTAAAATTAGCTAGCTAAAAGTTTAGGAGGTGGATCCAAACAGTGCCTTACAAACCAGCAGAAATGAGAGAGGAATTAAGGAAGAATTAATTAGCGGCCAAGGGAGAAGAGCAAGGTTTTAAAAAACTGTTCCGGACCGGATCGGTTTCACTGTTTTTTGAATGGTTTCAATTTTGATCAAAACCGGTCAAAATTCATCAAATTCTTAAATTCTTTTGAATTCTGGATTTTATTTTTTAGAGGGGAGTTGAATTCTGGATTTGAAAACCGGTCAACCGAAGGAACAGACCGGTCCTGGTGCACATGGTAGAACTTTTTATTGATCTTTTTTTTTTGAGAGGGGAAAGTTTTTATTGATCAACTCAACGGGTTGGTCATGGGCTCAAAGTTAGATTCGGCCTTGTTGTGTTGGGCCAGAGTATGTTAATCATCAACGTTCCCATTCAACCGCAGGAACATTCTTGGCACCCAAATAAAAATCGAAAAACCAAAAAACCAGCCGCGCCAATTGATCATCCCCATCAAAACCATCATCTAGCACCATAGTAATTATAGCACGCATAAACCCAAAGAAAAATAAATAAAAGAATCTCCTTTCTAAACAAAAATCACAAGCATAGACAGCAGCGAGGGACGCAGCACGCGTCGCTGCACTTGGGGCAGGCGCAGGAGCACCTGGGGCACGAGCAGGTGCAGTCGCACGAGCAGCGCGGGCACGGCGCGCAGCAGGCGCCGCAGCAGCAGCTCTCGTACACCACGCCGCACCGGCAGCAGCAGCCCCTCNNNNNNNNNNNNNNNNNNNNNNNNNNNNNNNNNNNNNNNNNNNNNNNNNNNNNNNNNNNNNNNNNNNNNNNNNNNNNNNNNNNNNNNNNNNNNNNNNNNNNNNNNNNNNNNNNNNNNNNNNNNNNNNNNNNNNNNNNNNNNNNNNNNNNNNNNNNNNNNNNNNNNNNNNNNNNNNNNNNNNNNNNNNNNNNNNNNNNNNNNNNNNNNNNNNNNNNNNNNNNNNNNNNNNNNNNNNNNNNNNNNNNNNNNNNNNNNNNNNNNNNNNNNNNNNNNNNNNNNNNNNNNNNNNNNNNNNNNNNNNNNNNNNNNNNNNNNNNNNNNNNNNNNNNNNNNNNNNNNNNNNNNNNNNNNNNNNNNNNNNNNNNNNNNNNNNNNNNNNNNNNNNNNNNNNNNNNNNNNNNNNNNNNNNNNNNNNNNNNNNNNNNNNNNNNNNNNNNNNNNNNNNNNNNNNNNNNNNNNNNNNNNNNNNNNNNNNNNNNNNNNNNNNNNNNNNNNNNNNNNNNNNNNNNNNNNNNNNNNNNNNNNNNNNNNNNNNNNNNNNNNNNNNNNNNNNNNNNNNNNNNNNNNNNNNNNNNNNNNNNNNNNNNNNNNNNNNNNNNNNNNNNNNNNNNNNNNNNNNNNNNNNNNNNNNNNNNNNNNNNNNNNNNNNNNNNNNNNNNNNNNNNNNNNNNNNNNNNNNNNNNNNNNNNNNNNNNNNNNNNNNNNNNNNNNNNNNNNNNNNNNNNNNNNNNNNNNNNNNNNNNNNNNNNNNNNNNNNNNNNNNNNNNNNNNNNNNNNNNNNNCCACAGCCGCTTCAGCTGCCCGGCGCAGGAGCAGCTCGGCGCCGGCGGCCTCTTGAGATGGAACGGCGAGCAGACGCCGCAGCAGCAGATCATCCACGACAGGTAGCTCAGGCAGCTCTTCGCTCTGCAGAAACAGTGTATTCAGTAATCAGCTCGTCGGTGACAAGAATAACAGAGATAGCTGATGATTGCTAACAACAAGTTCAGGAATTATTAGGCCAATTGGTTGTACTAGCAATAATAGTTGATGCTTCTGAAATGTATCGTGTAAGTGAATTATTAGTGCAGGCAACGACAAGTTCAGTAATCATTGACGGGCGTAACATCGAGTTGATAGTTCTGTCAGATGTGGCGATGGCAAGAAAGAATAAAGTTAGTTATGAAAAACATCATTGTGGTACTGGAGTCAATATATAAACATATTGTTTACTACGCGCGACCAAGAACTAAGAACATCAAGAGAAGAGAATACATCAAGAACGTACCGGAACTTCTTCGAATGGAGAGATAGATCATTACTTCGTTTCTCTAGCCCACTGCGCTTAAAGAGAGATAATGAGAGAATGTACCATTTATGTGAAGTTATAGACAATTCTCAACCTGAAAATCCATACATCGTTATGAATGGATCGGGATTTCTAACCACAAAGTCATCAACCCTGCAAAAATTAATCCAAGACGCAGTCAACATCTTCATCATTCTCCATGGTTTTATTTCTAACAACAACAATTTATTTATCTGTGCTATCGGTGCACTAGAAATGAAATCACTTATCTCTCGACAAGTTGAGATAATCTAGTGAATGAAGATCGTTTTTTCACAGGTACAACAAAAGTAAGATCGTTTTTTCATAGGTACAACAAAAGTAGCTTGCATTTGTTGAAACTAGCTTCTCCTTATCAAATTTTCGATAGTTCCAGCAAAATAAGTATATGTGGTCATTGCCGAAATTCAAAAATAAGTAACTGCGTCTTACTCTTTGCAACATCTGGAGGCAGCGTGGACCCCTTCAATGGAACTTATTTCACCCTGCAAATGAATATTCTCTTGAATTAAAATTAGCTTGCTTGAGGGAAATAGTATTTTATTCATAGCAGTAACACTATACAGTAATTAAAAGCCATGTGTTCAGCTGCCAACTTTTCAGGTGCTGTCTGGGATATTTTAGCACACAGCACATTCAAAAGTTGAGTACTGAAGAATATCAGAGGGCTTTCCAATATGTTAGCGAGATGCTGGAATCTTACGGCAATATTTACTCAGATCACTGTCATTATTCAGACAACATATATAACCATGAAACAAATGATAGGATGCGTAAGGAAGCTCATTTGCGATGAAGTAAGCTCATATATACTGCTATTTAAAACAATAACAACCTGAACTTAGTTTTGTCACTCCAAGATCAATACTCCATCCGTCCCTAAATATAGTGCCTTCTGTGTTTGGTCAAAGTCAATCTTTACCAATTTTGACCAACAATATCAATAAAAATAAAATATTATAAATAAAAAGAGTTATATACTATGATAGATGTTTTTATAGTGTATCTAAGAATGTAAATTAGACTTTGTAAAAGTTTTTCAATATTTCGTTATTCATAGTCAAAGTTCACAAAGTTTGACTTTAACCAAATCTAAAACGAACTACATTTAGGGACGGAGGGAGTGTATATATACACACAGCTTGGTTGGTAATAATTTGAGCTGTGGTAATATCAGCATTAGAGACTTGCACTGAAAAGAATTTACCAAACACATATACTGCCAAACTGATGGCACATAGACATGGAAAGGCCCTGCATGCAAGGCTGTATTTTTGCAGGCTCATCTGGCAGTAATCAATATAGTGTATTAACCAATATAATTTGCAGGAATATTTTATTGCTCCCAATGAATAGGTTTATAGGTTTGGACGTCCATGAGTAGTTAGAAATCTGGAGCAGTGCGGCACTCTCAATGCTATCATAAGCAACAGAAGGCGGCGAGTGAATATCAAGAATAAGTATCATGTTTCAAAAAAGAAATAATATAGAGAATATACAAGTCTGAATGACAATACATACTGACCCACTGGACGAGGCCTAACACTGGGAAATTTAGCTCTACCAGCGGTCAAAGTCAAGTTATGACACATACGTGACTAGTCAAATCGGGCTTAACCAATCGTGCATGTTGCCATATGATATTTAAGAAGTTTAGATGGTAGATCCTACACCCAATAGACCTTACTAGAACAAAAGCTAGCACTACATAACGGTGCATAGGGGACGAGCTGCTTTGCGATGAAGCCTGCAGGCAAGATGGAGCTAGCAAGTTCGAAATGGTAGGCAGAAGGAATAAAAAGGGCAGGGGTTAGCCTTGACACGCGCGCAAATCCATGGTACGGTCCGCACGTGCCCAGAGGAAAGACCAGAGATTAGCAGGAATAAACTAATCTTGCTCCGCGCTTAACGGTCTTATCACTCTTAGGTAAGCATATCTCAATGATTCTTGCTCTCAATTCTTCTCAGAAAAATAATAATTGGAACTTGTTGCTCCGGAAACAAAATAAACACTTCTTGTTCACTGTGTAATCAATTTCGTTGAGGCAGAAGCATGTTATGTACATATTTGGCACCTTATTTTTTTCTCCCAAGATACTGCAGCGGAGGAGCAAGCAGGAGAAGAACAGCGTAATGATGGGGGAGGATCGTCGGTGAAAATGGTTAGTGTAACCAAATCAGGATTCAGAGATGCATGTGGGAGGGTTATGATGTTATGCAAGAACAGTGTATAACTGATTGATCTCAAGGGGGAAAAAAAAAAGCGTACCTCGAGGAAGCCGATCTCCCGGTGGAGCGCGTCGACGGCGAGCTGCAGGCGGTGGCGGCCGCAGGGGTCGGGCGAGGCCGGCGGCGACTTGGGCCTCGGCGCCGCCGCCGCCATTGCCGCCTTCTTCGCCGCCGCAACTACCAAGATGACCGGACTATTTGAAGGAGCAGCTGGAGCTTGACGGGCAGAGCACGCAGCAGCAGCAGCAGCAGGCGAGTGATGAAGTGGAAAGAGAATGATGACGGAGCCTGCCTGCAGCTAGTGCGAGAGGTGGGCGGATTAAATGGTGGCGAAGGGGTCGCACATGGAGGAGGAGGAGGAAGGGATGCTGGGGGGTTGCCGTTTCGGGGGGTAATGATGGAGGGGAGAGACGGCGCCAGGCCCTCGTTTCCGGAATGGAGTACAGTACGAGCCTGCGTGGCTGGCGCTTTGTGCTCGACTAGCCAGGCCACCGCCGCGGCGTCGAGACGTTCGTTATTGCGAGTGCGGAATGCTGATCAGTGAGAGCGAGCGAGCGAGGGTGGCAGGGCAGAGGAGAGAGAGGATGGGGCTTGAAAGATTGTCGGGGCCGGATCTAGAGCGACGACGGATGGACGAAGGGATCCAGTCAGTGGCCGCGTGGGCACTGTTGATCGCCGCATGCTGCTCGCGCGCTGCTGTCGTCTGTCACGAGCTGGTGCTGGTGGGGCAGTGTGCAAACATGTGCCGCGGATGCTGGTATACTAGTCTATACAACCAGAATCGTCAAATACCTCTATATCAAGGTTTAATCAGATTTGGATCACCAACGCCCCAGCCGAGAAGCTGACATTGTCAACGAGCTAGCAGCGCTTCAACCGGCCGGCCACGCACGCTAGCTAACGGTCACAGTTCGAGGCTCGATGGTACTAGTAGATGCTACAACACCCGGTACTAGTAGATTCTACTACACCCTGAGTTCATTTTTAATTGGTCATTCAGAAGTTGCGCTGTTTAATTTCCAAAACTTCATCAGTAATAAGAGGCAAAGAGAATTAGTAAGCTTTTTTTTTCTCATGAAATTCCTTTGAACCAACCAAAGAAGCCCTGGTTGTTGGGACAAGGCAGCAGGGTGCATGGAAAGGTGAGATGGCTGCAATGCACGTCAGTACGGACAAACAAATTTGTGTTGTGCTACCGTACTTCGCTCTCCTTGTGCCTGCGTAGTGCGTGCGAAGAATATGTGTCGGTTGCCAGGCATCATCACTCATTCAAGTACGGAGGAGAGTACCGTTCTCTCGATCACCCCCGGCACCGGAGTACTGATCTGAACAGGTCCGGATGGGCAAGACCTGTACGTACGTCCGTGATGAATAATCGTTCCACATTGTGTCACGTGATCGTTAACTCGTTCATCCTGGACGATCGTAACAAGAAAAGGTCGTAAAGAAACAGTGGGCCTACCACAGTCGATCGAGACTGAGCGTGCTCTCTCTCTCTCGGTCTCCCCTTTTTGCATCGAAACAGTGATACGAGCTGCTGAGGCCTATGGCCTAGGCAGAGCTTAACGCACATGAGCAAACAGTCGTATACGTACTCTTGCTAGTGTACTACACGTACGCATGCGTCTGCTACTGCACCGCTCATACCGGCAGCCATACCCGCCCGGCCGCGCGCACCTGCATCTGACTGCATACTCGGCTCGGCTGATCTTTCTGACAGGTCGCGACGTGGACGCACCTCCTGCCGCTCGCTGGTGATCTATGCTCGGAAACATGTCGATGTCGAGCTCATCTCTTAGCGCAATGGCACGGAGGCTAGCGGTGCCATTTCTCCCTCGGAATTTAGCCGGCACTTTGTGCTTTGATGCAGCTATGGTCAAATCTGCCTGCTTGCTATCTGCTCTGCTCCAAGTCGAGTCGACGGCGCATCCCTCTAGTCCATTTCCTCGTTGCATCCACATTAGTCACGTCGCCATCACCAAGTTCGTCTATTCATGCGTATTTACCATCTAAAATTTGACTACGACTAAGCTCAGAAATTTTTACGTTTCTGGGCGGAGAGGGTACGAAACTACGAACCCTGTATTTGTCGCGACTCTCGGATGTGATTTTAAGTTCTTTTGTCTAGGTGGTTTGATTTAAGTGCAATGCGTTCTAGCGGAGCATACGAAGACAAAAGAAAGTGCAATGTTAAACCTGTTGGACAACTAGCTGTGAAATGGATATGCTGCACAAGCATATATGTGAATTAATCACAGACCAGTTACAGTACAAGCATACAAATCAAGTAAATGAATATCGAATATCCAGTCCAAGGTTCCTCTTTGTACATTATTGATTTCGACAATTGAATTCCTAAGATTTCTACTGCCTTTCGAATTCGGGCCCATATAATTTGGACTATATGGCTGCCGTCGCAGTCTAAAAAGATTCAAGTGGGTACCCTGCACGACTGCTCCCTTTTGTCCCACACTGTCCAAAAGGCTCCATGTATCCCTGCCCTACTGTGCCTAGTGCCTGCCCCTATCTAGCACCCACTGCACTACAACAACGAAAACGAGACAAGCGCGAGTGTGCAATCCCATCATGCATGGCCCTGCTGTGTACTTTGTCTTTGCCTTGGATCTCATCACCGTTGATCAAAAGATCAGTGACAAAATGAACAAAGAAGAGGCCATGCCCCCAATAACAACCCGAAGAAAAGAGAAACTATTACAGGGCTGCAATAAGTGCAACCCTGAACCCCTGAAAAAAAAAAACAAGAAGAGGCAAAGGCCATGTATCACCTACATTTACATCTCAATCTTTTACACACGTTCAACACTGGTAATTTAGTTACTATTACCCTATGCTTTCCCTTCTGCGGGCATGTGCCACTCCCAAAACTCCTCACCTTGTCCCGGAGCACGCCTCCTGACGTTCGTCCTCTTCGGCGACCTGGCTCTGTTACGCCGTCCATGACCACTGCTCTTGTTTTCTCTCTGATCACCATCTCTTCTCCTCGCCGTCCTCTCCTTCTTCGCGTCATCGTCACGCGCCGGCGCCAGCGCCGCCGCCTCGCCGGCCGGCACGTCTTCGTCGACGACGCCGAGGAGCGGCTCGAGGTCCTCGACGACCGCGGACATGTCCGGCCGGTTCTTCGGGCTGCCGCTCAGGCACCGGTACGCCACCATGGCCGCCTTCTGGGCCGCGGCGAAGGAGTACTTGCCCTCAAGTGCAGGGTCCATCACCCGGCTGAGCTTTTGCGGGTCCTTGAGCCACGGCCGCATGTGCTCCACCAGGTGCTGCTCCCTGCTGGGCCGCGTCTTGTCCACGGCTTTCCTCCCGGACAGGATCTCCAGGAGCACCACGCCAAAACTGTACACGTCGCTCTTCGCGGTGAGATGACCTGTTGTCCATATGGTTTTGAGGTTAGGACATGCAAATTTTCAGGAAACTGAAGAGCATTTGCTTCCATTTGTGCGAGAGAAGTATGTGCTCACCGGTGAGGATGTACTCAGGGGCGGCGTAGCCATGGGTGCCCATGACACGAGTGGATACGTGAGTGTCGTCGCCCTCCGGGCCATCTTTGGCCAGTCCGAAGTCCGAAAGCTTCGCCTTGTAGTCCTGGAGATCATAACAACAACAAAACGTTAGCCATCTTTTCTCTCGAAACCAAAAAGGCTATAGCAATCCTACTTGTAAAGCTTTCTTTACAATGTTTGTTCCCTTTATTTTCCTTTTTTAGGAGAAGTTTGTTCCCTTTGTTACACCACACTTCTTAAATTGGGCCGGTTTTGGATCAGTTCAGCCCAATGCCTCCCAAATATATTTCAGCTCATTGTGGCCCAATTTTGTTTGATTCCAAGTCCACTTAATTTGGAACTGTGACATCGGAAAACTCTCCATAGGGTGCTACGAATAAAGGTGTAGGAACACAATCTAGTCATTTTGACATTTCTGTCGAAATCAAGACTTTCAAAATAATAGATGGTGGTATAATCTCTATTCCTCCTACTTTTCATCCATTTCTTTTTCTACTCTGGTTCCTTCTGAAAAAAACAAGTATGAAAAGTAGTGATGCAAATTTGCTCTCCTAGCCAGAAAGTCCTCTACTGTTATTTTCATAGCCACAAGTTGCTCTACCGGAGCAGCCCGCAGTCGAAAAGTAGCAAAACCACGCTACCAAGCGATTACGACCATCTATGTGTTGACCGATCAGTTTATTCATACGTGTGAAGTCAACTGGAGTGGCTAGGAGGAGCACTCAGTACATAATGTCTTTTTTCTTTTTTCAGAAGAAATTGGTGTTATCAATTTGTAAAGAAAGCAGATCAGGAAACTGATTGATTCTTACCGGATCGAGCAGGATGTTGGATGTCTTGAAGTCGCGGTAGATGACGGGCTTCTCCGCGTCGTGAAGGAAGGCGAGGCCCTTGGCGGCGCCCACCGCGATGTTCAGCCGCGTTGACCATGACAAAACGGGCGGGAACTCTGCATGGATCATGCGCAAACCCATCACAAATTAATTACTCCTCCATCTCAGATGGCATCCGGCATGGGTGGAATGCCGAAACTAATATAATCGATCAGCTCCAATAAGCAATGAACATTTCTCTCCAGCCAACAAATCATTGCACCTAAAAAAAGAGGATTACGGTACTATATGTGTGGAGATCAATGTCAACGTGCTATACGCATGCAGTCATCTTGTTGTGAGGTGTATGTGTGAATATTTAATTAGAACGGGCCAATCAGCGAGATCGACAGCACCTGTCTAATTAACATATAGCACATCTATTAATCTATATGTGTTCTAACAAATATCAGCACTATTGATGTAGAAAACTATAGCTAGCTGGGACATCGATGTTGATAGATCTATTGCTTGTCAATTGTGATTTCTTGACATCCTATATATAATAAAGTCCTGCAGCTTATATGCATGTTAGAAGCCAAGCGTACGTGCACTCACATACATAAATATGCAGGTGCACATAATTTATATTTTGCTCAATTGTTCGTAAGACAATACGGAGACACTTTATCCACGCACAATTTCAGCGAAGGTAGGTATAGTGGCACACAGTTTGGTGTTGAGGTCAAAGTTTTGCTTTGCCAAAATTTGATTTGTATATGAATGACCTACTTCTAACAACTGCGGTTTTACTTTCAGTCCATTCTTTACCATCCGCACCCACAGGTCTGCATGCAGAAGGCTCGCGTGCGTCAGTTGTTTCTTCTACAGTGAACGTGAAGACCAATGAAGGGTGAGCCAGCAATAAAGTCAACAGGGATATGTTGGGCTGGTCAACCGCTGGCTTCTTCCCATCCCTGTCAGCTTCCAGACTTGGCGTGATTGTTTACTGATCGATGGTTCGTTGAGTCGAAGTTATTAGTGCGAGCTAGCTGGAGCGAGATCGTGTGCGCGATCTACCGTTGACCAGGTCAGAAACAATTAGCTATCCAGCTCGCCGTAGTGCAGTACAACTAAGGTCTGTCTAATCTAGTTAGGTGATGCATACATGGGTGATGGGTCCTCCAACTAGTTTGTCGGCCGGCCTGCCAACGATGATGCCATCCAGTCTGCTGACGCGGCACGTATGGACGCCGNNNNNNNNNNNNNNNNNNNNNNNNNNNNNNNNNNNNNNNNNNNNNNNNNNNNNNNNNNNNNNNNNNNNNNNNNNNNNNNNNNNNNNNNNNNNNNNNNNNNNNNNNNNNNNNNNNNNNNNNNNNNNNNNNNNNNNNNNNNNNNNNNNNNNNNNNNNNNNNNNNNNNNNNNNNNNNNNNNNNNNNNNNNNNNNNNNNNNNNNNNNNNNNNNNNNNNNNNNNNNNNNNNNNNNNNNNNNNNNNNNNNNNNNNNNNNNNNNNNNNNNNNNNNNNNNNNNNNNNNNNNNNNNNNNNNNNNNNNNNNNNNNNNNNNNNNNNNNNNNNNNNNNNNNNNNNNNNNNNNNNNNNNNNNNNNNNNNNNNNNNNNNNNNNNNNNNNNNNNNNNNNNNNNNNNNNNNNNNNNNNNNNNNNNNNNNNNNNNNNNNNNNNNNNNNNNNNNNNNNNNNNNNNNNNNNNNNNNNNNNNNNNNNNNNNNNNNNNNNNNNNNNNNNNNNNNNNNNNNNNNNNNNNNNNNNNNNNNNNNNNNNNNNNNNNNNNNNNNNNNNNNNNNNNNNNNNNNNNNNNNNNNNNNNNNNNNNNNNNNNNNNNNNNNNNNNNNNNNNNNNNNNNNNNNNNNNNNNNNNNNNNNNNNNNNNNNNNNNNNNNNNNNNNNNNNNNNNNNNNNNNNNNNNNNNNNNNNNNNNNNNNNNNNNNNNNNNNNNNNNNNNNNNNNNNNNNNNNNNNNNNNNNNNNNNNNNNNNNNNNNNNNNNNNNNNNNNNNNNNNNNNNNNNNNNNNNNNNNNNNNNNNNNNNNNNNNNNNNNNNNNNNNNNNNNNNNNNNNNNNNNNNNNNNNNNNNNNNNNNNNNNNNNNNNNNNNNNNNNNNNNNNNNNNNNNNNNNNNNNNNNNNNNNNNNNNNNNNNNNNNNNNNNNNNNNNNNNNNNNNNNNNNNNNNNNNNNNNNNNNNNNNNNNNNNNNNNNNNNNNNNNNNNNNNNNNNNNNNNNNNNNNNNNNNNNNNNNNNNNNNNNNNNNNNNNNNNNNNNNNNNNNNNNNNNNNNNNNNNNNNNNNNNNNNNNNNNNNNNNNNNNNNNNNNNNNNNNNNNNNNNNNNNNNNNNNNNNNNNNNNNNNNNNNNNNNNNNNNNNNNNNNNNNNNNNNNNNNNNNNNNNNNNNNNNNNNNNNNNNNNNNNNNNNNNNNNNNNNNNNNNNNNNNNNNNNNNNNNNNNNNNNNNNNNNNNNNNNNNNNNNNNNNNNNNNNNNNNNNNNNNNNNNNNNNNNNNNNNNNNNNNNNNNNNNNNNNNNNNNNNNNNNNNNNNNNNNNNNNNNNNNNNNNNNNNNNNNNNNNNNNNNNNNNNNNNNNNNNNNNNNNNNNNNNNNNNNNNNNNNNNNNNNNNNNNNNNNNNNNNNNNNNNNNNNNNNNNNNNNNNNNNNNNNNNNNNNNNNNNNNNNNNNNNNNNNNNNNNNNNNNNNNNNNNNNNNNNNNNNNNNNNNNNNNNNNNNNNNNNNNNNNNNNNNNNNNNNNNNNNNNNNNNNNNNNNNNNNNNNNNNNNNNNNNNNNNNNNNNNNNNNNNNNNNNNNNNNNNNNNNNNNNNNNNNNNNNNNNNNNNNNNNNNNNNNNNNNNNNNNNNNNNNNNNNNNNNNNNNNNNNNNNNNNNNNNNNNNNNNNNNNNNNNNNNNNNNNNNNNNNNNNNNNNNNNNNNNNNNNNGAAGCTCGGAGACGGTGAACACGTGCAGGTTGGACCCCACCAGCGACACCGACAGGTCCTGGTCCGACGCCGTGCTCATCACCTCCGTCAGCGACAGCCGCTGCTGCAGCCCCGTCGCCGTCGCCGGCGACCTCGCCACGGGCTTCCGGTGGTGAGGGCCCGGACGGACCTTGCCGCCGCCGCCGCCCAGGCAGCCGCCGAAGACGAGCCGCCACATCGAACGCTCCTGCTGCTGCTTCTTCTGCTTCGTCGTCATGGTCGCACCACCATGCAAATCTCAAAAAATCAATCTTTCTCAGCTGGATCGATGACAGATCACACCTGCGCTCTAGCTCAAGCTGCAATCGAGTCCGTTGACAAGTACAAGAAGGTATACTAACAACGAGCACTGAGTTTCTGATGATGCAACAAAGTGCTGATTGGATGGAGTTGAGAGAGGCGGAGGGAGTTTGCGAGGAGTATTTGTAGGGAGGTGTAGTGTGGAGAGACGACTTACCAGCCATGCCGTATATATGGACGCCTTCTTCTTCTTCGTAGTCTCTCCTTCACCGGTAACACGGGATCAGAGACGGACGGCCGCGTTGAGGCGCCTACGTTCCGTCGGATGACATGTCGCCACGCTGACCGTTGACCTGACTCGCCAACACATGCGTTCTTCAGTCCGTTTCCTCTTCCTTTTCGGCTTAGTACTAAATTAATTAAAGGATAGTCCCCTTATTTTGTGCTAGTTTTAGTCTCAAAGATTTAACAGAAACCTTGAAACTGTGTGCCCTGCTAGCGACTATGTCGTCGTTGTTGGTGGCCAAAATGTTGCGCTCCCTAACTGGACTACTCAGGTGGGGTATGCTGAGCAGAAGGCTGAAACAGATGACCATCACGTGGTGATGTTTTCATGTAGATGCATTCTGGTGTTTGTTAAGTATACGATGTTTGTTATTAAAGCATGCACTTTCTTGCCTATAGATACTACTTCCACGCCAGTCTTTCACGGATCCTGAACTATCTATAGACAAACTCAAATTGATGAATATATCAAGTTTGTGGAGTTGTATATTGACCTGATATTGAATTTTCCAGCCGCACGCACAGCACGATCCAATACATAGAGGTCTTGAGAGAATCAGATCACATCAAATCAAATCGAAATCATCCTAGGAGAAAAAACATTTCAGTTCTAGCATCTAGGCCCTCGAATTTCCTGTCCCAGAAGCAACACGCAGACGCATCCTAGGAGAAAAACATTTCAGCCTAGCATCTAGGCCCTCGAATTTCCTATGTCCCAGAAGCAACACGCAGACAAGACAGCAGAAATGGAAGTCCCCATCACCCGCATCTCAATCAGAGACAGCGAAAACTTCAGCTGTGCCATTGAGGTTGTTAACGAACAGATCCACGCCCTGATCCTAATTTCCCATTCATATTCCTCTCTGCACTGGCGAGAAACAACGAGATGCTCGTGGTGCCCGGATCGCCACACGCACGTTACCAATTTGGACAGGGGAAAATATACGGGTGGCGAAAGCGACAGGTTACCATTTCCGGCGCCCAACCTCGAAAAGGAGATGAATTGGGTCTCTAGAAATCGTTTGGGAACAAGCCAGGGCGTTGATTTTTGTGAGGGGTTAGCAGTACCGATCGATCTCGTGCAAGCATGCGTGAAGCTGTAGCCTTTTCCCCGTTTCTTGCCTGGTTTCAATTAATGGCGATGAAGGGGAGTAGGAAGCCAACCGGATTAAGTTGGAGTTAGGCTTGACATCTGGCAATCCAAAAACATCTCGATGACTCTTCTTATGATAGACACTGGACCTGAAACCCCCCTTTTTTCCAAGTTGTTATTTACGATTGTACTTTTAGTATACTTCGTACGTATGTGAACTTTTCAAACTTTGAGGTTCTGAAGAAACATAGTACCCTACTCACTAATTGTACCAGTGTTTACATTACCAGAGAGTTGGATGCTGCTGCACCGTTGACGTACAAGTCAATTGCTACTTACGGTACACTCAATGTTTACAAATGTTGTTGAGAAATTTCCAATTTTCAGCTTGAACATTTTAACTACTGACGTAACAGAGATTCTGGTGGGCCACGAGAGTTCGAGTCTTAGGACAGAGATTCTGTCCTAACCCTGCAGCCACTGCTGCTATGGGGGCCTGTAAACTCTGCTTTTCTTCCTAAATGAATGGGCAGTGCACTGCCGGTTTCCCTTCAAAAAAAAATTATTCTTTATAAGTAGTAGACAACGATGTCCATCAACAGTGTGGTTTTTTTTGTTGATTTGTCAGCACCCAATCTCTCAAAGTCCTTCATGGGGGCATGCTTGCGCGTGTATAGATTAGCCGTGTTTTGCTTTTTTCAAAATGCTCTGATCGTGTAACATGAAATTCGCTGGAGTTGATTATACGCTGTTTGGTAAAAGAACATAGTAAGTTCTAGGCATGCATTTGACTTAAGTGTTTTAAACTGTTTTCAAACTTTTCCTTGCCATGTAAAACAAACTGTGTTGAAATATTTCCACAATGTTACAGAGTGTCATTCACATATCAAAGCTCGTGCTAAGATTCATCACATTTCAGGAAAAATAAAATAAAATGTAGCCTGAAACGAGGAGCAGGTTTCTCCGATTTTCATGACATCCCCATTGTGGCACGAGACAAAGTCGGTCAAGTAAGTGCTCACATCGACCACGCGCGCGTTGGGACGCGATAAACAATTGCCATCGTTGCTGCAGTCAACAGCGAAGCGCCCTACTAGTTGCCAGCGCGTTTTCGGCCAGTTGATCTGCTACATCGCAATTGAAAAATTTTAGTTCCACGGACACCCTCGCCCGGGACCTCCACGTGACGAAAAAAAAACAAGTGAGACGTGGTGCGCAACAACCCCCGTGCAGCGAACGAGGAAACGCTCGTCCGTATGTTGTGTGGCTGTACGGTTCACGCGTCGGATCTCCCACGAGCGTGACGAAGCCAAAAATAATCTGATACATGGTCTGTTAAATTACGAGCACGTAAGTATGACGTGTGTTCGTGCGCGCCGTGACGCGCCAACGGACTCCGGCGATAGAATTGAAAACGGGTTCCCGGTTTAAATAAAAAGGGAAAACGAATTTCCAAAGTTCATTAGAAGCTGAAACAAGAACACGGCACCGAAATCAATGTTGTGTTCGGGTGGAAAATGTATGCTTCAAATCAAGTTGATATATCGAACGCGGAAAAGGGAGGTGCCATGCTAAAACAGGGGAATCGGCGGAGAAGAGCACAACGAGGGATGATGTGTAATTCATGGTCAGTATCCTGGAGAGAGGGACAAAAAGATCATTCTAGAACTACTTGAAAAATATGAATAATAAATTATCATGTCAAGAAAAGTGTGTGCACTTGCTAATGTTGTCAAAAGATGGTACTTAAGCTCAGGAGATAAAAATTAAATGGAGAAAACGACGGGTTGGTTTGCTGTCCATTTCGATGAAGGCTCAATTGTGGTACCAATAGCAAGATGCATATTATCACATAATATTCTCGTTTTATTTTAACAAAAGTTCATAATATTAAATTTTGCGCCATATTGCCATATGTCACCCTCGAGCTTAAGTTCTTCCTCTCTGTACTTGAGCGGACAAGTTTATTAAATAGAGGAAAGGATTGGGTTGGTTTGCCATCTATTTCTATGAAGGCCCCAAATGTCTTACCAGCGGCGAATCATATTTTACTGTTATTTTAGTAAAGTGCACATACCTATATTCATGTGTATGCCATGACACTCTCCATCCGAATTCTTTGTCTATGTTTCTCTACGTTCAATGGATGAAGCACACAATTGGATCGGTTGGTACCACCAAAGCTCTGGACTTGGCACTTATTTGGTGATCTTACTGATGCATGTCAACTCTTCACATTCAAGAAGAGAATTATTTTGAACAGCGAAAAATATTGAGTGCAAGTGAACGAGTTTGATGTTGGCACCATTGCACCAAATGTTTCCCCGTGTAAGTGAACATCACGCGATGTGGCGGTACAACTACAAATAAACGTTTCCTCTAATAGCGTGTACGTCCTGTACAGCTGCAGCCATGACACCTCATACATCTGACTCTTTTATCTCCCACGAGCGTTTATGCAAGAAAAAAAATAGTAATTAAATTTCCCGATTCTCATTACAGCCTAATTGAAATACTGCGTACCCACCTGACTCCCAATGATCCAACTGAAAACGAATTCTTGAAGTCCAAATGCTGAGGAAAAAAACTCCCGATACTGAAATCGATGTTGTAACAAGAGGAAAATGAATGGTTCAAACTGAACATCACTCGCAATCCGAATAGTTCTGTTAGGTTTCCTAAGTTCATCGGATCTCTCACTTGTTACATTTCAAAACAAAGTATTCCCTCCTGTTCGACAAGCTCGATGTTCGGACAACAACACTTTCAAAACCGTGGAGTATATGAAGTTTTGAAAGGAAATATGAGAAGAACATTCAAATGACGAGTGACACATTGAAATACCCCGAAACCATATATTCATGTACTATTGCCTCCAACAAAAAAAACGCATTCTTTTTTTAGGGTAGAAAAATATTCTAGAAGTAAAGCAAACAAAAATTAAATCATGATGCCAAGCAAAGGATACACTAATGCAGCCAACAGTAGTAGGTATTTGAGTTTCATAAGAGAAAAAAAAGTTGATTAGTACCATTTATTTTTTGCCAACATGAAAGCTTTGTTCGTCAGTGAATTATAAAAATGAAGCGCTAAGAGTATCTTCAAGAGAGCCCCATAAATTAACCTCCCTTAAGTATCTCATCAGGCAATGGTTCATGTCTGCACTTTTTCATAGTTGATTAGTCCTCTTTATTCTTTTTTCTATTGGATAGTTGATTAGCACTTGACCGGATGAGTTATGTAATCTTCTATAGGATAAAGGATGGCTCGGTTTTTGCCTTCTTTTTCCATGAAGGTCCCAAATGTCTTTCTAACGACAAAGATACATAACAGTTACTACTAGTATATTCATGTGTGTATGCCGCATTGTCATATGTCACTCAGTCTTAAATTCTTGATGTTTGTTCTCTACGAACTGGAGTAGACAGAAGAAACATCTTGGCAGGTACAACCACAGCTCAAGACTTGGGCTCTTGTCTGATCTTTGCAGACGTGGCGTTGTTAACTACGAACACACATTCTAACAAAAGAACAGTTCCTTAAGAGGAGAAAACAGAGGACAAAGATGTACGTGCACATCAACACGCTAATTATTACATTTTTCAAAACCAAAATTCTATTTCGAGAAATAGCTCAAATTATTTTCAATTGGTCTATCCTGTATTCCCTTACGACATCTAGCTAGCATTCTGGACTACGGTTCTTTGAGCTGACAATTGTGAGAAAAATATGTGCAGGGAGATTCTTTGTTACCCTCTCGGAGTTTCTTACAACACATCAGAGTCAACTGACGTTATATATGTCTGCTGATCATCAGATAATTAGTAGATCAGCGGGGTTTTTTAATCATAATTTGCCGGCAAGACGCGAGCATTTAATCGTTGATCTGAATCAGTCAACCATCCCCTTCTGCCCCACGCTCATTTTTCCAGTAGCCACTTGTGTTGATACTAATATATATAAACCCATGAGGAGCATCATGTCTTAGCTATACTCTACTTTCCTTTCCCTTGGACTAATCCTATAGACTTACTAAACTAATGACCATGACCTGCTGTTCAAGTTGTTAGTTTCCCTTGGACGTCAACAGCGTAATGGAGCTAGGGTTATCTTGTTCAGGTACAAAACAAAAATATCAACACTAGCAGGTACGTAGTATGGTCGCACAAGTCATGCATCAACTTGCCCGGCATTTGGAATTGAACATATATTGGCTAAAATAGTGACTTGCCTTTCATGCTGCATCATCAGTTTAATTACTTTCTCTGTCAGAGAACCGTCGTCTCTGACCACTCTTGCGCCACTGGCGCGTGCGTATGGTCTTAGTCTTACAGGCGATCAGCTCCATGATGGTTAAGTATGTAACGGTGGTCATGGTCCAAAGTCCAAACAAACTTCTAAGTTTGGTTCTGGTTTTAGTAAAAGAGAGAAAGAAATGGGATGGCTAACTGTTCAGACAGATTCCCATTATTTCACACTGATGAGATAACTCTGCATACGGGTCAGAGAGTAATGCTTCGTTTGATTGCTCTTTAGCACATGAAACGTAGTAGTAACTTTTTAATCGGTCAATGCCAATAAATCTTAGTGCAAATCTCTACCAAGAAGTTTCCAATCTAAGTTATTGAAAATATTGGAGCTTATCTATCTGGTCGCTCATTTTCGGTCCCACCTTTTTATTTTTGCTTCTGAAACTGTGCGCTACAGCAGAGCTTCTATGATCCCAGATAATAAATGAATATCGTTGCTAGTAGTGGACAATTACGTGTCCTTACTCTTGGTAAATTTTAACCAAATCTATATGTTTATATTTTATATATAAGGAGAAATCACTATGTTTTTGTAGTTCAGATCCCTTTACTCAAATGGCTCTAGTATTTATTTTTCCCAGCCCCAAGGACGGCATATGCTGTTGTACATGTAGTTCTAAATAGAACAACATAGCGTCGTCTCAAGAGTTCGATTGAATTAGATTCGCACTTTACAGATATGCCTAAAACTTCCTGACATGATTTAAGTCAACAGAGACAGATAGAAAAATAGAAACTTTCAGGCAGCTTCCCACTAGCCCGAATACACATGGTGACGTGGATGGTCATTTCTGAACTTTGACATACATAATTACATATACACAGTGACGTGGATGCTCCTAATTTTCACGAAGAGCATATCAGAGCGTCGACACATCAGCGTCACAATTAAAAGAGGTCCTCGCTGTCGTATCTTTATCTGAAAGGTGACAAATGTTAGAACATGTACGAGTAACAGAAAGGAGGAGCGTCAATGTGCTAGCCGAGAAATTAAAGCAACAAAGACTTGGACGGAGATCGCCAACATCCGAAGCAAAACTACAGAAATTCAATCTACCCGGCCTGTCAGACATCGAACATGTGGCTCCCGATCATGATTAAGTCAACAGAGACAGATAGAAAAATAGAAACTTTCAGGCAGCTTCCCACTAGCCCGAATACACATGGTGACATGGATGGTCCATTCTGAACTTTGGCATACATAATATATACAGAATGACGTGGATGCTCCTAATTTTCACGCAGAGCATATCAGAGCGTCGACACATCAGCGTCACAATTTAAAGAAGTCCTCGCTGTCGTATCTTTATCTGAAAGGTGACAAATGTTAGAACATGTACGAGTAACAGAAAGGAGGAGCGTCAATGTGCTAGCCGAGAAATTAAAGCAACAAAGACTTGGACGGAGATCGCCAACATCCGAAGCAAAACTACAGAAATTCAATCTACCCGGCCTGTCAGACATCGAACATGTGGCTCCCGATCACTGCAGAGGAACAGTGAACTATCTTTTTCCGAGGGATTAACTTGCCTTATCATGTCTCTTGATCGAGAGATGCACTAAGCATGGAGAAAACGAGAATCTCCTTTGTTTCGTTTATCACGACATGATCTGCGTATTCTTTTGATATACAAAGCGACATGTATGCAAACTAATAAATGAACTGTCAATAATTTCAAAAAAAAAATCATCTCACTTCCTGTCTCTATCCAACGAAGTGCGGCACGCATGGATAAGACTGTTTATCATAAGAGGGGATCTGGGGTAGTTGAACACAAGTGGGCTCTAGAATTTCCATTTTTTTATGAACTGAGGAGTGACTAGCAGCAACAGGCCAAGAGAAACAACAAAAGCAGAAACTACACCGTAAGTAGGGGCCCCTGGTAGCATCCTGATCACGCCATCATTTCCGTGTGGAGACTAGAAATAACTTAAATTGGGAACGAGTTCGGCCAAATCGTCCAACAAAATCAGAGGAAGGAGGATCATACTAATAACATCGATAACCTTGGATATCATACTATATTGAGCAGCTTGATCATCGATGCGTGCAGGTATGTCACACATCAGCAGGAATGGCACGAGCCGAAATGTCAGGCCACTGAAAATCAGTGGAAATGCAATACACAAAGCCATTTTCGGCTCGTCAAAGTCAAATAATTCTTAAATATTAAATGAAAAGCATGTGTTATGGTCATTTGCGTAGTTCATAGAAGAAAACTTATGCACATCTTCTGTAGATGAAAATTAGATTTTTCTTTTTAAACACATGGCACATTTAAGTGAAAATTGTCAACTTGTATTTTTTTTGAAGCTTGAAAGTACTAAGTTTATAAAAGCGCACAAACTACAAATGGATGTATATAAATGGGCATCTCCAAAGCGAAAAATTCCTAATCCCTCGTACAATATCGATGTGTCTGTAACACGTGAGGCCACTATGCAATACAAGAAATTGTCCCTATGTAAAATTGTGTCAATGAAATTCAAGATCGATCGAGTTCATATGAAGAATCCCAGTCCATCATACAAATTAATTAAACGGTGTCTATAAACTTACCCCACGATATCACTGCTGACTCGATTATCCCCCCATTTAATATTCATAATATTATTTTATGGTGCTCCGTCGATACCTGGACTTTCAAAAGGTTCTCGTCCCGCCTGAAACCCGCACTACGACGGCGCCGAGGTTAATATGAACCCGCTGTGACCGTTTGACATTTCCATGCACTGGGGCTGCCAGCGAGCTTTTGAGGACCCTGAGATCGGCGGGTTTCATGACCTTGTCCCCTAAAAGGCAGAGGTTGACGACGATCCTTCGTGCCGGGAATCGAACAAGCTGATACGCCGTCGGCGACTGATAGCCGCAAGCAATGTGGCAATTGTCAACTGTGTGAAACTAAGGGACCATGGCAGCTGGAGGGGAAGCAGCACACGGTTCCAGCCTCACGTTAACATCAGCATGACATGCTCATGACATTAGCATGGCAGGCTACCTCGCTGAACGTTTGCATCACGTTATTTCTCTGTCATTACTCAGTATATCCAAAACCGAAATTTAACCACCATATTCAGATATATTTGTAATAAGTTAATCAAATATTATAATATTTCCATTTTACAAAGGTATTTTTCTACAAACCAACAAACATAAACGAAAGCCAGAAAATTTGACCAAATATACAATAAAATCCTCGTGTCAAGGTAACTCTAATGGGAGTTGATACTTCAATGTATGTTAGGATTTTTTTCTTATGTCTTTGTAAGAGGCAGTGCCTAAATACGACATCAACCAAAATGTCCCGAATTGAGGAGGATGACTACAAGTTTTATTCTAAAAATATCAATCTTTGCCTGCTAAATATAAATTTTGTAATTATTACAAATTCATAAAGGTGGTGTTTGGATAGTGGGAAATGTGGGTTGGGTTTGGGATTAGTGGAGGGTTGAGATCTAATATGATGGAATGGCCTAGATCTCCTATCTCATTTCCTTATCCCAATTCCCGTTTCCATTTCCCAATTGCCAAACGCCCCCAAAGGGTAGTGTTGTGCCAAAATCTCTCTAACTTGATAAAAATTTCATAAGGCATTCTCCATTCAGTGCTCAACTGCACAAAGAGGCCATCCCATGTGACGTCCTCTTGTTGTGGCAATACTTGTAGGGTGGTGATGATTTCTACTGTCACATTAAAATCAGGTCACTTAGTACTCAAAAGTCCACACCCTTTGGTACTGAGATAGAGATAACACCCAAGGCCTTTCATCTGTATGAAAAGCAAGGCCATGAAAGTTATCTCAATATCGAAACCAAAGGGCACGGATTTCTAAGTACTAAGTGGCCCGATATCAATGTGACAGAATGAGTCATCATCACCCTACAAGGATTGCCACAGCAAGAAGACACCACATTGGATGTCCTGTTTGTGTTGAAGTACATAGGACAACAGGAGAATGCCTTGTGGAAAATTTTGACACAACACAAAAACCCTTTATGAATTGGTAGGCAATGATTGATACTAATTTTAGAATGAAACTAGTAGCCATCCTCTTCAATTCGAGGCTGAACAAGACGAAGGATGAAGTTGAGAAAATATTGGTTTGAGTTCAAATCTGTTGCCAATGTTACGATGGGGCTCGCAATATGAAAAGGAGATCTATTAAAGGCGTGCTGAAAACATTTATATCACTAAAAAGTCGTCTATAGCTTATTACATCCATTGTTTCGCATGTCGCTTCTAATTAGGGGTCTTGTTGTTTGTTGCCAATGGAAGTGATGGTTGCACGTGGTTTTCTGATCATCTTTCTCTTGCTTGATAGTACTATTTGGGTACGTTTCTCGTAAACATCATGACACATGCTTCCAGAAACGTTAGTCTTCATAAAAGCAAATGAATGTGAGGCAAACAATAATTTGCTGTGTTTTGTTGCTGCAGAATGAATCCAAAGGACACCTTCGTTCCCAGCGACGTGCTAAGGCCACTCTCAGTGGCCCTTTCATTTGGCATTAATTAGATGCCACATCAACAAAAATGATGACATGGCAAGGAATTAAATATAAAAGAGAGGAGGTGTCTTCCATCCCCATGAAAGAAAGTAGGCACTATTTCCAGCACGATGAAAGCTAAATAAAATTGCATTGAGAGTCTTTCCTTTCATTCTAAAGGATCCTAAACAATAGATGATGTCCATCTAGCAATAGTTTAATATATTAACAAACCTATGAAGGTGTGACATGAAAGTAGGCATTGAGAGATGCGGTTTCATTTCATAAAATAAAAGTACAAAACTGATGTGTCACTCTTGAAAACTACGAAATGAAAGTGTCCATTAAGATTGGCCTAATGCATCATCACTAATTAATTAGTGACCCATCACAACGGTCTTTCAGAAAGAAAAAAAAATCACCACATCATACTAGCTAATAGCGTCGAATTGATAATTAGTACTAGTATAGGATGATGTTCTCGTGTACCTGCGCGCGAGAGGAAGTCGGTGGCTGGGAGATGACGAGCAGAGGTACTGAATATGACTTGGGGCTGCGCTAAGCTGTCCTGCCCGTCGACGGGCTTTGTCGACATGTACAATGCAGCGCGCTGTGCGTCCTGATCACCCCCGCAAGCCTGGGGGCCACGATCCGCGCGACATTCTTGAACCGGCGGTACTCGTCGTGGCAAAGGGCGATGTCGACGCCATCGTCGTCGTCCTTGTACTGGCAGTCGGACACGAGCAGCCGGACAAGCCTGGGCGCCTCGACGGTGAGCTTCCCGTCGGAGCGGATGGCGAGGTCTCTGACTCCCGCGGAGCCGAGCAGCCCCGCCAGCGTGAGCTCCTGCAGCCGCGGGCAGCACTGCGGCGTCGAGACGAGGCGGCCCACGTCGAGCTTGCCGNNNNNNNNNNNNNNNNNNNNNNNNNNNNNNNNNNNNNNNNNNNNNNNNNNNNNNNNNNNNNNNNNNNNNNNNNNNNNNNNNNNNNNNNNNNNNNNNNNNNNNNNNNNNNNNNNNNNNNNNNNNNNNNNNNNNNNNNNNNNNNNNNNNNNNNNNNNNNNNAGCCCGCCGCGACGGGAACCGCCAATCCTCAGCTGTTCCCTCTCAGGGGTAGGGGGCGTAACGCCGCGACGGCGGCTTGTTGTTGTTGTTGTTGTTGTCGCAGTCTCCGCCGTACGCGACGTCGATGTCCAGGCGGTCCAGATCGTCCCCGCGGGGGAGCGCGGCGCCGATGGCGTCGAGGGTCGCGGGGGTGAAGTGAGGCCGGTCCGCGCGGTCGACGAAGGCGACCTCGCGGATCTGCGTCCAGACGCGGTGCCAGCGGCGGGAGAGAACGCCGGTGCGCACGGCCGTGCGGATGCAGCAGAGCGGGTCAGGATGCACCGGAGGAGGTCGTCGGGGAGGGCACTGATGCGGTCGGCGCCGCCGCCGCTATCCATGACGTTTTTGTTAGGGTTTCACGTGGAGAATTTACTGTTGGCTTGTACAAACAACGTGAAACCAATTTGGTTGGGAAGCATGCGTCCCATTTTAGATATGAACAATGTAATAATAATGGCCGTGCCGTAGCAACTCCAAATTATTGTTTGTTTACATTTTACAAGTTGTTGCGTCGCCTCTTTCGAATTTGGCCCATGGATAGGCCCAAATTTGAAAGTCCAGAGCTAGTTCTAGTCCAATTCAACCAGTATAATTTATGAATTTTAATCATTTTCACCAATTATCTTGTACCACAAACCATTTTGGTTTGTAAATATATGGTTATCGGCATTTCGGTCCTACTTATGCGAAGTACTATTTTGTTATGGATGCTTCTTGTACTTCAGTTCAGTCAGATTGTCAGAATGATACTAGAAGGATAGGTTCATGGGCCTTTTGCTTTCTCCCCGGATTTTGTTGTGATGGTCTGTATTTTAGGCTTCGGGCCTGTTGGCCCATATCTGGCCCGGGCCTACCATAAACCCCAAAATCCTCCATCCCAAGGCATCTTCCCTTTCGTTTTGCTCGCCCGCTTCCTCGCCGCCGAGCCCGCCGCCGCCTCCGTCCACCCAGCGACGGCAGCTGGCCGCACTTCCATCACCTTCTTCCCTCCCCACTACGGCGCAGCAGCAATCAATCGGTACTTCGGGCTGTTCTCTTTAGCGGAGAAGGAGCCCTGAAACCCTAGAGCCTACGCACTACAGCAGCGGCTGGCAAGATGAAGAACCTTCGCCTTATCACGAGGCCCTCGCAGCAGCTCCAGCTGCAGCTCGACGGCGAGACCGTAGTCGCCTCCGCCATCGATGCGGAGCGCCGCCGGGCCTTCTTCGCCTCCTCCGCCAACTTCCTCTACACCGTCCACCTCGCCGCCTCATCCACCCAGGGCCAGGTCTCTAATTTCATCACGCTCCATACCGTTCAGTTCCATTTGGGTGCACTAGTGAAATTATACTCTACTGTTTGTACCTTGTCAAAGCTATTTTTTACTCTAGAAACAAGCAAGCAAACACTTTGCTGATCTTCGATTTACTAGCAAGCAACTCTAAACAAGCTTATTTTTTCAATCCTGTAAAAGATATCGATGTGCTGAAAACAATATATGCTAGTACTTTGTGTTGTGGCGATTGATCTGCTTTTATCTCTCTTCTGCAGCAACCCCTGCAATGGAGCAAATTTGACTCTGATGCAGAGGAGGTGGTTCTTGAGCCTGGAGACTCCATAGTTGCCATGGATTATCTCATGGAGAGGGAGTCTCTGCTCCTTGGTTCGTCGTCTGGTTGTTTGCTCTTGTACAATGTGGAGGAGAAGACAAGCCAAGTTGTAGGAAGACTGGAGGGTGGTGTCAGCACCCTCGCCTCTAGCCCTGATGGAGCCCTTATCTCTGTAACAACCGGATTTGGGCAATTGCTTGTCATTACGCAGGATTGGGAAGTTCTGTTTGAATCATCTCTTGACTCTCAAGTATGTGATTTCTCTATTGAATCTTACTGAAGGCGCGGTTTCTTGTGTTCTCAAGATTTTAACAGAGTCTTCTTCTCAGATTGTTACTGCAGGTGAGATGGAAAGTTCTTGTGGTCAGGTCCAAAGTGCAATTTCTTGGCGGGGGGACGGAAAATATTTTGCTACGCTTGGGGCTCTTTCTGGCTCTTCCAACCCAACGAAACTTACTATCTGGGAACGGGAATCAGGAGAGGTGCATTCTTCTTCAGATACCAAGACTTTCATGGGAGCATCACTGGATTGGATGCCAAGTGGAGCCAAGGTTGCCACAGCCCATGATCGCAAGACAGAGGGAAAGTGCCCTCTCATTGTATTCTATGAGAAGAATGGCTTAGAGAGGAGCAACTTCTCTATTGATGAGCCAGCAGAGGTTGCCATTCAGGCTTTGAAATGGAATTGCAATTCTGAGCTCCTAGCTGCTCTTGTTTCCTCTAGCCAGTATGATGCTATTAAAATATGGTCCTGTAGCAACAATCACTGGTACTTGAAACATGAACTGCGGTTCACAAAAGAAGAGGGTGTGAAGTTCTCTTGGGATCCAACAAAACCACTTCATCTGATTTGCTGGACACTGGGTGGCCAGGTCATTATGCACAGATTTGCCTGGTCTACTGCTGTAAGTGAGACTTCGACAGCACTAGTTATCGATGGTTCCCATGTCCGTGTTACTCCTCTCAATTTGGGTCTCATGCCTCCCCCCATGTCTCTGTTCCACCTTGCATTTCCTTGTGCAGTGAATGAGGTTTCTTTTGTGTCCAATCACTCAAAGAACCATTTGGCCGCCTATCTTTCAAATGGCAGCTTGTGTGTTGTAGAACTTCCAGCAACAGATACTTGGGAAGAGTTCGAAGGCAATGAAATAGGTGTTGAACCCTGCTATTCTGACTTCACTTTGAGTAACTGTATGCACCTCACTTGGATAGATACACGTACCTTGATTAGTATCTGNNNNNNNNNNNNNNNNNNNNNNNNNNNNNNNNNNNNNNNNNNNNNNNNNNNNNNNNNNNNNNNNNNNNNNNNNNNNNNNNNNNNNNNNNNNNNNNNNNNNNNNNNNNNNNNNNNNNNNNNNNNNNNNNNNNNNNNNNNNNNNNNNNNNNNNNNNNNNNNNNNNNNNNNNNNNNNNNNNNNNNNNNNNNNNNNNNNNNNNNNNNNNNNTCTTGTGTTCTCAAGATTTTAACAGAGTCTTCTTCTCAGATTGTTACTGCAGGTGAGATGGAAAGTTCTTGTGGTCAGGTCCAAAGTGCAATTTCTTGGCGGGGGGACGGAAAATATTTTGCTACGCTTGGGGCTCTTTCTGGCTCTTCCAACCCAACGAAACTTACTATCTGGGAACGGGAATCAGGAGAGGTGCATTCTTCTTCAGATACCAAGACTTTCATGGGAGCATCACTGGATTGGATGCCAAGTGGAGCCAAGGTTGCCACAGCCCATGATCGCAAGACAGAGGGAAAGTGCCCTCTCATTGTATTCTATGAGAAGAATGGCTTAGAGAGGAGCAACTTCTCTATTGATGAGCCAGCAGAGGTTGCCGTTCAGGCTTTGAAATGGAATTGCAATTCTGAGCTCCTAGCTGCTCTTGTTTCCTCTAGCCAGTATGATGCTATTAAAATATGGTCCTGTAGCAACAATCACTGGTACTTGAAACATGAACTGCGGTTCACAAAAGAAGAGGGTGTGAAGTTCTCTTGGGATCCAACAAAACCACTTCATCTGATTTGCTGGACACTGGGTGGCCAGGTCATTATGCACAGATTTGCCTGGTCTACTGCTGTAAGTGAGACTTCGACAGCACTAGTTATCGATGGTTCCCATGTCCGTGTTACTCCTCTCAACTTGGGTCTCATGCCGCCTCCCATGTCTCTGTTCCACCTTGCATTTCCTTGTGCAGTGAATGATGTTTCTTTTGTGTCCAATCACTCAAAGAGCCATTTGGCTGCCTATCTTTCAAATGGCAGCTTGTGTGTTGTAGAACTTCCAGCAGTAGATACTTGGGAAGAGTTCGAAGGCAATGAAATAGGTGTTGAACCCTGCTATTCTGACTTCACTTTGAGTAACTGTATGCACCTCACTTGGATAGATACACGTACCTTGATTAGTATCTGTTGCAATAGTGAACACTGCAATTCAACACCTGTGGGGGTCAGTGAAACTAGTAACCTGGTAGGCAGGCATGATTCACGGTTCTTTGTCAATGAGATTGAACTTGTATGTTCTGAGGATTCTTTGCCAGGTTCTGTGAGTTCATCTGGCTGGCAAGCTAGAGTATCAAAGGAAATGACCTTGGATAGTTCAGTCATTGGAATCTCTGTAAACCCAGCAAAAAAAGGTTCAGCCTTCATTCAGCTAAACGGAGGAAGGGTTGTTGAATACTGCTCAGGTACAAACATGTTGAGAGTGGGTGCACCAGCACAAGTTTGTGACATTGGTTCTGACAACAGTTTTCCAGCATCATGCCCTTGGATGACTGCTATTCTGTGTCATGAAAATGGTGCAGTCAGACCCTTTCTATTTGGACTTGATGACAGCAGCAAGCTTTATATGGGAAACAGGTTATTGAGTAACAATTGTAGCAGCTTCACATTCTTCTCCAGTGCTTATGGAGCTACTGAGCGGGTTATGACCCACTTGCTTGTAACTACTAAGCAAGATCTTTTGTTCATCGTGGATATCAATGATATTTATCTTAAAAACGGCGCATTGGTAATTGACAGCCATGTCAGTAGTCATCCTCGAGGAAACCAAAGCAAAGAGCATATCATTGTGTGGGAAAAGGGGGCAAAGTTGGTCGGTGTTCTCCATGGTGATGAAGCAGCTGTCATAATGCAAACAAACCGTGGTAACTTAGAGTGTACCTACCCTCGAAAGCTGGTCCTTGTTTCAATTGTTCAGGCTTTGGTTCAGAGGCGTTTCAAAGATGCAATGGACATGGTAAGGCGGCATAGGATAGATTTCAATATTATGCCTGACTATTGTGGGTGGGATGTTTTTATCAAGTCATCTGCAGACTTTGTTAAACAAGTTAATAACCTTAGCCACATCACTGAATTCGTTTGCGCGATTAAGAATGACAATGTCAGTGGGAAACTGTACGCAGCCTATATATCTTTTCCCGACCAGTGTGCAGACTCAGTAGCCAACACGGGAACAGATTCTGGAAATGGGAACAAAGTCACCTCTGTTTTGATGGCAATACGGAAGGCCCTTGAGGAACAAGTAGAAGAAAGCTCATCAAGAGAACTTTGCATACTGACCACTTTGGCACGTAGTGAACCTTCATTGTTGGAGGAAGCACTAAATAGAATAAAGTCCATTCGGGAACTGGAACTTCGTGGGCTTGATGATGCCAGACGAAAGCTCTACCCATCTGCGGAAGAATCTCTGAAGCACTTGCTTTGGTTAACAGACCCTGAAGCTGTTTACAATGCTGCATTGGGGCTATATGATCTGAACCTTGCTGCTATTGTTGCTTTAAATTCCCAAAAAGATCCAAAGGAGTTCCTTCCTTTTCTCAAGAGTCTTGAATGCCTTCCTCCTGCTATAATGAGGCACACAATTGATTTAAGACTTGGAAGATATGCAAGTGCTCTGAAAAACATTGTTTCTGCTGGTGATGAGTATCACGAGGATTGCATGAAACTCCTTAATGCCAATCCTCAGCTGTTCCCTCTCAGTCTGCAGTTATTTACTGAGCCAGATAAGAGATGTCAGATTTTTGAGGCATGGGGTGACCATCTTTCTGAAGAGAAATGCTTTAGAGATGCCGCATTGACTTACCAGTGCTGTTCATCGTATCAGAAATCTTTGAAAGCTTACCGTGCCTGTGGGGACTGGAGAGGTGTGTTTACCGTTGCAGGTCTTCTGAGGTTAAAGGAGGAAGAAATTGTTCAACTTGCACACGAGTTATGTGATGAATTCCAAGAAATTGGAAAGGCAGGAGATGCTGCTAGAATAGCACTGGAGTACTGTTCAGACGTTGATAGAGGTGTTAATTATTATATCGCGGCAAGGGAGTGGGAAGAGGCTCTTAGAGTTGCCTATATGCACAGTAGACTGGATCTGGTTGAAAATGTTAGAGACGCAGCTTTGGAATGTGCCGCATCGCTGATTTCTGAGTATCAGGAAGGATTGCTGAAGGTTGGAAAATATGTAGCACGTTATGTGGCTGTGCGGCAGAGGAGATTGTCTCTTGCTGCCAAACTCCAATCAGATGATCGGTTTATGGATGTTGAAGACGACAACGTTTCGGAAGTGAGCACTAGCTTCAGCGAAATGAGTGCATATACCACAAGGTAAGATATTACTCCATTTCAAGTTGCTCAACCCTCATTGTTGTGGTTGCTATTTCCCTCTTTCACTATAATCCAGATTATCTTGCTTTCTTGGGAAGGGATTCTAGAATTGAATTCAACGGGTGTCAAGTCACCTATTGGTTGTTTATTCAGTGGTGTGAAACTCTGAATGTGTGAACAGGTCAACCAAGGCGTCAAGTGCTTCAGTGATGTCAAGCAGTGCCAGCAAGTCACGAGGGGCAAGGCGGCAAAAGAAAGGTGGCAAGATACGAGCTGGAAGGTTGGCAACTTGCATTGTGTCTGCAGATTTTCTATTTAAATGAAAGCCGATTGGTTTGTTTCAATTTTTGGTTTGTGTTTGTTCTGTGGGCTCACCTCATCCTTTTACATATGCAGTCCTGGGGAAGAGATGGCGCTTGTGGAGCATCTCAAAGGGATGACGCTGACGACCAGTGCTCAGAATGAGCTGAAGAGCCTGCTTATTGTTCTGTTACAGTTGGGAAAAGAAGAAATCGCTCGTCAGGTGCAGCAGGCTGGTGATAATTTTGAGGTGTCTCAGAGAGCAGCAGTTAAGTTGGCCGAGGATACAGTATGTGACAACAAGGTAGACGAGAATGCACATACTCTGGAGCATTACATGAAAATGCTGAGGTCTCTTGAGCCTGGTCTTAGCGAGGAGAGTGCTTCATGGCGAATCAGAGCATTATCTCCCCCCTGATAGAGTAGTATTTAGTACTTTTCAGTCCAGTCCTCCAGTACACTCCACATGGGTGACGGATTAAGCACCGATTACCAGTATTAAGGTTGAGTGTTGTTAGTACTATCTGTTGTGTCTTTTTGTCGAGTGGCAAGGCTATGATGGGGTTGTGAAATACGTTGACTGGCTTGCATACACAGATGTAAGAAGACAATATTCTGTCAGACTGGGAACGCACGCATAATATTATTTTCCTCTATGGTAATAGACAGGACCAGCCAGAGGTCCATTTAAAAGTATTTGGTGCCAAGTTTTTATGGTCGACTAAATTAGAGCCTCTAACCAAACAAGTGATTATCTTTCAACGGCGAAGTTGTAGCTGAATTAAGAGCGCCGCCTCTAACCAATACTGTCACAAGGTACAGAAATACTTGAATACGGAACACTTTGAAGTAGTAAATATATTACCTGTTCCACTTGAAATAATATCTTATCAAGATCATATAATTCATAAGAAATTGATCAAATTTCAAGAAAATAACTCAATTACTTGATAACTACAAGTGGGACTACACAAGCTCTTGAATTGTACCGTTTTTGATATTTTTGTAGTAGATATGTTGGTGATAGTGTCTTTGTGATTACATTCACGGTAGATATATGTTAGATTTGATTTTTTAGCTTATTCACACTTTGAGATATGGGTGCACTCGTTTTGTCATATGAAGCATGTGTATAACCAACACGAGATGAAAGGCCTCTTCTGTGAACACAAGTAACAAAACAAACTTCCTATGGATAACAAGATTTTGAAGTTGTTTGAATCTTATTTTCACCTTTACACCTAGTGGGAACGAGAACAACAATAGGAATATTTAGGTCAACATGAAGTCCACCAGATTGTAGCTAGTGTAAACAAGCTAAGTCAACATGAATTCTTGGAATAGCAAGATTTTATTAACTTCTAAATCTTTTGTATCCTTGTATCTACATTATTTGTCACCTTCCACCTAGTGGGAATGAGATCGATAACGAGCACGCTTGGGGTTGGCAAAATCATGAACTTCTCACAAAGTTAGCATGTATCATATGAAACGCGCTTCAGTGCAAGGTAGTATGAACGAGAGAACCCGGCATGAGAATTTTTGAATACCTCTTTCACTATAATCCAGATTATCTTGCTTTCTTGGGAAGGGATTCTAGAATTGAATTCAACGGGTGTCAAGTCACCTATTGGTTGTTTATTCAGTGGTGTGAAACTCTGAATGTGTGAACAGGTCAACCAAGGCGTCAAGTGCTTCAGTGATGTCAAGCAGTGCCAGCAAGTCACGAGGGGCAAGGCGGCAAAAGAAAGGTGGCAAGATACGAGCTGGAAGGTTGGCAACTTGCATTGTGTCTGCAGATTTTCTATTTAAATGAAAGCCGATTGGCTTGGTTCAATTTTTGGTTTGTGTTTGTTCTGTGGGCTCACCTCATCCTTTTACATATGCAGTCCTGGGGAAGAGATGGCGCTTGTGGAGCATCTCAAAGGGATGACGCTGACGACCAGTGCTCAGAATGAGCTGAAGAGCCTGCTTATTGTTCTGTTACAGTTGGGAAAAGAAGAAATCGCTCGTCAGGTGCAGCAGGCTGGTGATAATTTTGAGGTGTCTCAGAGAGCAGCAGTTAAGTTGGCCGAGGATACAGTATGTGACAACAAGGTAGACGAGAATGCACATACTCTGGAGCATTACATGAAAATGCTGAGGTCTCTTGAGCCTGGTCTTAGCGAGGAGAGTGCTTCATGGCGAATCAGAGCATTATCTCCCCCCTGATAGAGTAGTATTTAGTACTTTTCAGTCCAGTCCTCCAGTACACTCCACATGGGTGACGGATTAAGCACCGATTACCAGTATTAAGGTTGAGTGTTGTTAGTACTATCTGTTGTGTCTTTTTGTCGAGTGGCAAGGCTATGATGGGGTTGTGAAATACGTTGACTGGCTTGCATACACAGATGTAAGAAGACAATATTCTGTCAGACTGGGAACGCACGCATAATATTATTTTCCTCTATGGTAATAGACAGGACCAGCCAGAGGTCCATTTAAAAGTATTTGGTGCCAAGTTTTTATGGTCGACTAAATTAGAGCCTCTAACCAAACAAGTGATTGATCCACTGATTTTGCCCATAATGTCACCTTTTGACCTGAGTTGCAATAGAGCTCAAAGAATTCACTGACGGAATTAGTGAGGCTTGATCCATTGATTTGTTCAAAGAATTCAGTTGTCTTCATTCATTAATTTATCCTAATGTGTACAAAAGTACCATGCTCCAAAACAGAGAATTGGCGGTTCGCACTAAAAAGTGGATATAAGGATTAGTGAAATAAGTTCAAAGACAACACTATATACAAGGATTAGTGTATATAAGGATTATGCTCCAAATAAAATAGAGAGAATTGGCAGTTGGCTCTAAAAGGTGATTCATACAGTGGCAACATCGTCCACCCTAATAGGCATCATCATAGAATTCATAAGAAATTGACCAAATTTCAAGAAAATAACTCAATTACTTGATAACTACAAGTGAGACTACACAAGCTCTTGAATTGTACCTTTTTAATATTTTTGTAGTAGATATGTTGGCGATAGTGTCTGTGTGGTTACATTCGCGGTAGAGACGTTAGATTTGATTTTTTAGCTTATTCACACTTTGAGATATGGGTGCACTCATTTTGTCATATGAAGCATGTGTATAACCAACACGAGATGAAAGGCCTCTTCTGTGAACACAAGTAACAAAACAAACTTCCTACGGATAACAAGATTTTGAAGTTGTTTGAATCTTAATTTCACCTTTACACCCAGTGGGAACAGAACAAAACAACAATAGGAATATTTAAGTCAACATGAAATCCACCTGATTGTAGCTAGTGTAAACAAGATAAGTCAACATGAATCCTTGGAATAGCAAGATTTTATTAACTTCCTAAATCTTTTGTATCATTGCATCTACATTATTTGTCACCTTCCACCTAGTGGGAATGAGATCGATAACGAGCACACTCGGGGTTGGAAAATTCATGAACTTCTCACATAGTTAGCGCGTATCATATGAAACGCGCCTCGGTGCAAGGTAGTACGAACGAGAGAACCCGGCATGAGAATTTTTTGAATAGCAACATTTTATTAACTTCCTGACTTGTTTGTTTTTTAGAGACCGTCCACCATCTATATAGGTAGGATGGGCCAGCTATTGCTTTCAACAAAAAAAAAGAAGTTCCCTCCTCTCCTCTCTCCGCTCTCTTGTCTTCTCCCAAGTTGAATGTGCCATTTCCTTCACAACATCGGGCGCTAGAATGTCTTCTCAAGTTCTCATTCATGCTCTTCCTAAGTTTCGGCGTCAACAAATGCTACTACTACGTTATCCACGCAGGGTCGTGGACTCAAGGATGATTCGTGGGAAGTTGCAGGTCTTGATTCAATGGATTGGGTTTCCTGATGAAAACACATATTGGATTGATTTTGAGGAGCTCGGACGGTTGTACCCTTCCTTTCACTTCAGGTTTGGGATTGTCTATCATCGTCGTGGTCGTGAAGCTGGCATATGAGGCGAGGGAGACTAGAACAATCCCTAGGATCGCAAGATTTATATTATATTTAAAAGTCATTTATATTTCATCAATTGTTTGTGTTTACCTTCTGCATAAAACTTTGTTAGGCACACCTCTATCAAAGAGGATGGAACTATTTGCTTCATCATCAATTATAATATATTGAATTGCTTATTTTTCTTAAATGTCGCCATATTCGTCTCTATTGTCCCATATACCATAGTTTGAAATTGCTTTACCCTAAACTAATGTTTATTTAATTGGTTCATGGCTTCGTAGACATTATTGGAAACTATGTGTTTCCCTAGATCTCTAATTAGCAGGAAACACACACACACACAAAAAAAAGCAGGGAACTTGTTCCGTACGAGTATTTTTGTCTTATATGCCTTTATGGTCGTGAGGCCTGCTTTTTCAAACACAATAAAATCATAGTACAGAGAATGACATGAAACCATAGATAATCTCTAACATAAGAAGTGGTATGGTGTATTTGTTGAGACTGCTATTAAAGAAATCAGAAACTTTGAGGTAGCATTCAAACATTAAAAAAAACATTCCAAGCCAACAACACCTGCATTTTGTGCGAGATATCTGCTTTTACTGCCAACAACACCTGCATTTACATTGGATAGCCTGGCAGTAGTGGCTGTTTCTGTTGCTCTATTTTGCCTGCATGTTGTATCCTGTGACAATTTGGTCTTGAAGACGGCGTTCGCGTGCTCCTCTCTGTGATGTATGATTTAGAGTTTTTAGTCAGGTACGTTGTGAACAAAATAAATGATATGTTATGATTGGCTTTTTGTATATTGCCTCTTGCAAGTTTCCTTTAATTCTCTTATAATATGCTTTATATTTTGAGTGCAAAGTTTTCTTCATGATTTTGGTTAAAGTTCAGGTGTGCCATTGTGTCTCTCATAGTCTCTGTTATTTTTCACCAGATTCTGTTATAGCCAGTTACCTGTTTTACACTTTATTTTGTCACCGACATGCTTAGAATGCCTTTTTTTTTCCTTGAGGAATTTGACACAAAATGTGTACTTCTTGTGAGCTAATTAGTTCAATTGGGCTCAAAAGATTTGGGAATCGTGTTCCACGTGAAGCCTCCATTCACGCTGCCGCTACACAGAAAGATAAAATTCAGTGCAAACGTGCATCATCAAGCTGTTCGGCATCCTAGGAAACTTTTTTTCCCCTGAAACAAGACGACGGCTTAATCCAACATACAGAAACTACTAATTCAGTTTTGACTTTTGAATCTAACATTCACTACTGGAGATCGGCAACATAACAAGAAATACCAACAGATTACCTAGAACGCGCATAGTTTCTTGCCCACGCTGACTGCCACCCTGAATATGCAGAGTTTCTTGGTGATTTTGGAATGTCTTTTATATATGGTTTTAATGACAAATTACTACCTAATGAATTAATTATTATTAGAAACTATGGAGATGATGATGAAGAGACAGTTGGAGAAGTGCTTGGGGGCGTGGAAGGACTGCAAGTGTGCAAATAAACATTAAAGTCCAATCAAGTCGGTTGTGAGTCCGTTTCGGACTACAGGATCAGTTTGTATTAAAACGGGCTCCCAAGTCGCGTATGGGCTCCGTTTTGGATGTTCTACATATAGAGAGAAACCTAAGAAGACCACTTTTTCAATGGAACCGGTCACATGACTAAATTTGGTCGGAGTCGTCAGAAATCGTCAAAACAATTTGACGTTCAGAATCTGTCCGGTGCTGCGCCGCCGTCATTTGGCCTTTGGGCCTTGTATCTCGTGTGAGCCCATTAGAGGGCGCATCCAGGGCCTTTTGCACGACTCTTGAGTCTTCATAAACAGTCGTCGCCACATAATTAGGGTTTGGGTTTTGATTGATCGTAAGTTTAGCCATTGTTACTTTCTTGTATACACGCGAGATGGCGAAACCGCTCAGACTACTTGACTCGGAATCCTACCTTTGTTATCGAGTTACTTGTTTATCTTGTTTTTGTTTGTTCTTCGTTTGTTGGTAGAAATTACCTTAGTGGTTTGGTTGATTGTGCTCCACAAGATTGCGAATAACCGTTGAAGTTGGTGTATCAGTCACTAAGGTGTATCTTTGCTGGTTGTAGTCGGGCCTTGAACGTCTCTCCATCCAAATTAAAGTTATCTCTCTTCTCTCACCAAAGATCGGCACCAAACCCTAGCGGGTTCACATCATAAAGATCTACTAGTGGCTTGTCAGGCAAACCTTGGCTTCAAGCACCGTTCTTCTTGGGAAGTTCTTGTCAGGATATTTGTCAAAACAAAACAGAAGGGCCTGCACCTAAAGGAGGAGGTCGGAGACAGAGGAATGGCCAATGGGAGCGGTTGCAGGTACAGCCAGAGCCATGGAGCTCTTGTACCTGCGTCGCGCAACTTCAAAGAGAAATGGCTGCCTGATAAGTTGAAGAAATAAAAAAGAGCTCCAAGAAAGAGTAACGTAAAAAAAAATTGAGTAATGTAATAAAAAAATACTTTAAAAAGAAATGGCTGCCTGATAAGTTAAAAAGATACAAAAAAAGAGTAACATAAGTTAACGCTAAAAGAAATAAAAAAAAAAGCTCGACAAAAGAGTAACATATAAAAAATTTATACGACGCCTGAGAGATTCGAACTCTCGCGGGGAAACCCCATGTACTTAGCAGGCACACGCCTTAACCACTCGGCCAAAGCGTCGGTTGTGGTGATTGTGCTGAAACTAATAATAACTTCGATAAGTTTCTCGTGAACCAAAGCTCCTCTCCTCTCCAACTGGGTTGTACCCCACCGCGCTAGAGCTCCGCAGACAGCCGCACCAGCCATGGACGCCGGCGGGNNNNNNNNNNNNNNNNNNNNNNNNNNNNNNNNNNNNNNNNNNNNNNNNNNNNNNNNNNNNNNNNNNNNNNNNNNNNNNNNNNNNNNNNNNNNNNNNNNNNNNNNNNNNNNNNNNNNNNNNNNNNNNNNNNNNNNNNNNNNNNNNNNNNNNNNNNNNNNNNNNNNNNNNNNNNNNNNNNNNNNNNNNNNNNNNNNNNNNNNNNNNNNNNNNNNNNNNNNNNNNNNNNNNNNNNNNNNNNNNNNNNNNNNNNNNNNNNNNNNNCGCGGGAGCCGCTGCTGCGGGGGGATCCGTAGCGGACGAGCCCCGGGACGCGCGGGTGGTGCGGGAGCTCCTGCGCTCCATGGGGCTCGGCGAGGGCGAGTACGAGCCGCGCGTGGTGCACCAGTTCCTGGACCTGGCCTACCGCTACGTTGGCGACGTGCTCGGGGATGCCCAGGTATACGCTGACCACGCCGGTAAGCCCCAGATCGACGCCGACGACGTCCGCATCGCCATCCAGGCCAAGGTCAATTTCTCCTTCTCCCAGCCGCCACCCCGCGAGGTACAAGCCTACCCAAGATCCTCCCCCTTTGTTCTTTCACGTGGAAAATTAGCTTCCGTATATGTTTTGTTCATCTTTATCTGCGCCAATAGCTCTTGCCATGGATTAGTGTCCCTATTGTGGAGTCCAAGAACCCTAGTTGTTCTGCGTGTCTGATTAGTCGGTTTTTGACATTGGTTTGATTGTTGTATCCTGTTGGACCTAGACGCCTAGAACCTAAGATTTCGCTACTTCGTCAGTACTGATAGGATTTTTCCTTGCACTCCGAAGATTTCGCTTTGCATTGTTTAGTTGGAATTGTGTATTCTTCGCCCTTAAGATGCACGCCCTTTAGATTGCCTTATATGTGTATGATGAATTGCGTTGTAAATGTCATAAGATTTTCTAAAGCTGGCATGCTCTTCTTTGTTCTAATATTCACTGTTTCTTTCCAATATCAACTATTGACTGTAGTTTAACTCTGATCATCTAAGCTGAACATGGTATCACTCATTCTGCATTTCAAATTATGATGATACAATTCCGATCCCTGATTATTAAACACAAGCGACCTTACAATGGAGTCATATTGCCTAGGATTTACCTTTTGGAAAGGGAAATGACATCTAAATGTTCAATTTTGAACGATGCATGTTATGATACATTGTCCATGCCACTAGAACGGAGGGTATAAGACTGCAAGAATAAAATTTAACAATTTAGGTGATTCTAAATGGTACAGGTGCTTTTTCTGGATTATAGAAGCTTACATAACATACTAGGCTGTTATAGTTGTGTGTTCCTACTTCTTAGCTGATGCATATCCCTTCAGCCTCCTTCTGAAGTTCTAGTAAATGTGAATTATTCATTGTTTTATACTGGATGGGTTGAATTATGTTGACTATATCTGTCCTTCAATGATATATTGTGTCGGAGTATGGCAGCCCGGTGCACGTAGCTCCTGCTTGCGCAGGGTCCGGGGAAGAGTCTGGCCACTTTGTGTATTGCGTCGGAGGTTTTTTTTCCCTCTCTCCATATTTTGCTGTGTGAATAATCGACAGTGGTTACTGTTCCATATTCACTATTTTTAGAGGACTGCTATAACTAGATTGGTGGCAACTGACTCTCAATGAGAATTCATGCCGTGTCCTTCCAACACTACGAAACATTCATTACAGGTAGAATGCAAGAATGGCGTCGGACTAATTTACATTTTTTAAAAAAATCAACTAGGGGGGAGAGACATCCACCCTAGCATTGTTCGATAGAAGAATGCTGAGAATCAAACCCTCTTCTGCTGGGTACTAATTTACATTTACATTGATTGCATTCTGAACTTCAGCCTCTTATTTATTTTTCAGAATATGATAATTGGCTTGTTAATTTGCTTCTCAAATGTACTTTAGTAACACTTGTCATATGAAGTAACCTCAAGAAGCCGGCTTTTGAAAACAAGTGCTCCATCTGACTATTCATTGACAAACAGGTGTTATATAGCTATAAAATATGAGTTTAGGTTCCTTTACTGATTGCACCTTGTATGCTTATTATTTTGTTTATTATGGCTAATAAGGAATGATTAAAATAAGGAATTCACAGTAGACCTTTAGTTTTGTGGTAAGAGTTTTCTGTTTTGAAGCATTTCTACACCAAGCCCAAGAAGTATGCATATGTTATCCAACTTGATCCCTTAGGCCCATCATAGGCTTATGCTAACAGCTTACACATGCACTGAATATGTTGAAAGTGTACGGGGTATATCTTGCTCACTCCCATACAATGTCATGTCAATTATCAGTAGGTTTCTCAGCTAGAACTCCACCAGAAAAAAAAATCTTAGGAACATTGAGGGTCACTCTACTGATAAGATCATAGGAAAATTGAGGCTGGTGCAGGTGCTTCCTGCATTTAGGCGCCTGCTCAGCCATTGGAGGAGCATAGGGGATTTGAAGGGAAAACAGAAGGTTGAAATGTAGCAAAATTAGCAATTGGGAGGGGTAAAGTGTAAAACAGTTTCTTCTGAGATGTGTCCTCCCATGTCATCAAGTGATGCTTGCTGGTTGTGTACTGTCAGTGGTTTAAAATTATGGCAATCGTTTTCTCTGTTAAGTTCATATTTTTTTAAAAAATGGGAATCTTTGTTTTAAAATGTCCTTTAATACCTTCAATTTTTGCTTTTTTTAAATGTACAATACAGTAGAAAATAGTGTCCAAAATTCCAAATATCACTTTTTCATGATTGAGTAAAGGCTATCCTTTTTAGAGACAAAAGTTGACCTTTAGGGAACAATTCCGAAAGAGTTTAACGCAGAATTGGGGCAAAAAAATTCAGCCCACAACTGAACATGCACAACCTAAAGAGTGGTTTTGCCATCCATACCAACACAACTCCAACTGAGCTTTAGTTTCTCAAAACTCTACTCAACAGACCAGTGCATCAACTCCTCTGTTAGAAATCTTCAAATGTTCCGCCAGCACATCAGAGCACCCCAAATGATATTCAGAGTAGTCTTCCATGTTTACTGTCTTCATTTCGGCTAACAGATAAGTGCAATGGATAAGCTTGTGAATGTTCTGGTGGTTCTCAAGTTTTCGTGCTCCTCCTGTTTTAATAATGGAAGGGCTAGTTAATATCTTGACTCTTTTTATTCTTAAAATTGCTTATTAAAATGTTTGATTGGATGCCTGAAAGATTTAGACCTGCATGGTAGGCACACTATGGGATAATATATTATTTTTCTCATCTTGATAGGTTCTTCTTGAGCTTGCACGTAGCCGAAACAGAATCCCCCTCGCGGGAGCCGCTGCTGCGGGGGGATCCGTAGCGGACGAGCCCCGGGACGCGCGGGTGGTGCGGGAGCTCCTGCGCTCCATGGGGCTCGGCGAGGGCGAGTACGAGCCGCGCGTGGTGCACCAGTTCCTGGACCTGGCCTACCGCTACGTTGGCGACGTGCTCGGGGATGCCCAGGTATACGCTGACCACGCCGGTAAGCCCCAGATCGACGCCGACGACGTCCGCATCGCCATCCAGGCCAAGGTCAATTTCTCCTTCTCCCAGCCGCCACCCCGCGAGGTACAAGCCTACCCAAGATCCTCCCCCTTTGTTCTTTCACGTGGAAAATTAGCTTCCGTATATGTTTTGTTCATCTTTATCTGCGCCAATAGCTCTTGCCATGGATTAGTGTCCCTATTGTGGAGTCCAAGAACCCTAGTTGTTCTGCGTGTCTGATTAGTCGGTTTTTGACATTGGTTTGATTGTTGTATCCTGTTGGACCTAGACGCCTAGAACCTAAGATTTCGCTACTTCGTCAGTACTGATAGGATTTTTCCTTGCACTCCGAAGATTTCGCTTTGCATTGTTTAGTTGGAATTGTGTATTCTTCGCCCTTAAGATGCACGCCCTTTAGATTGCCTTATATGTGTATGATGAATTGCGTTGTAAATGTCATAAGATTTTCTAAAGCTGGCATGCTCTTCTTTGTTCTAATATTCACTGTTTCTTTCCAATATCAACTATTGACTGTAGTTTAACTCTGATCATCTAAGCTGAACATGGTATCACTCATTCTGCATTTCAAATTATGATGATACAATTCCGATCCCTGATTATTAAACACAAGCGACCTTACAATGGAGTCATATTGCCTAGGATTTACCTTTTGGAAAGGGAAATGACATCTAAATGTTCAATTTTGAACGATGCATGTTATGATACATTGTCCATGCCACTAGAACGGAGGGTATAAGACTGCAAGAATAAAATTTAACAATTTAGGTGATTCTAAATGGTACAGGTGCTTTTTCTGGATTATAGAAGCTTACATAACATACTAGGCTGTTATAGTTGTGTGTTCCTACTTCTTAGCTGATGCATATCCCTTCAGCCTCCTTCTGAAGTTCTAGTAAATGTGAATTATTCATTGTTTTATACTGGATGGGTTGAATTATGTTGACTATATCTGTCCTTCAATGATATATTGTGTCGGAGTATGGCAGCCCGGTGCACGTAGCTCCTGCTTGCGCAGGGTCCGGGGAAGAGTCTGGCCACTTTGTGTATTGCGTCGGAGGTTTTTTTTCCCTCTCTCCATATTTTGCTGTGTGAATAATCGACAGTGGTTACTGTTCCATATTCACTATTTTTAGAGGACTGCTATAACTAGATTGGTGGCAACTGACTCTCAATGAGAATTCATGCCGTGTCCTTCCAACACTACGAAACATTCATTACAGGTAGAATGCAAGAATGGCGTCGGACTAATTTACATTTTTTAAAAAAATCAACTAGGGGGGAGAGACATCCACCCTAGCATTGTTCGATAGAAGAATGCTGAGAATCAAACCCTCTTCTGCTGGGTACTAATTTACATTTACATTGATTGCATTCTGAACTTCAGCCTCTTATTTATTTTTCAGAATATGATAATTGGCTTGTTAATTTGCTTCTCAAATGTACTTTAGTAACACTTGTCATATGAAGTAACCTCAAGAAGCCGGCTTTTGAAAACAAGTGCTCCATCTGACTATTCATTGACAAACAGGTGTTATATAGCTATAAAATATGAGTTTAGGTTCCTTTACTGATTGCACCTTGTATGCTTATTATTTTGTTTATTATGGCTAATAAGGAATGATTAAAATAAGGAATTCACAGTAGACCTTTAGTTTTGTGGTAAGAGTTTTCTGTTTTGAAGCATTTCTACACCAAGCCCAAGAAGTATGCATATGTTATCCAACTTGATCCCTTAGGCCCATCATAGGCTTATGCTAACAGCTTACACATGCACTGAATATGTTGAAAGTGTACGGGGTATATCTTGCTCACTCCCATACAATGTCATGTCAATTATCAGTAGGTTTCTCAGCTAGAACTCCACCAGAAAAAAAAATCTTAGGAACATTGAGGGTCACTCTACTGATAAGATCATAGGAAAATTGAGGCTGGTGCAGGTGCTTCCTGCATTTAGGCGCCTGCTCAGCCATTAGAGGAGCATAGGGGATTTGAAGGGAAAACAGAAGGTTGAAATGTAGCAAAATTAGCAATTGGGAGGGGTAAAGTGTAAAACAGTTTCTTCTGAGATGTGTCCTCCCATGTCATCAAGTGATGCTTGCTGGTTGTGTACTGTCAGTGGTTTAAAATTATGGCAATCGTTTTCTCTGTTAAGTTCATATTTTTTTAAAAAATGGGAATCTTTGTTTTAAAATGTCCTTTAATACCTTCAATTTTTGCTTTTTTTAAATGTACAATACAGTAGAAAATAGTGTCCAAAATTCCAAATATCACTTTTTCATGATTGAGTAAAGGCTATCCTTTTTAGAGACAAAAGTTGACCTTTAGGGAACAATTCCGAAAGAGTTTAACGCAGAATTGGGGCAAAAAAATTCAGCCCACAACTGAACATGCACAACCTAAAGAGTGGTTTTGCCATCCATACCAACACAACTCCAACTGAGCTTTAGTTTCTCAAAACTCTACTCAACAGACCAGTGCATCAACTCCTCTGTTAGAAATCTTCAAATGTTCCGCCAGCACATCAGAGCACCCCAAATGATATTCAGAGTAGTCTTCCATGTTTACTGTCTTCATTTCGGCTAACAGATAAGTGCAATGGATAAGCTTGTGAATGTTCTGGTGGTTCTCAAGTTTTCGTGCTCCTCCTGTTTTAATAATGGAAGGGCTAGTTAATATCTTGACTCTTTTTATTCTTAAAATTGCTTATTAAAATGTTTGATTGGATGCCTGAAAGATTTAGACCTGCATGGTAGGCACACTATGGGATAATATATTATTTTTCTCATCTTGATAGGTTCTTCTTGAGCTTGCACGTAGCCGAAACAGAATCCCCCTGCCCAAGTCGATCGCTCCTCCTGGCTCAATTCCTCTGCCGCCTGAGCAGGACACATTATTGGCCCAAAACTACCAGCTCCTACCTCCGTTGAAGCCACCGCCTCAAGTTGAGGAAACAGAAGATGAGAATGAAGAAGCAAACACGAGCCTGACACCAAACTCTGTGAATCCTAATCCAGTCTTCTCACAAGATCAGAGGGGTAACGAACAGCAGCATGCACCACAGCATGGTCAGAGGGTTTCTTTCCAACTCAACGCTGTGGCAACTGCCGCTGCAAAACGTCCACGGATGACCATGGAGCAGCTGAACATGGGCTAACCGTCCGCCGTTGGTTGCATTGTAGTATTTATTTCCTTGTCTTACAAGTCTGGCAGTGGTTGAATTGTGTTTGAACTAGAAAAGATTATTGTCATAGTGGTTTGTTCTTAATTATCAACTCTCACCCTCCGATTTAACCACGAGATTTGCAGTACAGCAATAGCATATGTTTCTGTCTGTGAGAACATCGATCTGGGAACTGAACTCTGCTGGTTGTGTATGCATGTGTTTCAGACAACCCTATCTGAGTTTACATTGCAAGACTACCGGTGTGCCCATTTTTTCTTTAGCATTAGCAACAAAATTAAAGTGAGGATTGAAGTTGAATATATTGAGTTTGGAGGCAGATCGCTGTTGAGATTGAAGTCTTCCTTTGGAACAAAGGAAAAATGAAGGAATTTTGTAGGATTCAAATCCTATAGGAAAGTTTCCTATAAGATCCTTTGAAACAAAAGACTGTGCTCTTACAAATCCTATGAAATTCCTATGGAATGCCTTCGTACATAGCAATTTTGAAAGAACTCTAACAAGAGGTCCAATCTCTTAGAAAGTTTCCTTTGAGTCTATCTCTTACTTCCAATTCCTGTATTTTTTTCTGCGGTGCATCCAAACGCTCTATAGAAAGTTTTCATGTGTTCTCAATTCCTATAGGATTGCACTTGATAGCAAACTCTAATCCTACGTTTTTCCTATTCTTATGTTTTTCAACCCTGTGTTCCAAAGAGGGCCTAAACTTATCAAACTTCGCTATCTATTGTCCTTTGATGCATGTCTTCTACCCACTGCTATTTCGGTGGCATATTTCTGTGGCAGGTCACTGGTATTACATGAACCCACACAATAATCGCTTCAAGTTTTTTCAATATAACAAAAAACATGGTGTTCTCCATTGTTTCCTTTTTAAAACTTCATGTGCTTCAGACAGCCATGAGACGATCTGCTCCATAACAGAAAACGTCAAGAACTATCTTGATCTCCACGTGAGATTCCTCTCTGCGACGAGATCGGCCTTCACTACTGACCTGCCATTTGATTTGATTCTTCTGAATTGCACGAAACACAACAGTAGATGCCACTGTGCATACATTACTGACTCGTGCAGCAAACAATCAGCCTGCTGCGCCTGCGGCTGCAGAAAAATAACTGCGAACATTGTAGCGGCTGCAGAAAAAATAACTAAAACACTGTAGCTGCTGCAAAGCAGCCAAGCGAACACGCTGAATTAGGTGAGCAAAACACGGAGAGCCTTGACTTGTAAAATGCTTATTGACACGTTTAAACCCAAATCTGCACATGATGCAAGTTGCCATGTCCTTCCAGAATTATGCATGCTCGAATATATCAAAGAAATCAACAAGGAATCGGTATGTACAAAAATGGGCACTAACAACCGCGCAGTAATTTTCTTCTTCTACAAAAGGGGCAGGAGAGAGAGGGGGGAGAAAAAAGAGAGCGGGTAAAATGGCCTCACCGCCGGAGTTCGCGGTGCGTCCGCGTCCCGATCGAGATGGCCATCCATGCACGCGAACATGAGCAATGTTTCCTGCACCGGCACTGGACACGATCAGGGGAGATAGCTGATCTCTCCGCACTGCCAGGACACTGTACGGCCGAGATCATCGGCGGCATATATCGATCAGGGGAGAATCGGGAGATCGCTGATCGATCTCAGGCGGCGCTAGCTGCAAGCCGGCCGCCACAGCGAGCAGAAGGACACCGGCTGGCCCTTCCACCTTAGCGCCACCGCGGCGCCGGACGATGCGGACATCTCCCATCCGCCGTCGTACTTCCTGAGCAGCGACTTGGCGGACTCGACCGCCTCGTCCCCGAACGGCGCCTCCTCGAACCCCTCCGCCGCCATGCGGTCCCGCCNNNNNNNNNNNNNNNNNNNNNNNNNNNNNNNNNNNNNNNNNNNNNNNNNNNNNNNNNNNNNNNNNNNNNNNNNNNNNNNNNNNNNNNNNNNNNNNNNNNNNNNNNNNNNNNNNNNNNNNNNNNNNNNNNNNNNNNNNNNNNNNNNNNNNNNNNNNNNNNNNNNNNNNNNNNNNNNNNNNNNNNNNNNNNNNNNNNNNNNNNNNNNNNNNNNNNNNNNNNNNNNNNNNNNNNNNNNNNNNNNNNNNNNNNNNNNNNNNNNNNNNNNNNNNNNNNNNNNNNNNNNNNNNNNNNNNNNNNNNNNNNNNNNNNNNNNNNNNNNNNNNNNNNNNNNNNNNNNNNNNNNNNNNNNNNNNNNNNNNNNNNNNNNNNNNNNNNNNNNNNNNNNNNNNNNNNNNNNNNNNNNNNNNNNNNNNNNNNNNNNNNNNNNNNNNNNNNNNNNNNNNNNNNNNNNNNNNNNNNNNNNNNNNNNNNNNNNNNNNNNNNNNNNNNNNNNNNNNNNNNNNNNNNNNNNNNNNNNNNNNNNNNNNNNNNNNNNNNNNNNNNNNNNNNNNNNNNNNNNNNNNNNNNNNNNNNNNNNNNNNNNNNNNNNNNNNNNNNNNNNNNNNNNNNNNNNNNNNNNNNNNNNNNNNNNNNNNNNNNNNNNNNNNNNNNNNNNNNNNNNNNNNNNNNNNNNNNNNNNNNNNNNNNNNNNNNNNNNNNNNNNNNNNNNNNNNNNNNNNNNNNNNNNNNNNNNNNNNNNNNNNNNNNNNNNNNNNNNNNNNNNNNNNNNNNNNNNNNNNNNNNNNNNNNNNNNNNNNNNNNNNNNNNNNNNNNNNNNNNNNNNNNNNNNNNNNNNNNNNNNNNNNNNNNNNNNNNNNNNNNNNNNNNNNNNNNNNNNNNNNNNNNNNNNNNNNNNNNNNNNNNNNNNNNNNNNNNNNNNNNNNNNNNNNNNNNNNNNNNNNNNNNNNNNNNNNNNNNNNNNNNNNNNNNNNNNNNNNNNNNNNNNNNNNNNNNNNNNNNNNNNNNNNNNNNNNNNNNNNNNNNNNNNNNNNNNNNNNNNNNNNNNNNNNNNNNNNNNNNNNNNNNNNNNNNNNNNNNNNNNNNNNNNNNNNNNNNNNNNNNNNNNNNNNNNNNNNNNNNNNNNNNNNNNNNNNNNNNNNNNNNNNNNNNNNNNNNNNNNNNNNNNNNNNNNNNNNNNNNNNNNNNNNNNNNNNNNNNNNNNNNNNNNNNNNNNNNNNNNNNNNNNNNNNNNNNNNNNNNNNNNNNNNNNNNNNNNNNNNNNNNNNNNNNNNNNNNNNNNNNNNNNNNNNNNNNNNNNNNNNNNNNNNNNNNNNNNNNNNNNNNNNNNNNNNNNNNNNNNNNNNNNNNNNNNNNNNNNNNNNNNNNNNNNNNNNNNNNNNNNNNNNNNNNNNNNNNNNNNNNNNNNNNNNNNNNNNNNNNNNNNNNNNNNNNNNNNNNNNNNNNNNNNNNNNNNNNNNNNNNNNNNNNNNNNNNNNNNNNNNNNNNNNNNNNNNNNNNNNNNNNNNNNNNNNNNNNNNNNNNNNNNNNNNNNNNNNNNNNNNNNNNNNNNNNNNNNNNNNNNNNNNNNNNNNNNNNNNNNNNNNNNNNNNNNNNNNNNNNNNNNNNNNNNNNNNNNNNNNNNNNNNNNNNNNNNNNNNNNNNNNNNNNNNNNNNNNNNNNNNNNNNNNNNNNNNNNNNNNNNNNNNNNNNNNNNNNNNNNNNNNNNNNNNNNNNNNNNNNNNNNNNNNNNNNNNNNNNNNNNNNNNNNNNNNNNNNNNNNNNNNNNNNNNNNNNNNNNNNNNNNNNNNNNNNNNNNNNNNNNNNNNNNNNNNNNNNNNNNNNNNNNNNNNNNNNNNNNNNNNNNNNNNNNNNNNNNNNNNNNNNNNNNNNNNNNNNNNNNNNNNNNNNNNNNNNNNNNNNNNNNNNNNNNNNNNNNNNNNNNNNNNNNNNNNNNNNNNNNNNNNNNNNNNNNNNNNNNNNNNNNNNNNNNNNNNNNNNNNNNNNNNNNNNNNNNNNNNNNNNNNNNNNNNNNNNNNNNNNNNNNNNNNNNNNNNNNNNNNNNNNNNNNNNNNNNNNNNNNNNNNNNNNNNNNNNNNNNNNNNNNNNNNNNNNNNNNNNNNNNNNNNNNNNNNNNNNNNNNNNNNNNNNNNNNNNNNNNNNNNNNNNNNNNNNNNNNNNNNNNNNNNNNNNNNNNNNNNNNNNNNNNNNNNNNNNNNNNNNNNNNNNNNNNNNNNNNNNNNNNNNNNNNNNNNNNNNNNNNNNNNNNNNNNNNNNNNNNNNNNNNNNNNNNNNNNNNNNNNNNNNNNNNNNNNNNNNNNNNNNNNNNNNNNNNNNNNNNNNNNNNNNNNNNNNNNNNNNNNNNNNNNNNNNNNNNNNNNNNNNNNNNNNNNNNNNNNNNNNNNNNNNNNNNNNNNNNNNNNNNNNNNNNNNNNNNNNNNNNNNNNNNNNNNNNNNNNNNNNNNNNNNNNNNNNNNNNNNNNNNNNNNNNNNNNNNNNNNNNNNNNNNNNNNNNNNNNNNNNNNNNNNNNNNNNNNNNNNNNNNNNNNNNNNNNNNNNNNNNNNNNNNNNNNNNNNNNNNNNNNNNNNNNNNNNNNNNNNNNNNNNNNNNNNNNNNNNNNNNNNNNNNNNNNNNNNNNNNNNNNNNNNNNNNNNNNNNNNNNNNNNNNNNNNNNNNNNNNNNNNNNNNNNNNNNNNNNNNNNNNNNNNNNNNNNNNNNNNNNNNNNNNNNNNNNNNNNNNNNNNNNNNNNNNNNNNNNNNNNNNNNNNNNNNNNNNNNNNNNNNNNNNNNNNNNNNNNNNNNNNNNNNNNNNNNNNNNNNNNNNNNNNNNNNNNNNNNNNNNNNNNNNNNNNNNNNNNNNNNNNNNNNNNNNNNNNNNNNNNNNNNNNNNNNNNNNNNNNNNNNNNNNNNNNNNNNNNNNNNNNNNNNNNNNNNNNNNNNNNNNNNNNNNNNNNNNNNNNNNNNNNNNNNNNNNNNNNNNNNNNNNNNNNNNNNNNNNNNNNNNNNNNNNNNNNNNNNNNNNNNNNNNNNNNNNNNNNNNNNNNNNNNNNNNNNNNNNNNNNNNNNNNNNNNNNNNNNNNNNNNNNNNNNNNNNNNNNNNNNNNNNNNNNNNNNNNNNNNNNNNNNNNNNNNNNNNNNNNNNNNNNNNNNNNNNNNNNNNNNNNNNNNNNNNNNNNNNNNNNNNNNNNNNNNNNNNNNNNNNNNNNNNNNNNNNNNNNNNNNNNNNNNNNNNNNNNNNNNNNNNNNNNNNNNNNNNNNNNNNNNNNNNNNNNNNNNNNNNNNNNNNNNNNNNNNNNNNNNNNNNNNNNNNNNNNNNNNNNNNNNNNNNNNNNNNNNNNNNNNNNNNNNNNNNNNNNNNNNNNNNNNNNNNNNNNNNNNNNNNNNNNNNNNNNNNNNNNNNNNNNNNNNNNNNNNNNNNNNNNNNNNNNNNNNNNNNNNNNNNNNNNNNNNNNNNNNNNNNNNNNNNNNNNNNNNNNNNNNNNNNNNNNNNNNNNNNCGCCGCCGCTCTGGTGCCCGGACGCCCTGTGCACCGGGGACTTGCGCTTCTTGCTCGACGAGTTAGGAGGCTCCGCCGACGCCGCCGGGGACGCAATGCTCGGGGAAGCGTACTGCGCCGGCGCCGCCGCCGGCGCCGCCGGGGGCGACAGCGTCTGCGCCACCACGCTGCCAATGTCGTCCTGCTGCACCGCGGGCGGGATCGTCGACCACCACCCGGCGATGTCCACGTCGGCCGTCAGGAAGGAGATGGAGTCCTCCAGCCAGTCCAGCCCGTTGGCAGCGGCGAGCTCCTGCTCGTGGCCGTTCATGGCCTTCGCGCACGCGAGCTTGCAAGACGCGGTGTGCATGGACATGCAGACACAGACTAAGCTTGCTTCTTAATTTGGCTTTATGGAGCTTAATCTAGGGTCTGGCATATTGATTGGAGCTTAAGGAGGAGCACAGGAGCAGGGGATCACAATGTTTGGTGGCGTTATTTTAGAGTCCTGCCCTCGTGTAACTGACATATTCCCTACACTTTGGAAGCATACGGCTCCCCCTCTGTGCACTACTTACACTACTCGATGGATGGCATGTCTCTCGGGATTCTGCTATGGAGATGATTAACAAACACGACCGTGAGATGGCACTCCCTCCTCTCTTAACATAAATCTATGCTTGATTGGCTGGATTGGGATGTTGAATCTCAACTTCTTGTGCTTCTGAATTTGTCCTAGCGAGATAAGCCTTGCACCAATAGCGCACAAACTGGATCGTGAATTTGTGACTGTAACTTCCTTGGCAATTGCCCGGATGATTCCTTTTCTGTTTCCTTCAGTATTGATGTTCTAGAACCCACTGCTTACAAAAAAAGTTCAACTTTTGCATAAAGCACACGCTTGGGAGACCAACACTGAATTCTCAGCCCTTGACTTTGCTGGGAGGAAACATTCAGAGCCCTTGAGTGCTGGAGCAGCTAATAATGCTGCCAATGCTTTCCCCCATTGGATGGGAACTGAAGGAGGGCGGCTGTCACGGCATTTCGGTACAGAGATGAACAGTTTGGTTGGAAGGCGACAGGAACGACGGCTACCTGTTAGTTGGTACGCCAATGTTACCTGTTGTTTGGATCCACACTAAATCTCAACTGATTGCGATCCAAATGACACCATCACAGGTGCATGTGGGCTGTTGTGGGGACAGCCTCCCCTGCACTGCTTATGCTGATCATGCCCTCAGCAATGGCTCTTTATTTAGTTCATGACGACAGGTAGGCCCAGCTCACCTCCGTCTATCAGCAGTCGACAGATGTTCGTCCGAAAATGGGAGATGCGTATTTTAGTCGGTTCTCCATGTATTCGTGTTGGTTATTGATATGTCAAGAGTACAGTGCATTTTTGAGACGTGTGTTAGTAAGCTAGAGTATGCTTTTTTTCTGAATATTGGGAATTGATGCCTGCAAAATTGCACGTCTCTTCTAATTATCTGACGCCTCTAAAAAACTCTTTTACGTCAAGCAAAGTCACAACTGTACGTTTACCCCAAAGTATAAGATTGATAGCTTCATATCTCAAACATAAATATCAGAGAGGTGGGCAACAGTTTTCTTTCTTCAAAACAGCTTCACTGCTAGTACTTATTCAAACGTCAAATTAACTACGCAGATGGTGATGGTGAAATTTGTCATCAAAGATGAACACAAGCAGTACTGCAGTCCTACTCCAATCAATCAGTCTCAATTTTTTTTAACACACTGGCTGTACGACAGCAATTTCTTTGGTGTATTACGATGGATACAATGATATACTTAATTACTGAAAGGCAGTAATTTTATCGGCAGGCATAAGTCCAATTCTCTATATTCTTATCGTCAGCTCCAAATTTCTTGAGTTTATCTAGTCCATATTCTTAGGGGAACATGCAGGCGCCACCTACAGAAATTTTTCGCGTGAAAGTATTGTTTAGTTTTATAGTGATAGTAATAATGAAGAGCTCTGTTCTGAAACAGTTGCAAAATATTCTCACAGCTTTCAGTGGCCTCAACTATTTCATTTTTAATCTCCACTAGTGGTTAAGTGGTCTGTCAGTACTCTTTCACTGGGAAACCCACTCCAGTATGCTAGGTATTGACCCCTTTTATTTCAAGTTGCTTCGCAGATTCCAGATTCAGAGAAAAATATATGATTGGATTGTGTGTGTAACTAATGTTTTTATGCAAGTGGTCCATAAATAAATGATGCTCTCCTTTTCTGATGTGGAGAAGATTGCATAAATCTCGTTCAGATTCTGACAATTGTACATGATATAGCAAATTTGGTATACAGTTATACACTTGATCAAAACTACTTGTTTGTTCTTATACTGCAGTTCCCAAAGTAACAATTACTGAACATATTGCTCCATGCAGTTAAAAAGAGCCAAGTGAGAGTGATGCCTTTGACTCCTGGGTCTTGATCATTTCAGTAGCGTTCTAAAACATGCAACTTCATGCAGGTTACTTGTGAAATCATTCAGTGCGAACTTTTTCCTTAACTTACGCCATCCTGCATTGATCTGATTTATCATCTGCTGTTTCTATGAAACCTTTTGGCTACTTATTCTCGAAAAAAAAAAACCTTTTGGCTACTTCTCCATGTTCAGATTTCATGGTAACCTGAGTCTTCGTGCTTTCCAAAAGCAACGGGTTAAAAACGGAGCTGCAGTAAGAACACAATCCACCAATGATTCGACGAATTTAGACGCAATAATGGCACCAAGATTAAACAAAGCACGATCTTGTCCACATCCTAAGCATCAAGACAACATCACACAATTAAATTAGACCACAGACAGTAACCGCCTTGCAATTTTCGTCCACGCCGCGCAATCATCTGTCTCATTGGAGCCTTGGCAGCAAGGGGACTCCACTTGAAATCCAGGACCATATCTTATGAGTTATGAGTTCTCATTCCACACACAGCTCTGAAAGAGAAACCCAAACAAAACAAATACCTTATTCTAGCCATCATGTTGCGCTGGGCGAATGCGAGGTTGGCACACAAGACATCATCTCGGTGTAGGCAAGGAACTAAACAACTCAAGATTCTTGCAGTGACGTTGCGCTGACAGGAATATACCCAATCAAATCCATTGTGCCTTCGTATTAAAAATGCAATTTGGTTTCAGAGCAGCAATATTATGTTTTCATTGACGTTGGTGATGCGGATGTGCGCATTGTTTATTGGGTGTACTACGCAAGTATCTGAAAGAGGAAAGAGCGATGGTACTTTCCCTCTCATCACTTGATCCGACTTGGTTTGAAAACTTGGAATCGCAGCATTTTGTCACTTGTGGTGACATGTAGTGAGTAAAGGGAAGTACATACAGCTTTGCAGCAAGTACATGTAGTCGGATAAGAAGTTCGTACTGCAGTAAAGCGAACAAATTAAAGCCATAGGCTTGATGGATGAACAAACTTGTTGTTGGGGTAACGAAGCCCTCCAACAGGAATTGTGCTTGTCTTTGGTTCTCGTAAAGCCCTTCGTATGCTATGCAGTGCGGAGCTTGCCGTCCGAAGCCCTCTTGAGGCTTGGTCGAGTTTCAGGTTCACTAGCGAAGCCCTCCTGAAAGATTGGTATATTCAAGATTACGTTTCTCCGTTCTTTGTACGATGCCGAAGCCCTCAATCGTCTTCGATGCTGGGCGAAGGGGTTGATGGAAAGATGAGTAGACATGGTTGAAATCAATGATGTGTCTTTGGCTCGTGGTCCGAAAAACAGATGTCATTACAAGAGCTCCTTCCTCTATATTTATAGGAGGAGGAAATACTGTTATTACATTTGTGTCATTGATCAATTCTGTTACGGCTATTCTTTATAGGCTTTAATTCGTCTATGCTTTAACATCCTTCGCTCCTTCCTTCAAACCCTTCGTTGTGTTATGTTGAACCTTCGCATGAATCATGTAGCCTTCGGCATTATCTCCAACACTTGTCATGTAGGCAGATAAACAAACAGTGCTTAATTTTCTCCATTGACATAGTATTTTTTTGGAATAATTCTACATGAGAAGTGAGTCTTACCATCATTTAGCAAACGAGGTACGGAGCATATGCCCTTAAAGAACAGATATTAATTTTATAATTTTTGACTGTAACAATTCACATTAATAAATCACCTTCTCTCGTTGCGTACACATCAGACTTGCGGAGACCAGCTGGGGCACTTTTTTTCTTGATCAGAAACATCAGTTGACATCACAAGTTAGATGGGGTCGGCGACACATGAAACCGACAGAAACCATAACAAATCATGAACGCTGATGACAGACTAGTGAAGAAGGTCAGACGATTTCTGGACGGGCGAAAGCAAAGTTAATATGTTCTAACGAGGAGCCAATGATGATTGATCATCACTTGTTGATGACTAGGTACTAGTAAAGTAAGCAAACAGTTCAGTGATTCTTCCTGCAGTGTTGCTGAATGTTGATAACCAATGTGTTTCTTGACCATCCTTGCTTTTAGTCAATGCTGGTGCACGGCCAAAAGCACGAAGACATGTCTCTGTCCGCGGGGCATCTTCCTCAAGTCACTGTCCTAAACAAAGTGGCTATGAATGATAATAGCTTAATCATGATAAGCACGACTGGATGAGCCACCAAGCTTGGTCAGCAAATGGAGAGGAGAGGCAAGTTGCGGCTTTGCCTATCTCTAGTTAGATCTTGCTATTTTACAAGACTGATGAAGTCATCTTAATTTGATTGATCCGCTCAAGTTTATTTCCTAGACGTGTTGGGGAGCATAATGTTAGGGTAGTTGGGTTTAAGGCCCTGTTCATTTTGAAGATCGTTGTAGTACTTCATTGTGCTGCCAATGCATGCTCATAGAGTCATAGACAGAGTATTGACTCGTGGCGTTGAGCTTTTCTTTTCTTCCCTTTTCATTCTTTGTTTGCAACCATGGTATTGAGCTATCCTGGGCCGTAATCAAGCCCCCCCCCCCCCCCTCCTTTTATTTGTGAGTAGGAAAAAAATTCCATTCATGTTCTGCAGTAACAGGTCATTTTAGCAAGATGGAAATAAGTAACTATGGCCAAGAGGCCCATGGGCTCACGAGTAATGACACAGTGCATACGCCCAGGTCCATCAACGGGTATTAGCGTTACACCCAGTTCACAGAACTGTTGCTATTTTCAGAAACACAGTGCATACGCATACCTTCATTTACACTCATAAACTAACACAATCACTATTATGAAATCACGCACACCGATCCTTATGAACTTCTTCTAGTTCGACACCTTCAAAAGAGAGGACTAACACATTCAGTATTGAAGAAATCACCACTAGTGTCCCTACATCGAAAAATAAATTGCTTACCCAGATGGATAGGTTTCACTGAAGAGAGTTTTACCAGCTGAGCTAATTTTACAAGTTCACAAAACCGCTACGGGTCACAAACGTCCTGATCCTGGCTCCACTAGTAATGCTTAGCCAGCACATGAAACTTGCTAGTATTGTTTTAACAGCCTACATTTGCTACCTGGACCGAACAGAAAAAAATGAAATCTCCATAAAAAAAAAAAAAAACGTACAGTTCGTCAGTTCGAACAAAAAACTTGTATACCCGGTGTAGGTGCCCCCATATCGCAGAAAACAGGAACACATTCAGAAATAAAAGCAAAGCTGCAACACCAAAAAGTCCTGAAGAATGTACACTATACACAGCAAGCACCAACATCTACACAGAAGCTGAAGCAAAGCCAATATAGAAATGCGAAACCACAGTCCGGTCGCTTATTTCTACTACCACTGATCTCATTTTATCTACAGCAGCGATACTACAAATATACAGGCAGCTCCTACTTCTCGTTGCAGGACAGGTGCTCAAAAGCGTCCTTGATCGCAGCAATGGCAGCCAGGTACTTGCTCCTGCGCTCAAGGTACATTCCCTCCCGCACCAACCTCTGTGCAGTGTCATCTAACTGAAGCATTGCCTGCTGCTCCAAACGATCATCGATGAACAATCGCGCGACAAGGAACGAGAAAGAGGAGGCAGAATGCAGCCTGCTGAGATGCACGGCCTGAAGGTCTCCCCTGAAGTTCTGGTGCAGTTTTTCTGAAAGCATGGCCACATTCTTCAGGTGCTGCTCCACTTTCTGCATCAGACTCCCCATGTCATCTTGTGGCTCTGAGCAGACCTGGTCGTTCAGTACTTGGATTCTTGCAACCACATAGGGCTTTGTCCTGATGATCTCAGTGACCCGGAAGCGGTCTTCACCAACACAGGTTAGGAAAAAACGGTCGTCGATGAGCCGCTCACACTCGATCACTTGAACAAAGCACCCCACATCTGCCATTCTGCCATTTTTGCCAGAGTAGATCACCCCAAACTTGAGGCCCTCCTGAAGGAGGGTCTGCATCATGATGCGGTACCTGAACTCAAACGCCTGAAGCTGAAGTGTTGCACCGGGGAAGATAACTGAAGGGTACAGAACGATGGGGATCTCTGTTGTCTGATCCTTCTTCGACTGAACAACCTCATCTGTGCAGCCGTGCAGATTGGCCCTGCATTTCAGGACTGAGGTACTCCTCTTTTGGTGCCTCTTTGCAGCATTTCTTGAGTCAGGTCTCAGTGGAAGTTTTGGCTTGTAAGAAGGCGAGGATGGACTGAAACTGCATATGCCTGGCCTTGCTAAATTTGATAGTACTTGCACGGTCTTGAGCGCCATATATATGTACAGACTTCCCGTGCAGTGTGATCAGCGTTGTTTCCAGAATCTGTAAACAGAAGAAAGGATGCATATTCAGTAACTTGGACAGTTGATAGGATTACACTTTTTATGCAACAATAGCACAATTCTGGGCAACCAAGACATATCAGTGCATATTCAAGTATTAAGTGCAGAGCTAAACCTGCTATTATGAACTTATCAGGTTTATAAGAAAGGAAAATATCAATACTAACAGACACAAGAAGGAACCAAAACCACTGAAAATTTCAGAACAGAATGCCTTCCACTACACTAGCAGCACATAAGTAACAACTACACTACTTATCCAGCTCAAGTTCCTGCTCATTTCAACGGCTCAGAAATGAGAAGCAAAGGTTTATGCCTCTCTCGTCCTAACTGTACATAGAGCAGGCTCTGAGCGGAGCAGAGTAAGGAACTCCGGTTAGGTGTGGTTGGACAAAGGCTTGTCCTTTGATGATTGGGACCGCGTGATAGCGCCCCAACTCGCGAAAGCAATCCAAGGGATTCATACACAGCACCCTGCGGGGAGCAGGAGATAAGCCTGCTCTGTAAACCCTCATGGCAGGAAATCCTGACAAGAGGCAACAAACAACCACTGCTGGGTTGAGTTGAGTGGGATTCTCATTCAATCTCGTCACTGATTTCAGCAGATCGGAGAAGTTCCACTCTCTGAATGAACTCTGAAACTCTGAAGGCACCGCAGAAACACACGAGAAGAAAGAGAAGAAGAAGAAGAAGAGGAAACCAGGAACAGAAAGGATAAAAGAGCAGTCTGCGATGGAAAGTTGCAGAGAACGAGTGGAAGAAATCAGGGTACGGCTGCTGTATTTACCTTTGCTCGTTGTCACCCCTAAAGTTGGAGGTCCAGCAGCTGGAACCGGAGTGGGAGCAGAAGAACCAGCGGGCGCGGCGGGTTGATTGACGACTGATGAGATCAAAGACCAGGGGGACAGAGGAATGAAGGAAGCCGAGAGATGATAGGAGCAGAGGAAAGAGATGAATACTAGATATATAGATAGATAAAGCCTACCACTTATACTACAGATTATTGACGCCCATCCGATGAACAGTACCCTTCCTTCTTCTAGACTGGATCAGATCCACGGTCGCTGACGGTGGCCCCAGCGACGCCCCTGCTCGATTCAATCGAAGGGTTGGTGTGGTGCTGCTATTGTAGCTCTGATGCTGAACAAATATCATCGTCACAGATTCGCCGTAGTGCACGAGTATTATATTAGCGTCAAGTCTCCGACTAAGATAACAACCGATGGAAAAAGGCAGATGGCAACAAAATGGCGAGGCGAAACAGGGTGCTGTCTCTGTTGGCGCCGAGGCAGGCCTATGGCTGCCTGCCTACCAGGCCGGCACCTTGTTCCGATTCGGTTGGCCCCATCCCGTTCCATAGCCTTCGTGGTCTTGTGGATAAGGAGGATGACGACCTCCGATAACCAAGGTAAATAATTAATAGGAAACGCTTTACGGGCACATTCGCTTGGGAAGGGAAAGATAAGGGGATAGCGATAGCTTATCAAACCGTTTTTATTATGTATCGAAGGCGAAATTTTGATGAACTTGTATACTATGTTCAGTAGATGGATGCATTGGTCCTTCTACTGAACAGCTCGGGCACAAATACGTTTAGCAGAAACACACGGGGAACAGGGACAAATATGGTGAGCAGAAACACACGGGGAAAAGGCACAAATATCTTGAGCAGAACACACGGAAGAAAAAGATGCAAGAAAAAGAAGAAGACAGGAGCATAAAATAGAAAGAGCAGTCGTCTGTGATGGAAAGGTGCAGATGAACATTGATGAACTGTTGTCTTCCATTTCCCGGGACTGCGAGATGCTGCTTGGACCTGCGGCCCTGCTGCCCCGTCGACTTGGGACAGTGATCGCATATCAGAATCAGAAGCACTTTTTTGGAGCCTAAAAAAATTCTAAAAGATCGCAAGTACGCAAATATCTTCACGGAGGCACACAATCACAGCTCATATGTGAATTGTTTTAGCATCATTTGCTTCGTCTCCTGCATTACGGTATCATCCTTCCACACGGCACGATAGGATCAATGTGTATCCGGATCCAGTTAATCCTCCAACACCTGTATATACTTACGCCCCGTCCAACCGTGCATCGAATGTGACCAAAGTGGTATCTTTTCTCTGTGAAGATATTTTTATGAAACTGCCTATCGAACAGGGTATAGAAAACCCTGGTTTCAAGTCAATGCTCACCCTCTACTTTGTTCGACTAGAAATTAAAGATCTAGATTTTCTCTTCTTCCTTCATACAGTCGTCATATCTCCCTCAAGGCTCCAGCTTCGTTCACCCCGTCTCTCATCAGCCAGCATCTTCTCTCGTGGCATCTCTACGGCCGAGTCCGACCCAACCACCCCTATTGCATTATGCTCTTTTGTCGCTACACCTCCCCCCGCCCCCTTTTTTTTCCCTGCGGGCTCTGATGGCCGACTGATCCGCCCACCTCCCTTGAACTTTATTCTAAGACCGACGCTCCTCACAGAGAACCGGAAAAATCCTCGAATACTAACCCCAATCCCCCGCGCATTTGGCGCCTCACCGTCCTGCCAGTACGCCCACCTTCATTTGAACTTTATTCTAAGACCGGCACCCATGAAGAACTCAAAAAATTCTCCAACCCTAATATAAATAACCCTTCGCCAACCCCCCCCTCCGCCCCCCCCCCCCCCCCCCCCCTCATCCTCTGTACAACCCCAGTCTGCGTGCATATGGCTTCCGCACGTCGCCGCCCTACCGGTCCGCTCACCTAACCCCGACACCCATAGAACCCCCGAAGAGAACCCCGAACCCTAACCCTTTGTCATATTAACCTCATCGAAGAACCGGCTTCGCACCATGGACTAAGCTAGAGAATTAGGAGGCACTTTTATTGAGGTGAATGAAGAATAACCTACGATTTGTGTTTAATGAAGTGGTGTCCAAAATAATGTAGAACAATTTTAACATGTCTTCGCAAGTTTCTCAAGTCCTTTTTCGACAATGTCAGTGACTCTGGTGACCTCCACCTGTCAACTTGTCACAATCGCCCTGTTGCAGCAGTTATTTTGCAGCAGCTACAATGTTTCAGTTATTTCTTCTGTAGCCGCTACAGTGTTTCTGCAGCAGCTACAGTGTTCGCAGTTATTTTTCTGCAGCCTGCCGAACAGCCTCAATGATGGCAACATACACGATTACAATTTTCTTAAATGCAGAATAAAAAATTATATCAGTTTGAGAAAATGAACTTCGTCTAGATATACAAAACCCGAAAATTACCAATCCCGCAGATAAAGCCGAAAGCCTTATTTTTCTTGATTTTCTTTGGGCAAAAGGTTTCAGATTTAAATTGCAACTTAGTGAATCAATATATGAACCATCACTTCAAATCAAAATTTTCGACCTGTCACCTAGTGGTTAGAGTTCGGGCACAAATATCTTGATCAGAAGCAGACGGGAAAAAAGAGATGCGAGAAGAAAAGCCAGTAGCATAAAAAAAAAAAGCGCAGCCTGTGATGGAATGTAGAAGCGCGGAGAAAGAATATATGGAAGGAAGAAACGAAAGCATCACTACAGCGCGCGTGGTTTCAGGGAACTGCCGTGTTACTTTTGCTCTTTCTCTTTTTCGGGATTGTGAGATGCTTGCGGCTGCATGGAGGGAGCAGCACGGCCCAACACCACGTCTGAGCGCGCACAGCGCATGACACGGCGCTCTCCACCGGCGGGAGGGCTCNNNNNNNNNNNNNNNNNNNNNNNNNNNNNNNNNNNNNNNNNNNNNNNNNNNNNNNNNNNNNNNNNNNNNNNNNNNNNNNNNNNNNNNNNNNNNNNNNNNNNNNNNNNNNNNNNNNNNNNNNNNNNNNNNNNNNNNNNNNNNNNNNNNNNNNNNNNNNNNNNNNNNNNNNNNNNNNNNNNNNNNNNNNNNNNNNNNNNNNNNNNNNNNNNNNNNNNNNNNNNNNNNNNNNNNNNNNNNNNNNNNNNNNNNNNNNNNNNNNNNNNNNNNNNNNNNNNNNNNNNNNNNNNNNNNNNNNNNNNNNNNNNNNNNNNNNNNNNNNNNNNNNNNGCGCATGACACGGCGCTCTCCACCGGCGGCTGCTGCCCCAGCAACTTGGGACAGCGACCGCTTCTCAGAATCAGAAGCACTTTTTCCAGCTTCAAATCCTAGAAGATTGCACGTACACAAATATCGTCACAGTGGCTCAGACAGAGACATCGGGTCACACAATCACAGCTCCTCTGTGCATTTGTGTTCTAGCATCATTGTTTCTTTTGTTTCATCTCCTTGGTAAAATCCACACGCCAATTCTCTCTGTACCAATGCTTAAATTAGTCATAGTATAGGACTCCCCTACTGTTCGCCTAAACAAATAAGAGCTTAAATTAGTCATGAGTTATCTCCTCCGATTAATCAGTTATGAATTGATCTGCTTTGATTAATTCACAACTCTGACTCAGCTTCCGAAATAAAACCTATATATGAGGAAACAAAGTGTCTCTATATGTACCAGATGAACTATTAAGGTTATTAAAAGATACCGTTCCACGAAAAGTACCGATTGAAAGATAAAGCCAAAAGCCTGATTTTTCAAGATCTTCTTTGGACAAAATGTTTCAAATTAAAATTGCAACTTTGTGAACGAATAAATCGGCAATTCAAATCAAGACAGTTCAACCTGGTGGTTAGAGTTTGTGGCATTTTGGGTCTGTTGCTCGCCGCTGCAATAACCTTATCTGTAGGGCCCACAGGTAAGCCGGTTATGGCAGGAATGGGAATATCGCTTGGCGAACGAAGCTGAATTCCGTATTTCTGGCATGTGCGCTAATTGCCAACTCTGACAGTACTGTTGTTGCAACCACTTCAATCGTTAGCATGCCAATGGGACCCTGTAGATATTGTTCATGCACATAAAAGCACATATCGATTCATAGGTCAGAGAAAAATTTTATGGTGGGCTATAGTAAGAGTTTTCAAAAAAATAAGTATTTTGAAATGTTATCAGATATGCAATGGAAATGTTTCAAAAAAGAAACTAGTGCATAGGACAAGATTTCATAGGCGACAAGTTCACTTGTAATACCACATAATGTTCTCGCAATACCATAAAAAAATTGAGTTATTTACTTTTCTTCTAAAGATGTAACTAAGTTCTTTATAGATCCGTAAGGGAGAAATGGGACATAATGGGAGCACAGGCCCCTGGACTGTAGACAAACTTGAAAAAATGTGGACTTTCTACCTAATGGCGAAGCTGGCGCCAACAACATTTGAATTATCCTTTATAAAATCATTGTTCACTCATTGTTGGTGAACTGGGGGTATCCTTCGTGAAAAAGTAGCTCAGAGCATGAGGCAAAGGCCGAATCAGGAAATGAGGAACACAACCCAACGCAATACGACGTCCGGAATCGAGAGTGGAAGGGCAGATGCAACATGTAGATCCGAGATAAGTGCCACATAGCCTGAAATGTTGTGGGGATCTCTAGCCTCCTGCAATTGAAGCAAAGATTTTTTTTTGCGAGGATGAATATATATTATTTGAAAGTTTGGAGAAAAGTTTCTGGTTATTAGGATGGTCCTTAATGTACCTGCATGGTTGATTGATCATTGCATACAACTATATATCCATGTACGCATGTCGTGCATTCTTAGTAGACAGTCTCAGCGCTTATACATTATTTGAAGACATTTGTTTTTATGAACATTATTTATGAAATTTTGTTTTAAGATGTCTGTGTCCTTGTCAAATCGCCTGTTTCGTGGTTTCACTTTTCGGTCTCTCCGACATCTACGGGCATTAAGAAGCACCAAGGCATTCCCTTCAAATTTTTTTTTTAAAGTAGCTCCAAGGGAGCACACAGCAGAGGTAGATATACGGATCAATTAACACTCCATTTTCCACGGAACAATTTGAGATCAACGATTGCTACAGAGGGCATCTCAGATTCTCAGTACTCAATCACTAACCTACTCCCAGAACGGAAAATTGATCAAATTCTAAAGAGAAGGGAAATAAGGAAAATCCTCAATTAGCCTCATCAATCTTGAATCAGAGCGGTGTACGGTTCCACAGGCTCTGGGTTAGCACCCAAGCATTATGGGCTCAATCAACTCGCCAAGAGGAGCCAGCTCTGACCTGTCGATCAGCCGGAATTCCTGCAACACAGAAATTTTATTACACTTGAGTCAGCTGCCAGCATATGACGAAACATACAGTGGCTGCTTTGCAGCGGCTGTTTTGCAGCAGCTACAGTGTTTCAGTTATTTCTTCTGTTGCCGCAGCTGCAGCCTGCCGAACAGACCGAATACTGGGTATGCTGCGAGCAGTCAATGGATTTACTCATTGTCCTGCTAATAATACATTAATAGTCCGCTAATCAGGAAGAATGAACATTTGCTTATGAGCTGTCAGGCACTTCTTTCAGTGGAGCCTCGACAACATATGTCATCAGTGGATTAGAAGAAAAGCATTCGAGTATGTGTGTAGTGTGCCATCCAGACAAATCCATATTTTTCGTCAAGTGCGGGGTACATTGAAAAAACAAGCATGTCTTTGCTTGCAGCATGATGTATCACAACTTCTAAAGCAGTAAGCTGAATCAAAAGGGCAGAGATATTTTTTGTGCTGCATGCTGTTCTAACTTCTAGTGGCAAACAGAAAAGTATCTGATATGCTGGCAAGCTGATGTACTGAACCATTTATTTGAATGTGCTATTATTTCGTAACAATTAGTTTATAAATGCAACTAGAGAATTCTAGCAAGTGTCAATTTTCTTTAAATCGAGAATTACCAATGTGAACATCGTGAAGTGCTTGAAGCAAGTGTTGAGATGTGCTTCTTCCTTGAGCTTCACAATCATCTGGAAGTGCGAGTGGTATATGTGAGCATATACTCTGAAGAGGCGCTTGAAGATGGTCTTGACGACATCACGGAAGTTTGGAGGGAAAGGAGTCCCTGTTGGCATCCCAATTTTGAACCAAAACACATGATCAGAGAAAATGAACAAAAACCAAACTGTAGTTTTCAGTTTTTCACTCTGGGACGAGGGACAAATTACCAAGCTTTTGAGGGAATATGGATTCATCGTCGAGCTGGGCTTCTATCCAGTCCATCAGGTACTCCACATACTTGGGCGCAGAGACCTCAATAGGCCTCTTGATCTTGACTCCATCAGCCCATCTGTACTCATACCTGTGGAGGTTTCATTTTCATCAGACAGTTTGCAACGTAACACCATTTTTAAATGGAAGTTCTTTATAATACTATGTGTTGGACTGTATTTGGGGTTATCAAGAAAGATACTGTACTTTGGTCCGGCTGACATGGTCGGGCAGGTAGCTGGCGTGCAGAACTCCATCAGGGTGCCGTACAGAATGTTCACTTGATTGAAGAAGTCAACAGCTGCCATCACAGATGCAAGTTAACACACTACTACAAACTGCTACCGACTGAAGAGCCTAACAAGAACAGTTCATCAAAGTGCTGACAGAGAACATGACATCAAAGACTCGTTCCTTCTATTTCAACTGAGTCTGAAGTCTATGGTGTAGATTAATTAAGAACTGGAAGGATCAATAGAACTTACTGTTGACAGCAAGCCACTCGTTGAGATCCTCCCCAATCGGCAGGCGGACCGCCCCTCGNNNNNNNNNNNNNNNNNNNNNNNNNNNNNNNNNNNNNNNNNNNNNNNNNNNNNNNNNNNNNNNNNNNNNNNNNNNNNNNNNNNNNNNNNNNNNNNNNNNNNNNNNNNNNNNNNNNNNNNNNNNNNNNNNNNNNNNNNNNNNNNNNNNNNNNNNNNNNNNNNNNNNNNNNNNNNNNNNNNNNNNNNNNNNNNNNNNNNNNNNNNNNNNNNNNNNNNNNNNNNNNNNNNNNNNNNNNNNNNNNNNNNNNNNNNNNNNNNNNNNNNNNNNNNNNNNNNNNNNNNNNNNNNNNNNNNNNNNNNNNNNNNNNNNNNNNNNNNNNNNNNNNNNNNNNNNNNNNNNNNNNNNNNNNNNNNNNNNNNNNNNNNNNNNNNNNNNNNNNNNNNNNNNNNNNNNNNNNNNNNNNNNNNNNNNNNNNNNNNNNNNNNNNNNNNNNNNNNNNNNNNNNNNNNNNNNNNNNNNNNNNNNNNNNNNNNNNNNNNNNNNNNNNNNNNNNNNNNNNNNNNNNNNNNNNNNNNNNNNNNNNNNNNNNNNNNNNNNNNNNNNNNNNNNNNNNCTGTTTTGCAGCAGCTACAGTGTTTCAGTTATTTCTTCTGTTGCCGCAGCTGCAGCCTGCCGAACAGACCGAATACTGGGTATGCTGCGAGCAGTCAATGGATTTACTCATTGTCCTGCTAATAATACATTAATAGTCCGCTAATCAGGAAGAATGAACATTTGCTTATGAGCTGTCAGGCACTTCTTTCAGTGGAGCCTCGACAACATATGTCATCAGTGGATTAGAAGAAAAGCATTCGAGTATGTGTGTAGTGTGCCATCCAGACAAATCCATATTTTTCGTCAAGTGCGGGGTACATTGAAAAAACAAGCATGTCTTTGCTTGCAGCATGATGTATCACAACTTCTAAAGCAGTAAGCTGAATCAAAAGGGCAGAGATATTTTTTGTGCTGCATGCTGTTCTAACTTCTAGTGGCAAACAGAAAAGTATCTGATATGCTGGCAAGCTGATGTACTGAACCATTTATTTGAATGTGCTATTATTTCGTAACAATTAGTTTATAAATGCAACTAGAGAATTCTAGCAAGTGTCAATTTTCTTTAAATCGAGAATTACCAATGTGAACATCGTGAAGTGCTTGAAGCAAGTGTTGAGATGTGCTTCTTCCTTGAGCTTCACAATCATCTGGAAGTGCGAGTGGTATATGTGAGCATATACTCTGAAGAGGCGCTTGAAGATGGTCTTGACGACATCACGGAAGTTTGGAGGGAAAGGAGTCCCTGTTGGCATCCCAATTTTGAACCAAAACACATGATCAGAGAAAATGAACAAAAACCAAACTGTAGTTTTCAGTTTTTCACTCTGGGACGAGGGACAAATTACCAAGCTTTTGAGGGAATATGGATTCATCGTCGAGCTGGGCTTCTATCCAGTCCATCAGGTACTCCACATACTTGGGCGCAGAGACCTCAATAGGCCTCTTGATCTTGACTCCATCAGCCCATCTGTACTCATACCTGTGGAGGTTTCATTTTCATCAGACAGTTTGCAACGTAACACCATTTTTAAATGGAAGTTCTTTATAATACTATGTGTTGGACTGTATTTGGGGTTATCAAGAAAGATACTGTACTTTGGTCCGGCTGACATGGTCGGGCAGGTAGCTGGCGTGCAGAACTCCATCAGGGTGCCGTACAGAATGTTCACTTGATTGAAGAAGTCAACAGCTGCCATCACAGATGCAAGTTAACACACTACTACAAACTGCTACCGACTGAAGAGCCTAACAAGAACAGTTCATCAAAGTGCTGACAGAGAACATGACATCAAAGACTCGTTCCTTCTATTTCAACTGAGTCTGAAGTCTGAACATGGTGTAGACTAATTCAGAACTGGAAGGATCAATACTAGAACTTACTGTTGACAGCGAGCCACTCGTTGAGATCCTCCCCAATCGGCAGACGGACCGCCTCTCGCAGGTTGCCGCTGCCGAGAGTGGCGTCGATGTGCCTTTTCAACTGCATGCCCTGAAATTATAGCCAAACAGTCACAAACTTTTCAGCTTCAGAATTAAACGTGCAGATACATGTAGTGGCATGATGCAGCTACGTTCCAGAGACTTCTTCTTATTGCTGGAAACGGACAGCCTGAATTTGAGCCCAAAGTAATCCTACTTTTTCTAGCTGTATCCGATTTAGCCACCGTAAACAGCCGGTTTACAGAGAGTTTCGGCGTTGCAAGAAAGCTATAGCTAAATGCCGACAAGGAAAACGGCTGTGACGAAAGGGAAGGACGAAAAAGGTGCACGGCTTTAGTTCCAGGTACAGGCCATTTGGAGGCAAACGGACGAAAAAAAATCGAGATAAACCGCAGTGGAAACACCCATCTCCAACTGAATTTGTCAAGGATATGAACAGCGTTCAAACAGCTTTTCTCTGAACGGAAGAAGCAGGTCCTGTAATCCAAAACCTAGAGCAGCTGACGACATTAGCAGGCAGGGAAGAACTCCGAAATTGGCGACATCCTAGATGACCAAAACTGGATGCATTTTAATTCGTAGCGCAGATTCAGGGGAACGCAGTCGAGCGTGCAAGTGCGAACTCGTTATCAGGCACAAAAAGTTCAATCTACTCGGCCGCGACTGGAAGAATTTGGTGCGTACGTACCTTGTTCCCTGACGTCGCGCTCTTCTTGGGCCTGAAGGTCCTCTGCTTTCTGCAAGAACAACCAGTCGACGAAACGCCATGAGATCACGATGGAACGAACCGGAACAGAGCTAGCGTATATCGATTCAAGAAAACATGGAGAATTCTCTTCTTCTAAAAGAGAAGAGAGCACTGAACAAACACAAAACACGATGGGTCGTCTCCTCACCTGCTTCCAAGGCCAAAGAAGCTCATGTTCTTCTTCTGCTTCTCCGGCAGTTCAGGACTTGCTGAAAAAGAATTAAGCGACGGCGACCGGGGTCGAGGAAGAAGAGGAGATGACGGAAAGGTTTCGGAGGAGGGCCGTAATAAATCCTCCGTAGGATTGATGATTCTGAGCCCTCCTGACGCTGACAGAGAGAGTATATATATGGCCTCGATCCCCAGGTGTGACGTACTTACTACTCGGATATGGACATCATCTCAGCAGAGAGAGAAGTTGAGAAAGAGAGAGAGAGAGAGATAAAGAGAGCCAGGCTGGCGGTGACGGAGAGCGGCAACGGGCTGGCAAGCAGAAACGAAACCGGCGGGCGCCAACCTCACGCCCGGCTGTCAGGCAGCGTTTTCCGTTATTCCGTTTCACCGCGCGGTGTTGCGTGCACACTGTCGTGGTCACGGCACGGCTGGCTGTTATCCGCTCAGGACCCGGCTTACTATGACCCCGTTGATGCTTTCCTCCTCTTCCAAGCCGGCTTTGGTGGGTCTTCAACTCCCAGATTGATGCCTGCTGCCTGGCTGATGATCAGCAACAGCTATCTCGCCTTTCCAACCATCAATTGATAATAATCTAATTAAGCCTTGGCGCTCGGCTGTCTATCAAGTCCATGTTAATGTCTCTCTGTTTTTATGATACACGTTTGTTTTCAACTAACGTGTATCATATATTACTATACTAGTTTTATTATACACTATTGAGTCGAGTTCAGTTTGGACGTTGGGTTATGGATCTTAGGATTTTGGGGAGGGAGGTTTCCCAAGTCCGGTATGCTCTTGAAGTACGCTAGTCAGATGGTGAAGGAGCTGAGGCAGGGCAACAAGAACACCAAGATACATTGGTAGACAAGGAAAGCTAGATTGCAACATAAATTTGGTAGCTTTGACTACATAAATCAAAAAAAAATGTAGTATTTGGGTCTATTCAGTTGCAAATGGAGTAGATCAAGCGCTAACAGAAATAGTATTGAGAACAGCAAGCATGTTTTTTTTTACTATGGACTAAAAGTAACAAGTTAACGCAAGCATACATTAGGATAAGATGATTTCATGCATCAGAGGACAAAAGAAACGTCACCTAAAACAGAAGAATGTTGACGCGATTCCTGACAAGAAAGACCAAAAGAAAAGAGGAAGAAGTAAACGAGGTAAATGACATTTCGGTACTCTCACACGTCAAGAAAAGAAATGACACTACCAAAACGTGTGAGAAATACAACGGTCAATTTCCGACAAGAAAGATCCAAGTTTGTAGGGAGGATTGAAGGAGGTAACCGGCATTTATTTTGTAGTTATGCACATCAAGAGAAGAGTATCCAAAAACAGAAGAATGTCAGCTCAATTCTTGACACGAAAGACCAAAAAGAAAAAAAAAAGGAAAGGGTAAAGGAGGTAAACGACATTTTCATACTTTCACGCGTCAAGACAAATCCAAAAATATAAGAATCGCAATGGTCACTTTACGACAAGAAAGATCCAAGCATGTAAGGAGGATTGAAGGAGGTAACCGACATTTATTTGGTAGTTATACAGATCAAAAGACGACTATCTAAAAATGGAAGAATGTCGCCGCAATTCCTGACAAGAAAGATCCAAAGTCAAAGAAAGGGGTAAAGAAGGTAAACGACATTTTGGTCAAGACAAAAAAAAAGACTACAACAAAAATTTGCGACAAACCCACAATGGAACAACACTCAAAAACATAAGAATCACAGTGGTCAATTTGAGATAAGAAAGATCCAAGCATGGAGGGAGGATTGAAGGACGAGGTAACCGGCATTTATTATGTTGCCAGCAATTTCTAGACCCAAAAAATAGAGATAAGGGTAAAGTAGATAAGCGACATTTTGGTCCTTTCACACGTCAATACAAAATACAAAGACACCATCCTAGAACACGAGAATCACATTGGTTAATTCTCGAGGAGGAAGATCAAAGCATGTAGGAGGGGCTGCGTAGATAACCGACATTTATTCGGTGTGTATGTGCTTACCAGAAAAAAAGCACGTTAGAATCATAAAGGTGAAAAGGCATTCTGATATATTGGACCAACCATGTCATATTATGATATGTTACAATGAAATGCATCCTCTTATCTAATATTACGCTTCCAAGTCAAATATTTCTACCTTTGACCACAATCTAAAGTTATATATATAGTTCAACAATATAAGCTTTCATGATATGTAATCCACATGTTGTTAAAAAAAATAGAAATGTTTGACTTTGATCAAAACTATAATGACATTCATATCTTGGAATGGAGGGAGTATAACATACCCAAACATTTTTTTAAGTATAATAGAGCCAGTTTGCCAACCCATCTCATTGGTCGATGGAGCCAAATAAGGATCGAGATAGAGGCGTGCGTGTGGCTGCACGTTGCCGACGTCTCATGAGGGTGCAAATATTCATCCCTAAATACCTTGGCACCCCTTGCTTACAAATATTTCATGGGAGAGCTGCAAGTACAGTTGATTGAATTAGCTGGTCCAACGAGTTTGGACCAACGAAAGTATTCCACATGTAAAGAGAAAGAAATCAAATTTGACATTAACCATCATGATCCTTCCCGTACCCAACAATTGTGACACGAAATTGTAGTCGCTGAACACTCACCATTAAAGACGCCACCTCCTCCCTAGTGCTGAACGTCCATTTCAATGCCACCTCCTCCCTAGTGCTGAAAATCCAGTACGCACAGACATATTACTTATACTGTAAAATACTTGTGAGGATTGGGCATAGGGGCATGTGCCCCAAGTGATGTAAATAGCCTGACATCTCTCCAATTGTGATGAAACGAATCAAATTTAACATCAATTTAATTTGACCCAGTATGCTCAAATGTCTCAGTAATTTTCAGATGTATTAAAAACTTTTCAAACCACCAATTTTAAAGCATCTTACAACAAATTTAGGATTTAGTTCAACATCAACCGTAACATGTAATGATTCACCAGACTACAGATAATTTGGAGGAACTATCGCAAACATACATGTTTAAGGAGTTGAATATATGCAAAAGTATTTTGTGTATCATTTTATATTAAGCTGCAGAATTAAGAAATGAAATTTCAAAACTGTAATTTTTTTAGGCGTTAAAACTACAGTTTTTGCGACCAATTTATTCTATTTGATGTGTTAATTCTATTGAGTTGCTTATCATTAGTAATCATGTGGTGTAAGTATAAAAAAAGGCAGATATAATTTATTTGCGTATGGGTAGATTAGTACAAGAAAACGCGCATCTAGCTGCAGGTTGGGACGGCACCGGTGCCTTGATGCTAATTATAGTTTCCATCTTGGATACTGTTAATGGCGAGGACCGACTTATCTAGGATTCGAATGGCGATTATCAAGCTGCCGTGACCTTGCTGGTGTATAAGTTTCTTCTCTTTAACGTTTGCTCTGAAAAAGAAAGTTTGTAACTAATAAAAAAAGAAGGAACTGTTGCTGCATCCTCTGTCACTGAATATTCTCTCCAATGGTTTCTATATTTTTATTAAAAAAGCTTGTCGCTTAAATTTCCAAAAATTAAACCATTTGGTGTGGGTCCCACTTAATCCATGTGTGGTCCCTCGCTCATCTCCATCAAATCGGTGGAGAAGCTTCCTCTCTTCTCTCCTTCTCTCGACTCTTCCGCTCCTGCTACAGTAGCCCGCTCGAGCGCTCCATTTCTCCGGCCAAATCCGGCCCCTCCCCTCCTTCTCCGGCTTGCTGCCGGTGTGGGTTTGGGTGAGGGACCCATCCTGTACAGGTTAGGTTGTTTTGCCTTTCAGTTTTAGGTGTAGTTGGAGTCTCTTCTCTAGCAAGAGTCTTTCGCCTTCACTCTGAAGGTGGTTTGTATCTCCGTGTTGGTAGTTCTGTGCTCAAGTAGCTTGTCCCTAGTGGTTGTTGCCGTGATGGCCGATTTCCCCGTCGGCTTCTTCCCCGGCGGGCGCTTCTTTCTGGTTTTCTTCTTCTCCATTGCTCAGCTGCAGGCTTCATGGAGGCTTGGTGATTGCCGGTGCTCTGCTTGTGTGGAGCTGGTTGATGGATGCTGTGGTTCTCTTGATAGGGTCGATCGCAGTTGTTTGTTGATCCCCGAATTCATCTGGAAAGCCTCCAAGTATTTTGTGTATCATTTTATATTAAGCTGCAGAATTAAGAAATGAAATTTCAAAACTGTAATTTTTTTAGGCGTTAAAACTACAGTTTTTGCGACCAATTTATTCTATTTGATGTGTTAATTCTATTGAGTTGCTTATCATTAGTAATCATGTGGTGTAAGTATAAAAAAAGGCAGATATAATTTATTTGCGTATGGGTAGATTAGTACAAGAAAACGCGCATCTAGCTGCAGGTTGGGACGGCACCGGTGCCTTGATGCTAATTATAGTTTCCATCTTGGATACTGTTAATGGCGAGGACCGACTTATCTAGGATTCGAATGGCGATTATCAAGCTGCCGTGACCTTGCTGGTGTATAAGTTTCTTCTCTTTAACGTTTGCTCTGAAAAAGAAAGTTTGTAACTAATAAAAAAAGAAGGAACTGTTGCTGCATCCTCTGTCACTGAATATTCTCTCCAATGGTTTCTATATTTTTATTAAAAAAGCTTGTCGCTTAAATTTCCAAAAATTAAACCATTTGGTGTGGGTCCCACTTAATCCATGTGTGGTCCCTCGCTCATCTCCATCAAATCGGTGGAGAAGCTTCCTCTCTTCTCTCCTTCTCTCGACTCTTCCGCTCCTGCTACAGTAGCCCGCTCGAGCGCTCCATTTCTCCGGCCAAATCCGGCCCCTCCCCTCCTTCTCCGGCTTGCTGCCGGTGTGGGTTTGGGTGAGGGACCCATCCTGTACAGGTTAGGTTGTTTTGCCTTTCAGTTTTAGGTGTAGTTGGAGTCTCTTCTCTAGCAAGAGTCTTTCGCCTTCACTCTGAAGGTGGTTTGTATCTCCGTGTTGGTAGTTCTGTGCTCAAGTAGCTTGTCCCTAGTGGTTGTTGCCGTGATGGCCGATTTCCCCGTCGGCTTCTTCCCCGGCGGGCGCTTCTTTCTGGTTTTCTTCTTCTCCATTGCTCAGCTGCAGGCTTCATGGAGGCTTGGTGATTGCCGGTGCTCTGCTTGTGTGGAGCTGGTTGATGGATGCTGTGGTTCTCTTGATAGGGTCGATCGCAGTTGTTTGTTGATCCCCGAATTCATCTGGAAAGCCTCCATTTGGTCAAGCCCGGTGTTGCTCATTCAAGATCTACGGCTTGGTCCTTCTCAACCTCTTTCAATGGTGCTGGTCTTGAGGCGACTGTCAGAGGGGCCGCTGGCGAGTCAATCATCCAGCATTCTGAGGCTTCTTTCCCATCTATGTTGCGGTGTATTCTTCCTCCTTATGCACCGTCCGGTCCCCGCGTGGATGGAGGCATCCTCGACTAGCGAAGTGCTACAGGCCTGGTTGAGCCATTCTCTGAAATTTCTGCGGCGATGCAGGCTTCTCCGGCGGCATCGATCTGAAGTTGCTAGTTTGGACCTTGATGTAATCTTTATTTTCATGTATGTCCTTTTGATGCTGAAAGACCTTAAGTTAGCTTTAGAATCGTGTAGGGGTCTTTCTGCAAATGTATTGATACTTCTGGAGTAATGGAGATCTTGATGCCCTGCTGGGCGTTCCGCCTTCACAAAAAAAAAAATCGGTGGAGAAAACATGGGCTTTGATATGTTGACAAATTTTCTTATAAAAACACAAGCAAGTTAAGTGTAAATGAAACCATGCCTACAAACGAAACGCACCATTCTGAAAAATATTGTGGCATGGCTTCTTCTTAATTGCAATTTCGGCCTCTGACTACACTCTCAACTGATCAGTTGCACTGAGAAAATATAATGAGACCTTAAGGGGCAAAAACAATCATAATTTGCGAGCCAAACACTTTTGCCTTCCTAAAAATGGTATATCTACAACGCAATCTTGTCAAATTATGATCAATGATTGTACTAACCACTTTAAAACCAAACTTATAAATCTAAATAGAAAAATGGAGTGGTGACAATGTCACCTCATGGTACTCACTCTGTTGCTTTTCTCGAAAGAACGGTCATAAACAAAGTTTACAAATAATTGGCTTCATTGTGCAGCAGGCGCAATATATGCTTGGGGTACATATCTGCGCTTTCTGATGATTGGAACCGGTTTCTTCAAGCTGTCACCAAAATGGTCATGCGTGTATCTTTCTCTGAAGCCAGTGATTTGAAAGGAACAGACTGATATTTCTTCTTAATATAATTTTTTTTTGCGAGTGAATATAAAACTTTGTTGAGTACCAATTATGGACACTGGACCGTGAAATTGAAATTTATGTAGCTTAGTTCAGGTCCGATGCAGCATATGAATTTGAGTCTGAGATATTGGGCAAGGATAAAAATTTGGTGGCGCAACTAATATCAAAAGACAAACTAGTTGTTCGTTCGAAAGAATAAGGCGGTCCAGATACGAAAACCACGACAGATTTATGAACTGGATGTTTTCTTTAGCTAATATTATTTTCAGAATAGCTCACAATCAGGTTGATCATTATGATGATAATGGCATGTAGTACGTCGAGCCCGCTAATTTAGCAAGTCATGCGAGACGGCTAGCTTATACCACATCCTTCACGTGATATGATTGGGGAAACGCAGAGAGAATCGGCATTATTGTTACTCGAAAGAAACGGGATTGGGGAATGATGGTGTTGCCTGACGGACCAGAATAGACCAAATCCGCCTAGCTGGATGGCAGTTGTGTGATGTTCTTGTTGACCTGCTGCTGCCTTTTGCTCTGGGTCCCTGGCAGTTTTTCGAGTTTCCTTTTCCCCTTTTTCGCCTAGCGGTTTAAGTGGCGGTAGTTAGGCTAAACTCCTCCTGGGCTGGGCTACCATTTATCTTGTTGTGCCTAAAAGGGCCTAAATTATGTCCTGACGCCCATCCTACATAGCCCATAAGATGTGTAGCCGTGGCCTAAAAAAAAGGCCGTGGGCAGAAAACTTGAAGAGTTACAATTGGGCTCGGCCCATCTAACGGCGCCAACTGAGCCCATAACATCCCTCCCACGGCTTCCACCATCTTCTCTGAAGCCAGTGATTTGAAAGGAACAGACTGATATTTCTTCTTAATATAATTTTTTTTTGCGAGTGAATATAAAACTTTGTTGAGTACCAATTATGGACACTGGACCGTGAAATTGAAATTTATGTAGCTTAGTTCAGGTCCGATGCAGCATATGAATTTGAGTCTGAGATATTGGGCAAGGATAAAAATTTGGTGGCGCAACTAATATCAAAAGACAAACTAGTTGTTCGTTCGAAAGAATAAGGCGGTCCAGATACGAAAACCACGACAGATTTATGAACTGGATGTTTTCTTTAGCTAATATTATTTTCAGAATAGCTCACAATCAGGTTGATCATTATGATGATAATGGCATGTAGTACGTCGAGCCCGCTAATTTAGCAAGTCATGCGAGACGGCTAGCTTATACCACATCCTTCACGTGATATGATTGGGGAAACGCAGAGAGAATCGGCATTATTGTTACTCGAAAGAAACGGGATTGGGGAATGATGGTGTTGCCTGACGGACCAGAATAGACCAAATCCGCCTAGCTGGATGGCAGTTGTGTGATGTTCTTGTTGACCTGCTGCTGCCTTTTGCTCTGGGTCCCTGGCAGTTTTTCGAGTTTCCTTTTCCCCTTTTTCGCCTAGCGGTTTAAGTGGCGGTAGTTAGGCTAAACTCCTCCTGGGCTGGGCTACCATTTATCTTGTTGTGCCTAAAAGGGCCTAAATTATGTCCTGACGCCCATCCTACATAGCCCATAAGATGTGTAGCCGTGGCCTAAAAAAAAGGCCGTGGGCAGAAAACTTGAAGAGTTACAATTGGGCTCGGCCCATCTAACGGCGCCAACTGAGCCCATAACATCCCTCCCCCCCGGCTTCCCCCATCTCCGTCCAGGCGTCCATCCATCAGCGGCGGCGGAGCGACACCAGCGGAGGCGGCGATCAGTTCGGCTGTCGGCGAGCAGGAGAAGCCCAGTGAGTTGCTCTCCACCTCATCCATCCTCATATCGCTCGCTTGCGTCCTTTCTCCAATTCGTTCTGACTACGTCGGGATCCTTAGGGTTCATACAGTTTGCTGGTGTGGATCCTGGTCTAGGCTGATCCAGTGGAGGATCATCGTGGGTGTCGTGGTCTCGTGGACATAGAGATTAGGGGCAATGTGATTTTCTCAGATTTCGTGGTGCTTCTTTCAGGGTCTTTTATGAGTAGTTGGTGGTCACAAATCTAATCTCAGGAGATGATTGATGCTGTTGGTCGTAAACGATTTCTGGAATCTCGTTTTGGTGTGGAGTATTGGGGAATGGAACTAATTAAGTGATTTTTTGTCAATTTCGTCAATGATTGGTTTCTGTGATTGCGTCCCGCTTCAATCTTATGCTTAGTTGGTGTAGATTCTTCCCCAGCACACTGTAGTTGGTAATATAGTTGCGAAAAGAAGCTATGATTAGTACGTCAAACATGAATACATCACTCGATCCTGATGAAGTGTCATTGATATGTGTGCCCTAGACAGCAAAGAGGAAGGGGCCTTTTGTTGGTCTGAAGGCAATAAGGTGTTGAAGAAATTTCATCAAGGATCAAACCTTGTGTCGCCTGTGCCATAGCCTTTTTGTTGAAGTTGTTATGTAGGAGGACTTGGGTAGTAGCAGCTATGTTGGTTCATGTCTTGCTGATCAGGCAGTTCTGAATTTGTTGTGTTGCTGCAGGGGTAGGATGGTGGCTGCGGCCAATTTCTCCTGAGTGGTTGCAATGTGGTGTTGTTTGGTTAATTTTTCCCCTTTTTTCATTTTAAAATATGTCAGTCTTGCTTTAGTGGATAGAAGAATGAAGTCTTTGCTTCCATGTTTCAACTGGAAAATTTCAATCTGTTTTATTCGTTTGTATGATGTAAAACTGCTACATGACATGGAATGGCATAAGCGGTTACTTCTTTCATTGGTATGGGAGCCTAGCTTGCTTCGACGAATAGTTATTGTTGTTCCATGGAATCTTAATTTGCGGCAAGTTCTCATATCCTAATCTCTTCTTCTTTTTTTCCTTCTTTTTTTTCTGTGATGGTACTCCAATCCACCTAATGTTGTCAGAAATGCACTTTACAATTAGGTTGTATGTTTCTGCATAGTTTCGTTTTCATGGGGTAATATATTAGATTTCACATTCTATATAGAATATGTTTTTAGTCATGATATCAATTACAAATGCAAATATATCCATTGCATCCTTCAATTCTACTTGCATTAAAAAAATAAAAAGGCTATGTAGTCATCATGACTGAAAAGTATGTAGATATCCTTGTTTCATTAGTGACAGTGGTGATGTAGTGGAAAGATGTTAGTACTTGGCACAAAGCATGACCATCCATCCTTAGGTGTATTTTCCTCAATAAATATTTGCGTATCTGGATGTATTCAAATTTATGTTTGTTAGAAATGTCTGGACTTGATAACACTCATATTTATGTTCCTTGTCATCAGCAGTCCCCCGTCCTTTTAGTACTGCATAGCATATGGTGATTGTACAATTATGGCTATGAGATAGGTTTGTGAATAGCAACAGGATTTTTATCTTTGCCATGCATTTTGATATTTCTACTGTGCTTAGTATAGATATTGATAATGAATTTACTAAATGCGTTGGTGCATTTCTTGCTTTCTGTTCGAAACAATCAAGTGCAGTAAGATCATCACTAATATCCTCAATTTAGCTTTGTAAAATTCGGCTAATCATGTCCCTTTACCAAGCTGTGATTCTGTATTGATTGCTAATGTACGATTATTGGATGTTGCGCAGTGATGGAGAAGTATCAAAACAACGCTAGGTTTGCATCCTTTAGTGACGCTCCATTTGCTCTCCGCTGTAAGAAATCATGCGTGGATTTGTTATTCTTGTAAGTACTATGTTCTTTGTCATTCTGGTATAGTTTTCAGATGCATCCATTAGGAGATAGTGGAGACATAACTGGCATCTCTTCAAGTGCATTAGGCATTAGCTCATTTAGGATGTGATGTAATATGGAGCTTGAAGTGGCAATCTGCTCTGCTGGCACAGATCTGAACTGAGCTGTTGACCAGTGAAGCAATACATTTCCCATTTTTCATAGCTCAAGTAAATGTTGATTCCAGTCCTTTGTAACTCTAGGAAACATTGATAGTGGATACTGATCCTTTCCACTGTTATTGTGTGTGTGAAAGACATTATTATATAAAACGTTTGATTTTTGGAAACGTACGGAAGTGCTTGTCCATGCCTTGTGGCTTTAACCATTTCAGTCCTCTTTCTATTTTATCTGGATATTGGTATTATGTGTTGTACAGCTTCATGGGGAAACACCCATCTACTTTGAATCTGTGATTCTAAATTATCTTCTTTTTTTTTGTATGAGCTTTCGTCAATGCAGTGGGACCTCTACTATATCTGTTCTAAGTTTGTTGCCTTCTTTTCAGGTGATCTTGGTAGCTTGATCTCGAATCTTGAGTAAGCTAAGGGCTACGCATCCACAGGTGCTCTCGACCAAGGACATCTCCATTAGGAAGCCGGCCTCCATGAAGGTCAAGATCTTGCAGTGGCATGCGGTGGCTTCATGGACGTGGGATGCGCAAGATGAAACATGTGGCATATGCAGGATGGCATTTGACGGCTGCTGCACTGACTGTAAGTTTCCAGGTGATGATTGCCCGCTCATGTGGGGTGCGTGCAATCATGCTTACCATCTCCACTGCATACTGAAGTGGGTCAATTCGCAAACATCAACGCCCCTTTGCCCCATGTGCCGCACAGAATGGCAGTTTAAGGGCTAATGCACATTAGTGGAGTATCTGGAATGTATCAGCACGATGCAGCTTTCATTATTGGGAGAAAGCTAGGATGTATTGTTGGAATGGAATTTTAGCATGCTGTCACTTTATATTGTGCTAAAAGGCTACAATCCTGATTATCAGCATCTGCTTAAGTGAACTTGTGCCGACTTGAGGTTGTGGCCAAGCAAATGAAATGATAATATATCAGAGGCGTTAAGCTTTCTGACCTCATTGCTATTTTTTGTGGTGCTATTGTTTACTTTCTGAAGGATTTAAAGTGTCGACTTTGGGTTTTGTTCTGGTAAAGTAGTAACAAGAAGCATGAAGCTGTATATGGCTTTGCTTGCTTTTATCTCTGTTCTCTTCTTCAACAGAAATAGTGATCCATAGGTTATCAGGTGTCACTTTAAGCTCTGCACACAACTTCAGTAGGCATGTGCGTATTATTTAGTCGTATTCTGAAAAAGAAGTATCTAAATTTTGTTTAAAGAAGGAACGAACGGGACATGCAAATTATGCTATCGTGGTAAATTATATCTATTCCTTCCAAAACTTTGTCCCTTGCAATTTGTTTCTTCCGTGCCAATGTAGTAATGGCAATATGCCAGCAAATTATTCCTTATTAGCTTATGCAGAAGCATGGATTGACTGTTAGGGCCGGTAGAGCTTTTCTAACGGCTTCACATAATTGTCAACCAAAGGGGTGCTTTTTCAGATGCTTCTTCTTAAAAATAGGAAGAAGCTTTATCTAAAAATAGGTTGAACCATGAAGCCACAAAATGGTATGAAGCCACANNNNNNNNNNNNNNNNNNNNNNNNNNNNNNNNNNNNNNNNNNNNNNNNGCGGGAAAAGCCGTTTTGCCAAATGTTTTGGGCACAGCCGTAGACACAGCGAGCACAACCGTTTTTCTAAAGAAGCCACAGCCACAGCCGAAGCCGGCACAGCCATAGCCCTACCAAAGAGGCCCTTAGTGCTTAGTGCATTGAGTTACTTTCCCCATTAATAGGGAGGCTTTCTGTATGTCTCATAGTTTGGTTCATATGAAAATTGGATGTATAGCAGTTACACGTTCATAGACTTGTTTTGTTACTTGTCCAAAATTCCCCCCCCCCCCCCCCCATCTGATTAAGATATATGGAGCCACCGCAAATATTTGTTTGAGATCTTGTTTTGCATGCAATTGTAAGTTCGATTACCATTAAGGATATATTGACAGTTAAAAAATTGTTAACCTTTGTTTGTTGGACGGACCCCATGCAGCCTTGGGCTTGTGCTAGGTTCCGTTGGCAGCCAAACTAAGCCAAATCGCCGGCGAGAAGTTTGGCCAATTGCACTGGGCACCAAATTTTTGGCAAGGTTGAGATCATGAGATGGGCAGTAAACCAAACACGCACTTGGATCTGGGGGTAACGTAGGTGTGCTAACAGTACATTCTAACCCGAGTGACAGCGCAACATGCAACATTTGCAAGCAACCAACATTTTTCTTCCTAGCCAAACGATTGAACACACCGGGTATAAACCAAATTAACTTGTTACCTCCTGTAAAAACAAAGCCCGTCTAGCTCAGTCGGTAGAGCGCAAGGCTCTTAACCTTGTGGTCGTGGGTTCGAGCCCCACGGTGGGCGTTATCTGTAGCACTCTTTTTTTGAACCCTATCTTTTCTTATATGATTACTTCAAAAATATACAGTGGAATTCTGGAATGTCACGTGCTTTTGTTAAATTTGTCTCTTCGTATGATAGTGCAAAATACGCTGGAATTCTAGAATGTCGCATCCTTTCGACTTGTCCGATTTGCTTCTTTATTCAGATCATGCTGGTCCTCCTCGATGAGCTTAACTGGCATCATATGTAGCCAGCCATATCTAATCAGCGTACAGTAAGATGATGATGAGCTTTGCCCTTAAATCCTCACTAATTCCCACCACGTCGCGGTACAACCTATAGCAACATCAAAACCATGCAAACTCCACGTCATCTCCTGCCGGCTCCAGCAGCAGCGCCTCCATGTCACCCTCGCCGCCGACGAACTGGCCGGCCGGCCCCACCACCCCGTCGCCGGCCTCCCCGTGCCACGGGCTCAAGAAACCCAGCTCGTCCATGTCGAAGTCAAGGTCGATGGACACGTCATCCTCCGGCAAATGCTCCTGTAGCACCTCCGCCGCCGCCGGCGCCTCCGGTGGATGGCCGTGGCTCTCGGCCGGCGAGGCGGCGGCGCGCGGCAGCCTGGCGGGGCACCGCATCGCCTTGGTCCGGACCGGTGGGCTCGTGTTGCCGCCGCCGTCCGGCGACGGAGGCGCGGCGTCGCTGCTGGTTCTCGCCGGCTCCGACGACGGCCGGCTACGCACGACGACGGGCGACGCTGCGCTGGCCTGATGACACTGTTTCGAGTGGCCGCCCACCTCGCCGCGCACCTTCTTGCCCAGCGTCGTGTTCCAGTAGTTCTTGATCTCATTGTCTGTTCGCCCCGGCAGCCGCCCGGCGATCAGGGACCACCTGTCCGGCGACGACGACAAAATATGCAAACACGTACGAAATCGTTAATTAGTCACTGCTGGATTGCAGTTTCAGAGACATAGCATTAGATGTGTCAATCATCGATGGTGCCTGCCAGCCTGCTGTTACTCGATCTGTCCCGTTGATCACATCAAGTTAGGAAACTAATCACATCAAGTTATTAGCAAGTAGATACGAAATACTTGCATTGGCAGCTAGCACAGTTAAAGTTTCCCATTTCTGAAACGACAACGCTTTTGCGGTCAGTGAAATTTTTTGGAATTTTCAGGGTGAAGGAAACGCGAGTTAGGAAGTGGTTACGCGCGCCAGAGGCCTTTCCTGAGTGCGAGATATTCTCCTGTTGCTGTATATTTAGGGAGGGAGGGACAGGGCTACTAGCTGTCATCATCAACTTTGCTGACCTAATAACTGGTTCATCTTCAGAGGGTAGGACTAGGAGACACTGCCCTGCCAACGAATACGTGATCTTCTAGAGCGCGAGAGCTAAACAGGAAAAAAGAGGTGCTGACTGATATGGACAAAGTGGCAGTAGTGGTTATGATAACTTGAGGCAATTGGCCGTTGTGCTTGCCTGCTGGCCTACTTTGTCTGCTCAGATCTCTACCACAGCAGGACAAGGAAAAGTTTCCAGCAGAAAGAGTTTAACATGCAAGCTAATGTGTTGTATGCATGAATAACGCAATTACCCTTCTGTCTTCTATACCATACAAACTAACATTTCTTTGAACAATAACAAACCAAATTTTAGTATCTTTAGGATTTAATTTGCGAAATGCAAATCGATGGAAATGATCGAAATATGGATAACCACATACCTGTTGCCGAGGAGCATGTGCAGCCTGATGATGAGCTCCTCTTCGTCGTCGGAGATGTTGCCCCGCTTGATGCCCGGCCTAAGGTAGTTGAGCCACCGCAGCCGGCAGCTCTTCCCGCAGCGCTTCAGCCCTGCAACACGTCAATGTCGTCAGTGAGGCCGTTGCGCATTCCCAACAGTCAGCGAATTACTGTACTGAATTTGTCATGTCACATTGCATTTTGTCCTTGCATCGCCCTACTTGTAACCGATCCAAGCGTACTTGCTCTCCGTTCCAGTTACAGAGGTTTATGTAGACTCGTATGGTAATCTAGTCAGCATTGATTATGCCTTTGATTAACAGTTTGTTTAGCGAATACGGTGAAAAAAAAAAGACCTGTTTTCATTTCATTTTTATCCTAATATCTGATCTTAATGTAACTTAGCATGCATGAACTACAAGACGTGACAAATCCATACATAGATGACGATAAAAATCAGAAATGACTAAAAAGAGAAGATAAGAAACAGGGGACAGGAGAAGAATTTCAGGATCAGACCAACTTCCCGCATTGTGAATAAATTGATGTTGATGACGAGAAATCAGCAAGAGAAAGGGAGGAGGTGTCACTGACCAGCTCTTTTGGGGAGGCATCCCCACTTGCCCTCTCCGTGCTTCC***TGTTGGAGTGTGTTGGCCCATATAGAGGCCCATGTATGTTGGATATATATATACCCACCCATCTAGGGTTGGAGGAATCAAGTCGTCCAGTACCCTAACTCTAACA***TGGTATCAGAGCCTCTCTCTCTCCCTACCCAACCCTAGCCGCCGCCGCCGCCATGGCCGGCTCGGCGGNNNNNNNNNNNNNNNNNNNNNNNNNNNNNNNNNNNNNNNNNNNNNNNNNNNNNNNNNNNNNNNNNNNNNNNNNNNNNNNNNNNNNNNNNNNNNNNNNNNNNNNNNNNNNNNNNNNNNNNNNNNNNNNNNNNNNNNNNNNNNNNNNNNNNNNNNNNNNNNNNNNNNNNNNNNNNNNNNNNNNNNNNNNNNNNNNNNNNNNNNNNNNNNNNNNNNNNNNNNNNNNNNNNNNNNNNNNNNNNNNNNNNNNNNNNNNNNNNNNNNNNNNNNNNNNNNNNNNNNNNNNNNNNNNNNNNNNNNNNNNNNNNNNNNNNNNNNNNNNNNNNNNNNNNNNNNNNNNNNNNNNNNNNNNNNNNNNNNNNNNNNNNNNNNNNNNNNNNNNNNNNNNNNNNNNNNNNNNNNNNNNNNNNNNNNNNNNNNNNNNNNNNNNNNNNNNNNNNNNNNNNNNNNNNNNNNNNNNNNNNNNNNNNNNNNNNNNNNNNNNNNNNNNNNNNNNNNNNNNNNNNNNNNNNNNNNNNNNNNNNNNNNNNNNNNNNNNNNNNNNNNNNNNNNNNNNNNNNNNNNNNNNNNNNNNNNNNNNNNNNNNNNNNNNNNNNNNNNNNNNNNNNNNNNNNNNNNNNNNNNNNNNNNNNNNNNNNNNNNNNNNNNNNNNNNNNNNNNNNNNNNNNNNNNNNNNNNNNNNNNNNNNNNNNNNNNNNNNNNNNNNNNNNNNNNNNNNNNNNNNNNNNNNNNNNNNNNNNNNNNNNNNNNNNNNNNNNNNNNNNNNNNNNNNNNNNNNNNNNNNNNNNNNNNNNNNNNNNNNNNNNNNNNNNNNNNNNNNNNNNNNNNNNNNNNNNNNNNNNNNNNNNNNNNNNNNNNNNNNNNNNNNNNNNNNNNNNNNNNNNNNNNNNNNNNNNNNNGTGTGAGTTGTTCTTTCTTGTGTCCAGTCCTGAACACCGCTGCGACGGTTAGTTCAGACTGCGGGGGGG***TGTTGGAGTGTGTTGGCCCATATAGAGGCCCATGTATGTTGGATATATATATACCCACCCATCTAGGGTTGGAGGAATCAAGTCGTCCAGTACCCTAACTCTAACA***CTTCCTGATGTAGGAGACGAGGATGTCATCCTCCATTGCTGTCCAAGCTCCCCTATTCAGCCCCTCCTTGGCTCTCTCTCTCTCTCTCTCTCTCTCTCTCTCTCTCTCGGTTAGATGGAGGGTTATGCTCTGTGATTTCCACTGAGGTCCCTATTAAACAAGGTGCAGAGCGCTCCGTATTCCATGCCCATTTTTTTTTAAAAAAAAATATTTGTGATATAGCCCCTCGAATGGACATTTATGCCCTACGGGCACTCTGTTTTCGCCCACACAGCTACTTCTGATCAGATGCTCGTTATGAAGGCAGTTTCTAATCGGACATCAGGAAGCTTTCGCTTGTTTATGCACATATAGGGAATGCTTAAAATTTTGACCAGCATAAAATTTCAGAAAATGATTTACAAAGCACCGAATTACTTAAGACCATATCGAATACGACAAGTGCATGTTCTAGATGCAGATGCCAATGTGGCACCTGCAACAGGCTTGAGAAGTACCGAGAGGGAAAACATATTGACATACAGTTTTTGTCAACTCTGATTCTTCTTCGTTTATCAAGGTTCAGTAGCCATGGTAATGAAAAAAAAATCTGATCTACAACCATGGTGTAGGAAACTACATCCTATCTGGGAATATACTCCTACTAATTCACAAATAGTCAACACTTACCTATTAATATAAATCTAAAAGGTGGATAATAGCACAGAATTTATTTGCAAATGCAACAAGCATAACCTTAGCTAAAATCCTTAAGCCTTCTCTTCAAAACATCCATGGACCTCTTGCGAGCACTCTGCTTAGCGTTACGGTTCGAAAGGCTCCCAAACAGCCCGTCAGCAGCGTCCTTGTACTTGTCACAAATGATGGAAAGCTTTTTGTAGTCCAGTGATCCGTAGTTCCTCTGTCTTATCACCGGATTCATGAAGTTCTCTGGTTGGCTACTGAGCAAGAGGTTGACTAGCTGCCTTATTTCAACCAAACAATCTCTGAAAGTTGTTTCCTTTCCCGAAACCGACAACCCCATGCTTTCGAATTTCTCATCTGCAAATGCCTCCAGCATTTTCAAGTCCATATCAAGACCTAGAACTGCATTAACAGTGAACCTTTTCACACCTTCGTTAAGAAGTGTTGTCATTATGGAGTCTGAGATATGGCTCATTATACCAGAAACCACTTTGTACAGAGCTTCCAAGGGCAAAATTTCCTGTGCTGTTGATACTTGAGTTTCGAGATATATCAGGACCTCATTCATATAGTCGTTTGAAATGTCTGGGGCTTCCTCTGCTATCCAGTTGACGTTTTCCAAGAGTATCATAAACTCATCGACCTTGAAATTGGCCAGGTTTATCAGAGCATTGTATACTGCATTCTGCGAGGCTTTCAAGACAGCTCGAGCAGTTAAACTAGAATGAGACCTCTCCGCAATGCGTTTTGGTATGCCACACAGATGAGCAGCATGCAAGAGGAACATGCCACATGCTTGCTCAAGAACAGAAATGTTACCAGCGAGCTGCATCATCTGTGACATGGCCAATGAGCGAGCATAGATCATGTTCAGAAGACTGTCATTTAAGACCTCAATCAACAGCCTGTCTAGGTATGCTTTCACAACATCATACATGTTCATGAAGCCACCATATGACAAGTAGCTGACGGAATCTTCAATGAAAGACCGAACGATGCGACAAATTTCTGGAACAGAAGAGGAGAACTGCGCCACATATGGGAAATCAGGGGCTGCATCATCAGNNNNNNNNNNNNNNNNNNNNNNNNNNNNNNNNNNNNNNNNNNNNNNNNNNNNNNNNNNNNNNNNNNNNNNNNNNNNNNNNNNNNNNNNNNNNNNNNNNNNNNNNNNNNNNNNNNNNNNNNNNNNNNNNNNNNNNNNNNNNNNNNNNNNNNNNNNNNNNNNNNNNNNNNNNNNNNNNNNNNNNNNNNNNNNNNNNNNNNNNNNNNNNNNNNNNNNNNNNNNNNNNNNNNNNNNNNNNNNNNNNNNNNNNNNNNNNNNNNNNNNNNNNNNNNNNNNNNNNNNNNNNNNNNNNNNNNNNNNNNNNNNNNNNNNNNNNNNNNNNNNNNNNNNNNNNNNNNNNNNNNNNNNNNNNNNNNNNNNNNNNNNNNNNNNNNNNNNNNNNNNNNNNNNNNNNNNNNNNNNNNNNNNNNNNNNNNNNNNNNNNNNNNNNNNNNNNNNNNNNNNNNNNNNNNNNNNNNNNNNNNNNNNNNNNNNNNNNNNNNNNNNNNNNNNNNNNNNNNNNNNNNNNNNNNNNNNNNNNNNNNNNNNNNNNNNNNNNNNNNNNNNNNNNNNNNNNNNNNNNNNNNNNNNNNNNNNNNNNNNNNNNNNNNNNNNNNNNNNNNNNNNNNNNNNNNNNNNNNNNNNNNNNNNNTCTCTCTCTCTCTCTCTCTCTCTCTCTCTCTCTCTCTCTCTCTCTCTCTCTCTCTCTCTCTCTCTCTCTCTCTCTCTCTCTCTCTCTCTCTCTCTCTCTCTCTCTCTCTCTCTCTCTCTCTCTCTCTCTCTCTCTCTCTCTCTCTCTCTCTCTCTCTCTCTCTCTCTCTCTCTCTCTCTCTCTCTCTCTCTCTCTCTCTCTCTCTCTCTCTCTCTCTCTCTCTCTCTCTCTCTCTCTCTCTCTCTCTCTCTCTCTCTCTCTCTCTCTCTCTCTCTCTCTCTCTCTCTCTCTCTCTCTCTCTCTCTCTCTCTGTCTCGGTTAGATGGAGGGTTATGCTCTGTGATTGCCACTGAGGCCCCTATTAAACCAGGTGCAGAGCGGTCCGTATTCCATGCCCATTTTTTTTTAAAAAAAAAGAGATAAATATTTGTGATATAGCCCCTCGAATGGACATTTATGCCCTACGGGCACTCTGTTTTCGCCCACACAGCTACTTCTGATCAGATGCTCGTTATGAAGGCAGTTTCTAATCGGACATCAGGAAGCTTTCGCTTGTTTATGCACATATAGGGAATGCTTAAAATTTTGACCAGCATAAAATTTCAGAAAATGATTTACAAAGCACCGAATTACTTAAGACCATATCGAATACGACAAGTGCATGTTCTAGATGCAGATGCCAATGTGGCACCTGCAACAGGCTTGAGAAGTACCGAGAGGGAAAACATATTGACACACAATTTTTGTCAACTCTGATTCTTCTTCGTTTATCAAGGTGTCAGTGGCCATGGTAATGAAAAGAAAAATCTGATCTACAACCATGGTATAGGAAACTACATCCTATCTGGGAATATACTCCTACTAATTCACAAATAGTCAACACTTACCTATTAACATAAATCTAAAAGGTGGATAATAGCACAGAATTTATTTGCAAATGCAACAAGCATAACCTTAGCTAAAATCCTTAAGCCTTCTCTTCAAAACATCCATGGACCTCTTGCGAGCACTCTGCTTCGCGTTACGGTTCGAAAGGCTCCCAAACAGTCCGTCAGCAGCGTCCTTGTACTTGTCACAAATGATGGAAAGCTTCTTGTAGTCCAGTGATCCGTAGTTCCTCTGTCTTATCACCGGATTCATGAAGTTCTCTGGTTGGCTACTGAGCAGGAGGTTGACTAGCTGCCTTATTTCAACCAAACAATCTCTGAAAGTTGTTTCCTTTCCCGAAACCGACAACCCCATGCTTTCGAATTTCTCATCTGCAAATGCCTCCAGGATTTTCAAGTCCATATCAAGACCTAGAACTGCATTAGCAGTGAACCTTTTCACACCTTCGTTAAGAAGTGTTGTCATTATCGAATCTGAGATATGGCTCATTATACCAGAGACCACCTTGTATAGAGCTTCCAAGGGCAAAATTTCCTGTGCTGTTGATACTTGAGTTTCGAGATAGATTAGGACCTCATTCATATAGTCGTTTGAAATGTCTGGAGCTTCTTCTGCTATCCAGTTGACGTTTTCCAAGAGTATCATAAACTCATCGACCTTGAAATTGGCCAGGTTTATCAGAGCATTGTATACTGCATTCTGCGAGGCTTTCAAGACAGCTCGAGCAGTTAAACTAGAATGAGATCTCTCCGCAATGCGTTTTGGTATGCCACACAGATGAGCAGCATGCAAGAGGAACATGCCACATGCTTGCTCAAGAACAGAAATGTTACCAGCGAGCTGCATCATCTGTGACATGGCCAATGAGCGAGCATAGATCATGTTCAGAAGGCTGTCATTTAAGACCTCAATCAACAGCCTGTCTAGGTATGCTTTCACCACATCATACATGTTCATGAAGCCACCATATGACAAGTAGCTGACGGAATCTTCAATGAAAGACCGAACGATGCGACAAATTTCTGGAACAGAAGAGGAGAACTGCGCCACATATGGGAAATCAGGGGCTGCATCATCAGGCTCCAAACAAAATGCTGTCACATTCATGTTATATTCGTATTCCTTCTTTATAACCATCTGCTCATATGAGTCATTAATAAGGATGTCATCTATCTGCTTTCGGCATTCAAGAAGAAGCAACTGGTGGTACTTGTCCCTGCTTTTCTCAAGAACTTCAATCAACTGTGTAATTTGATAACCATATTTCTTCACAGCTGCACCAAGAAGAGTAACATAGTCTTTTATGAGGAGGAAGTGGCTGGCTGTACGCATGCGGGCGAACTGCTCCTCCAATGTAGATGTGACTTTGCCAATAGCTGTTTCCCATGTTGTTTCCACTTGACTCTCAGACAACAACCCGTCTGCAGTTCGAAAGACACGTTCTTCAACTATAAAAAACCCTGCCACCTGAGAAAGGAAGGGCTGATGGGACTCCAGGAATGGCTGTGAAGTGGAAATTTGCATGTCCAAGTTGAGTTGCATGAGCCTATTTTTGTAATAGTAATCTTTGAACTTCTCCTCAATACCAAGGCATATGTGCATGTGATGTGCCCGGTACACTGGTGTGAGATCAAAATCTAGTGCCGATTCCTCTTCTGTATTCTCCAGATCCAAGGTATACAGATGCTCATCTGGTCCGGCATGGCTATGCCCTTCTGCCTCTCTCTGTCGTGCACGCATTTCCTCCTCTCTTTGTCGAGCTAATGAGGCCTGGCTTATTGAGACCTGGCCAATCTGCTTCGCCATTCTTCTGATATGAACAAGCCAATCATTGAACTCGCTGGTAACTTTCTTCTCAATATGCAGCTTTATCAGTGGTATCTGCCTCGCCACCACCCTTTTAATAAGTTTCAATGGAATATTTTGCAAGTATCCCTTCTGAATCAGGTCCAAAGTCTTCAGTGCAGGATGAAACTTAGCTTCTGCAATATAATTGTTGCATGTCATGCACAAGCTGATGACCTTCACACATATCTTTAGAGTCGTTATGGCCTCCCCAACGTTTTTCTTGACAGAATATAACTCAAGAAGTTCATCAAGCTTCAGCAATTGAGCAGTGGAAGCCTGTTGTAGGTGAGAATTCTCACCAGATAGCATGCTCTTCAGCTCCTCAGCATCGACCAACACCCCACGTAGCTCATCAACAGCAACGATGAAATCCTCATAGTGAAGTCTACATAGTTCTTCAATTTCAACTTCCTTCTTCTTCACAATGCTTCTGAGGCAATGCGTGAGAGCTTCAGGTTTCCCAGACTCAAATGAGTGACGAATGATGGGACCCAAATCATCGCCATTTGCAATAAATGTTGCAAGGCTCGAACCAAGGCCTCCATCTCCACTATCCACTACACTCCTCTTCTTTCCTTGAGCAGGTTGAGCAGTCATTGTTCAACTGTAGAAAAGACGAATAGGTACACAAGTTTCAGAACTAAATGGCATTGTGGTTCACGTTTCATCCAAAATAAAACAAAGATTGGGACAACCATACAAGACCGGATGCATTATAATTTTAGACGAACAGCAGGAATAGCAATTAGCAGCTGATAAGCTTCATTTTAATACCAAGTTTGCCTAATGAACAGTTAGTATATTAGGCATTGGCACAAAGAGTTCAAAACGTGATGCTTCCTATATTCATTTCTAGACTTTAATTTACAATTGTCTACAGTAGTAATTATATTATTGACTAAAGGATTCATTGAAAATATTAAAAGCTGTACCTCAATACTTGTAGAAATTTAGAGACTCACACTTTACTATTCATTTTGAAAAGACTCGCGCATAGCAATTACCGCAATAACAGATCGACTAAAACAATGAATTGAAGAAAAAAACACTGAATAATCTGAAAGCATTTCATCTTGGGAAAGAGATTGATAATGCACAGTTCTGGAATGATTCTCACCACGTTACATATTTCCAGCAGATAAGGCCAAACGTAACAATTGATTCAACCGGACACAACGCGATAATCGCACAAGAACACAAAGGGGGCCAACTAAAGACCGAAGCACCAACAGCAATTGTAATAACATGTAAAGCACAAAACGGGGTATAGCCGTATAGAGAATGAAAAGCTTACAGTGCGAGGGGCTCGTCGCGGTGCCAACCCTGTCTAGCTGCAGGATCCTGGCGCCTGCCCTCGATGCAGGACGGTGACGAACTGCGAGACTGCAGCACGGAGACGCAGCGACGATGGAGGGAATGGATGGGCGCGCCTCGCCTGCCGCCCGAGCCCTCTCGCCGCCGCCGAGTCGGTCAGAACCAGAGGACCGGATACAAGCGAGCGCCGGGTTGTTTGCTCAGAGAATACAGCACCAGCGGACGCAATGGGATCTTGAGAAGGAGAACAGACGGACCACCAGAACGACGAGTTGATCGAAACAACAAACGGAAGCCGGAAGGGGGCAAGTAGGAGGAAGAGGAGAGAGAAAGAGAGTAGGAGCTCCAACGGTTGCAGGGGAGGGTGTCCATTTGCACGGGCGGGTGGATCCACGAAACTCGCGCGTTTACCGATTCTCCTCCGCCCGCCTCGCTCGGCCGCGAGGACAGGTGGTCGCCGCCGCCGGTGACGGAGGCGAGATCGACGCGGAGCAACGGCTCCAATTNNNNNNNNNNNNNNNNNNNNNNNNNNNNNNNNNNNNNNNNNNNNNNNNNNNNNNNNNNNNNNNNNNNNNNNNNNNNNNNNNNNNNNNNNNNNNNNNNNNNNNNNNNNNNNNNNNNNNNNNNNNNNNNNNNNNNNNNNNNNNNNNNNNNNNNNNNNNNNNNNNNNNNNNNNNNNNNNNNNNNNNNNNNNNNNNNNNNNNNNNNNNNNNNNNNNNNNNNNNNNNNNNNNNNNNNNNNNNNNNNNNNNNNNNNNNNNNNNNNNNNNNNNNNNNNNNNNNNNNNNNNNNNNNNNNNNNNNNNNNNNNNNNNNNNNNNNNNNNNNNNNNNNNNNNNNNNNNNNNNNNNNNNNNNNNNNNNNNNNNNNNNNNNNNNNNNNNNNNNNNNNNNNNNNNNNNNNNNNNNNNNNNNNNNNNNNNNNNNNNNNNNNNNNNNNNNNNNNNNNNNNNNNNNNNNNNNNNNNNNNNNNNNNNNNNNNNNNNNNNNNNNNNNNNNNNNNNNNNNNNNNNNNNNNNNNNNNNNNNNNNNNNNNNNNNNNNNNNNNNNNNNNNNNNNNNNNNNNNNNNNNNNNNNNNNNNNNNNNNNNNNNNNNNNNNNNNNNNNNNNNNNNNNNNNNNNNNNNNNNNNGCCCCCCCCCCGCCGGTGACGGAGGCGAGATCGACGCGGAGCAACGGCTCCATTTTTTATGGGCCACGAGGCGTTCTGTGGCCTGCCCGGACTCTTTCGGCCCATTCCGACGGCAGTGGTGAACGGGCAGCACGGGCGAAGAAGAGCCTAAAGTTCCCAAGTGACACATTCATTTCTCCTGCTTGAGTCACCTCATTTCACGTGGTTTGATTTTCTGGTGTTGTTCCTTTAAAATAAACGAATAGATCTTATCAAATTCACCCCATATACGGGTAACATCTGGCCCTACGGTCACGGATAACGAACATCTCATTCCATGTGGTTTGATTTTGTGCCTCACGCGGATGAAATTTCTACAAATCACCAACAAAATACTGCTCGTTACTAAGGTTGAACCCATCCTCTGATGGAGCGTAAACATGGTACTTATGAGGCCTTCCGTTTGCGAGACCTCGAGGTTATGATGCAGATGGTGCCGTGGCGAGCATGGCAATCTTATTATATCATCGTTAGGCAAGATGGCTGCAACATGTTTGTTCCCCATAATTACAAAAAAAAAAGGTTCAAAAAACAAAAGAGCTTCAGATTCTTGCGGGACCGTCGTCCTTTAGCTAACGGCGGATCGGATCGGATGGCAATAATGACATGGATGGAACTTAAGAGTCAACAATCCTATCATCTGCTGGATTTATTCCACGAAAAGGGCCCTCAATTCTTTTCCATCATTCCCTCCATCTGTCTCTGCTCAAGGCGGCACTTACTTATGCTTTCCTGCATGTAGAGCAGCGCATGACTGAATGTGCTAGCTCAATTCAGCTTCAGGTCATGCTGGAAACATTGGCAGTAAGGAGTTGATTATTTTAGTTGCGCTCACCACCAGTCGATAACTATCTTGCGAGCATGTAAATCATTTAATTTGTTGTGATTAACACGATGTTCACGAATCACATATTTTATCCGAAGTGTGTCATCACATGCAGGCATCAGTTGGACTAATTTAGACAAATTACCATTAGTTGCTGAAGCATATGTTACAATCCAGGGCTGCTCCCATATTGTTATTATTTACTCCCTCCATGTCCCTAAGGGGAGTCGTTTTGAGTTTGTCCCAAGTCAAAAAATTTAAAACTTTGATTACAAATAATTTTTATGTGTTAAGTAATTAAGAAATATGAAAATTGTATGGTAGGTTCATATTGAAATATAGTTCAATTGAAGTACACTTTAACTACATCTTATAAATAGTTTATAACAAAAGTATTAGCCAAAATTATGTTTTAAAAACACGTGTGAATGTCCAAAATGACCTCTTTAGGGACTGGGAGGGAGTACGTTCCTTTGGCATATTAGGATTAATGTTTTAAAATTCGATTCAATGTACTTATTAAGTGGGAGCACAACATTCACGTGAAAATTCAAAAAAAATATTATTGAATAATTTACTTTGTCATCGGTTCTGGTGAATGTCAAATGCCCCTTTTCTTCTGCGTTGTCCATCAATTTTATAGTTTAGCGAGTTTCTTTATCACTCCAATATTATCATAACAAGTTACTTAAATATATGACACTATCGACTTTTTAGTTGAACTTTGACCAACAATATGGATTTAAATAATTAGTTACCTACTGTGCAATGAATATTATTGAATCTATCATTAAAAACACTTTAAGATTAACTTATACTCCCTCTAGGATTTTCTTGAATCATGTGTGTTTTCTCTTTAAATTATGTACTTTTTCTGTCTCAGAATATTTGGCATTCTAGCTCTGTACCAGATTCATTGAACATTCTCTGAATCTGAACAACCTGTCTGTCCAGGTTCATTGAACATTCTATGAATCTGGACATCAGAAAACACTCACAGAATGCCAAGTAAACTGGGACGGAGGGAGTACATGCATATAGCTATTTGAATAAATATTTATAGATTAGAAAAAGGTCAAAGTAACAAAAAGATCAATGATGTCATATAATATTTAAAATAGGAGAGACTAATCATTTCATGAAAATTCTACCATGAGTGTAAATCATTGCGGCGAAATTAAGACTTGTTGGTTTCCTTTACACTTGCACTGCATATTTTCCTCTTTCCCTTCCCTTCCTTCCTCCATTTCTCTATGTACATAAAAAAAAATTTACTAGTTTAAAGAAAATACCAGCAGTTCCCCTACTAGTACTCCAAAATGGAAAGAAAAACCACCCTGTTGCAACTTTTTGAATTGGCATTAGGGTTCATTGTAGATATTGGAGGCCTTAGAATTGAATTGGGTTCAATACCAAATTAGCTTAGGCATTGAAATGAGCTTAATGTTAATTGCGATTGTTTGGATGTAGATGGAATTGAGGGCTAGAATTCAACTCAGGAGCTGAATCCGGCGATGCAACTTAACAACACGATGGAATTTTCGAGTTCTTGTTCTACCACTGCGCTGCAAATTTTATCTAGATTTTTTAAAAATATATATATATATATATTTTTAAAAAATCTAGATAAAATTTGCAGCGCAGTGGTAGAACAAGAACTCGAAAATTCCATCGTGTTGTTAAGTTGCATCGCCGGATTCAGCTCCTGAGTTGAATTCTAGCCCTCAATTCCATCTACATCCAAACAATCGCAATTGGCATTGAGCTCATTTCAATGCCTAGNNNNNNNNNNNNNNNNNNNNNNNNNNNNNNNNNNNNNNNNNNNNNNNNNNNNNNNNNNNNNNNNNNNNNNNNNNNNNNNNNNNNNNNNNNNNNNNNNNNNNNNNNNNNNNNNNNNNNNNNNNNNNNNNNNNNNNNNNNNNNNNNNNNNNNNNNNNNNNNNNNNNNNNNNNNNNNNNNNNNNNNNNNNNNNNNNNNNNNNNNNNNNNNNNNNNNNNNNNNNNNNNNNNNNNNNNNNNNNNNNNNNNNNNNNNNNNNNNNNNNNNNNNNNNNNNNNNNNNNNNNNNNNNNNNNNNNNNNNNNNNNNNNNNNNNNNNNNNNNNNNNNNNNNNNNNNNNNNNNNNNNNNNNNNNNNNNNNNNNNNNNNNNNNNNNNNNNNNNNNNNNNNNNNNNNNNNNNNNNNNNNNNNNNNNNNNNNNNNNNNNNNNNNNNNNNNNNNNNNNNNNNNNNNNNNNNNNNNNNNNNNNNNNNNNNNNNNNNNNNNNNNNNNNNNNNNNNNNNNNNNNNNNNNNNNNNNNNNNNNNNNNNNNNNNNNNNNNNNNNNNNNNNNNNNNNNNNNNNNNNNNNNNNNNNNNNNNNNNNNNNNNNNNNNNNNNNNNNNNNNNNNNNNNNNNNNNNNNNNNNNNNNNNNNNNNNNNNNNNNNNNNNNNNNNNNNNNNNNNNNNNNNNNNNNNNNNNNNNNNNNNNNNNNNNNNNNNNNNNNNNNNNNNNNNNNNNNNNNNNNNNNNNNNNNNNNNNNNNNNNNNNNNNNNNNNNNNNNNNNNNNNNNNNNNNNNNNNNNNNNNNNNNNNNNNNNNNNNNNNNNNNNNNNNNNNNNNNNNNNNNNNNNNNNNNNNNNNNNNNNNNNNNNNNNNNNNNNNNNNNNNNNNNNNNNNNNNNNNNNNNNNNNNNNNNNNNNNNNNNNNNNNNNNNNNNNNNNNNNNNNNNNNNNNNNNNNNNNNNNNNNNNNNNNNNNNNNNNNNNNNNNNNNNNNNNNNNNNNNNNNNNNNNNNNNNNNNNNNNNNNNNNNNNNNNNNNNNNNNNNNNNNNNNNNNNNNNNNNNNNNNNNNNNNNNNNNNNNNNNNNNNNNNNNNNNNNNNNNNNNNNNNNNNNNNNNNNNNNNNNNNNNNNNNNNNNNNNNNNNNNNNNNNNNNNNNNNNNNNNNNNNNNNNNNNNNNNNNNNNNNNNNNNNNNNNNNNNNNNNNNNNNNNNNNNNNNNNNNNNNNNNNNNNNNNNNNNNNNNNNNNNNNNNNNNNNNNNNNNNNNNNNNNNNNNNNNNNNNNNNNNNNNNNNNNNNNNNNNNNNNNNNNNNNNNNNNNNNNNNNNNNNNNNNNNNNNNNNNNNNNNNNNNNNNNNNNNNNNNNNNNNNNNNNNNNNNNNNNNNNNNNNNNNNNNNNNNNNNNNNNNNNNNNNNNNNNNNNNNNNNNNNNNNNNNNNNNNNNNNNNNNNNNNNNNNNNNNNNNNNNNNNNNNNNNNNNNNNNNNNNNNNNNNNNNNNNNNNNNNNNNNNNNNNNNNNNNNNNNNNNNNNNNNNNNNNNNNNNNNNNNNNNNNNNNNNNNNNNNNNNNNNNNNNNNNNNNNNNNNNNNNNNNNNNNNNNNNNNNNNNNNNNNNNNNNNNNNNNNNNNNNNNNNNNNNNNNNNNNNNNNNNNNNNNNNNNNNNNNNNNNNNNNNNNNNNNNNNNNNNNNNNNNNNNNNNNNNNNNNNNNNNNNNNNNNNNNNNNNNNNNNNNNNNNNNNNNNNNNNNNNNNNNNNNNNNNNNNNNNNNNNNNNNNNNNNNNNNNNNNNNNNNNNNNNNNNNNNNNNNNNNNNNNNNNNNNNNNNNNNNNNNNNNNNNNNNNNNNNNNNNNNNNNNNNNNNNNNNNNNNNNNNNNNNNNNNNNNNNNNNNNNNNNNNNNNNNNNNNNNNNNNNNNNNNNNNNNNNNNNNNNNNNNNNNNNNNNNNNNNNNNNNNNNNNNNNNNNNNNNNNNNNNNNNNNNNNNNNNNNNNNNNNNNNNNNNNNNNNNNNNNNNNNNNNNNNNNNNNNNNNNNNNNNNNNNNNNNNNNNNNNNNNNNNNNNNNNNNNNNNNNNNNNNNNNNNNNNNNNNNNNNNNNNNNNNNNNNNNNNNNNNNNNNNNNNNNNNNNNNNNNNNAAATATATATATATATATATTCAAATTTTCAAAAAATTTTTGAATTTTGAATTTGAAAACCAGACCGGTTTTTTCTAGTAACCAGTCCGGTTACCAACGGTTTTGCAATCATATTTTTGTCTTAAATTTTCATTTTTTTGAATTTTGAATTTGAAACCGGACCGGTTTTTTTTTGAATTTTTCTTGTAACCGTTCCGGTTACCGACGGTTTTGCAATCATATTTTTGTTTCAATATTTTGTAATTTTTTGTTTGAAATTTGAATTCAAAATTTGGCAATTCCTGATGAAGACACTCTAATGCTACTGAATTCAGAGGGTCAGGACTCTGAATTATGGACAGGACAACTCAGTTATGTTTCAATGCCATGGCGTTGGGCTTTAAATGTCAATTCCTAATTCTGGCGACATCCAAACACTAAAATTGATTTAGCCTAATACCAATTCCTAATTACAGCTTTCAGTTATAACGTCCAAACGTTACATTAGGTTTATTGCAGCTCCCAATAACGTCTTCACTCTTCAGACTCGGGCACACATGCAAGGCACAGTTCAGACTATCACAGGCAAAGGCCATGCACACAGGTACAGAAGCATGACCTTGTGCAGCCGTTGCTTGCGCTGGGCAGAACAAAGCGCACTTTCAGTGTCACCCGTCTAAAATTTAGATGAGTTCTAAAAAAAATTTGTTATTTAAGAGTATTAATTACAAACTAACTGCTGAATACCCTGTCTAATTCGCGAGAGGAATATGAGGATATGAGGGTACTTAGGGTGCGTTTGGCTAGCAGAATTGTTTCGGATTCTGGCTTGAATCGCGTGAAACGGTGCCAAACGGAAAAAAGGTGAATCGTTTCGAAGGAGAATCAGAGAGAAACGTTTCGTAAAAATGAACTACTCCAGTGAATCACCCAAATCGGTGTTCCGCGGCGATTCGTTCCGATTACTCCTTTGCCCCTGTCTGGTTCGACTCCGTTCATCCGTCACAGCCGCCGGACGGAGCGCGGCTCGGTCTTCTGCTTCAAATCCGGCCTCAAAAATAGATTGCTCTTAGTCCGTGGATCATTTCCCCTAATTATCTCCCTCTGATTAATCGCTTGAGCAGCGCCAATCGAACCCTGTGTCTACCTTTCTCTCTCTTCCCCGTTTCTGTTTTGCATCGTTCTGACTTCTGAGGAAGACGACGACCTGGCGATCGATGGACGTGGTGGTGGGCTTGCAGGCTGAGCCTAGTGGGCACCCGTATGACGAGCCCAACAATTACAATTTCTTTTCTTTTTTTCTTTTATATTAAATGTGGCATGTAATTTGTATGATGAGCTCATCAAATACATTTCTTTATTTTCTAAAATAATTTTGACATATATTTTGTAAAGATGGCATTATTACTAAATCTCTTATAAATACTATATTATGAGACTTCATTGTTACATATTGTAATATGGCAGAAATAACATAATGCAACCAAGGGTAAAATGGTCAAGTTGATCCTCATCCTCTCTTTCTTGGAACTGAAACCAGAAAACTATCCAAACGATTTCTAAAGTGATTCTGTTTCAAGTTGGGAAACATTTCTAAATTGAATCTGAAACCTTTTTTATTCTCGATGAATCTGGAGCTGTACCAAACGCACCCTTAATACGCGATTAGACGATGGTTACTGTAGCATTACTGTAGCATCATTGTAACAAAACATAGACTAATTAGGCTCATTANNNNNNNNNNNNNNNNNNNNNNNNNNNNNNNNNNNNNNNNNNNNNNNNNNNNNNNNNNNNNNNNNNNNNNNNNNNNNNNNNNNNNNNNNNNNNNNNNNNNNNNNNNNNNNNNNNNNNNNNNNNNNNNNNNNNNNNNNNNNNNNNNNNNNNNNNNNNNNNNNNNNNNNNNNNNNNNNNNNNNNNNNNNNNNNNNNNNNNNNNNNNNNNNNNNNNNNNNNNNNNNNNNNNNNNNNNNNNNNNNNNNNNNNNNNNNNNNNNNNNNNNNNNNNNNNNNNNNNNNNNNNNNNNNNNNNNNNNNNNNNNNNNNNNNNNNNNNNNNNNNNNNNNNNNNNNNNNNNNNNNNNNNNNNNNNNNNNNNNNNNNNNNNNNNNNNNNNNNNNNNNNNNNNNNNNNNNNNNNNNNNNNNNNNNNNNNNNNNNNNNNNNNNNNNNNNNNNNNNNNNNNNNNNNNNNNNNNNNNNNNNNNNNNNNNNNNNNNNNNNNNNNNNNNNNNNNNNNNNNNNNNNNNNNNNNNNNNNNNNNNNNNNNNNNNNNNNNNNNNNNNNNNNNNNNNNNNNNNNNNNNNNNNNNNNNNNNNNNNNNNNNNNNNNNNNNNNNNNNNNNNNNNNNNNNNNNNNNNNNNNNNNNNNNNNNNNNNNNNNNNNNNNNNNNNNNNNNNNNNNNNNNNNNNNNNNNNNNNNNNNNNNNNNNNNNNNNNNNNNNNNNNNNNNNNNNNNNNNNNNNNNNNNNNNNNNNNNNNNNNNNNNNNNNNNNNNNNNNNNNNNNNNNNNNNNNNNNNNNNNNNNNNNNNNNNNNNNNNNNNNNNNNNNNNNNNNNNNNNNNNNNNNNNNNNNNNNNNNNNNNNNNNNNNNNNNNNNNNNNNNNNNNNNNNNNNNNNNNNNNNNNNNNNNNNNNNNNNNNNNNNNNNNNNNNNNNNNNNNNNNNNNNNNNNNNNNNNNNNNNNNNNNNNNNNNNNNNNNNNNNNNNNNNNNNNNNNNNNNNNNNNNNNNNNNNNNNNNNNNNNNNNNNGTCTCGTAAAGATGTACCTATCTGTAAAAAGGTTTTGTCATTAGATTTTATTTAATACTCTTAAATAATAAGATTCTCTTTAATTTTAGAGATCTAAAGTCTAAACACTAATACAACGAACGCGTCCAAAGGTGTGGTCAAAAAGGGCTGGCATGCCGCCTTCTTTTGGTTACCAGATTCTGGTCAAGTGCTGTATGATTGGAATCAGGGAAGGCATGCCAGGTAAAAGATCCTTTTCACGGTGATTGCGACTTTTTGTGTCGGTAATGCATTGCTTGCCCATGATCCCTTTTGAATTCCCCTGCTACTGTAGTGGAAGCTTTATTCATAATCCTAGTTTTAGGATAACATCTTCGTTGGTTACTTAGAGGTCACATCAAAGAGAATTGAGAATTTTGTCTTTTAAAAGTATTAAATAAAGTTTAATTACAAAATTTTTTTATAAATAAGAGCTAATTTTCGAAACGAAACCAACGAGCCTAATTAAGCTATGTTTCGCTTTAGTGATGCTACAGTAACCATCTTTTAATTACGGTTTAATTATCTTCATTAGATTCGTCTTGCGAATTAGCCTAGAGACCCTGCAATTAGTTTTGTAATTAGACTTTATTTACTACTCTTAAATGACAAGATTTTAAATGGCTAAAATTTTGGCAAAGAAACGAACACCCCATTAAATTTTTTTCCGTACTTGAACTCCTCTATTTTGGTATTTCTTTCTGTGCATGAGATTCCCAATGAAACCACTATCTGCTCAGCATTCAGGTTCTAAAAATCAAATGAAAGGTCTTGAGTTACTTGCCTCCAAGAACTTCTCGCAATGCTGCCAACGGACCTAACCTACAATGACTCCATCATACAGACAAATGCTTGGCCGTCATACAGTCGAGAAACACACAAAATCGGAATGCTATATCAGTTGGTTTTTCAGCGAATTCAAAGAACTGAAGCCATTCAAGTAAACCAACTGTGAGAGAGATACAAACAAACTAAAAGAGTTTATAGTATGTTCCAAATAGGTCTTGAAAATTGGGATTCGGGCAGAGCAACTTTTATTGCCAGAGCTATATATTCCAAATAGGCCTTTAAAGACCCATTTGTAATTCCAGAAAAACTAAAAGAGTTCACACATCCTTTTAGCAGAGGCCTTCTATCCATGAAAATGAAACGGCATAGGCGAATCTCATGGAATGCCCTACGTCTACAAGCAGGCGCCTACCACGACAAACATGCCATCCCCATAAAAAGGCGTGACGACAAAGCACTTTACATTTCCCTGCCCAGCTACAAAAAATTTTGGGCCAGAGAGCGAAAGAGCTGCAATCATCCAATGCTGAATGAAATGCCCAGCATTCACAGATTTTTGCAGAAAGTTGATGCTGCAAGCAAAGCGAAAGTCTTGGCAAAGAGTACGCTAGAAAGAAGCAATGGTGGCATGCATCGGCGCGGAAGGGCACACGATACAGAATTACAGATGGCGAGCGAGCCTGATGAAAGAAAGGCAAGCAGGACAGCGTGGAAAAGCAGAGGGCAAAAGCATTCGGGGGCAAAGCAACCCGATCAATCATGGGCAGAGTTCATGCATGAGCTCAGTGCCTTCTCTGACAGCGTAAAGCAGGGATGAAATCTAGGGAAATTTTAGTGAGAACATCAGAGTTTTTTTACCCCCTAGATTTTGAGGGCCCATCCTGGCATCATCCAGGCGGTTTGGTCATTGATCAACCCATAACAAGAACAAAAGATATTGTAATCTTTTGAGGTTTTCATTGAAAATTGCGAGTGAAAAAGTTGTTGACGCCTGACAAAGCATGAAGCATATTGAAAACAAGGTAAATAAACAAGCGAGAGGACCAAGGGAAACAAATCTACAACTGGACATAATTCGATTCTACAACCACAGGTTCACCGCAGTATCATCAATCATCATTTCAGAATGGGAAACTGAGAGAACCCATCACTCATGTCATCATTTCAGAATGGGGAATTCAGAGTACTTGTCAACCTCTGTATTGCATCGTGAATTCATACCTCTAGCTCGTACCAACAATGTAATAAGATTAAACATCAGCTACAAGAAGCTCATAAGTAATTGTAGGAGAAGATTGCTCTCGCTCACATATACTAGTCGGAGGAGTACGAATAGGTGGTTGGTAGTAGATACAAGAACAGCTCAAGTCAAGAGAGAGCTCCCTAGCTAATAGCCCCTACTCGGGCTAAGAAGTTTAGAACAAGTTAGTCGAGGCCGGACCTGATCCTCTTGTGGCCGGCGCCGGCCTGGCGCACGGTGCCTACGGAGCCGCCCTCGGATGCGGCGCGCCCGCCCTCGGCGGCGGCGAAGAGCGAGGAGAGGTCGGCGTAGAGGTCGACGACGCGGCGGATGTTGTTGTTGAGCTCCCTGATGAGGCCGACGTTGCGGGAGAGGTCGCCGGGCACCTTGGACTCGTGATTCTGGTTGATCTCGTTGATGAGCACCCGGTTCTGGTCCAGCAGGCTCTGCACCTGCACGAAGCTCGTCTGGAACGCGTGCAGCACCTTGGTGTCCACGCCGGCGCCGCCGAACCCCGACAGCGTCGTCTCACCGCCCTCCATTACTTTTGCTTTTTCTTAGCCTTCCTGACCGCACTGCGCGGCTATTACTAGCTTCAGGAAGAAACTGGTTTCTTTGACCTCAGCTCTGCAGAGACCAATGGAGAAATGGTGAGAATGATAATATGGCAGAATGCTCTTGCAATAGATGCTTCAACTGATAAAGATACGTGTTCACTTGCGTTTAAAAGATTGATTTTTTATTAACACGGGTGGTTGGCGTCTGGGTCATCAAGATTTGGACCTTTACTGCCGGTCTCAGCAAAGGGAGAGATTTCACTGTTGGGCCATGGGAGGACAACGGTACTGTGCTACAAGATCAGATGTACTTGGCGAATCTTTCTTAGCTTGGACCCCCGAGAAAATCAAACCTTGGAATTAACTCGGTTACCCCTTTTATATTTTTCAAAACCAAAGAACAGATAAAGCCCGTTGATTGAAACAAGAAAGAATACCGAGCAACACAAAGCGCGTCAGATTTTTCTTTTTCTTTTGGAAGAAAAACGAATATGATAAGGGGAAGGCTCGCGCTTCCGTGCTCGTGTACAAGAAGCACGGATTGGGAAATGATTGAGATCCGCTCGGAATTCCCCAAAGCATGTGCAGATGATACTACTAGTAGTAGNNNNNNNNNNNNNNNNNNNNNNNNNNNNNNNNNNNNNNNNNNNNNNNNNNNNNNNNNNNNNNNNNNNNNNNNNNNNNNNNNNNNNNNNNNNNNNNNNNNNNNNNNNNNNNNNNNNNNNNNNNNNNNNNNNNNNNNNNNNNNNNNNNNNNNNNNNNNNNNNNNNNNNNNNNNNNNNNNNNNNNNNNNNNNNNNNNNNNNNNNNNNNNNNNNNNNNNNNNNNNNNNNNNNNNNNNNNNNNNNNNNNNNNNNNNNNNNNNNNNNNNNNNNNNNNNNNNNNNNNNNNNNNNNNNNNNNNNNNNNNNNNNNNNNNNNNNNNNNNNNNNNNNNNNNNNNNNNNNNNNNNNNNNNNNNNNNNNNNNNNNNNNNNNNNNNNNNNNNNNNNNNNNNNNNNNNNNNNNNNNNNNNNNNNNNNNNNNNNNNNNNNNNNNNNNNNNNNNNNNNNNNNNNNNNNNNNNNNNNNNNNNNNNNNNNNNNNNNNNNNNNNNNNNNNNNNNNNNNNNNNNNNNNNNNNNNNNNNNNNNNNNNNNNNNNNNNNNNNNNNNNNNNNNNNNNNNNNNNNNNNNNNNNNNNNNNNNNNNNNNNNNNNNNNNNNNNNNNNNNNNNNNNNNNNNNNNNNNNNNNNNNNNNNNNNNNNNNNNNNNNNNNNNNNNNNNNNNNNNNNNNNNNNNNNNNNNNNNNNNNNNNNNNNNNNNNNNNNNNNNNNNNNNNNNNNCGAATCTCATGGAATGCCCTACGTCTACAAGCAGGCGCCTACCACGACAAACATGCCATCCCCATAAAAAGGCGTGACGACAAAGCACTTTACATTTCCCTGCCCAGCTACAAAAAATTTTGGGCCAGAGAGCGAAAGAGCTGCAATCATCCAATGCTGAATGAAATGCCCAGCATTCACAGATTTTTGCAGAAAGTTGATGCTGCAAGCAAAGCGAAAGTCTTGGCAAAGAGTACGCTAGAAAGAAGCAATGGTGGCATGCATCGGCGCGGAAGGGCACACGATACAGAATTACAGATGGCGAGCGAGCCTGATGAAAGAAAGGCAAGCAGGACAGCGTGGAAAAGCAGAGGGCAAAAGCATTCGGGGGCAAAGCAACCCGATCAATCATGGGCAGAGTTCATGCATGAGCTCAGTGCCTTCTCTGACAGCGTAAAGCAGGGATGAAATCTAGGGAAATTTTAGTGAGAACATCAGAGTTTTTTTACCCCCTAGATTTTGAGGGCCCATCCTGGCATCATCCAGGCGGTTTGGTCATTGATCAACCCATAACAAGAACACACAAAAAATTGTAATCTTCTGAGGTTTTTACTGAAAATTGCGAGTGAAAAGTTGTCGACACCTGACAAAGCATGGAGCATATTGAAAACAAGGTAAATAAACAAGCGAGAGGACCAAGGGAAACAAATCTACAACTGGACATAATTCGATTCTACAACCACAGGTTCACCGCAGTATCATCAATCATCATTTCAGAATGGGAAACTGAGAGAACCCATCACTCATGTCATCATTTCAGAATGGGGAATTCAGAGTACTTGTCAACCTCTGTATTGCATCGTGAATTCATACCTCTAGCTCGTACCAACAATGTAATAAGATTAAACATCAGCTACAAGAAGCTCATAAGTAATTGTAGGAGAAGATTGCTCTCGCTCACATATACTAGTCGGAGGAGTACGAATAGGTGGTTGGTAGTAGATACAAGAACAGCTCAAGTCAAGAGAGAGCTCCCTAGCTAATAGCCCCTACTCGGGCTAAGAAGTTTAGAACAAGTTAGTCGAGGCCGGACCTGATCCTCTTGTGGCCGGCGCCGGCCTGGCGCACAGTGCCGACGGAGCCGCCCTCGGAGGTGGCACGCCCGCCCTCAGCGGCGGCGAAGAGCGAGGAGAGGTCGGCGTAGAGGTCGACGACGCGGCGGATGTTGTTGTTGAGCTCCCTGATGAGGCCGACGTTGCGGGAGAGGTCGCCGGGCACCTTGGACTCGTGATTCTGGTTGATCTCGTTGATGAGCACCCGGTTCTGGTCCAGCAGGCTCTGCACCTGCACGAAGCTCGTCTGGAACGCGTGCAGCACCTTGGTGTCCACGCCGGCGCCGCCGAACCCCGACAGCGTCGTCTCACCGCCCTCCATTACTTTTGCTTTTTCTTAGCCTTCCTGACCGCACTGCGCGGCTATTACTAGCTTCAGGAAGAAACTGGTTTCTTTGACCTCAGCTCTGCAGAGACCAATGGAGAAATGGTGAGAATGATAATATGGCAGAATGCTCTTGCAATAGATGCTTCAACTGATAAAGATACGTGTTCACTTGCGTTTAAAAGATTGATTTTTTATTAACACGGGTGGTTGGCGTCTGGGTCATCAAGATTTGGACCTTTACTGCCGGTCTCAGCAAAGGGAGAGATTTCACTGTTGGGCCATGGGAGGACAACGGTACTGTGCTACAAGATCAGATGTACTTGGCGAATCTTTCTTAGCTTGGACCCCCGAGAAAATCAAACCTTGGAATTAACTCGGTTACCCCTTTTATATTTTTCAAAACCAAAGAACAGATAAAGCCCGTTGATTGAAACAAGAAAGAATACCGAGCAACACAAAGCGCGTCAGATTTTTCTTTTTCTTTTGGAAGAAAAACGAATATGATAAGGGGAAGGCTCGCGCTTCCGTGCTCGTGTACAAGAAGCACGGATTGGGAAATGATTGAGATCCGCTCGGAATTCCCCAAAGCATGTGCAGATGATACTACTAGTAGTAGAAAACAAGAAACTGAGCTATACACTGTACGAAACAAGAAATTGAGCTATACACTGTACGAAGGGGGCACGAAATCCAAATATCCCGAATTCGCCTTCTAACCGCGGCCGAATTCGGGAACCGTGACCCAAATTTTGACAAGACAGCGAAACGATCGCTTTCTCTTGTTACCTTTTTCCCTCAATAATACACAAAAAGAGAGAAATTCAGGCGCAATCGGCCTCGAAGGTACCTCAGCCCGGGATACGGATTTACTGGAAACGGGAAAAGAAGGAATGGGATCCACCCATGCACGGAAACGGAGGGATTTGCTTCCCTTTTCTCTGGCCATCTTCCGGATGTTCTAGAGGGAAGGCGGCGGCGGCGGGAAGAACTCGTCGCACTCCAGTCTAATACGGAGTAATGAATGAAGAACCCAACTCACGTCGGCCCGAAGATTCGAAGAAGACAAGAAATCGAAAGAGATTGGATTTTTCTGGGAAACGAACAACACAACGGAGATTTCGACTGGAGAAGAACGGAACCAGACCAGAGAAAGAGCCGTAATACTAATACAGTAGCAGGCCTCTACCCGAATTCAACTTGGTGGATATGGCAGAGAGAGGGCGGGAGGTGGCGGTGCTTACCTCGGGACTCCGAAGCATCGACGAGGAAGAGAAGCTCCTCCTCTCGGTGAGATCACGAATACGAGCTGAGGAGAGAAGAGACAGGAGGATGAGGAGAAAAGGGGGCAGAGAGGGGAATGGGGGTGGATAGAGAAGAGAGATCAGGAGATCCTCAGACACACGAAACAGCCCGGCTTGCCTTTTCTTTATTGTTCTGCGGGCTGCGGCGGCACAGAGAGAGAATCTTTTGCTAGAGAAAGAGATTTGTGGCATGTTCCGACACGTTTCTCCTCCCCCTGCTCACGCGCACAACCTCCCCCCGATGTCGCTGGCACGTACTGTGCGTGTCTCCTGATATTTTCGGCGATCTTTCCAGTGAATCACACTCGAAGAAATTCGAATGTTTTTCGTGGGACAAAGCTGGACGTTGTACTACTACTGATCGAGTGGCTTTCATCTCACACTTGACTCTCCTCAGCTCATTACCCAAAGGGGGCCGACGAGCAACAAACTGTCCTACTACTATTATATATCTACCTCTGGCTTATACTACATGGGATTTAAAAGAACTCCGGCAGTTTTTGATGAGAAGAACATGTACGTATATATCTCTAGAAATCGCCATCACCGCCTTTCGACTGTCCAACGGCGGCAAAAATACAAAGGTTGCTGATACGTGCCACCTGAACCGTACGTAAGCTTTCCGTTGGATCTATCATTTTCTCTTTAGGGCCTGCCTGTACTCGTGCAGCTCGCTGGGTACAGATGATGGACAGGAAGCTTCGCATAAACGCCAAGGCACACTTGCTAATTGAATATCATTTGTTAGGTCAAATCACAATTATCTCTCGTGGGAGACAAGTGGAAAACCAGTGCCCTTAATTATCAGAGGTAGGCCAGCTAGGCCATCACGAAATCAGATCTCCGGCTCGCTCATTCCCTCTCTCTCTCCCCTTCCTAGGTTGCAGCCAAATCCAGAGGCCAAAATGCTTTGGCTACATGGAACTTCAACACCTACAAGCAACTACCTTGGGGTTTGACAACTTGATTGCCCTTTTTGTTTAAGATTTCTTAGATCTAGCTGCCAAAGATTTAAGTTTTCTGGAAAGAAACAAAAGCAGTTGGGCATATGGCATTGACTTCTTGTACTCTGACTTATTTTTTCCTTTTGAATCAATCTCGTACTCTGACCTGAACAGTCAAAAACCCAATCTTCGATAGATTAGCCACGAAGAGAAGCTAGTACTTTGGTAGATGTAGAATTCATCTAGAACCCTCTCTATGAAAGGAAAAAGTTTCATATATATCACAGAATACGCTGCCACTCTCATAAAAACAATGGACATTCTGATTGCGCAAATGTGTGTTTACAGTAGTACTTATAGTTTTTTTTGCGGGAAGTAGTATTTATAGTGATGCATCTGTTTTTTTTTCAGAAATGCAGAATGCATTTTTACAGAATTTAGAATCTAGCAGTCACCACTGGCTCTGAACTTTCCAGGTAAAGCATCATTTCATTGTTTGTGGCCTTCAGTATATGTCATTGATTTGGTCACATAGGGATCCAGCTTCAGCTAACTGCAGATTCGTGAAGGCTTCCTGGAGGCTCAGGCAGAGATGTTTCCATGCACTTTTCAAGAAGGCCAAATGATCTGAGATACAACTCCTTTTTACAATCTTCAAATCCTTTATTTCAGCCTCCTCTCCTGTTTTGTTTTGTAACATTTCCACAAAATGGTGAAAGAAAATAGGCCTTTGATCTTGCATTTACTATACTTCATCATCGCTTTTCATCTGTTGAGCGAACCATTCTTTCCAGTCCTCCACCTTTTTATCACCATTTGCAACCTGACATTAGATTAGAAGCTAGATTTGAATATTTTCTTCACAGCTCAGAGAAGAGAGTTATGGAACAACATTTCATATGTTCTGTTACCTATCCGACAACAAAAGACGGAAAACAAAATGAAATAATGATCTATGATACGAAGACCTGAAGCCATCCTAAGCTGATTACCTCAAAAATGAGCGTCTTTGGGGGAATGGCATCCAGAGCTTCCACACATATGGTTGCAGCATCCTCTCTGCTTATTCTTCCCTTGGCTGCTACCCCCTGCATATTTGAAGAATGGTATTGATATGCATAGGTCCAGATAGCCAATACGAGAAATACATGAAGCATATTGGCATTGTTGTATGAAGTTACACCGTACATAATAACCACAATGATGTCTATATATGCCTTACTCATAGCACATGCTAGCACAACTGCCTGAGTAAAATAGGTGGCAATACATCCTTTTTGCAGTGATACCTCTGTAAAATCGAAACCCCTCTCACCACCAGGGGTGCTTTGCAGAGAAGCAGTCCTGATTATCGTGGAAGGGATGCCAGATGCAAGAACCACTTCTTCATCCCTCTCTGCTAACTTCTTCAGTTTGCTATTCAGAATAGCTTGTATCCCACCACTATTTCTGTAAACAACCAGCTGCACTCTTATCAAGATCGCATAAAGGAGAATGGCTCAAACTAGGGTCAGCTAGATAAACGGAATTGCAAAAGCATTGCAACCTGTGACAGCAGAACAATGTGTTGGACACCCTTGAGGTCAATCAAGTCTGAGAAGAAACCATCCTATCAAAACATAAAGTTTTTTTTTAGAATCCATCAAAATATAAAGTTAAAGCAAAGCATATATTATATAATGCAAAGAAAAGGCACTCTAGAAACAGATAATATGTACATATGTATAGGTAGCAGTGCTAAAATAAAATGCACATGGCAATCCCTTGGATCACAAAATTATACTAAAAAATATTCAAATCTAATGAGCGTTTCGCATTGAAATTTATAAAGCAGAAACAAGATCTAGTAATGCCTACTCAAGTTATACATGTATTGACTCATGGAAGAGCTAAACTATCAGAAATTATTCAAACAAAAAATGGACAGAGTAAGATAGAGGCATACATCAGCTGGACAGATTACTGAACGAACACCCCTTAGTGCTTTCTTCGTGAAGGTCTTATCTTCCACATCACCAACCATGCACTACAAGTGAAAAAAAATTATGATTGTTTGGATAAGGTTTATTATATGTACAAAGAAAAATGTGACAAGGTAAATTAATCGAATACGAGTTTTCTTTCTGTAACTAACTTCACCTCTTAAACAACAGTAAAGCAATATGATATCGTCTATATCAGAATACAGAATGTTGCAGCAAATGTCAAGCAACAAGATGTCACCTCCACATAAGTTCCAAAAGCCTCTTCGGTGGATCGTTTGTCTTTAACAAGTGCTTTTATTCTAGCTCTTTTCAGGATCAGTGCCAATATCACCATCTGGAAAGACAAGGAAATGAATTTTCACATGCTGAGTGAATTTCTGTCCTTTTCTTTGGGGAGACTTACACGAAACTTCAGTTTTTTCTTGAATAAACTGAAATGTTAGGTGTTTATATAAGCAACCAGTAGTTGGTCCAGATAACATTTTGTTAGAATATTACGTTCCACTTAGGTCACGAGGGAACAAAATGAATATAGGCAAATTTCAGGATAACAATGCACGTTTTGCAGCAGAAGACATAAATGAGCATGGATATAAGCACAACCTGCCCAATCTCACTGTCGCCATTGGTGACTAGCACAGCGTCCCGAGGCTCTTCGATATCTATTCTCACACCAAATCTGCCAAGAAATAAGAAACAATGGCAGCCAAGTACTAGTTTACAGAAGTACTTTCTCTATGCACCTGAAGACACATCCTCCTCATCCTCCGCACCCCCATCCTTGGGAAGCAGATCCGGCGCTCCATACCATCTCCTTAACTTTGGCCCCCCTATAAAATGATGTCAAAAAAAAAAGTTTAGAACTAGCTTGGATTGCTGGTGTACTTTTCTTCAGTTTTCTGATAATACACATGAGAGCTGTGTATCTTTGTATCAAGAAAAAGGTGTTCATTTTACAATGCGAGGCGCACTTTGACTTTTGCCAACCGTAGTTTTTCACTAGTGGACTATACTAAGAACTAAGAAGCATGGACAACTGAATAAGGTCAATGGACACTGAAAGCATACAAATACTCTGCTGTCACAGAGTGCTGATGCCCTTGGCACCCTCTTGTACAATAGCCAAGCTCCTCTTTGAGCGCTTGCAGTCATATTGCCCTTCAGTTTGTTCAAATTTTACTTTTCAATGGCATGCATTCGACTAATGCCCACAATTACGGGCGCTACATCTTATTTTCCTTCAAATGCACGATATATTCTTGTTTACTCTTCTTTATTGTCTCAATATTTGCAAAAGATGTGGCAAACGTTATGCTTGTCATAAACAATTACAGAATGCCACATATTGGCAAAGAGCATTGGCCAAGTTAAGTATACCCTCAATTAATCCATCATTGCTAAAGCAAATAGCTAACAGAATCACCATCGAATTCTGGACTTGGTAGAGCCATTGGCTACTAACGATATAGTGGACACTATTTGGCTGTTGCAAACCAAGGATATATAAGTTGGTTTGCTTAACTCAGTGCTCACTACCCAGTGATTCGTAACTATCGATATAGAGTTCTGCTACAATAAAAGATTCAAGAGGTTGGGGCTCGCTGTGCTGATTCAGAAAAGGCCCACTGAGCCAGGTAAAACTGATCACAAGTCCTCGCATCGGTTTTGTAACAAGAGAGCACGAACGCGTACCCTCGATGTAGTCGAGGATGCGGTCGGTGAAGCTGGCCTCGCCCTTGCTCTTGGCGCGGACGACGGCCGCGGCGCTTCTTCGTGGCTTCCGGGAGAGAGGGGCGCCCCACACNNNNNNNNNNNNNNNNNNNNNNNNNNNNNNNNNNNNNNNNNNNNNNNNNNNNNNNNNNNNNNNNNNNNNNNNNNNNNNNNNNNNNNNNNNNNNNNNNNNNNNNNNNNNNNNNNNNNNNNNNNNNNNNNNNNNNNNNNNNNNNNNNNNNNNNNNNNNNNNNNNNNNNNNNNNNNNNNNNNNNNNNNNNNNNNNNNNNNNNNNNNNNNNNNNNNNNNNNNNNNNNNNNNNNNNNNNNNNNNNNNNNNNNNNNNNNNNNNNNNNNNNNNNNNNNNNNNNNNNNNNNNNNNNNNNNNNNNNNNNNNNNNNNNNNNNNNNNNNNNNNNNNNNNNNNNNNNNNNNNNNNNNNNNNNNNNNNNNNNNNNNNNNNNNNNNNNNNNNNNNNNNNNNNNNNNNNNNNNNNNNNNNNNNNNNNNNNNNNNNNNNNNNNNNNNNNNNNNNNNNNNNNNNNNNNNNNNNNNNNNNNNNNNNNNNNNNNNNNNNNNNNNNNNNNNNNNNNNNNNNNNNNNNNNNNNNNNNNNNNNNNNNNNNNNNNNNNNNNNNNNNNNNNNNNNNNNNNNNNNNNNNNNNNNNNNNNNNNNNNNNNNNNNNNNNNNNNNNNNNNNNNNNNNNNNNNNNNNNNNNNNNNNNNNNNNNNNNNNNNNNNNNNNNNNNNNNNNNNNNNNNNNNNNNNNNNNNNNNNNNNNNNNNNNNNNNNNNNNNNNNNNNNNNNNNNNNNNNNNNNNNNNNNNNNNNNNNNNNNNNNNNNNNNNNNNNNNNNNNNNNNNNNNNNNNNNNNNNNNNNNNNNNNNNNNNNNNNNNNNNNNNNNNNNNNNNNNNNNNNNNNNNNNNNNNNNNNNNNNNNNNNNNNNNNNNNNNNNNNNNNNNNNNNNNNNNNNNNNNNNNNNNNNNNNNNNNNNNNNNNNNNNNNNNNNNNNNNNNNNNNNNNNNNNNNNNNNNNNNNNNNNNNNNNNNNNNNNNNNNNNNNNNNNNNNNNNNNNNNNNNNNNNNNNNNNNNNNNNNNNNNNNNNNNNNNNNNNNNNNNNNNNNNNNNNNNNNNNNNNNNNNNNNNNNNNNNNNNNNNNNNNNNNNNNNNNNNNNNNNNNNNNNNNNNNNNNNNNNNNNNNNNNNNNNNNNNNNNNNNNNNNNNNNNNNNNNNNNNNNNNNNNNNNNNNNNNNNNNNNNNNNNNNNNNNNNNNNNNNNNNNNNNNNNNNNNNNNNNNNNNNNNNNNNNNNNNNNNNNNNNNNNNNNNNNNNNNNNNNNNNNNNNNNNNNNNNNNNNNNNNNNNNNNNNNNNNNNNNNNNNNNNNNNNNNNNNNNNNNNNNNNNNNNNNNNNNNNNNNNNNNNNNNNNNNNNNNNNNNNNNNNNNNNNNNNNNNNNNNNNNNNNNNNNNNNNNNNNNNNNNNNNNNNNNNNNNNNNNNNNNNNNNNNNNNNNNNNNNNNNNNNNNNNNNNNNNNNNNNNNNNNNNNNNNNNNNNNNNNNNNNNNNNNNNNNNNNNNNNNNNNNNNNNNNNNNNNNNNNNNNNNNNNNNNNNNNNNNNNNNNNNNNNNNNNNNNNNNNNNNNNNNNNNNNNNNNNNNNNNNNNNNNNNNNNNNNNNNNNNNNNNNNNNNNNNNNNNNNNNNNNNNNNNNNNNNNNNNNNNNNNNNNNNNNNNNNNNNNNNNNNNNNNNNNNNNNNNNNNNNNNNNNNNNNNNNNNNNNNNNNNNNNNNNNNNNNNNNNNNNNNNNNNNNNNNNNNNNNNNNNNNNNNNNNNNNNNNNNNNNNNNNNNNNNNNNNNNNNNNNNNNNNNNNNNNNNNNNNNNNNNNNNNNNNNNNNNNNNNNNNNNNNNNNNNNNNNNNNNNNNNNNNNNNNNNNNNNNNNNNNNNNNNNNNNNNNNNNNNNNNNNNNNNNNNNNNNNNNNNNNNNNNNNNNNNNNNNNNNNNNNNNNNNNNNNNNNNNNNNNNNNNNNNNNNNNNNNNNNNNNNNNNNNNNNNNNNNNNNNNNNNNNNNNNNNNNNNNNNNNNNNNNNNNNNCCGCTACGGACGGCCGAAGCGGCGACGGCGGCGGCGTGGGGCGCCCGGTGGAGGGGCCCGGAGACAGCGGAGGCGCGGGCATTGGAGGGAGGGGAGCGCGCGCGTCTCCGGCGGATTTGGTGTGCGTGACCGTGGTTTCGCGGCGGGATAACGCTTGGACGGGTGGTGCGTTTCATCCGCGCCACGAACCTCCACGAGGGGACGTTGTGACAGCGCAGTGTGACAACACCGACCACTATGCTTCATATGCCTAGGGATGGCAATGGGTAAAAACCCGTCGGGTTTAGACTGTCAGCAGCAGTACACTGTAAACCGGCCGGAACGCTAAACTTAGCGGACGAATGGTCCTAAACGCGCTGCAGCAGCTGCCTGTAAACCATCCGCCAGAATCCAGGTTCGCCTCGCGCACCTCCAAATGTGGGGGCTGCCGTCCCGTCCTCCATATCGCCTCTCCTCCCCCTCTACGCGCCTCCCCTCCCGGCGCGATGGGACAAGACAGCGTCGTGCCAGGCGGGCGAGCACGAGGACGTGGCGCAGCTGGTGGTGGAGGTGGACTTCCGGTCGGAGTTCGAGTTGGCGGCGCCCACCAATGCGTACCGCGCGGCGCTCACCAATGCGTACCGCCCACCAATGTCATGTCGAGAGCGTCCTCTTTACGCCCCAGAAATAGGAAATGCATATACATACAAATAGGAAAACATGCTTTTCCAGTCGGGAATTGAAAAACTAGAAAAACATATATGAAGCCCTTTTAACAGCATGAAAAAATATGATATGATACTTGCACAAAACATCAGTTGCTTCTCATTTGGAATTTAAACAGCCAGCCGCTAGGCACTATTCGAGTTTCTTGGTTTCCTTGTGTTCGAGAACACTAGAAAAACAGAGAAAAAAAGATAAGAAAATTATTTCTGCAAAAAAAACATGATACATACTTTGTGTTGGTAGTGAAAATCCTTCTAGATTAGATAAAGACAGCAAGATAGTTGTTGCATGGTGCCTACACGTCTTTAGTTATATATGTCTGTAACTTCCATGTTTTTTCATTGTCCAGCAAGTTAGATATTAGCAGCACTTGATTATCTCAAACTCCCACTACAAGATCTTTTGCTTGGCAATAGAACATGCCGCCACTACATTTGGTGAGTCATAACTGAACATCTCTGTATATGCATGTATGTGAGAATAGTAATCCATTTCATTATTACCATTCTCTCAGGCGATAGATTTGTTAAAACAAACCAACAACACATATGACTATCTAATTACTTGTTGATAAACAATGTCATGGATCCAAAGTATAGACTACTATCACAAAGTGAAGATGAATTCATGTTTTCCTTTTTCTTTATCGCAGAAGGGGTTCATCACAACCATCATCCAGTAAGAAACCGATTCATACATTAAAACTTTTATGAGATTGTCATAGTGAAATCTCTACACATGAGGGCCTAATTAGTAAAAGGAACTTCAGCGAGAAAGCTATTGTGTTTTTCATACTTTGGTCAGTATGTTGCGTGAGAAGTAACTTCCTGTCGATTTAAGAGCTATCTCAGTCTCAGGAGAAGAGCAAGTTATTAGTGAACCACATGACAAGTAGAGCAACTCCCTCCATCAAAATCTCCTACACATAGGACACCCTTGAATCAACATGTAGACTAACCCAACACGCCACCTAGCCCAAAGTAGCCCATAGTTGGTCGTAGATATTATGAAACAACTGATTGAACATTTTGTAAAATAATAACCCTACAGTTTCGGAAAAACAAGATCAACATTCTCATATAGAACCACAACCAAATCTTGATTTAATACTCAATAAAATGTTTAATTAGTAAACTAAAAAATGAGTTTTGGTATTATATTTATGGATTTTAAAGCTATCAACCTTGGATACCATTTTACAGCAATAATTTCAAATATTATGGTGATCCGCACGGATTCAGACGAACTTAATGGTTTAGGAGAAAAATACATTTAAAAAATTTGAAAACCTTCTCTTTTTCTCCCGGCACCCACTTCTCCTTCTCAATAGGAGGCCAACATGTGGCAAAATCTGGCTCCACCTCTTTGCAACTGTACGGATTCACATAATAGGTTGATCAATTCTTTTTTTTTTGAATTGTAGTGTCGGTGTTATGAAACCGGCCGACAAGTAAAATTTGTAAATCGGGGGCTCGACGGCTCTCTTTGATGGCGAGGTTTTTCGTTTGAGGGATTCGTGGAGAGAGAGATTTATACTGGTTCGGGCCAACGGTGCCCTACGTCCAGTTTCGGGTGAGGTTCGTGTTTCTCGTCTGTAGAGAAGAGCTACAAACGACGGCGAGGGAGGAGTGTTGCTCCAAGCCTCTGAGTGTGAATGTGTTCGATTGATCCTCCTTCCCCCTTTGCAGGGTGCNNNNNNNNNNNNNNNNNNNNNNNNNNNNNNNNNNNNNNNNNNNNNNNNNNNNNNNNNNNNNNNNNNNNNNNNNNNNNNNNNNNNNNNNNNNNNNNNNNNNNNNNNNNNNNNNNNNNNNNNNNNNNNNNNNNNNNNNNNNNNNNNNNNNNNNNNNNNNNNNNNNNNNNNNNNNNNNNNNNNNNNNNNNNNNNNNNNNNNNNNNNNNNNNNNNNNNNNNNNNNNNNNNNNNNNNNNNNNNNNNNNNNNNNNNNNNNNNNNNNNNNNNNNNNNNNNNNNNNNNNNNNNNNNNNNNNNNNNNNNNNNNNNNNNNNNNNNNNNNNNNNNNNNNNNNNNNNNNNNNNNNNNNNNNNNNNNNNNNNNNNNNNNNNNNNNNNNNNNNNNNNNNNNNNNNNNNNNNNNNNNNNNNNNNNNNNNNNNNNNNNNNNNNNNNNNNNNNNNNNNNNNNNNNNNNNNNNNNNNNNNNNNNNNNNNNNNNNNNNNNNNNNNNNNNNNNNNNNNNNNNNNNNNNNNNNNNNNNNNNNNNNNNNNNNNNNNNNNNNNNNNNNNNNNNNNNNNNNNNNNNNNNNNNNNNNNNNNNNNNNNNNNNNNNNNNNNNNNNNNNNNNNNNNNNNNNNNNNNNNNNNNNNNNNNNNNNNNNNNNNNNNNNNNNNNNNNNNNNNNNNNNNNNNNNNNNNNNNNNNNNNNNNNNNNNNNNNNNNNNNNNNNNNNNNNNNNNNNNNNNNNNNNNNNNNNNNNNNNNNNNNNNNNNNNNNNNNNNNNNNNNNNNNNNNNNNNNNNNNNNNNNNNNNNNNNNNNNNNNNNNNNNNNNNNNNNNNNNNNNNNNNNNNNNNNNNNNNNNNNNNNNNNNNNNNNNNNNNNNNNNNNNNNNNNNNNNNNNNNNNNNNNNNNNNNNNNNNNNNNNNNNNNNNNNNNNNNNNNNNNNNNNNNNNNNNNNNNNNNNNNNNNNNNNNNNNNNNNNNNNNNNNNNNNNNNNNNNNNNNNNNNNNNNNNNNNNNNNNNNNNNNNNNNNNNNNNNNNNNNNNNNNNNNNNNNNNNNNNNNNNNNNNNNNNNNNNNNNNNNNNNNNNNNNNNNNNNNNNNNNNNNNNNNNNNNNNNNNNNNNNNNNNNNNNNNNNNNNNNNNNNNNNNNNNNNNNNNNNNNNNNNNNNNNNNNNNNNNNNNNNNNNNNNNNNNNNNNNNNNNNNNNNNNNNNNNNNNNNNNNNNNNNNNNNNNNNNNNNNNNNNNNNNNNNNNNNNNNNNNNNNNNNNNNNNNNNNNNNNNNNNNNNNNNNNNNNNNNNNNNNNNNNNNNNNNNNNNNNNNNNNNNNNNNNNNNNNNNNNNNNNNNNNNNNNNNNNNNNNNNNNNNNNNNNNNNNNNNNNNNNNNNNNNNNNNNNNNNNNNNNNNNNNNNNNNNNNNNNNNNNNNNNNNNNNNNNNNNNNNNNNNNNNNNNNNNNNNNNNNNNNNNNNNNNNNNNNNNNNNNNNNNNNNNNNNNNNNNNNNNNNNNNNNNNNNNNNNNNNNNNNNNNNNNNNNNNNNNNNNNNNNNNNNNNNNNNNNNNNNNNNNNNNNNNNNNNNNNNNNNNNNNNNNNNNNNNNNNNNNNNNNNNNNNNNNNNNNNNNNNNNNNNNNNNNNNNNNNNNNNNNNNNNNNNNNNNNNNNNNNNNNNNNNNNNNNNNNNNNNNNNNNNNNNNNNNNNNNNNNNNNNNNNNNNNNNNNNNNNNNNNNNNNNNNNNNNNNNNNNNNNNNNNNNNNNNNNNNNNNNNNNNNNNNNNNNNNNNNNNNNNNNNNNNNNNNNNNNNNNNNNNNNNNNNNNNNNNNNNNNNNNNNNNNNNNNNNNNNNNNNNNNNNNNNNNNNNNNNNNNNNNNNNNNNNNNNNNNNNNNNNNNNNNNNNNNNNNNNNNNNNNNNNNNNNNNNNNNNNNNNNNNNNNNNNNNNNNNNNNNNNNNNNNNNNNNNNNNNNNNNNNNNNNNNNNNNNNNNNNNNNNNNNNNNNNNNNNNNNNNNNNNNNNNNNNNNNNNNNNNNNNNNNNNNNNNNNNNNNNNNNNNNNNNNNNNNNNNNNNNNNNNNNNNNNNNNNNNNNNNNNNNNNNNNNNNNNNNNNNNNNNNNNNNNNNNNNNNNNNNNNNNNNNNNNNNNNNNNNNNNNNNNNNNNNNNNNNNNNNNNNNNNNNNNNNNNNNNNNNNNNNNNNNNNNNNNNNNNNNNNNNNNNNNNNNNNNNNNNNNNNNNNNNNNNNNNNNNNNNNNNNNNNNNNNNNNNNNNNNNNNNNNNNNNNNNNNNNNNNNNNNNNNNNNNNNNNNNNNNNNNNNNNNNNNNNNNNNNNNNNNNNNNNNNNNNNNNNNNNNNNNNNNNNNNNNNNNNNNNNNNNNNNNNNNNNNNNNNNNNNNNNNNNNNNNNNNNNNNNNNNNNNNNNNNNNNNNNNNNNNNNNNNNNNNNNNNNNNNNNNNNNNNNNNNNNNNNNNNNNNNNNNNNNNNNNNNNNNNNNNNNNNNNNNNNNNNNNNNNNNNNNNNNNNNNNNNNNNNNNNNNNNNNNNNNNNNNNNNNNNNNNNNNNNNNNNNNNNNNNNNNNNNNNNNNNNNNNNNNNNNNNNNNNNNNNNNNNNNNNNNNNNNNNNNNNNNNNNNNNNNNNNNNNNNNNNNNNNNNNNNNNNNNNNNNNNNNNNNNNNNNNNNNNNNNNNNNNNNNNNNNNNNNNNNNNNNNNNNNNNNNNNNNNNNNNNNNNNNNNNNNNNNNNNNNNNNNNNNNNNNNNNNNNNNNNNNNNNNNNNNNNNNNNNNNNNNNNNNNNNNNNNNNNNNNNNNNNNNNNNNNNNNNNNNNNNNNNNNNNNNNNNNNNNNNNNNNNNNNNNNNNNNNNNNNNNNNNNNNNNNNNNNNNNNNNNNNNNNNNNNNNNNNNNNNNNNNNNNNNNNNNNNNNNNNNNNNNNNNNNNNNNNNNNNNNNNNNNNNNNNNNNNNNNNNNNNNNNNNNNNNNNNNNNNNNNNNNNNNNNNNNNNNNTCGGACGAGGCGGCGGGCGCGGGCCTCACTCTCGCGGGCTTCGCACGCCCAGGTTAAGGCTCTTTGACCGGTTTTATCTGGGTTAAGCGTCCCAGATTAGGATACCTTAACTCAGGTATCCGACATGTAGGTTCATCAATTCTTGTGTGAATGTTAAACACTCATCTAGCATTCCGATATTCCTTTTTTTAAGGAACATCCAGATATTCGCATGGCGTGCCCCTAAAATCCTTTCCCCAATCACACATTTTTTTTTTTGAGGAGTTTTCCCAATCACACTTGCTAGCAGCAATTCGTGCCCAGCCCATGTAATAAGCCAATCGAGGGCCGGCCCACAATCATCTCCAGCTCCAGTCGTTAGGGTTTGTTTCGAGGCAGCTGTGCTACGCCGCAGGAGCTCCTTTTAAAACCTGGACGCCTTGCCGCAGGTCCGAGACGGCGGCTGCTAGGGTTTCGAGAAGGCGAGGCGAAGCTCCGATACCCCATCCCCTCTCCTGAGTTCGAGTCGAGGGAGCCGCCATGAGCCGGTCGGGGCAGCCTCCGGATCTCAAGAAGTAAGTTCCCGCCTCCATGAGCCTCCACTCTTCGTCAGATCTCACTTTGTTCTGTTCACCGTGCTGATTTTTGGTTTTCTTTTGTGCGCATCGCAGGTACATGGACAAGAAGCTTCAGAGTAAGTTGAAATATCTGACTCCAAACTCATGGCCTTGTAGTCTACATGTAGTTATCCTGCTAACCTGTCAACGATCCAGCTCATCTGATCTCTAGACGAATTAAATGGTAGTCCCCGTAGCTCTTACTAGCATCGGAGCATGTAAAAAACATGTAGTTTACATGTTGCTCTGTTTTACTGTTCCTTATTATCTGTGGTCATCTTGACAAATGAAGTGGATTTCTGTTTGCTTTCTGTACATTTGCGTCAGGTTTTATTTGTCAATTTCAAACCTTTTCGTAGGTTTCCTGGATGCAACTTTGTAAGTGTAGCCCCAGCCTGCATTTTGTTTTTGAATTCCCAATGCCCCAAATGATGAACATAATATAAAAGCTATGGATTTATCTCCAGATGAAGCTTTATGTCATAGTTACTCTGATGGGCATGTACTGGCATTTTTATTTCTTTTGTAGCTAGCAAGCGTGCAGGTTTCTGATATTCGCAGTGCGAGGCACCAATTATTGTGGTTGTCATTAACTACAGCGCTGCACGTTTGGTAATGACTGCCTGGATGATGATGACATAGTGAAATAGTCTTTTTTGTTAAATTTCCATAGTCGTTTTAGTTACTAGTCTTTTTTTTAAAAGTAATCCGTGTTGGTTGGCTGTCCTACATGATGATGATATTCCAAAAGCTATGGATTAATTTCCAAATGAGGCTTTTTTTGTCATAGTTACTCTGATAGGCTGCCATATCAACCTTTTGAACTTGCTTACTTGCAATCAGCCAATGCCTTTCATGGAACAGATGTTCATGACAGTTCCTAAATGTTGGTAAGTTTATGTAATTTGAGGATGAGCATTTTTGTTTTATAAAGTGTAGCAGCAAATTCTTTCCACCAGAAAGCTTGTTCCCAGTATCAATGTGATAATCACTTATAGCACCACAGAAACAGCACTTTATAGCATTTTGATTGTTTGAAAACTTCACTGGCCAGTTCAGTCTGTTTACTAGGGTCCTTCTACTTGTTAGCCATCCAGGCTATTGAACTCTTGTGCTGTGATGTTTTAGCGTCAGAAAGCAACCCAAAAAGATGCGTGGCTGAACAGCCATGTCTTTACTCTGGAATTACTTGTACCAGATAGCATTTGAATGCGTTGTCTCGAAAACTTCATGAATACTATTTATGTTCTTACACAATCGCAGGCAGTAACAACTGAACTAAGATCTTTATGATTTTGCTAAGGCTTCATTTGTCTGAAGGTGATATTTTGCCAATATCCATGCCAGAAAATTTAGAACATAAATCCTGTAGCCCAAACGAGATGTCCAAATAAGAACATCCAGTCTCACTTCATTACTGATCCAATTTTATGATCAACAGCTAGAAAAATGTTGGCGTTGGCAAGTTACTCTTTGACTATGACTAGTGTTAAATCTTTGCCTTAATGGATCTAAGGTACTTGCATTGGATTTTGCCTGTTGATGAGTGGCTTAATGTTTACCATTTGTTCTTTTCTACAGTCAAGCTGAATGCAAACCGTGTGGTTGTTGGCACACTCCGTGGGTTTGATCAGTTCATGAATCTGGTGGTGGACAATACTGTGGAGGTCAATGGAAATGATAAGACTGATATCGGGATGGTGGTAAGTTGTCAGCTATTGATATGTGCTTTTTAATGAATATATACTTGGTTAGTTCAAATTTGAGCTAACCAGCGTGAACAGTAAATGAACTGATTGTATTTAACTGTGTTAAGCCGTGGTTAGTCAATAATAATATGCATCGTAAGCTAATGTCCTGAAATCATCCGCAGGTTATCAGGGGGAACAGTGTTGTCATGATTGAGGCGCTGGAGCCCGTCGCCAAATCACAGTGAATTTACGAGGCATTATTCAGCATCAGTAGATCTAGCCTTGGCACTGTGAAAGCTGATGTATATGATCAGTGAACATGTTTGTACCTTGACTTGTTTGCACAGCAATATAACGTAGGACGTGGTTGATAACTCTTTACATGTAGTATTTTTTGTTCTCTGCACATATGTGGTCACTTATTAACTAAGGTTACCTCAGTAGTGCCCGAATGCTTGGTGTTTGCATATTACTACTACCTTGTCACCTTCACAGCAGGCCCAGTTTAATTTGTGCTGGCTCCTGGTTCTAAGGTGACAACATCAATAGCTTGTTTTGCATTTTTGCAACCTTTTCCAGTATGCTCTGGGCAATGAGCGATTCGGTAAATTGTTGCTACGTTGCCTGTTGTTTTGGAGACATCTCATTGGGGTACATCCAACACAACCCTAAGTGCTCAATCAGTTTAGCTGTACTTGTTCATTGAGATTTGAGAGTCGATCAGTTTAGCTGTACTTGTTCATTGGGAATTGTATTTCATATGCGTGAGGAGAAGCAAATCGCCAGATTGTCGTGAAACTGTAGGTGTGGGGTTACCAGAGCTGCTAAATTTTATTCATTGCCAGCGCGGTAGATCACGGCTCTGGTGCCGATGGACTGGCTGTGAGTGTAACCAGTCTCTTTGATATAAGCACAGTATAGTTTTTCCTTTTTACTTGAACTTAATGAGACGGCCATGCAGTCCAATTAGTTTAACTAGACTTGCATATTTAAGTTTGAAGAAAATTAGTAGAAAAATAAAGTGAGCTCTTGTTGCTCATAGAAAAAATAATTTCTTACATTGTTAGTGACTATGACATGGATTGGTGAGAGCTTAGCGGAACAACATGGCTGGTGACCCTCTGAGAGAGCGCTTCGTGGAAGGCATAGAAGCCGAAGCGAGGGCCAAAGCATTGCTGTTAACGGGGTTAAACAGTTGCCTGATGGTAGTCTTTTTTTTTTGGCATCATAACTCGCTCATTTAGTCATTGTGAACTGGTTCAGCCATATCGCGAACCATTTAAGTTTTAACAAAATGTGGGGTCATGCCAATGCTTTTGCACACGATTACGCGACCTATACAATGTCTTCAACTGAATCAAATCAAAATGCATAGTAATTTGAAAGCGAACCTCTCAGAATAACATTCCACCCGGGGCCTCATCTGACTTGAGTGCACTCACCAATACTGAAGAATCGGTCTCTAAAATAACTCCTGTCATTCTTGTTGCACATTATGCTTCCAGCGCAGCAATGCTTCTCCATCAATATTTACCTTCAGAACATCAGCAGGGGGAGGAGACCACCTCCCACAAGCATTTTTTTTTGGCTTCTTGTCCTTCTCAATTTTCTGCTCCGCTACACGCTTCAGCGAATGATCTCGTCTCCAGCATTTTGCTTTGTTCTTTGCACTCCACCAGCACCATAGAAATATGACGGTAAGTTGTAGATCCCCCTCCATCGCCAGAACATGGCCTACCACTTGCTGTGCACTACCAATCTCCATTCTCCAGTAATTTCAGTCGTATATTTTCGCGATATGCAAGTCCTGATTAATCACATCAACACGAGCCCTTCGCTTTGGCCCTTTAAAAGCAACGGCGATTAAACGAATGTACTTCACATAAAAACTTATGTTTACGTTGTGGCTGCCGCCTGCCGGTGGGTACTCCGGGCACCTTCACGAGACACGTACGGCCACATATCAATCAGCCTGTTCGGCAGTCTGCGGCTGCAGAAAAATAATTGCGAACACTGTAGCTGCTGCAGAAAAATAACTGAAACACTAAGCAGCCAGACAAACAAGCTGAATGACAGAATAAGAAAAGTACAAGAAAAAAAAGACAGAATGAGAAACAAAACAACTGACCATTCATACAGGGGCAATACAAGGATTTCTGCTGAAAAGAAAAACGCCACAGCGCAGGGGTATATACATGGATCAGTCGATAGTGGCAGCCGCAAGCATGTCCTCTCACCCACGGCCACCGTAGAATTACCAGCGGTGGACAAGAGCGCCACGAGGAGGACGACGACGCTGAGCAGTGAGCGGACGCCGACTTGTGTGCTTGCAGCCATGCTTATCTACTGGTCCATCACCTCACTCATTGAACATAGCTGAATCGTTAGCCAACAAAGTTTGAATACGAATGGTTGTTGTTTCCGCCAACTCATGATACTGAAGCTAGTTCGTGTGGCACACTATTACAAGTCCAAGGGTCAAAAGGGAGGGTTTCATGGCATTGTTCAGGTTCGCCGCATCTGTTTCAATGAGAGTTCCGCTCAATACCATAAGGATTAGCAAAGTTCTTCAATCGGTGTTCTCTTGTAGACAGGCCAGGAAGGCACCGCTCGCTGCTTGAACGTGACCTGCACCAGCTCCATGAAATCTCAATCGTGTTCCCGAGGAACAAAGTGAAAGCCCGGGACTCCCTACCCCTTTGTGATGTTTGATCAAATGAACCATCAGTGTTGTAATCGTCATACCACCAGGAGTTGGTTTCTTCCATGCTGCCTTTTCCCTGTTTTAAGTCTCGCAACATTGTAGCATGATAACAACATTGAGTTAATGACCGTCACCTGGTGACATTGCGAATTTCCACCAGAATCGATGCGTACGTTTTTTATTTTGAGCCAGTACGTTTTTCTTTTCCCTCTTTTTTTTATAAAGGAAGAATTGTGCAAAAATGTCCCTGAAGGGCCTACATGTAGGCAGAAACCAGCCCAACGAACCCTACGCCGTCTGAATGCCCGGCCCAAGAGAAATTGATCGGCCCATCGTAGCCTTGCCAACTTCCCCTCAAAAGTCTCGCACGCGCACAGGCACAGCATCGCCATCGTCATCCAAGGGGAAACCCCTCCTCGCGCCGCCCGCGCCGCCGAACCCTTCTCCTCGCGCCGCCCGCCCGCGCCGCCGACTACCACCACCGGACCGTGCACGTCGCCGGGGGAGGTTGTTTCTCCTAAACAATCCACTTTGTCTATTTTACCTCTTAATAAACAAAATGATGGCTCATTTTACCCCTCAAACAATTCTATTTGGTGAAATGTACACCTTTTTAAAGTTTGTCTTTTTTTATCTCAGCATACAAGTGGATTTTGAAATGTACATCTTAACATAGATTATTGTCGCAACTAATATCATGTAAAATTTGAGCTTCAAATTCCACTTGTAGAATGAGAAACAAAAGAAACAAACTTCCAATAAGGTGTACATTGCACTAAATAGAATTGTTTGGGGGGTAAAATGAGTCATCATTTTGTTTAGGGGGTAAAATAGACAAAATGGATTGTTTAGGGGAGTAAAATGGACGTTTTCGAAATAAAAACAAAAATGACAGAATGAGAAACAAAAAAACCGAGCATTCATACAGGGGCAATACGTTATACAAGGATTTCTGCTGAAAAGAAAACGCTACAGCGCAGGTATATGATGTAGGGATATTTGTATCATTGCCCCTCCTGTATGGATCAGTCGATAGTGGCAGCCGCAAACGCAAAAGCATGTGCTCGCACCCATGGCGACCGCCGTAGAATCACCGGTGGTGGACAAGAGCGCCACGAGGAGGACGACGACGCCGAGCAGGGACTGGACGCCGACTTGTGCTTGCAGCCATGCGCGCATAGCCACGCATGGTTATTATGATAATCTACTGGTCCATCACCTTACTCATTGAACATAGCTGAATCGTTAGCCAACAAAGTTTGAATACAAATAGTTGTTGTTTAATTTCCGCCAACTCATGATAGTTAAGCTAGTTCACGTGCCACACTATTACAAGTCCATGGGTCAAAACTACCACAAGGATTAGCAAAGTTCTTCAATCGGTGTTATCTTGTAGACACGCCTCGCTTCCGATTGCAGGAAGGCACCACTCGCTGCTTGAACGTGACCTGCACCAGCCCCCATGAAACCTCAATCGCGATCCCGACGAACAAAGCTGAAAGCTCCGACCTTCCCTTTGTGAAGTTTGATCAAATGAACCATCAGTGTTGTATTCGTCAGTCGGTTTCTTCCATGCTGCCTTTTCCCCGTTTTAAGTCTCGCAACATTGACGGCAGATTAATTAGTTATAGTTATATCAACATTTTAGCATAATAACCACATTGAGTTAATGACCGTCACGTGGTGCCATTTCGAATTTCCTCCAGAACCCATGCGTACGTTTTTTATTTTGAGCCAGTACATTTTTCCCCCTTTTTTTTGATTTAACGGAATGTGCAAAATGTCCCCGGGCCCATCAGAAACCGGCCCAACGAACCCTACGCCGTCTGAATGCACGGCCCGGGACCAATTGGGTCGGCCCATCGTAACCTTGCCAACTTCCCCACAAAAGTCTCGCACGCGCACAGCATCGCCATCGTCATCCAAGGGGAAACCCCTCCTCGCGCCGCCCGCGCCGCCGAACCCTTCTCCTCGCGCCGCCCGCCCGCGCCGCCGACTACCACCACCGGACCGTGCACGTCGCCGGGGGAGGTTGTTCCTTGTGGTTCTCGTCCTCCTCCTCTCTTCTCCTGTAATGGCGTCGAGGGGAGGCTACCACTCCGGGTTCAACGGTGAGCCCTAGCTTGCTCCGGATTTTTTTTTTTGTTCTAACGTTTTTACTCGTACTGCGGTCGATCGAGCCCTAGTCCTCTGTTCCCTGACGGATATATACTCCGATCCCTCTGGTTATTCGTTAGTTCGATTGCTACGCTGGGGTAATGAATACTCAGCTCTAATCGATACCCGTCAGTTCTATTCCGTTCTCCTTCTTCACCAAATTCACCGCAAGGTTTATCTCCCTAGTTCGCATTCACGCTATTTCAAAGAGCATGTACTTGAGCTCGGGCATATGTTGTAACCCTGACCAGGTTCACCACTATCAATTTATTTTGCAATAATTTCACCCTCCAGCTGCTCCTTGCCCTACATTGTGCCACTAGTGGATGCAAAAACAATTTCCTAGATGATATGCAAAAACATAAGATGAACAGAGTGGTGCTTGGTCTTGTGACTCTTGTCTGAATCTGAGAATGCTAGGCAGAAACATAAGGTGTATTTTACTCTGCTTTAATGACGGCATTGGTTGAGCAACTTTATGGGCAACTTTGCTAGGACCTTCAGTTGATTTCTTTATGGGCAACTTTGAGCACATTAGTAATCCTAACAATGTTCATGCTCTGCAGATTCTTTCCGTAATGTCACAAGGAGACATGACAATAATAAAGAGGTACAATTCTCAGTTGATGGGTCATAGTGCTCATGTGTTTCCCTTCATTGCCTAGACATTTAGCCTTCTCTGTAGTTGGGCTATAGTTTCAGATGCTTTTAACCAATGGTGCTTATCTTTCTGTAATTCTCAAGTTAAACAACAAGAGCCTCATAAATTTTCTAGAATTGGCCGTCGTTTCATAAAGCAAATTGACGCCAGCAGAAAAATGGGTTGTAAATTTTGTTATTTTGAATTTCATGTTCTAAATACCACCTGTTGGTATTGGTTGCATTTGCTAATAATAAATATACATGTTTTTCTTTTTCTTTTCAAACAGTTACATCAAAATGAACAAATGCTACTGTTTGTCCAATCTCTTGTATTCTTATATTTTTATCATTTATTAATGAGTTACTTTAGTTGATTATATATCTGCAATGTGAGTTTGTAACTACTATACGACATGTCTCATCTTATACCTAGATTATTTTATTATTATTTCAGAACACAAGTCAGCAAGGTTTCAATGCTAGGCAAACAGAAGTGGCAAGATCTGGAAGAGTTCCCAATAGATATACTAATAGTGGAGGCAACTCTCGAAGTTCATTGCCTGGTAAACCCCTCTATAATTGACATTGTACAGAATGGCATGACATGCTTCAGTTTCAGAATTGTAGTGATTGCAACCTTTTATTTGTACTATGTACTCTTTAGTTCATGACTCTTGTTTATGCTTTTGAGCATTTTGCATAAATCCAAATCATGCTGACATTGGTTGTATGAATTGTACCATCTTAGCTAAACCTTGGATATGAGGATATAACCATTGTCGCCCCTTCTAATTCTTCAGTTCATAGTTTCTCATGTGTTTCGTGGATTTCTGCTAGCATGTTCAGGGCAAATGCAGATGTCGCGTATTATTCTGTAGCACACCTCAGCACGATCAATATTGAACTGTTGATCATGACTTATGATTTTCAACTACCTAACCATATGGTGGCACGTTAAATTGCTTCTTTGCTCCTGCTTTTAGCCATCTGACCACTACTCATGCTTTCATTAATCACATTATGACATTATACAACACTGTTTTTCCTAGATAAACCGTTATGATATAAAGAACTATGAAAAATGGAACCTCTGTAACACTTAAGAACTTTTATGTAGGGTATGGCTACATATTTTTTGGGTCAGCAATCCTTCATTCTTATTTAAGCGCCTGAAAATAATTTAGGCAATAGTGCCTATCCTCTGCGGTTTAAGTCAGTGAAATAGGAAATTATTATGTTCAGAACTTCTCTATGTTGATCCAGATTTAAAAACTTTTGAACATCTGATACTTTCAGGAGTCATGCAAGAATATCGTATTGTGAAAGACAGCAGAACTAAACAGAAAGAAGCTAGTGGGACTGTGCCAGAAGTAGATAACAATGGAGAATCTTGCATTGAACATGCTGTTTCAAGCCTTGGTGGTAAAAGGTATCCCCTGTCCCTATTCACCTTTTTATTTACATACAAATAAAAAATGTCATATCATCTTAGGATAATCAGTCGTGGTAGCTTGCAATAGCAGTAGCATTTTGGCTTCTTAGTTCTTATGAAGTACTACTGTTTGTTCCCATATTCATGTAAGCTAACTGATTTATGTTCTATACACCCACACCCACACAATGGAAATAGCAATATAGCATGCATCCTTTACTTGGTTTTAGTATTTTCCAATGTATGTCACCTTTATGATATGATAGCTGCTTTAGTTTTCTATCTGTACTGAAATTTGCTGCATCATTTTCACCAGTATCACATGTGTTCATATATGTCATTGTCCTGTTTCTGAAGCTCAACTGAAAAGCTAGCTGCCCTGGACTCCTTGGTTACTGGAAATGGTAATGGTCATGGAGCAGCTCAAGCAGACAATGGTATTAAAAGTGTTGCTCTATCCCATGACAAAGAAGCCTCTAGTGTCAGAAAGATGGAACAATCTGGAGGGATGCAAACATTGGTTGGTCCACATGAAGTTCTGGGGAAGGACATCCAAAACACAGAGGTCACTGTGACGTCAGGAAAAAATAGTTTTGCTGGTGAACTTTGCTGTTCTTCGTCAGATCCAATACATGTACCCTCTCCTGGTTCTAAATCAGCTGGAACTTTTGGAGCCATAAAGCGTGAAGTTGGAGTAGTTGGTGCTAGGCATCGGTCTTCAGATAAACCAGGAACAAACACATCTACTTCACACGGCTTAGTCAAAGTGGCATCAACACCAAAGGATAATCCTTCAAATGAACAACAATCTGGACTACCTGGTTCCTCTCTGAAAAGTGGACGCCCTAATGTGCCTGTACCGTTGAACAATAGGCCATTTCCTTCAAGTCAATACCATCATAAGCCGCAGAACCATGTTAGTCAAACAAAAGGTTAGCATTTGGAACTCGATAACCTGAGTTATATTGATAACATCTAACTTTATTGCGAGTGTCATAAACAACTTTTTGTGGCACATAACTTATTTCTTTTCCTAAGACGCTACGAAAAAGCTTGGCAGTAAAAAACTACATGCAAATGAGTAAAGTCTGTTTAACTGCCGTCATAGTTTAACCATGCAAATGATTTTAGATTCTTGGTTTCAGTTGTTGGTCAATTCACAGAGGGATAGGCTGTGTTTCTTGATGTCTTTAGAAGATGTTTACATTCTTTTCTAGCACTTTGAAGCTAAAAAGGATACTGCTGAATTGTATGCAGCTAGTGCACATTTGGAGTGGAAGCCCAAATCAATAAGCCCCAGCTCTACCAATCATGAAGTTAGTCTTTCATCTTCTGGTGCTCGGTCACCTGTTGATGGTAATCAGGCGGAAGTGGCTGTCTTGTCGAAGAAGCTTTCGCAAGCGAATGTATCTGGAAATGAACAGGTCATCATACCTGAGCACATCCGGGTACCAGGCTCCAAAAGAACTCGTCTCATCTTCGGTACATTTGAATCTGGAGTATCGGATGCTTCTGGGACCACATCTGATACTGTTGTGACTAAAGAATGCTTGAAGGATCATTCTCCTTCGAGGTGATTCTACATTGATCCTGCTCCCTTTTTTGCGTAGCTGGAAACTGTAGCAGCTCCGGAGTTTCCTTTCAATGACTGTACCATGATTTTCTGATTTTAAAAGCTCTACGTGTTCAAACTGATAACTGGTGTGTGTTTGTTGTCTGCAGTCTAACAGCATTGAATGCCATGGTATTGACTGATATTTCTCCTGATGACAAGATGGATCATGCTGTTTCCCAGAGCTCACTTCCACAATCAGATTCGAATACATCAGTTTCTGAACATCAGAAGTCATCATCCAAAGCTGTGGAGGTTCCGAGTCCTGGTGTTGTTGGTGAGTATGTGACTCAGATGATTTCAAGCAAGGTCATGCACTCTCAACCTCAATTCCAACATCAGGACAATCAGGTTGTGCCAAACTTTAAGGTGACTCCTGACGACATTCCATTTACGTTTTATGCACCAGCTTTGGTGTTTTAACTGAGACTTTAACCCCTCCATTTCCTGATGTACCAGAAATATGAGCCAGATTCTAGATATGGGGTACCGTTTACCACTAAAGCCGTCGATGATGAAGCAGCCGAAAGTATAGCGTATCCATCCGAGGTAATGCTGACACGCTATATCATCTGGTGTACATTTGGTGTCTGATCTATCCTGCTAATGTTTCAACCATGGAGGACTTTTTTGTGCCAACTTAATTATACGTTTTGTTGTATTTTGATACTATTTCTCATCACTTGCACCTATTGCCATGAAACACCATATGGGAACAATGGATAGTGTACTTATATCCCTTGTTTCGGAAGTCTAGGACAGTATGCCTGTTCAGATTTTATATCAACATAGCTACTGGCTCCACATTGTTAGGATGCAGGTTTTGTTGTTAGTGCTTATCCATTATCCATGCCTACGTTCTATTGACAAGGCTGTATGACAATTATATGCGATTCTGTGAACAGGTCTTGGGCTTACATCCTGCAAACACCAATCAGTTACCTGCAGCAAATCAACAACCGGTGCCCCAGATGTATCCTCAACAGTTTCAAATACCTCAGTATCCAAACTTGCCATATCGGCATATGTACCCGCCACATTATGGGCCTCCTGTGGTTGTCCCAAGCTATTCAAGCAATCCTGCATTTCCCCAGTTGCCGCATGCAAGCAGCTATTTGGTAATGCCAAATGGAACCTCGCAGCTAGCAGCCAATGGCATGAAATGTGGATTACCCCATCAGTATAAGCAAGTGTTTCCGGGAACCCCTACTGGATATGGTGGTTATGCAAATCCTAACGTTGTTAGTGCTTATCCATTATCCATGCCTACGTTCTATTGACAAGGCTGTATGACAATTATATGCGATTCTGTGAACAGGTCTTGGGCTTACATCCTGCAAACACCAATCAGTTACCTGCAGCAAATCAACAACCGGTGCCCCAGATGTATCCTCAACAGTTTCAAATACCTCAGTATCCAAACTTGCCATATCGGCATATGTACCCGCCACATTATGGGCCTCCTGTGGTTGTCCCAAGCTATTCAAGCAATCCTGCATTTCCCCAGTTGCCGCATGCAAGCAGCTATTTGGTAATGCCAAATGGAACCTCACAGCTAGCAGCCAATGGCATGAAATATGGATTACCCCATCAGTATAAGCAAGTGTTTCCGGGAACCCCGACTGGATATGGTGTTTATGCAAATCCTAACAGCTACACTGTTAATGCTGGGGTAATTGGCAGTACAGGACATGTAGAGGATGTTAATATGAGTAAATACAAGGACAACAATCTCTACCCACCCAATCTACAGGTAGTGATATATTCTTTTATTTTTGCGGCACTTACTAATCGTTAGGCTTTTCTTCCCCATGACAATGAATTAGTTAATGTTCTTTTTGACAGGCTGAAACAGCAGACGTGTGGGTTCAGGGTCATAGGGAGATTCCCAACATGCCATCAGCCCCGTTCTACAGCATGATGGGACAACCAATGTCACCACATGCAGCGTACTTGCCGTCACAAAATAGTCACGCTGCTTTCACTCCAGCTCCTCCCCATCCTGGTCAGTTGCAATACCCGGGCTTTATCCACACCCTGCAGCCAACATCGATGACCATGGTGCAGAACCCACACGCCATGGTACATCAGGCCGCTGTGCCGCAATTGGCAGGAAATGTAGGTCTTGATATGGCAGCCATGGCTCCTGGAAGTCAGGTAGCAGCATTTCAGCAAAACCAATTTGGCCATCTCGGCTGGGCACCAACCTATTGAAAAGTTTGACATTCTTCCCTTGTGCGAGAAGTAAAATGATTCCTTGTGATTCGCAAATGCATAGCTGCTTGTACATTACTTGCCTTATACTATATACTATCTGTTACTCGTTTTCAGTTTTTTTTCCCTCCAATCCCAAAGTAAAGCACAATGTGCCCTTGATTGCTGACAATTGTTACTCGTTTCTGTTGCTCGTATGGTCGTTTTGAGATATTTTATTGCTATAATAAAGTAATCTTTGTCAGTAGCTACGAACTACTAGGAATGCTGCAAACAAGATCACACTGCAAAACAGAACCGGTCAATGACAAGTCACATTTAAACTGCATATATGGGCTTCCCGATGGTTGACGATTCGTAGGGGCATAATTTATCAAAAGCGGGATAATGCCTGTCAGAAGCCAGAAATAATGTTCAGTAAACAGGCTCCTACGGGAGATGATAATCTGATGTGCATTTTCGAACGTGTTCATATGTCATCGAATGTTATTCAACCTTTTCGTTAGTTATTTATTATTATAGCCCTTCCAAAGGGATAACCTTTTCTTCTAGGCGAGCAGGAATGTTATCTCCAACGATAAACTATAATGGTAGGTATAAAGTGGGTTGGTAGAGAATAAAGCTCTAGTTTGAAGACAACGACCTTGGTGGTGATTAAAATCTTGCTGTAATACAATGATGTGCAAACCCTGTTTATTTCTGTCTGGTTATGGATGGAGTCAGCAACGATTGATGTGCAGTTTTTTTTTTCTTTTTATTTTTTGCGGGTAGATGTACAGAAATTATATCGATCCATTTTTTTCTTTTTGCCAGTGAATCGATCAGATTGATTAAGAATACTTAGCTTGGAACATCGAAATCAAGCGCAGGGATAGAATAGTACTCCTATATTACCCCATGCAGTAACTTATTACAAGTCTGAGGTTTTATAATTAAGTTACAATAATAAGTCAAAATTTGCCCGGCACCAAATTGTGTCCAGAACAAGAAGCGGTGGCAGTAGTAGTCGTTTACGGCAATGAAATAAACATACAGATCCTTCAAGAACAAAAAAAGAGTAGTCGTTTTCATGCTCATCTTATCTTCTGCTGTAATTTAGTGAGCTACTACTCGTAAGTCTAATGAGGCGCTGAGTGCGATATAACCCTCAAGAGAAAGCTAAACTGCCTTTTTACTCTCAGGTCTCCACACACCATAATAAGCAAACAATCAGTTGGGTTGGGTATAGTTAGTTTGCCTTGGAGGTTCCATGAACACTTCAATTTTATATTATGCCTAGGATAATATATTTATAGTATGTTAAGCTAGATTATCCATTAAAAAAACACTGTCAATAGGTGCACTGATGATCCTTTCATACAAGCACGCATCTACCGTTTGTCGGGAATGGTAGTTACATCACTAAGCAAAAACATTCCAAACCGTTCCAACTAATAACTCGAGTCCATAGTCAAGACTGCTGGTACGAAAGGACCGAGGATCTACGTACCTTCGTCAATATATATTTCTTTCTATACCCACCAAATGGAGAGGACATGGTCCTGTCATTAGCTGCTCGTACTGATGACTAATGCGTGGCCAGCAATAATATGCGTGCACACGTTAGTTGAAAAGACACCTACACGACGCGACAAACTGAAACTCGAGTTTCAACTTTGTGTGTGGCATGTCACAGAAACGAAACTTCTCCTTCATTTCAATACCGAACCTTCGATTTTATAGAGGTGTTTCAACTTTCGTGTTTCGGGAATGTGCTTCCAATTTTCAAGTAAGTCGATCATTCTGGTTGTTTTTTCCATATTTCGAAAATGAACTGATATTTTCTCAAACAGGTCGAATTTTCCTTTCATTTATCCAGTGCCTTTTGAGTTGACAACAAAAGATTAAGAAGTTAAGCCTGATTTCTCTAATAAATGGACGCTATCAAACAGGTCTCATGGTTCCAAAGGCACGAAGTAAAAAATTTGAAGTGTTGAGCACATCAGATTCATTAGAAGTTCTCATGCATCGAAGCTTGTTCTCAAGATGTTGGCAGACAAGGTGTAGATGTCCTACCACTGCCTCACCGGATCGGTGGTCTCCACAACGTCATCCGTGGTCATAGCTACTCGCATCACCTACTACGCGACGATCGAAGCATCTGGAGCTATGATAGATAAAGTTCCACGCAGCAAATAATGGCTTCGAAAAACTGAATTGTTAATTATGTGACGATTTTTATATCAAATAGAGGCTTTATTAGGTATTAATAACATTACAGTGCAATCCACTGTCATCGATCATAGAGGCAACAAAATCAGGAACATGTTGTAGCCATCTCGAGAATGACCAACTTTATTTTGATCTCTACCACAATGTGTAATCTCCACTTCACGAAGCACAATGGATGATGCGACAATTTTAGCGCAGGATGGATCAATCAGAGTAGTTAAGGTCCGGGGAAATGGCTGAAGAGATCCGTTGGAAATGGATGGATACATGAATGTTTGTGACTTGAGAAAGAATAGAGATCCGTTGGCCATTTTCTCCGCCAGATCAGTGGATCGGTCGATCTATGACTAATAACTCATCAAAAAAAAATCATCTGAAAAGGCATGTGATGAGGGATACGTTCTCAAGATTTTCTGAGAGAAAAAAATAGTATGCACATGCCAGAAAGGAGTCTCATCTTATCCATCTTCCTGTCCAAGATTGTTAAAGAAGCGAGGGTACGTAGACGATGACTTTATTTGCTCTCCTTTTTGAGGTTGCCCAGATAGATCATTTTTTCTTTCTTGTTTGTTTTTACCCGAAAGCCCAGATAGATCACTGATCGAGGAGATCTCATACTTGAATATTGCACATGATAACCTTGATTCCCTCAGGTTTGTCTCGTTCTCTCTCGATCAATTAGCGTCTCATTACCTATCCCCCCCCCCCCCCACAAAAAACANNNNNNNNNNNNNNNNNNNNNNNNNNNNNNNNNNNNNNNNNNNNNNNNNNNNNNNNNNNNNNNNNNNNNNNNNNNNNNNNNNNNNNNNNNNNNNNNNNNNNNNNNNNNNNNNNNNNNNNNNNNNNNNNNNNNNNNNNNNNNNNNNNNNNNNNNNNNNNNNNNNNNNNNNNNNNNNNNNNNNNNNNNNNNNNNNNNNNNNNNNNNNNNNNNNNNNNNNNNNNNNNNNNNNNNNNNNNNNNNNNNNNNNNNNNNNNNNNNNNNNNNNNNNNNNNNNNNNNNNNNNNNNNNNNNNNNNNNNNNNNNNNNNNNNNNNNNNNNNNNNNNNNNTTTTTTAATATTTTTCCCCCCCCCCCCCCCCCAAAAAAAAAAAAAACAATTGTCTGTAATATGCACGGCCTCTTTTCTTCAATTATTGCGATGCAGCTATCCTAAAACCTGTGTTGTTTATTAGTCTATATTTTGTTCCTCTCGCTCCAATGTTCCGCGTTGCGTCATGTATAACAAGTATTCACTATTGATTCTTCAGTGAACTTTCCACTCAAAATTTTCGAATTCTTAACATTCTGTTAAAACTAGTTCAAAGCAGATTTAGATAGATTTAGCATGATTTCTGAATCTAATTCAACAATATTTGGATTTGCAGTGTTGGCGCCCCATAGTACATTCCTTGTTGGGTCCCTCCATTGCACCCACCAAAAGCATTGGTTATGAGTCATCGTCCCCACCGTTACGTGTTGTGCTTTAAGAAATCTTGGTGTCGCTGCCGATAATAACCTCCAACCTGCCCACGTTTGTGCCTTTGCGTGAGGACAATAAAATATTTGATCCCGGTTCTACCCTCCAACGAAATGCATGACAGAAATCTCAACTGCACCGCACTTTGCTACCTAAAATTATGGATTGGATGTACAAATTGCTTATGGAGACAATTTAAAATATTATAGTATTCGTACGAACTTGCCTTTAGACCAATTGATCTCTGTCTTATTTTGGCTAATGTGAGAAATACATGAACATATGGTTTGCTAACTTTATATGTTATCATCTGTCTAAATTATCATGTCCCCGCAAACTGATGTTATATATATTATATTTTTGCATGGACTATAATAAAAACTAAGCAGTTTTGGTGACTACACTCTTACTTGTTTGTTTGCACTTGTCCTCTTTGAAAGAATGCCATTTTAGATTTTAAAGGTGTATTAGTGGAAATAGTGTTGTACCAATCATGCCTTTAGAACAAACAATTCTTACCACTCATCATGTAAGAAATATTTATGGAGCAAGCATCCTTCAACCTTTCAAGGAAGTGTTTTTTTTAGAATTAACTAGATCATAGGCTTATTTTTCAACAGGGAACTGCATCTTGGTACAATGTAATTGACATAGTTGCATTCTACCAACATGAAGCACTCAAATCTATAAAGTGGTGTGTACGGGTTTCTTTGGATTAATTGAATTATAGAGGAAGAAGGGACAGAATAAAAAAAAAGAGCAAAAGATCGGAAATGGAGGATAGTTCCTCAACATGTTTGAAAAGGAACTATGACATAAACCATGTAAATTTAATATGTCTGATAATTTCATTTTGTTCTTTCTTGTCCTTGGTCTCACTTGTTAATGAAAGTTAACTCCTATACTTTTAGTATATAACAACCCATCATCTTTTAGTCATTTCATTTGGTTTCATTATGTTGGATTCCGGATACTAGAAAAGTCCAATCCTATTTTTTCTCGTTTCTACATTTTTATAATTTCTTACTCGGAAGAAGCCCTAAACTATGTCAACCAAATGGCTCACTGTGAGATTGATTCCAAAATGCAAAATTAGGTAACGGTTTATGCTAGTCATACTTTAATGGTGCTATAGCTTGCACTATGAATTGAAGGGGGGGGGGGCACTTTCGCATCTTTTTATTCAAAACTTAAAATTAGAAAATAAATTAGTATTTCTCGTGCGAAAGCGTCATTTGCTTGAAACTTCTCTATGACCGGGTTGCTTGGGAGCCATATATCGGTCCTATCCTGTGTGTACCCAACAATTTTGGGGTGGATGATGTATGGTAGGGTCCCGAGAGCGACAGATCATATTTGTTTAACAAAAAGATAAACCAAAAATGTCGAGTGTATATTTGGCTGCCGGGGGAGGGGCCTCGTCATGCTCGAGCAGCAGTAGCGTCATCTTTTTCCCGACGAGAGAGAGCCGAGAGGGAGAGAGAGGAGAGAGAGGGTCGATTCAGGGGAGGACGGAGAGGTACTTCTCTTCTGCTTCTACTTTAGCTACCTAAAATTTGCGGTTTCTTGCTGCCTCGCTCGCTGGATTACGTATTGGGGTGTCTGTGCTCTTGCTCAATTGCTACAGAGTTCGTGCGGACGCGGTTCTTGCTTCACTTTCGATCTGTCCCTTTGTTTTCTCAGCTGGATCTTCAGAAGTTTCTGCGAGTTATTTGCTGGAGAACACGTTCCTGGTTTTTTATCCATCCCTGCTTGTTTCAGCAGTCAGAACAACAACTAAATCTTTCTGGGAGTCAAAGCTTCCTCGGAAATCTTGACAACAGTTTTGCTGATTTGGGTTCTATTATGTGTTTTTCTTTTGTCAAAACAATCGAGTTAAATTTGGGTGTGTTGTACTGCTCTCGGGAGTGTTCTTTTTTCCTTCCCCTTCCAGATTTATTGCGTTGTTTTGCTATCAGAGAAATGGATTGCAAGTACAAGGACTTGGTGTCAATATTAGAATTTGCTTTAGCAGGCATACGTTCATTTGATTTGTGACAAGTATAATCTGGTTTGTGTAGTATCTCAAAATGGAACCAGAGAATGATGGAATTTCAATTGTTTTCTCCTGCTAAGTGTCAGTTTCTCAAAACTTTAAACAGTGATGATAAAACTAAATCATCAAGGAAAAGCAGGGTTTCCGTTTAGTACTTTCAAAACTAAAGATTTAGAATGATAATGTACCATCTTCTGCAGGCTAGACCGTACAGAGCAGGCATCACTTTAATTATTGAAACCAATCGTGTGGTAATCTAGAACTGCCGGTGCAGGCTTTGTTACTCTTTTTCCTTTTTCGGATTAGCACGGCAAATGATTCCACTCAGAAGCTCTGGTTATCCTGGAGCAGGCCTAGAGTACCGTTTATTTGATACAATTGATACTTCACCGTTTTCTTCCATATAAGTTAATGTTTCCATTGTTTTCCACTATTCATCTAACTGTTATGGTAATTGTTAACCAAATATGTAACTATCAAGAGTGAAAACCGATTCACCTTTCTGCATATTTACCTGTTACTTTGACATTGTTGGGTGCATAACTTTCTCTTCAGCTGATCACTAACAGATATCGAGAGTGAAAACCGATTCACGTTTCTGCATATTTACCTGTTACTTGGCATTGTTGGGCGCATAACTTTCATTTCAGCTGATCAATTATTGTGTGTCCGTGAATTTTCCTGAATCTTACTGTAATATTAAATACAGGAGGAAGCAAAATGATTCTGGAGCTACACTGGTAACAATTGATAACAATAATTCTTCTGCAAGATATGGATGGTTATCCAGTCCATTACATGCCCAACTATGTATGTATATGTTTGTTGTAGTACTGTTGTAGAACCTGCATTATCGAATGTACAGCTTTGGATGACCAGTTCTCTTCTGTGTGGATTAGGATTTCCTGTCTGGGAATGGTTATTCAATGAAACACTTAGCTCATAGCAACTCTGATGGGGACTGCTCATCAACAAGTTCTGAGCAGTCTCGCCAAGATGTTTCTGCAGTGAGCGACAGCAGTCTCAATGGACAGTACACACCGATACACACTGGTAATGTATTTCTGTAAACGCCATTACATCACGACAAACCTATTGTCTTATTTTGCTAACTAATTTGATGTTTCGTTAGTAAGAACATATTAAATGTGACCCTAGGTTTGCAGGTTATTATTGCTATGTATGTCAAACAATAACACACTATTGTACATGAAGTTGTCTTCAAACAAAGATTGGATTTAAATCTCTGTAATTCTGGCAACATTTGATATTTCACAAAGAAATTATGTTCAATCAACCAAATATTTTTGTCTGTGTTCTAAAAACCTCTTTCATAGAAACATTAATAATAGAGGCTATTCAAAATGGACCTGTTTCAGGGGACTGGAAGGTAGGGACTGATTGGCCTCTAGCCTAAAAATGTTTGCTGACGTAAACTTTTCAATTGTTCACATGGTGGAATATGAACTAAAACCCTAACAAAGAAAATGACCATGATAGCTTTTCAAAAAAAAATCGCGGCTATCAATATATCTGTTTTTTTCATAATGTAATTTGGTATAGTAAACAACATTTCAGAACATTTTTTTACCTATTGTACATTTTTCTAAAATGGATTATTACAGATTAGTCCTTGATTGATCATTGCACACATTTCACTTTGTTCTTTATGGCTCATTCTCTTCCAATTCTTCTTCTAAAGTAGAACCTTTCATGTAAGTTTCTATCATGTTAGTTGTTTATCAGCTATGGTATTAAGGAATCCGATTGAGATGATTTGATCCAAATAGTCATGACAGATTCTTACGGGCTACAGAAGAAAATACCTTTTGCATTAGAGTTAGTCATTTACAGGCTTTTATACCAGACTTGGACGCCTATAGGCGAGCGTTTGATAGACTCAATCAAAAGATTAGGGCCTATCAACTCAAGAAGATAATGCGAAACCAACCAGATAATTTAATATGCACTCCTTATTTAAATAATTTATTATTCAAGTTTTAAGGTGCACAAGTGGATGACAGAATAATCTTGTTTGAGCTCTATTGCACTGCATATCGTATAATGTATTTTGTACCTCCCAGACAATAATGATAATTGTGGAAAGCAAGACCAGGGCACGATAAAGTCTGTGTTGTCCTTGGGGAACCCAGAATCTGCCTTTTCGCCTCAGAAGTTTGATTACAGCCAGCCTTTGGTAAGTTAATTGAGCTTTTAATTTTTACTACTGCTGCCATATTGGAAATTACTATTTCAGTTAACCACCCTTTGTTTTTCAGGCTTGTGTTTCTTATCCATATGCTGCGGATTCATATTATGGTGGGATCTTAACAGGATACACTTCAAATGCCAATGTGTGTTTTCTCTATAGTTTATAGCTTTACAATACGGCTTTACAATGTTCATGAAGCATTATGCTCATCAACACGATAAACTTATACAACAAATATTATCCACCTGCTGATGATAGAACAACAACCTTTTGTAGGAAAACATGTAGCTTTAACCCTAGTCTTCTTGTGTGAAAATAATTTTATCAAGAATTTGTGTAAAAGAAAACCTTTTACACACACTCCAATTGTTTTGTGATTTGCGATTTTTTTTTTGACAAAACACCAAAAAAGTATAAACTTAGTTTCAAGCAACAAATGAAAATGAAAAATGAGTGAGCTAGTTAAATTTTAGTTTAACCCTAGAAAACAGTTTCTTATTGGTTAACCGAATTATGACCTTTGATTGTAAAATCTAGGAGAATATGTTGCCCTTTTCCATGGTTTATAATAAAAAAATACGCAAATATATGAATTTGTTTTCTGCATGCTAACATTTATGTTGTCATGCAGACTCATCCTAATGGTACAACAAACTCTAGAGTACCATTACCCGTTGAACCTGCGGCAGAAGAGCCCATATTTGTCAATGCAAAGCAATACCATGCAATCTTAAGGAGGAGGCAAGTGCGTGCAAAATTGGAGGCCCAGAATAAGCTGGTGAAAGCTCGGAAGGTAAGTCAGAACGGATCTCACATCTGTTGCTACCAGTTTTTCTCTCATTATTCACCATTTTAACATCAAATTTTGAGATTTACATTACACTTTCCTTTAGTGCAATCTACATCCAACCATGGGACTGTGTTGGTACAATTTGATTAGTATGTCATATTGTTTCTGCTCTCACCTTATGTGGGAATGTATCCTAGGATCTGTTACCACAATTTGATTCATAAACAAGAAATAGTAACCATATCCCATCCTCATTTGTTCCCTTGCCAGGTGTACACTTTTCACTCTACTTACAACTGGTGTGAAGTTTTTTTTTAAAAAAAATGTATGTACATTCCTTGGGGCGTGATTATCTGTGTATCATAGAAAATAATATTTTCTTCCACCCATGTGTCCTATAATGTCATGTTACTGAAAGCTGAGGTGCACTTTATATGCTTCACTTTCCATTTCTTGCGTCAAAAAAAAGAAAGAAACATTCAACCTGCTAGCTTTACCAGCATATGATCTATTGGCACTTGGCAGGCCACATATCAATTGGTATGGTACCATTCCCATTAATTCAGCCACATGGAATCAGCACGTTCACAATATCAATTTCTCTAACTAAACCGTCAGTCAGACTATTCACGCAACCATTAGGCTACAGGAATTTTTGGACGAAAGGCCACTTAGTTATGTAAAAGGGATGAGATGGAATTTAACACTAAGATGCCAAATATTTTAGTCCAGCAACCGTCATATCGTGACAATTGTACTGGAATCAATCAATTTAATTGACACATTACATTATCGAATACCATTGTACATAGTACCTCTTTCCTTAAAGCATCAATAACATTGTGTAGGCATGCATATGAATCTAGATACAGTAAATTTTCTTTCCAAATCTACCTTTTGTTTCAGCTTAGATGCGACAGATTTGATTCTTCCACACATGTATATACTTGGCCGTCTATGTTATAAAATTTTCCTGTGATATACTCAGCCTGGTACCTTTCCCCACAGCCGTATCTTCACGAATCCCGACATCGTCATGCAATGAAGCGAGCTCGTGGATCAGGAGGCCGGTTCCTCAACAAAAAGGAGCTCCAGGAGCAGCAGAAGGCACCGCCTTCACCTCAGACTCCAACTGGTGGAGTCAGCAAAGTGGGAAATGGCGGTAATCCATACACTGCAAACAGGACGTCTAAGTTGCCTGCGACACCGACCAGCTCTGGAATCTCAAGTGTTTCAAATGGGGGTGGCATGCTGGCACATCAAGGACACATCAGCTTCTCATCTACTAATTTTGTCCCCAGCATGAACTTCAGCGCACAGAACAGAGGTGAAAAGATGGCCGTCAATGGTGTCCCCTACCGCGCCTCCACGGTGAGGTGAGCAGCCTCCATTCTGGAGCGTTGGCATCATCGCTGGTCCACTCCTGTGTGCCCGTGGCAATTCATCCTTGGCTTATAAAGTATCTGTATTCGGTAATAATTTGCTTTGAGTTTCATATGGAGTAGTATCTGAGATCAACTCATTTATATAACCTACTTGCTGATATATTGTTGATACAAAAGGTCCATGATGTACCAGTACTTTGTCCATTACAAAAGTATTGACTTGAAAATGTAGAATTAATAATTTGTCATGGATTTGAAGAATCTGCTGATGACTCCCTTTGCTCTGTACGTTGCATAGCTCAATTTGAAATGGCAACACCTTTTTTTTTTCTGCCCACAGCCCGAAGATGCTATAGCTCGCCAAAATACAGAAAAAGTCGACAAGAGGTGGAATAGCCATATCGGCCCCCATCTTTAGCATAAGATTCAACTTCGTCCTCCAGCCGTCCAATCCAACCAGCCAATTTAGCCCTCAAACTTTCCCTGTTATAAGCATCCATCCATGTTGGCGTCATGATCCTTAAGCGGAATTAATTCCTGAGTCACCTCGTATCAGATGATGTTTGTTTTACCCTTATTTTTTCAACACAAGTAGAAAAAAAAAATTATGCAGCTCTTTCACACCTTCGATTCCCGTCAATTAAGATTCAGGCCACTCATAGCTATCAAGAAACACCTTTAGTAAAGGCAACATCCAATGAACACCACGAGCTGGCACCTCGAGTCCCCAATTTTTGCCATTATTCTCATTTTCAATTGTTTTTTAGGACTAAATCACACGGAAAATGACCCTATTGCCCCTGCCTTCAGTCTGGGTGAAGCTCACGGCGATCCCGCCGCCATGTTGCTCGACGTCGCCCGCCCCCTCCGTCGCTGCCCGCATCTCGTGTGCTGCTTCTGCGCGCACGCTCCAATGGGCCTCCCCGCTCACCCGGCGTGGCACACGACGTCAGGCTCTTGGTGCGACCTTGCCCGCCGCCGCCGTCGCTAAGCGCGAGAGGCAGGGCGCAATGTCTGGCGTGGTAGGATCGGGGCAGCGCGGTTCGACGCGGGATGACAAACAATATTGAGCCCTAATACAAAGATGTCTAGATCCGGGATAGTTGAGTCTTGAGACACAAAGTTGATGCCTGAGGCAAAAGACTAGGGACGAAAAAAGTCACTCTATCAAGAGAAGAGAGTACTTGCATAACACATTAACATGCTTTGCTGCCAGTATGGATCTGGAACTCTGCTTCCAAAAAAAAAAAGAAAAAGAAAAAGAAAAAAAGAGGATCTGGAACTCGATCATATGTACATCCTAGCCGATACAAACATTTGCTGGAAGCTACTCAAGAGGATTCATACACGTCAATAATTTAGTTCTACCATTTCATTTTCTAGTTCTTTGTGTTTTCTCTTGTAAAAAAAAATCACACATATATGCACACAATGGGAGTTAGAACTAACTGTTTACCAAGAGAACAGGAGATATTCACAAGCAAATGCAGGTCAACGCCATCCAAATACCACGTCAGTTGTCACTCTACAGAGGTTTCAAATCTTCACCTCTCGTGAGGTGAAAAGATGGCCGTCAATGGTGTCCCCTACCGCGCCTCCACGGTGAGGTGAGCAGCCTCCATTCTGGAGCGTTGGCATCATCGCTGGTCCACTCCTGTGTGCCCGTGGCAATTCATCCTTGGCTTATAAAGTATCTGTATTCGGTAATAATTTGCTTTGAGTTTCATATGGAGTAGTATCTGAGATCAACTCATTTATATAACCTACTTGCTGATATATTGTTGATACAAAAGGTCCATGATGTACCAGTACTTTGTCCATTACAAAAGTATTGACTTGAAAATGTAGAATTAATAATTTGTCATGGATTTGAAGAATCTGCTGATGACTCCCTTTGCTCTGTACGTTGCATAGCTCAATTTGAAATGGCAACACCTTTTTTTTTTCTGCCCACAGCCCGAAGATGCTATAGCTCGCCAAAATACAGAAAAAGTCGACAAGAGGTGGAATAGCCATATCGGCCCCCATCTTTAGCATAAGATTCAACTTCGTCCTCCAGCCGTCCAATCCAACCAGCCAATTTAGCCCTCAAACTTTCCCTGTTATAAGCATCCATCCATGTTGGCGTCATGATCCTTAAGCGGAATTAATTCCTGAGTCACCTCGTATCAGATGATGTTTGTTTTACCCTTATTTTTTCAACACAAGTAGAAAAAAAAAATTATGCAGCTCTTTCACACCTTCGATTCCCGTCAATTAAGATTCAGGCCACTCATAGCTATCAAGAAACACCTTTAGTAAAGGCAACATCCAATGAACACCACGAGCTGGCACCTCGAGTCCCCAATTTTTGCCATTATTCTCATTTTCAATTGTTTTTTAGGACTAAATCACACGGAAAATGACCCTATTGCCCCTGCCTTCAGTCTGGGTGAAGCTCACGGCGATCCCGCCGCCATGTTGCTCGACGTCGCCCGCCCCCTCCGTCGCTGCCCGCATCTCGTGTGCTGCTTCTGCGCGCACGCTCCAATGGGCCTCCCCGCTCACCCGGCGTGGCACACGACGTCAGGCTCTTGGTGCGACCTTGCCCGCCGCCGCCGTCGCTAAGCGCGAGAGGCAGGGCGCAATGTCTGGCGTGGTAGGATCGGGGCAGCGCGGTTCGACGCGGGATGACAAACAATATTGAGCCCTAATACAAAGATGTCTAGATCCGGGATAGTTGAGTCTTGAGACACAAAGTTGATGCCTGAGGCAAAAGACTAGGGACGAAAAAAGTCACTCTATCAAGAGAAGAGAGTACTTGCATAACACATTAACATGCTTTGCTGCCAGTATGGATCTGGAACTCTGCTTCCAAAAAAAAAAAGAAAAAGAAAAAGAAAAAAAGAGGATCTGGAACTCGATCATATGTACATCCTAGCCGATACAAACATTTGCTGGAAGCTACTCAAGAGGATTCATACACGTCAATAATTTAGTTCTACCATTTCATTTTCTAGTTCTTTGTGTTTTCTCTTGTAAAAAAAAATCACACATATATGCACACAATGGGAGTTAGAACTAACTGTTTACCAAGAGAACAGGAGATATTCACAAGCAAATGCAGGTCAACGCCATCCAAATACCACGTCAGTTGTCACTCTACAGAGGTTTCAAATCTTCACCTCTCGTACATCATTTTGTCCAAACGGAGTACCACAACCTGGACATTTCCGGTGACGAATCTCAAGGTTCCTCTGGATGCACGGTGAGCAGAATAGATGGAAACATTTGGTAATCACAACCTATGAAAGATCAAGGGGAGAAATATGATATTTGTCAGCGTAGGTAACAAAACTGGTCACGCGACACCTGGGAGATTAACCTGTACGATACATTACACATTACCTCTTTAGGCCGGTCAAAGCAGACACCACACTTGAGAATAGCCTTGCATTCTTTTATTTCATCCTGGAGCTTCTGGATAGTAGTCTCTTCGGACTCAGATGTCAGCTCCATGACTTCCTTTTCTACCTCTACATATTGTTCCTCAAGCTTCCTCCTCTCGCTTCTGGAATATTAAAAATGCGATAAGCTCCTGGTGGCAGTCCAGCAGAAGATCAAGAATAACATGGAAATAGTTAAAAAAAAACAGCAGTCTGACCTCTCGTGTTCTAGCAACATTCTCAGCTCAGCTATCTTTTGCTGCGTTTGCTCGTATTCTTTCTCAGAGGATCCGACAGAGGACCGAAGCCACTTGAGCTCCTTCTCCGTATTGGATACCTCCAATGCAGTCCTGTCCAGGCTAATTGTAAGATGCCTGTTATCCGCTGATGTTTTTATAGCTTGTGCTACACAAGCCTTCATCTGTAAAAATCGTTCAAGTAAGCCAATTTAAACTCGCACTTCCAAGCATACGAATAAGTTACTTTATGCAATTACTTCCTTTTCTTTTAAGTTAACATCTTTCTAGAACTGTGGCACACAGTTTCAGTGACTGCAAGTTATCAACAAACAATACTATATATGAAAGAATTGCAACAGAAGAAGACTACCTGATCTTCACCACGGGCAATTTTTAGTTTATATGACTCCAGTGAAGTATTAACTTGCTGGAGTTGCTGCTGTAACATAAGCTTTTCAGAGAGAAGGGAACTAGAAGCTTGTTTCATCTTCACACTATCTGATACCAGCTGTTAGATAAGAATTGAGTTACTTATATAATAATAAATAAGAATACTAAATAGAGTTAGTTTTCGGCAATGCGAAACCACAATAAACTTAAGTGTGACAACTTCGAGCAACACGTGGATTGATAGACACCCTGATATAACAAAATATTCACAACTCAAGTTCTGTCTATGTCACTACAACATTATGCATATTGATCATAAAAGATAAAACCCACAAAACTGGATTCATCCATGCCTGCCAGCAGATTACACTGCATTGCATATCAAAATTTCGTTTATGGTTCATGTAATATAATGGAAATTTAACAACATCGAAACACTAAGGTTGCCTGCCAATCAGTAAATGTTTTTTTCATTCAAATCAAGCAGTATTTTTTATGATCATTAGCATCAAATACTCCAGACATCTATGTACTATACATGTATTAAACAAGCAAGTCCAGACATTACAGTATCAGTCTCGTCGCAAAAAAAGACGAAATGAATAGCTAATACCTTTATGTTGAAGTCATCTCTATCAGAAAGCTGTTGAAGAAGATGCTGATTCTGTGTTTGCATGTCTTCATATGCTTGACCAATTGTCTGGAATGCAAATAACATATTCTTTTAGGCAACAAGTTGGGGTAGGCAAAAGATGAAAGCCAAAGAGATACTAGTGACAAGGATAAATATATAACAGGGTTTGTATTAGCAAAACACATATATCATACATACAAACAAAAGCTACTGATCTGAGCTAACCTCGATTTCAGAAATATAGGCATCTCCTTCAGCCTCTTTAATTCTTATTGCCTCCTTGAGTTCCAGAACATCTCTGACAAACACATCATGAATTCGACAGAAAGTGTGGCTTAGATAAAGATCATCAGTTTCTCATTATAAACAACCTAATATTTGCTGGTTCACAGCTTTTATATTATCATATTATGTCTTCCACTTTAAAATGCAAAAGCACTGATCCAATATAACAGCTCAAATATACTATACACAGCAAGCTGGGTACTACATACAACTTGATAAAAGCATGTTTTTTTTGGTCACATAACTGGATTATAGAAATGAGCAGCCACTAATTGTTAGACGCATCATATTTCTCAAGAGCAGATAGACAACCTTTCAGATGCATCAACTTTGGCCCTCAACTCTTCTAGTTCTGCTTCAGCAATTGAAAGCCTTTGCTGGCATGCAGCTTCAGCTTCATTTGCTGCTTTTACTCGAAGCTCTAGGTTATGCTCCTCGAGATTACTTCTCAAATATTCAGCTTGACTGCGAGCCCGGTTTTCTGATTCTTCAATTTCCGCAATTGTCCTAGATGCAAAATGGTGGAGTGACAATGATAAATTATAATGAAATGAAATGAGAAGTTTATGTCAAAAGAGTCAAAAGACTACAGCACATGCATAGACTTCCTCAGTCATCTACTGTTAGCCCTTCTTTTCTGATAGATATTTCGAGGCATATGTCAACATGATTCCAGGCCAAATCAACATACATCACATTTCCTCAACAGCTATAAAAATGTTGGATCAGGTCATGATTATATGTACATTAGATTCAGAAATGAAATTAATTTGCGAATGAGCATAAATATCTTAAGTAAAAACTCTATAATATTACTTTGAAGAATCAACTCTAAAATCCTAAAGTGACAGCACACCAACATTTTTATATACTATCAAACAAAACCTGAGGTCTGGTGTATAAAGCAGATTGGACATCTGCTAATTCTTTTTTTTTCTAGCAGTTGATCAGAAGAAAATTAGAGAAGCCAAAACATAAATTTCCAGAAAGAATCTGGTTATGAGGCCAACATGACAATGCAAATGCACGTCTGTTAAACATCTGAAATCGATGAAGAAGATGAATTCAAGTTATACCTTGACTCAGAACATTCTTTCGCATACATATCCGCAATAAATTGCAGCTCCTGCTTTTCCCGCTCCAACTCCTCAATCTATTGAGATCTCATTTACAAAATATGAATGAGAAACCAGTGACATCTTTTAATTGCATCTCCATTTGCAACTTCATTTTAACCATAACTGGGCTGGCAATTTGCATTACGTAAATGCTAAGTACACTTAACCTCCGCAAAATGTGAAAAAAAAAAATCAAATATGATAATGACCAGCCAATCCAGCATTAGAGAGATCATTCCACCCTTATTTACTTCACACAAAAATGAAATAGTTCAATATGGCCATGTAGGCCATAAGAAAAGAACAATCTTAGCTAGCTAATTGTAATAAGCACAATATAAGGCTCTAGATCCTACTTTTTTTCTACTTAATATAAATGATATGCAGCTCTCCTGCATGTAAGAAAAAAAATATGCTCAATATAAATCATCTTGAAAGACCAAGTGCTATGTTAATCCATAATATAGTATTTAGTATGCTAACAAAAATATTATGTCTAGTGTTATCTAATTGATACAAGTTTCACAACAAGCTGAAAAAGTAACGAAATACATTTTCTCAGGGGATATGAAATGGATGTTCAACTTCTCAGGCACTCAACTAATGAAAATTGAAAACTTGTAGAATAGCATACCAGTGTTTTGAGGGACTTTATCTCAATGACTTGTGAGTTGTATTCATCAGATACCGCCTTTTGTTCACCAATCTGAAAATTACACATATTTTCACCAGATGTAATAGATGTTTTGCCAGCCACTTATCGTTTTTCAGAAAAGAACTGAAGCATATAGCAACCTAATACAGAATACAAGCTCTAGTGCTCTACTGAAATTAATTAACCTGCAACTGCACATGATGGCATGGAAGTCTATCCCACAATGATCCCACATAAAATTCATCTTTGGCATGAACAGCTTGACATTTTTAAAAGTTCAAAACTACAGATCCATATTTAAAATGGTGCTGCTCAGATGAGATCCACGCCAAGATATTAAGTTCAACATCCTGTTTAACACAATCATCGTGCTCTAACTATGCATATACTGATGGCTGTTCTAATCTGAGAAAACTGCTTTCAACACAGATGATCATATTCTGAAATCAGCCATTCATGTGTAGAAAAATCAACAAGAATGAACGAATTAAGGTGACTGTTGATTGGATATACAGAAAGATAAACGAGGAAAAACATTTCTCACGGAGAAACCAAGGGAGTTCCAAAATCATGATACATGACATGATACGGTAATAAGAACCAGGATGTGTTTTCAGCGGGGGTGGTGGTGGGTGGGCTAGAACACCACTACCCAGGTTTGCCACCAAGAAAGAAAGGGTTAGGACCACCCATGACATTGAAAGATGTTACAAGGAATAATTAAGCACAGAAGTAAATCATTAGTGTGTGTGAAGCAGCTTCCAACTTGGTAAGCTCTAAAACAAAATCCACAAGGATGACAAAGAATATTGTATAATAATTCTGGCAAATGCAGGAGTAATGAATAATCAGATCGAAGACAGACCTTCTTAGCTACCAAGGTTCTTAAAGTATTAGCTTCCTCACGTAATGCAAGCGCTTCAGAAGCCGCGTCCTTTGATCTATTTAACTGATTCTCCATCATTTCTATCTCTTTGGAGAGTGCTGCAGCCATAACGTGAATCTCATCCTTAAAGTCTTTTTTACCTAAACACGGGAAACACGAAAAATGTATATTACATCCCGCAAGAAGCATTATCTCTCCTAAACAAGTTATCGATGTGCTAGGTCCTTTACCTGAATCTTGCAAGGTTTCTTCAACCTTGATTTCAAGATCATTCTTTTCAGCCATTAATATCTGAACTTGGTGTTCCAATTCTTCAATTTTGGTTTCATAACTGGTAATGTTAAGTCTGATGTTGTCAATTGATTCTGCTTTTGCACAAATTTCGTTCTCCTTTTGTATAAGCTGGTCCCTTTCATTCTAAACACAGAGAAAGAAAAAGCTAAGTGACCATATATATATAACCCAAGGCAGTACATCAGAGGACAAACATCACATCATACCTGTAATACTTCAACTAACCCCCTGTAGCGCTCTATCTCAGCATTCAGATGCTGTAACTGATCATTAAGAATCACATATGGTTTTGATACAAAAACATAGTTATCATCTTTCAATTGTATCTGCATACATAGCACAAAGAAGTCAGGAAACTAGGATTCAGCTTCAAATTGCAAAATGTGACAACCATTTACTACAATTCGATGTTCCAACCTCAAGATCTCCCAGTTCCTTGGATAATATCAGATTATCCTCTTGAGTCTCATGGAGTTCAAATAGACGGTTTGATGCGAGTGTCTGCCATCAACCGGGAAGATTAAGTGGTAAAAGATCAACACACATGATCGAGCATACAACATATCGATGAAGAGTATTCAAGGGAAGAAAGTTGCAAATTGTTCTGATTACAGATTTAAAGTCCACACCTTAGCCTCCTCAACAGCATCTTTTAGGTCTTGCCAGCTCATACTTTTGTCTGAAGATTTATCAGTAGAAACACCACCATTGACAGCGTTCGCAGAAGATGCATCCATTAGCGAACCATGCCTCTGCAGCTGAAGCACAACCAACTTCCGTTGGCTTTCCTCGAGCTCTGCTATGCTTTCCTCCAATTCACCTACAATCCAATGTATACATTATTTGGTTTATCGACCAGGTTAGCAGAACTTTTATAGCCCAAACATTTTTATCCAGGCTTAACAATTTGGTGTGCATTTATTTTGTGCCAATGCATGTGTAACCGAATATTAGTACAACTAGAAACCGAATGCCTTAGATTCTCAAATAAGCATGCAAAACATGACATAAGGTCGAACGCAAAACCTGGATTTATTTCACACAATAAGGCAACTTTTAATTATACAACAACATAAATTGTTTGGTATTTCCAGTTAGCAACAGAAATTATTTCAAGAACTCTGGACGGCATATATTAAATGCATGTACAGCTAATTTCATACAAACCAAAGGTAAGTGGGGCAAGGATGAGGGGGATGGACATTTTCAAACCTGAAAGCCGCTTAATTTCCTGTAGCTCCATTGAATGTTTGCTTTTAAAAGTCTCAATCTCATCCAAATACCTTTTGTGCTTCTCATTTATAATGGAGATAGCTTGAGTGGCATTATCAACCACCTCTTTTAAGTGATCGTTATGGTTTTGAAGGACAACAATAACATCTAAAAAAAGTTGAGACAAATGGGTGATTGGTAAGTAATATAGCTGCAGTGCAAATAAGGGAAAAAGTCCAGCATTCCCATTTTATTTACCTTCATTTGACTTTTGCCCTTTTAAAGCTAGAGATAAATATTCACTTCTAGCCTTCTGTGAAGCAATGGCCTCTTGCAAGGACTTCATCAGGGTAACAGTTGCTGAACGCCGAAAAGCGAGATTTTCTTCAACAATTTGTAATAAGCTAGTATCATTATTATTTTTGAGATTGGCGGACTTCAAGAGCCTGAAGAGAAATATCTCTTCAGAAGGACATGACTCAATAAAATCTGAGGAATAATGAGTGCAAAATCAGCTGAGAGAAACATCGCCGGTAAAATAAAATGGACTAATAAGCACTACTAAACCTTCTGATAGCTCGTCATGGTCAAGCGAATGCAAATTATCCAAATTCCCACCAGCACGTACCCCAAGAAGGACCAAATCGTCAATCAGCTATAATTTACATGTGCATAATGAGAAACATGTCAAACCGGAAAAGATTTATACAGCAACACGTATATTAGACCCGCAGGGCAGAACCTGATTCCACATTTTATTCAGAGAAATCAGAGTCTTGTCATAAGAACATTGCTCGTCCCTCAGTGCCTTGAATTTGCCTTCCAATGTATGCATTTCAGCCTTCTGCGCCTCCAACTGTTGTACCAACTTTTGGTTTTCATACTGAAGAGCAGCGGCATCCATCTATAATAAAAACAGAAATAAATAAATGATTGGACTGAAAATGCAAAAAAGTTGGATCTTAACAGCAAAGTATAGCGGACACCATAAGAAACTCGTAAAATTCACCAACCGAACTTTCACAGTTAGGTAACCTAATAAGAGCAGATACTCAGTTGCGGTTGCCTTGCAGGTTGTTTTGTGCAAATTATAACAACAACAACAACAACAACAACAACAACAACAACAACAACAACAACAACAACAACAACAACAACAACAACAACAACAACAACAACAACAACAACAACAACAACAACAACAACAACAACAACAACAACAACAACAACAACAACAACAACAACAACAACAACAACAACAACAACAACAACAACAACAACAACAACAACAACAACAACAACAACAACAACAACAACAACAACAACAACAACAACAACAACAACAACAACAACAACAACAACAACAACAACAACAACAACAACAACAACAACAACAACAACAACAACAACAACAACAACAACAACAACATAGCCTTTTGTCCCAAGCTAGTTGGGGTAGGCTAGAGATGAAACCCACAAGAAACAAAGACAAGAGAACACAAAGACAGAGGGAAAAACGTAGATTTAGAGGTAAACAGAGAAAGAACTTTCGTGCAAATTATAATTAAACTCAAATTCTATGGTGGCATGGTATTATAACTTACGATCTGTTTGGTTTGTGACACAAGTGGACCAAACTAAAACCTCTATCAATGCCAAAACAAGGACAAGAGAAAGATAATTTCATTGTAGCCATACCAATTTACAGGCAACATTGGGAAAATAGTCTTATTGCATACGTAGTACCGCGCAGGACGCGCGCACTGCTGCCATTGAGCAGGCCACCGTCGGCCTGCCCAAGCCGAGTACCCAGCAGCGTCTACAGACCAATCCTGATTCGGAGGAGGCGCAGAGGATATACCCAGTACAGAAATGAGTGGAGCAACCGATACCGGCCAGTACTGGATTGAGGCCCTTCTGTACCGCTCAAGTCACTGGTAGCCGGTACCAGAACGGTATTCATAAAAAAAAATCGTATCGAATTCTGCAAAAAATGGCGGGTATGATGCATATGGCCCAACGATAATATTTTTTTAGCATCATAGCATACCCAATAGGGATGTTGATTTATTTGTCAACCGTCACTCAAAGCTATTACGATAAACTGTTACAAAAGTCTCCAACTATGGGTCGTGTTATTACAATGTACGGAATTCAAACCAGTCATTTTTTTGAATAGGAGAGGATTAGCAACAATGCCATTGAGTCCTAGACCATGTTTTTGAGGCGTTGCGGCGTCCTGTGGCAACCCACCCTTGAGGCGATGCGGCGTCCTGTGGCGTCCCGTGGCGACCCACCCCCGCCACAACGCCTAGGCGTCGCCTCAAAAACATGGGTCCTAGATGAACCATATTTTGGACAACATTGCTCGCTTTCGTTTTTCTTCTGTTTTCATACTGTTTCTGGAGGCAGTGCGCTAGTTAGATTGTATGGCAGAACCACAGGTATAGATGCATAATATGAAGTCGATGTCTGGGTCCTTTCCATTCTAGTTCTCCTTATTCTAAACAAATCATACTACTATACATTCCTGCTACAAAATGCGACCTGTATGAACCAAACTAGTTTTCGAGTTCGGTCAAATTGTACTACTGCCATAAGGGATGAGGATTAGGGAGAAGGGGAGAAAGAGCCAAAGAGCAACCTGATTGCTTCAGCTAGGCAGGCAGTCCCGCGTGGGCTCCGACCTCCAGAAGACCAGGTCCAGCTGCAGGCCGACAGGAGGCAGCCGCGGTCGGCACTCCAGCAGGCAGGTTGCAGGCGCCGCCGCACCACTACCTTGGTCACGCCGGCGAAGAAGTCCTGACAGCGCACGGCCGCACAGGTCGAAGGAGCGCAGTCGACCAGGCCGCTGGGATAGTACGTCAAGGATGCAACGCCGAAGGGCCCTAAGCTGCGGCGGACGGCGGCTAGGGTTGGGCGGACAGCGCGCAGGCGACAGCTCGAGTGAGGGCAGGAGGCGACAGGAGAGCGGGCGTGAGGGAGAGACGTCCAAACGGGAAAGACTTTATGGGGCTTGGGCGACAGTCGGGTTGCTCGGGGAAGAGTCGGCCGGCGGCGGCGGCGGCCTCGTCGGAGCAACGGCGAGAGAGAGGGAAGGAGAAAAGAGAGAGCTGATTTACAGTGTCACCAGCTGCTTCGAAAACAACTAAACAAGTCGTCTCGGCCCACCAGCCCACAGTGATCGTACGGGCCTGATTTATTCGTGGGCACAAAAGGCCGGGAAAAGGAGCGGACGGGTCAGGCCCAAGTTAAGCAGCTCCCGTTAACCTAGCCATACGTATCAGGTTTATCTGAAAAGAAAAAGGAAAAGATTATCTAAAAAAATTACGTATCAGGTTCTATAATAAGAAAACCAAAAGAAGGAAATAAAAAATACAAACCCTATCCATACCGTATCCGACTGTGACAACGAACCCATAAGTATATTTTGCTTACCCATCCGTCGCGAATCCCTCCTCTGCCGTCAACCGCAACAATTAGATTTTGCGTTCGAATGACCTCTCTTAGACTGTCCACCTTCTTACAGGACGGAGCGCTTCGCGCGGCTCGCGTCAGTCGCGGTGTAGCCGTGGGCCCCAACAGCTTCAAGAGTTTCACACTTTCAAACCGATTTTCCGAATTACGAACTGATTATACCATTGCGTTTATTGCGACGAAATCTTCAAATCTAGACCCCACTTGATTATGTTTTAATTTATTTTTTTAAAAAAACAACGAATCAAAAAATTGGAGCAACAAATGAAAAAGTTGGACAACAAATAAAAATATAGAGCAACGAACGAAGAAAGTTGGACAAAACAAATAAAAATGTTGAACACCAAATAAAAAATTAGACAATTAAATAGAAACGTTAGAGCGTCAATTGAAAAAGTTGGCCAGCAAAACAAAAAATGCAGGGTTTATTTTATTTTTTTTCTTTTGCTCTCAGGGTACTCATAGTAAACACATGGGATGAGATACTTTGTGATTGATAAATACTCTGCTGTCAAAAGCAAGAGGAAATTTGTGCCAGTTTTTTCTTTTCATCGATAAACATAGTAAAGAATGCCGGTAGTAAAATATGCAGGGAATAAGACGGATCATCTAATCGATCTGGGATGAACGTACGTTATGGTCCCATGTTGGCAATGTATGACGTATCTTGTCGCGAAGACAATACATGTTGGCATTGTTGCTAAACACTACGTACCCCATGAAAACGAACTGAATACACACACATACACAAAAAAAAAGAAGTTTAGTGTCGCTCGCAAAGTGTCAGATCTAATGAACGAGGCTCTTTGTGCGGATTTCACCAAAGATGAGGTGGAAAGGGTTTTATTCCTAATGGGGCCTAATAAGGCGTCAGGCCCGATGGCTTTACAACGGGATTCTTCCAAAGACATTGGGATCTTGTTAAGAAAGATGTATGCACAACAATACTTAGCTTTCTCCAGGGTGGTGATATGCCCTCGACGATCAACAACACGATCCTAATGCTGATCCAGAAAGTCAAGAATCCACAGGAGATGACACATTTCAGGCCTATAGCACTATGTAATGTGATGTATAAAATCTGCTCTAAGGTTCTAGCTAACAGGCTAAGGCCTTTTATGGATTCTATTATATGAAGAACAAAGTGCTTTCGCCCCTGGACGTTTGATCACTGATAATGTGTTGGTTGCTTATGAAAATGTGCACTACTTGAAGAGAAAGAAAGGAAAATCAGGGGCTTGTGCTATTAAAATTAATACGGCAAAAGCCTACGACAGAGTTGAGTGGTCCTATCTACGTGCAATAATGCTAAAACTTGCTTTTCTGTCAGAGTTAATGAAAAACTATCTCATATCTTCACCCTAACACCAGGTATTCCACAAGGAGACCCCATATCTCCTTATCTGTTCTTATTGTGTGCTAGGGGCTTTCAAGTTTACTTAAATTCTCAGGGCCGCAATATCTTGCCAGGGGAATTCGAGTGAGGGTTCATGCATCTTGGATTTCTCACCTACTATTTGCAGAAGATTGCTTTATATTCACACAGGCTAGTGACAGAGGAGCACACAGGCTAATGGAGGTTTTACAAGCTTACCAAAGAGGCTCAAGTCAGATGGTGAATGTTAATAAATCAGCTATTTTCTTTGGTGGCAATTACAGTGATTCTACAAAACAAGAAGTGATGCAGATCACAAACATAACCACAGAAGCTCTTGGAGAAAAGTACTTGGGACTAAAGACTGCAGTTGGCAGGAGCACAAAGGACGCCTTTGAGCATATTCCAACTATTATCAGAGGCGTGGTTGGAGGATGGGGGGAGAAGCTCTTGAGCTGTGCAGCATAGGAGACATTGATCAAGACCGTAGCACAAGCAATCCCGACCTATTTGATGAGTTGTTTCTTATTGTCTCCAGAAACTTGTAAGAAGATCACAAGCACCATCTCAATTTACTGGTGGGGTAGCGCTATTGACCGCAAGGGGGTTCATTGGCGGAAGTGGTACGATCTTACGTTGTTGAAGAGTTATGGCGGCGTGGGATTCCGAGACATCAAGCAATTCAATATCGCCATGCTTGGAAAACAAGGTTGGCGCCTCATGACTAACCCAGAGTCACTGTGTGCTCGGGTGTTGAAGGGTAAATATTTTCCACATGGAGATTTCCTATCAGCCCAGAAAAAGGAAGAATTCATCCGATACGTGTCGTGCTATTTTGAAAGGTAGGACAACTCTGAATCTTGCCTAATCCGAAGAGTTGGTGATGGGTCATGCATGAATATTTGGAATGATTTTTGGATCCCTGGAGGTATTGGTGGCAAACCCATTTGAAAGAAGGAAGGGGCGACTGCAACATGGGTTAGCGAACTAATTTCTGCTAACTCCTGTACCTGGAATATTGCTGCTCTTGAGAATAATCTGATTTCGTCAAATGTTGATGCTGTTAATTGCATTTCTTTAGGCAGATGTTCTGAAGATTTTTGGACATGGTCCTGGGAGAAGCATGGGACTTACTCGGTGCGTTCAGCTTATAGGCTTCTGGCATTGAGAGATGCGCAGAATAGAGCCCACACGACTAGCTGTGCGACTACTTTGAAGGCAGATACAGATACTTACTGGAAGGTTTTGTGGAAAATCAAAGTGCCACTGAAAGTTAGGGTGTTTTGGTGGAGAGTTATAAATGAATACATTCCATGCAAGCCAACCTTCACTGCAGACATATTGAGCAGCAAGGGAATTGTATAACATGTGGTGCTAAATCTGAATCAACCTTCCATGCTTTGGTCGAATGTAACCATGCACGGGCATTCTGGTTGGAGCTGAAGGACATGACATAAGTTTAACTGCCCTCTCTTCATCCACTGACATGGGCACGAGACTTGTTGGATGCAAACATTTGTCCAAGAAAGAACGCTACCATCATATTATGTGGAATGTGGTCGCTATTGTGTTTGCGTAATGATAGGAAGCACGGAAAAAATGTGAATCCAATAAAAAAAGCTATAACATGAGCTCTTGACACTTCAGTACATCTTCTCCATGTCAAGAATACAAGTGTTAAGTCTCACTCAGTGGACACTGCTATGCATATATGGACAACCCCTCCAGTAGGTTTGTAGGTGCCATTAAAATCAACTCCGATGCTTCTTTTTACTACTAGCATTTTGCCCGTGCGTTGCAACGGGTGTTGAAATTTTTATTGTTTTAAATTTAATAATAAAAGCAATTCATTTAGGTAGCACAAATTCATCTAACAATGTCGATTTTTTTGGACTTTTACAGCACAAATTATCTTCTTTGATGTGATGTGTTTCTAGAAATAAATACATCAACAATAACTTGCAAATAATAAATAACCGGATATGTGTGCATGCCATATTTCAAGGTTTTTCTATGCATAGAAATGCATATATGCATTTATCTTTGTTTTCATTATCAAAAAATTTGTAAAACTATCTACTCAAAATCATACGGGTTTGATTGTACCTTTTGGTAGATCTATTCACATTGATTCTAACAAAATTTGGTTCATATTTTTCCGAGATATATATATATATTTTTTTTATATAAATTTTACAAATTTTTGGCTAAAAGAAAAAAAAAAGCTCGTTGTACATGCTCCTAGGATTCGAACATGTGTCTTTAGGGAGGCCCCACGCACTAACCATTACAGCACACCGTTCTTTTTGAACAAAAGCGATTCGGCTTTTATAAGATGAAAGCGGATTTTATTTTTTGGAATTCGTTTTGAATTTGACACAAATTTAAATGGCAGAGGTCTGAAAATTCAAGGCTGACGACCCTCTTTAGACTTATTAAGTAGTAGAGATAGTGATCAAACATGGGCCTTTGGTGTAGTGGCTCGTGATGAGAAGGGTCAGTTCCCGAACGGTTGCCTGTTGTATGTCACCACTTACTGCGGAGGCTGAAGCTTGTCGAGCTGGAGTGCAACTGCTCTCTGGAAATTCAGTTGCTAAGGTGATCACGGAGACTGATTCCAAAACCTTGGCGGAGCTTTGGCGGAATAGAAACTTGAATAATAGGTCAGATATTGCTATGGATCCTGACAGATATACCAGAGCTATGTAATGTTTTCTCTTCTTTTGATATAGTCTTTCTCAAGCGAGTGCAAACTGGGCAGCTTATCTATGTGCCCAGCACGCTATGCATCATAGGGCCTTCTATAGCTGGTCATATCCTCCGAACTTCTTGTTGCAATGCCTGCAATATGATTGTAACTCTTATGATTAATGAAGAGAAAGCGCTCTTGTTAAAAAAACACACACACACAAAGGAAGTTTTCTTGAACCGAGCGGTTGATTTATTCCAACGCCACCGGCCTTGTGATGCTCACACGTAGCACCCACCAACACCTAGAAACAGTACAGCGAAGCGGAATACAGAGCACAAGGCCAGTGACGCGAACCGACGTGACGTGGTCACTTGTCGTCGCCGGCGGCGGCCGCCGCCTTCTTGCGCTCCTCGTCGACCTTGGCTGCGATCATGGCGGCCTCGTGCTCGGCGGCCTCGAGCAGGAAGCTCCCGCCCTGGTCGTCGACGGCGACCTGGAGCGGCTGCGATCCGAGCCCGTCGGCGACGGCGAGCATGATGCGGCGCATCTCGTCTCGGAACTCGGCGCGGTCGACGGCGCCGCTGCCGTCGGCGTCGAACTGCTCGAAGACGGCGTCGTAGAGCGCGGCGACCTCGGCGGGGACCGGCGCCCGNNNNNNNNNNNNNNNNNNNNNNNNNNNNNNNNNNNNNNNNNNNNNNNNNNNNNNNNNNNNNNNNNNNNNNNNNNNNNNNNNNNNNNNNNNNNNNNNNNNNNNNNNNNNNNNNNNNNNNNNNNNNNNNNNNNNNNNNNNNNNNNNNNNNNNNNNNNNNNNNNNNNNNNNNNNNNNNNNNNNNNNNNNNNNNNNNNNNNNNNNNNNNNNNNNNNNNNNNNNNNNNNNNNNNNNNNNNNNNNNNNNNNNNNNNNNNNNNNNNNNNNNNNNNNNNNNNNNNNNNNNNNNNNNNNNNNNNNNNNNNNNNNNNNNNNNNNNNNNNNNNNNNNNNNNNNNNNNNNNNNNNNNNNNNNNNNNNNNNNNNNNNNNNNNNNNNNNNNNNNNNNNNNNNNNNNNNNNNNNNNNNNNNNNNNNNNNNNNNNNNNNNNNNNNNNNNNNNNNNNNNNNNNNNNNNNNNNNNNNNNNNNNNNNNNNNNNNNNNNNNNNNNNNNNNNNNNNNNNNNNNNNNNNNNNNNNNNNNNNNNNNNNNNNNNNNNNNNNNNNNNNNNNNNNNNNNNNNNNNNNNNNNNNNNNNNNNNNNNNNNNNNNNNNNNNNNNNNNNNNNNNNNNNNNNNNNNNNNNNNNNNNNNNNNNNNNNNNNNNNNNNNNNNNNNNNNNNNNNNNNNNNNNNNNNNNNNNNNNNNNNNNNNNNNNNNNNNNNNNNNNNNNNNNNNNNNNNNNNNNNNNNNNNNNNNNNNNNNNNNNNNNNNNNNNNNNNNNNNNNNNNNNNNNNNNNNNNNNNNNNNNNNNNNNNNNNNNNNNNNNNNNNNNNNNNNNNNNNNNNNNNNNNNNNNNNNNNNNNNNNNNNNNNNNNNNNNNNNNNNNNNNNNNNNNNNNNNNNNNNNNNNNNNNNNNNNNNNNNNNNNNNNNNNNNNNNNNNNNNNNNNNNNNNNNNNNNNNNNNNNNNNNNNNNNNNNNNNNNNNNNNNNNNNNNNNNNNNNNNNNNNNNNNNNNNNNNNNNNNNNNNNNNNNNNNNNNNNNNNNNNNNNNNNNNNNNNNNNNNNNNNNNNNNNNNNNNNNNNNNNNNNNNNNNNNNNNNNNNNNNNNNNNNNNNNNNNNNNNNNNNNNNNNNNNNNNNNNNNNNNNNNNNNNNNNNNNNNNNNNNNNNNNNNNNNNNNNNNNNNNNNNNNNNNNNNNNNNNNNNNNNNNNNNNNNNNNNNNNNNNNNNNNNNNNNNNNNNNNNNNNNNNNNNNNNNNNNNNNNNNNNNNNNNNNNNNNNNNNNNNNNNNNNNNNNNNNNNNNNNNNNNNNNNNNNNNNNNNNNNNNNNNNNNNNNNNNNNNNNNNNNNNNNNNNNNNNNNNNNNNNNNNNNNNNNNNNNNNNNNNNNNNNNNNNNNNNNNNNNNNNNNNNNNNNNNNNNNNNNNNNNNNNNNNNNNNNNNNNNNNNNNNNNNNNNNNNNNNNNNNNNNNNNNNNNNNNNNNNNNNNNNNNNNNNNNNNNNNNNNNNNNNNNNNNNNNNNNNNNNNNNNNNNNNNNNNNNNNNNNNNNNNNNNNNNNNNNNNNNNNNNNNNNNNNNNNNNNNNNNNNNNNNNNNNNNNNNNNNNNNNNNNNNNNNNNNNNNNNNNNNNNNNNNNNNNNNNNNNNNNNNNNNNNNNNNNNNNNNNNNNNNNNNNNNNNNNNNNNNNNNNNNNNNNNNNNNNNNNNNNNNNNNNNNNNNNNNNNNNNNNNNNNNNNNNNNNNNNNNNNNNNNNNNNNNNNNNNNNNNNNNNNNNNNNNNNNNNNNNNNNNNNNNNNNNNNNNNNNNNNNNNNNNNNNNNNNNNNNNNNNNNNNNNNNNNNNNNNNNNNNNNNNNNNNNNNNNNNNNNNNNNNNNNNNNNNNNNNNNNNNNNNNNNNNNNNNNNNNNNNNNNNNNNNNNNNNNNNNNNNNNNNNNNNNNNNNNNNNNNNNNNNNNNNNNNNNNNNNNNNNNNNNNNNNNNNNNNNNNNNNNNNNNNNNNNNNNNNNNNNNNNNNNNNNNNNNNNNNNNNNNNNNNNNNNNNNNNNNNNNNNNNNNNNNNNNNNNNNNNNNNNNNNNNNNNNNNNNNNNNNNNNNNNNNNNNNNNNNNNNNNNNNNNNNNNNNNNNNNNNNNNNNNNNNNNNNNNNNNNNNNNNNNNNNNNNNNNNNNNNNNNNNNNNNNNNNNNNNNNNNNNNNNNNNNNNNNNNNNNNNNNNNNNNNNNNNNNNNNNNNNNNNNNNNNNNNNNNNNNNNNNNNNNNNNNNNNNNNNNNNNNNNNNNNNNNNNNNNNNNNNNNNNNNNNNNNNNNNNNNNNNNNNNNNNNNNNNNNNNNNNNNNNNNNNNNNNNNNNNNNNNNNNNNNNNNNNNNNNNNNNNNNNNNNNNNNNNNNNNNNNNNNNNNNNNNNNNNNNNNNNNNNNNNNNNNNNNNNNNNNNNNNNNNNNNNNNNNNNNNNNNNNNNNNNNNNNNNNNNNNNNNNNNNNNNNNNNNNNNNNNNNNNNNNNNNNNNNNNNNNNNNNNNNNNNNNNNNNNCGGAACTCGGCGCGGTCGACGGCGCCGCTGCCGTCGGCGTCGAACTGCTCGAAGACGGCGTCGTAGAGCGCGGCGACCTCGGCGGGGACCGGCGCCGGCGCGGCGGACCCGAACCCGGCGCCGTCGAGGAGGCGGAACGACTCCAGCGCGCGCCGGAGCTCGGCCCGGGACAGCACGCCGTCGCCGTTGGCGTCCAGGTCCGCGAAGCGAGCGTCCACGCTGCGCGCGAACGCCGCCTCGTCCGCCACGAAGGAACGCACCGTCGAGCCGTCGAGGACAACCACGCTCATGGCGAAGGACTGGATCGATCTCAGCTGCAAGCCAAAGGCAAGGGAGTGGAGTGGAGTAGTGCTGTAGTGGAGCGGAAGGGAGAGCGGTTTGGTTTGTGATGAGCGTTGTGGCGTCGGTCGCGCGGCTTTGCGTGATGTTTATCTTCTTCATTCAGTGCGCCGTGTGGTGGTTAGGATATTTTTCTTGCTCCATCGTTGTCGACAGTCAGTGGAGCGAGAATCCACGTGCACCTGCACCATTTGGAATTTTCGCGTTCGCTTATTTGTTTTATTTTTGTCGATGAGGTTGTTTTACTGGAGAAATACTTGTACAGATTATATACCTGGTTGAAGAAAAAAAATGCAATCGATACTCTTTTCAGGATATGTATCAAATCATTCTGAAATGCGTGGACTGCGTTGCAATCAGCTGCTTGCAGTGGTACTTGCTCGACTGCTTCAGATAAACGCAAGCGGCACCTATGGTGGGTGGTATATCCGGCGGCACATCCGAAGAATTTGTCGGAGCAGCTGGAAAGTGTCAAATGACTTTTAAATTTTATTTTTCTCAAGGAAGGTGACCTCCGAGTTGAATAAAGACCAAACAAACAATACTGGTGGCCGTCAGCCGTTGGAAACTAAACAACGGCAAGCCCGCTGTTAAAAGATCAGAACCTTATCTTAACTAATTACTCCATACAACACGTAGTAACTTTTCTGATGCCTACAAGTGTCATTAAATTTTAACCAATAAAGATGTTGAGATCGATTAAATCATATCATATGACATGTTACAGGTGACATGACATGCAATCTTTGGCTTAAAGCTGGTGTAGAATTCTGAGAGCCTACTTAACTTAAAGCTGTTGCAGAATTAATATGAAATTATGTTCTTTGCCTTAACATTGAAGCAATTTCTGACATTGGTTTGCTTTATGTTCTTTTGCCTTCCGGTATACATCCACGATATGGTTAGAGTTATTTTGTATTGAACGACGTGGCTTAACACGTAGGGGACAACATGAATTGATCCACAGGCCTTAAGTTTAACTCCGATCTTTACTTGATGATGTTATAACACAACTTTTTAGTTTTTGTACTTTATAATATAGCAAAAACAAATAAAAAATCATTTCTTGCTGCAAATAGCGATGGCCAAATAGGCCGGGCCAAACGGGCAGGCACGAAGCACGTAATTTTTGACCTGGCCCGATCAATTTCGAGCCCGAGCTGACACGTCACGCACCATCGGGCCGTGCTTGGGCCTAGGAGTGGGCAGACGGGCGACACAGCACGGCCCGATTAAGGTTTATTTAAATCTATTTTTAGTTGTTATATGTACAGCTAGTAGTCTTAGTCTAGTGGTAAGTGAATTCCTTCTTAACCATGAGGTAGTAGGTTCAAAATTCTCCTTACTAGCATCTATTTTTGCACCATTTAACACTAAATTCCTTCAAAACATAGTTAAATGAGCTGAAATTTAAAACGGGCTGCCGGGCTAAACGGGCCGGCCCGACATGATTAATCAATTACGTGCCGTGCCACGGACCAAAGGAAGAGCCCGCGGGCTGGCATGACCACGGCCTGAAACTAATTGCGTGCCGTGCCAGCCCGAAATAGATTGGGGCCGTGCCTTTTCGGGTCCGTGCCGGGCCGGGCCGTCCGTTTGGCCATCACTAGCTGCAAATAGGAAGCGACCAAAGACACGACCCGGCCCAAGAAAGTTCAGCCCGTCTGAGACCTGACAGCGAGCGGGCGAGCCCGAGGGAGCGAGTCCAACCTCATCCCTCCTGGTCACTCCTCACTCGGCCCATGGCCGCCGTCTCCACTCCCCTCGCCGTCCGCTCCGCCCCTTCCGGCCTCCTCTCGCACCGGTGAGTAAAACGTTTTCCTCTCTCCACCCCCCTCCTCCGCCCCCAGCTCTCAGCTTCTCTCTCTCCCCAAGCTGCCCAATCGCCACTAATCACCTGTCTGGTTCGTTGTGTAGGCGGAACGGAGCCAAAGCGCACCTGGGTCTCCGAGGTGAGATCTTTGTACACTGGTTAGCATGATGTTTGTTGCATTCATGTAGAGCCACCCAATGAAGAGTAAGTTGTGCTGGCGAGGACTGAATTGGATCGTTTGATTTTATTCAGTAACAGTTCGATACGGTAGGGCATTTCGTGATTGTTGCAGAAACCAGATGTCTCGGGTATATATTAGCTGATAAAATGATAAGGCGATACCAATTGTGGCCGTTGTAAACTGTAAAGCCCCCAGGTCACTTCTAGACTCATGGGGGATAACTGAAAGTATCAGTAGATGTCTCACTTCTCTTCTACAACGTATGCTTTGGTCCACTATCTGCATATTAAGTCACAGATTCTAGCATTGCTGCAGCAGTAGAACACTAGAATGCCATGTTACTTGGTTGTATTCCTAAGTTCGTGACGCGAATGTCATAAAAGACTAACATCCGCCGGAGTAATGGCAATGTAATCAGACCCAATTTTTCTCCTTAATACAAAGGCGCACAGTCCTCCTGCGTGTTCGATAAAAAATGCATATTAGGTTCGGTGAACCGGTTCTTGAGTCCTGCTAATTGGTGGTGTCTGAACTTAACCGTGTAGGGAGCCAGAACGATGGATTACTTGAACAATGCCAATTGGAGTAGTTACCTGTTAATCTACCAATGCTTCAAATAAAGAACACGTGAACAATAGCAGAGTAACAGCTGCAGATGTTTGTCTTACGGAATGTGTAGCAAAAGATTCAGAAAATCATTGTATATTAGCTCACTTTCTTTATGATTTCGAGTTGTTTTAGGATTTTCTTTGCAATTCATGCTGCCAACATATGGGTACGACTGTAGTGTTGAATGATATTTCTTATTTTATTTGTGTTACTTATGTTAGCGATGGCCATGTATATTCAAAACTCCACATGTTACTTGAAGGTGTGGTCTTTTAACATTGTTATGAACACACAAATTGCCAATACTGAAATGGACGAATATTTTTCCTGTTTACATAATAGCATTTTTCAATAGTACTTTGCACTATGTCAAATTGCTATCTCACTCATATCATACACATAGGAATGCAATTCGTAACACATGGCGTTCCTTCACGGCCGCATGACAGGTTACATTGCTGTTCTTCTCTCAGGTAATATATTTGCAACACTGTCATCCTGATAAGCATGTCCTTAATTTTTAAGCCACAAAATTGTAATGTGTTTTTTATAGGCAAAACACAATTGTAGCATCGGAGAATGAAAATCCTCCTTTAATGCCGGCAATCATGACTCCTGCTGGGGCTCTTGATCTGGCAACTGTACTACTGGGAAATCGTGTTATCTTCATTGGTCAATATATTAACTCCCAAGTAGCGCAACGGGTGATATCACAGCTTGTCACACTTGCTGCTGTTGATGAAGAGTCTGATATTCTGGTTAGTATGCATTCATATATGTTCAAGTTCCACAAGCATTATGAAGTAGCAAGCCCCCATTGCTGTTCATATTATTTTCTACAAACCCTTGTATCACTGAAATTATTTTTTCTGTTTTCTCAGATCTATCTCAACTGCCCTGGTGGAAGTCTCTACTCTATCTTAGCTATTTACGATTGCATGTCCTGGGTAAGTGACCTAGGTTCCTTGCCTTTTCTGACCAAATATTGTGACTGTTCATAAATCCTACATTTCAAGCTTCAAGATTTACAATCTCGTCAGCACAATACATTCTTCTGTTTTTTTTAATGTGGGGAAATAAGTACGTTAGAAGGAAGGGGAAGAGGCCCACAGCAGAAGTGTTTTTGCTTGCCAAAACAAGTTAGTTGAGTCGTCAAATATGGTTGTTAGTGATAATCTGATTAGTTCAGTTAGGACTCTGTAATTTGAGTCGGTTAAGATGTACTACAACCGGCTATAACCATGGCAAGATCAAGGAACAAAGGCAAGCAAAGTTCTTAAGCTTTGGGGATAGCATAAACCCTAGTTACCGCCACCATAACAGTTCTGGTATGCTCCTTAGCTTCCATGTAAACTGATTGTGGAAGCCTTATAATGCAGATGCTTGGAGTATAAAGATAGTGGTACATAGTATGCTTGTATGATTTATGTTAGTGTCATTTTATGTTAGTAATGAGCACATGGGTTTTATGTTACTTCTTTTCATTGTATGTTTTTCCTTTGCTTATATGATATGTTTATCCTTTTGGGACATGCACTAAAACATTTATTAGCTTTAATGTTCTTTTATTGTGTTTTTATATTTTAGTAATCAAGTAAAAAAACAAGGGAAAACAATATGTGTACTCCAAACAGGGCCTCAGAGCGTGCTTGGCCTCTGGTCAGTATGAGCCATTCTTACACCACTCACCTGTGACAAATCTGTTCCCCCTTTTATAAATACTCTCCATTCTGTGATAAAAAAAGATACTCGCCATTCTTTTCTTGATGTTGTTTCAGACAAGCTTTGTAGTTCATTTTCAGTTCATTCTTGTTGTCTGAAACGACAACAAAAAAAAAGAATGGAGGGAGTACAAAACTATAAGTGCTCTCTAGAGTATATAAAACTTTTCTGGAGAAATCTACAAAACATTTTTATCTTGGTAGGGCCCTTCGGAGTCTCCCATAAACAGATTAAGTTTTTTTTTGGGGGGAAAAACAGGTTATGTTTTCCCATGATGTTTGTGTGCTAGACACTTATATTGGCTTAGGGCATGCGGATGATAATTTTTTATGCTTATGTAATATGCATCTAGTGACCATTGTTTTAAATAGCGGGCTATGGACTTATAGCGTCTTGCTTTTAAAACAGCTAAATGAAGCTATAGCGGGGCAATAGCGGGCTAAATCAGCTAAGTTGTACGTGAAAGCAAATAGCGGCATACCCACTAAAACAGCTATAGCGGGCTATAGCGGCAGCTATAGCCGGCTATTTGAAACTTCTAGTCGCCCAACTGTGAACGTTCACATATATATCATGTTGTTTTGATGTGCCACTCATATGTATTTGATTTGCCTGTTTTAATAGTTCCCTGTGATAAATTTTTGTTTGTGGGCATTAGATTTCATCCTTTAATTTCCCCTTTTGTCATCTTTGTAGCTGCTATACAACAAACACTTGAATCTGTAATGACATTAGTATACCCAGAGAGTTAATTCTCATTCTGATAACTGTGCTTGAGATCTCACTTCTTTTATCATGAGCAGATAAAACCCAATGTTGGGACAGTGGGTTTCGGTGTTGTTGCTAGTCAAGCAGCAATCATTCTTGCGGGTGGTGAGAAGGGAATGCGCTATGCAATGCCAAATACTAGAGTAATGATTCATCAACCTCAAGGTGGATCTGAGGTATGGCTGTTGGCGTTGTATTGGTTTGAGAAGCCAGATGAATTTCTAGTTGTCTGACTAGTATAGTAATAATTATCTTTTTGCTGGCGTCACATCTTATTTATAGTGCATCTGTAAACTGTCCTGAAAATTGAATGACCTTCAGGGTAATGTGGAGGAGGTGAGGCGGCAGGTGGGGGAAACCATTTATGCTCGAGATGTAAGTCCTGTGTAAGAGCGAAAGAGTGATGTGTCTTCTTCTGGATGATGCTAAGAAGCAACCTAACTAGGCTCTTAATTTTGATTTGTAAACTCTTATAATTTTGTTTGACATTCTGCTTGCAGAAAGTTGATAAAATGTTTGCTGCCTTCACTGGGCAACCCTTGGATATGGTCCAACAGTGGACAGAAAGGGATCGCTTCATGTCTTCTTCTGAAGTAACAATCATCTCTGATATATAGCAGAATAAAGCAAAACATCAAATTTGTCTTGTTGAGACTATAACTTAGGGTGCACCATTTGCAGGCCATGGATTTTGGACTAGTTGACTCCCTGCTGGAGACAAGATACTAGCTACGAAACGTACCCACACTCTGTTAGCAAAGATTTCACAATGGTAGCTGCTGTTAGAAGTTTTGGTTGGCACCCTAAAGCCTTGGAATGGAAGGAAGTTGGACATCACGATTTGTCATGGTGCATGAGTGGAGCAAATATTTTGATCACTGTTGTGTGTGGTTCGGCCTCTGCGTGACTGGGTCTGCAGTCTTGGGTTGTGACATTCTCCAGCTTTTATGGCGTCTGATACAATGGCCCGGTAGTGTACAATGAGCAAAGCTCCACGGAAAAAAATGACTTTAGATGCTTAGGTTTAGCCATACCAATAGGCACCTGTTGCGAGTTGTAACAGTTGTGTGGTTTTTCCATTTTAAAAGACACATGTACCTGTGGTGTGCATCTGGCCCTTCAATTCTTTTCCTGACCAATCTGTATCACCCTTTCTAACAAATTAGGAGTATGTGAACTGAAAGTTGAATTGGAAGTGGAGGTGAAATTGGAGCACCTTTGGCAATCGTGGTGATGGACCTGCAACCAACTAATTCCAGCGTCCTTCCACTAACAAGCGCCTGCGCCGCTAGTTGAGCGATACCGGAAGCGGGGCCCACCCAGCCAACCTCACTAGCAGTCGCTAATTACAAAAGGCCAGCTCACAAGGGCCCTCACTGATCACGCTTCTCCCTCCCGCTCCACCGACGCCGCCATGAAGAACGCTGCCGCCGACATCACCGTCGACTCGGTGCCGGCCACCAAGATCGCCATGCCTGCCTCCGAGAAGGGCTCGGAGACGGCGATCCTCGGGAAGGGCCGGTACAAGGTGTGGGCCTTCGCGGCCATCGCGCTGCTCGCGCTCTGGTCCATGTCCGCCGCCTCCGTCTCCCTCCGCTGGTCAGCCTCCGGCGACCTCGCTGTGGCCGGCGACCTGGATGTCCCCCTCGGCGACGACCTCGACTCCCTCGTACGCGCCCTTCCTCTTAAATTTCGGCCTAATTAGTCATTTGTGTCTGGTCTGTTCTAAATGCACGTCAACAGCTGTTCGTAATTTGCTTAATTGCGTAAATGAGTGTTTCTGTAGACTATAGTTTGTGCTTCCAGGTTCATTTTTTTTATTCTTTTAAGGTGAGTAAGAGGTGTGGCAAAGTGAATGTTACAACTGTTTCACCAATGTTACAAATATAAATCCCTTGCAATGTTAGTAAGAACAGGATTGGGGTGAAGCAGGGTTTCAAATCTTGTAGTAAGCAGTCGGTCACCCATTCCTCATGCCCCCTGGGTACTGCAAGCGCAAATCCGGACTGGCAACATAATTAGCCTAATCTAAATTCAGATTCTGTATTTAATAATAGAAACGAATCAAAACTGTTTGTGAGAACAACGGTTATGGGTGGTAGCCAAGTGTCTGGGGGTTTGTGGCAAATCAGAATTGAACGCCTCTGGTTGATCTAGGTTCCTCGCCAACGTAGGCGCTCCATGAATTTTTGGCGGGGTTCATCCTACATAGCTACTGCCTACATCTTACTTGTGCTTAGCATGCTTACATCCTTTTTCTGCAATTATATTTGTGGATAAGGCGTTCTTCGTTTATGTCTAATTCGAACCTTAGAAAGAAAAAGTTCCATGGATGTATGATTCACGCCTGAATCACATTTCCCAGTTGTTAGATAGGTGTTTCATTATGCTATCAATAGCAGAAAGCACAAGATACATATTTCGATGCTTTATTTTTCTGTGCAAGGATAAGTAACTTCTTTTTCATTAAAAAAATAAATTGATTAGGAGATGGAAGAGAGGGAGAAATTAGTAGGCCGAATGTGGGACATGTACACACGGACCAGTGATGAGGTCCGGCTTCCTCGGTTCTGGCAGGAGGCCTTTGAGGCTGCATATGAGGAACTCGCTGGTGATGATATGCAGGTCCGTGATGCAGCCATCACGGAAATCGCCCGGATGTCAGCCCACAAACTTGAGCTTGAGCAGCCAGTGAATGAGGATGAGGTGTGAAATTCTTGCTCATTCTTGTATTCTTGTTTAGCTTTTTTTTTTGTAAGACAGCCTCATTTTACCATGTCAATATGTACCGTTTCGAATCAGTAGTGTTGTTTTGAGGCCTATTCATGCTAATTTATATCATTTTCGAACAATTTTTGGAAGTCAATACAGTGCATGACCACGAATTTGCCATTTTCCCTGCGAGATGTGGACACATTTTATTCATAGCTGCTTTTAGCTAGAAGATAGAACATTCAGGTATTGTAGGTTTCAGATCATGGACAGGGCCCACTGTCTCAGGAGGATATCATCCTAAATGATGTATAATGTAACTGACGACATATGAAATACATAATCCACAATTGTCCAAAAGCAACACCTTGTTAGTACCCAGTTGCACTGTTATATGTGTATTGGCGGTCACTGTTATGTATTACAATGATTTTCTAATTCAGTGACCTGGTGCAGGAAGAGAAGAATGCAGTGGGCAACAGAAGTGGTACGTCAAAATGATGTAGTTTCATGAATGTCAGTAACCTGTGAGCCTGAAGATCCTTTGTGGCGTTCGACAATCAGCAGCTGATCGAATCTTGGAGTTTTGATGTAGTCCATTGGTTATTCACTGGTTTTGTGCGCCAGCTAAGCCCCAAAATAGGTGCATTTGTGGCGCCATGTAACGTTATGACTTGTTCCCCTTTTGTATCGGAGGGTAAGCTAGCAAAAGCTTGCAAATGCTGTGAGTAAGCATCCCAGTGAATACGATCCGAGTTACCCAGCTTCGTCATGCTGTCTTGCGCTCCCAGATGTCATTGACAGAGTGGTTGTATTTTTTTAGATGATGGGAAAAAACGGTTTCCGGCCTTACCTCACATGAGGCTACGGCCGATTATAACTCTTAGCAGCACATCCGTCTGCCACAAATCACGAACGAAAGTAAAGACAGCACCAACATAGTTTGAACAGAGCGGTTGCATAATCTAGGAGGCAGTCTTGGGAGCGGACAAAAGACACAAATTTTTGAATTTCTCATGACTCTTCGTTTAGGAGTGTCCAGCAGTGGCATCTTGCAAAATTTCAGTGTTACGGCTATTTGGTTGTGGTTGAGCTTCATTCCTTTGAATTCCTTGGATGAAACGGCGATGTCAAACAGCACAGCTAGGTTGTCCATCGACTAATAGAGGAAGAAGTCCATGTGACTGAGGAAAATAACGCATGGTTCTCACTGTGTTACATCGACTCTAATACTTGCAGAGTTTGCAACGTCGATGCTGCGCTGACGGCAAGAAAACTCAGCCGTAATAAGAGCATCAGATGCTTATTTACGAGTAGCAAAACCCAACCGATCGATAGCTACGATGCTTATTCAGTAGGGATCAAAGAGCAACAACAAGTAACGTTTGGAAATTCAAATCAGCTCAAAACCTCGAAAAAGTCGAATCTGAGAGTTTGCCCTCCTCTGCTGACTGCTACGATGAATGCGATTCTTTGGAGACCATGGACGTGGAAGGAGAATCGGATCAAGCGGTGGCCCAGTTGGGTTCTGGCAGCGGCGCCATGCTGGAGTGGTACCCGAACACCGTGCCGAAGTCGTAGTGGACCTCCCCGGCGAGCTCCAGCGTGGCCGCCGTGGCGCCGGCGCACCAGGTCGCGGCGCCACACCTGCGCGTCGTCGTCCCGGCAATGACGTCGTGCGCCACCCACAGCGGCAGGCGGGCCCCCCCGGGTCCGTGCCCCCTGGCACCNNNNNNNNNNNNNNNNNNNNNNNNNNNNNNNNNNNNNNNNNNNNNNNNNNNNNNNNNNNNNNNNNNNNNNNNNNNNNNNNNNNNNNNNNNNNNNNNNNNNNNNNNNNNNNNNNNNNNNNNNNNNNNNNNNNNNNNNNNNNNNNNNNNNNNNNNNNNNNNNNNNNNNNNNNNNNNNNNNNNNNNNNNNNNNNNNNNNNNNNNNNNNNNNNNNNNNNNNNNNNNNNNNNNNNNNNNNNNNNNNNNNNNNNNNNNNNNNNNNNNNNNNNNNNNNNNNNNNNNNNNNNNNNNNNNNNNNNNNNNNNNNNNNNNNNNNNNNNNNNNNNNNNNNNNNNNNNNNNNNNNNNNNNNNNNNNNNNNNNNNNNNNNNNNNNNNNNNNNNNNNNNNNNNNNNNNNNNNNNNNNNNNNNNNNNNNNNNNNNNNNNNNNNNNNNNNNNNNNNNNNNNNNNNNNNNNNNNNNNNNNNNNNNNNNNNNNNNNNNNNNNNNNNNNNNNNNNNNNNNNNNNNNNNNNNNNNNNNNNNNNNNNNNNNNNNNNNNNNNNNNNNNNNNNNNNNNNNNNNNNNNNNNNNNNNNNNNNNNNNNNNNNNNNNNNNNNNNNNNNNNNNNNNNNNNNNNNNNNNNNNNNNNNNNNNNNNNNNNNNNNNNNNNNNNNNNNNNNNNNNNNNNNNNNNNNNNNNNNNNNNNNNNNNNNNNNNNNNNNNNNNNNNNNNNNNNNNNNNNNNNNNNNNNNNNNNNNNNNNNNNNNNNNNNNNNNNNNNNNNNNNNNNNNNNNNNNNNNNNNNNNNNNNNNNNNNNNNNNNNNNNNNNNNNNNNNNNNNNNNNNNNNNNNNNNNNNNNNNNNNNNNNNNNNNNNNNNNNNNNNNNNNNNNNNNNNNNNNNNNNNNNNNNNNNNNNNNNNNNNNNNNNNNNGGCGCCAGCCGGCCTCGCCGGGCTCCGTCCCCATGGGCACCGCCGACCACATGATGTAGCATGTTCTGATGGTGTAAAACATCGCTAGGTGAAACCGTGAAAGAGAGTAAGTACCTGGCGCATTCCCTAAAAAAAAGGAGAGAGAGGAGAGTACCTGGCCAATCTTCACAAGAAAACCTTCACCTTTGAAGGGGCGAAGCCAAGCCTACTGGGTGCAGATGCACCCACTGAATTTTAGACTTCACCTGTAATTGACTTGTTTTCACCATATATGCACCCTATAACTTCAACTTTATGCATCTTTTGACAGAAAAATGAGTTATTTTCTGTACATGTTATCAAGTAATGCACCCGCTCTAGCTTCGCCACTGGACTCCTTCGATGGTACCTTACGACTGTCCACAACGCAGTCCATTCCTTCTCATCAGCTTTAGTGTTCGATAGTTGCACACGGGTATGTCATAAATCATTTGGTGTTAAACAGATGTAGTGCTTTTTTTGCGATGGTTAGTGCTTGCCGCGGCTGAAGCTCTGGTTGTGGCTGCAACAATTAGCATCCAAAACACTGTGCGTAAAGGTAGCCACAGTTGCAGGAAGGAAGCACATCCGGTCGGACCAGCGAGCGGGGTCGTTCGGTTGACGCATTTTTTTCCCGCGCACGTGCCTGCCGGACGAAGCCATATGAGGGACGGACGACAGTGTAGCGTTTAGGAAATGGACGGTAGGATGTTGATGGACATACGATAGTGAAGCGTTTTCGGCGGCTAGGTGGGCCCACCCCGGCGCACTGTGGGCCCCAGCCCGGTGCCACCAACCCGGCGCACTGTGGGCCCCATCCCGGTGTGTGGGCCCCATCCCGGTGTGTGGGCCCCATCCCGGTGCCACCTTCCCGGCGCACTGTGGGCCCCATCCCGGTGCCACCTCGGCGCCTTACGCGAGACTATAAATAGCAGGCGAGCGGGCAAAGGCCATCATCACAGATTCAGACCAACTTACCAAGACCAAGCTCCCCATCCACACACCATCGGAAGCCGTCGAGCTCTCCACCCCGCACGCCACCGGAGGCCGTCACGAGCTCTCTACCCCGCACACCACCGGAAGCTCAAGCCGTCGAGCTCGCCGACGTTCTTGAAGGGCAAGGTCCTGGTGCTGCTGTCCCTGATCGTGGCCGTCCTGCTCCTCCCCCTCGCCACCGGCTGCCTTCGCGGCGTCGACGTCCCCGGGCTCGCCACCAGGCGGAACATGATCCTGCTCTTCCACGCCATCCTCCTCTTCGTCCTCTGGGACGCCGGCCTCTTCGGCACCCCGTCTCGCCGGCGCACGCCAGCTGCGGGTACCCGGTACCCTGCGGAGCGGAGAGCCTGCATCGACGATCACAACCTCATCCAAGGTAAACTTGATCAGCCTGTGGCTACTGCCATGACCCAAGGCGTGCTCATTTATTTTTTCTATTTTTTCTGTGGATTCGACCCTGCTTTTGTTGTTGCTAACGACGAAAGAAAAGGAGCGCAGACGTGGAGGCGGCGGGACAGCAAGGTGCCTTAGCAGAGGAGTGCGGCGAAGGAGAAGAAGATGACCTCGACGAGATGAACAGGAGGTTCGACGCGTTCATCGCCGACATGAAGAGGAGGATGCAACTGGAGGAGCTGGAGCTGCGGCTGCATGCCTCAGGAAGAAACAGTCGAGGACTGTAAACACTACAGTGAGATGGGTTCAGTGACGCAAAGTGTGATTGCAGAGTGGGTACTGTTATGGAGTGGATATGGTTTAAGCTGGCCTTGGCTCGACTGGTTTTCTGGTGGCATTGGTTTGTGATGCTGCTGTGTTGGAATTCATATGATGCGATGCGGTCACAGATATGGAGATGGCTATGTGGAAGAAACTGTAGGCAGATTAGTAGTCAGTTTTGCAGGGGAGGAGCGTCAAGAGGCATGACGAATGTTTTTTTTTTTTTTTAGTTCGATCCTCGTGTGTGGAGGATTGCTTGTTTTTTTCTTTCTTTCTTTCTTTCTTTCTTTCTTTCTTGTATCGTTTGGTCTGACGGGCTTCAACAGAAGCCGGGGCCTTAATAAAGCCGGTGCTCTCCTGAAGATCGTTAGTTCTCACCATGTGTTAACTCGTTTCGTCTCGTCAGCAGTCACCATTCTAGGCTGTTCATGATACATTTTCCAGTGTTGATCGTAATGACATCAAATGGTGAATATGTCGGACGATCCTTTCGTTTTCCCTTTGATTTCAAAGGGAAACAGAGTATAGTCTGGATCCGGTTGCTCTGCTCCGAGAGTAAACACGGAGAAGCAGAGCGCAATCACCATCAGAACTGTCATTAGTAGCAAGTAATTATACTTGTCCCTGTCGCTTTATAAAGAGTATGCAATAATCTTGATGCATGCCGCTGCTTGCTGACAGGCACCGCCATCGCCGCATTTCTTCTCCTTCCCGGCCGTCGCCCGCTTCCGCCATAGCTGCGCCGAGCTCGAGCTTTACTCGCCGTTGCTAGCCACCACCGAGNNNNNNNNNNNNNNNNNNNNNNNNNNNNNNNNNNNNNNNNNNNNNNNNNNNNNNNNNNNNNNNNNNNNNNNNNNNNNNNNNNNNNNNNNNNNNNNNNNNNNNNNNNNNNNNNNNNNNNNNNNNNNNNNNNNNNNNNNNNNNNNNNNNNNNNNNNNNNNNNNNNNNNNNNNNNNNNNNNNNNNNNNNNNNNNNNNNNNNNNNNNNNNNNNNNNNNNNNNNNNNNNNNNNNNNNNNNNNNNNNNNNNNNNNNNNNNNNNNNNNNNNNNNNNNNNNNNNNNNNNNNNNNNNNNNNNNNNNNNNNNNNNNNNNNNNNNNNNNNNNNNNNNNNNNNNNNNNNNNNNNNNNNNNNNNNNNNNNNNNNNNNNNNNNNNNNNNNNNNNNNNNNNNNNNNNNNNNNNNNNNNNNNNNNNNNNNNNNNNNNNNNNNNNNNNNNNNNNNNNNNNNNNNNNNNNNNNNNNNNNNNNNNNNNNNNNNNNNNNNNNNNNNNNNNNNNNNNNNNNNNNNNNNNNNNNNNNNNNNNNNNNNNNNNNNNNNNNNNNNNNNNNNNNNNNNNNNNNNNNNNNNNNNNNNNNNNNNNNNNNNNNNNNNNNNNNNNNNNNNNNNNNNNNNNNNNNNNNNNNNNNNNNNNNNNNNNNNNNNNNNNNNNNNNNNNNNNNNNNNNNNNNNNNNNNNNNNNNNNNNNNNNNNNNNNNNNNNNNNNNNNNNNNNNNNNNNNNNNNNNNNNNNNNNNNNNNNNNNNNNNNNNNNNNNNNNNNNNNNNNNNNNNNNNNNNNNNNNNNNNNNNNNNNNNNNNNNNNNNNNNNNNNNNNNNNNNNNNNNNNNNNNNNNNNNNNNNNNNNNNNNNNNNNNNNNNNNNNNNNNNNNNNNNNNNNNNNNNNNNNNNNNNNNNNNNNNNNNNNNNNNNNNNNNNNNNNNNNNNNNNNNNNNNNNNNNNNNNNNNNNNNNNNNNNNNNNNNNNNNNNNNNNNNNNNNNNNNNNNNNNNNNNNNNNNNNNNNNNNNNNNNNNNNNNNNNNNNNNNNNNNNNNNNNNNNNNNNNNNNNNNNNNNNNNNNNNNNNNNNNNNNNNNNNNNNNNNNNNNNNNNNNNNNNNNNNNNNNNNNNNNNNNNNNNNNNNNNNNNNNNNNNNNNNNNNNNNNNNNNNNNNNNNNNNNNNNNNNNNNNNNNNNNNNNNNNNNNNNNNNNNNNNNNNNNNNNNNNNNNNNNNNNNNNNNNNNNNNNNNNNNNNNNNNNNNNNNNNNNNNNNNNNNNNNNNNNNNNNNNNNNNNNNNNNNNNNNNNNNNNNNNNNNNNNNNNNNNNNNNNNNNNNNNNNNNNNNNNNNNNNNNNNNNNNNNNNNNNNNNNNNNNNNNNNNNNNNNNNNNNNNNNNNNNNNNNNNNNNNNNNNNNNNNNNNNNNNNNNNNNNNNNNNNNNNNNNNNNNNNNNNNNNNNNNNNNNNNNNNNNNNNNNNNNNNNNNNNNNNNNNNNNNNNNNNNNNNNNNNNNNNNNNNNNNNNNNNNNNNNNNNNNNNNNNNNNNNNNNNNNNNNNNNNNNNNNNNNNNNNNNNNNNNNNNNNNNNNNNNNNNNNNNNNNNNNNNNNNNNNNNNNNNNNNNNNNNNNNNNNNNNNNNNNNNNNNNNNNNNNNNNNNNNNNNNNNNNNNNNNNNNNNNNNNNNNNNNNNNNNNNNNNNNNNNNNNNNNNNNNNNNNNNNNNNNNNNNNNNNNNNNNNNNNNNNNNNNNNNNNNNNNNNNNNNNNNNNNNNNNNNNNNNNNNNNNNNNNNNNNNNNNNNNNNNNNNNNNNNNNNNNNNNNNNNNNNNNNNNNNNNNNNNNNNNNNNNNNNNNNNNNNNNNNNNNNNNNNNNNNNNNNNNNNNNNNNNNNNNNNNNNNNNNNNNNNNNNNNNNNNNNNNNNNNNNNNNNNNNNNNNNNNNNNNNNNNNNNNNNNNNNNNNNNNNNNNNNNNNNNNNNNNNNNNNNNNNNNNNNNNNNNNNNNNNNNNNNNNNNNNNNNNNNNNNNNNNNNNNNNNNNNNNNNNNNNNNNNNNNNNNNNNNNNNNNNNNNNNNNNNNNNNNNNNNNNNNNNNNNNNNNNNNNNNNNNNNNNNNNNNNNNNNNNNNNNNNNNNNNNNNNNNNNNNNNNNNNNNNNNNNNNNNNNNNNNNNNNNNNNNNNNNNNNNNNNNNNNNNNNNNNNNNNNNNNNNNNNNNNNNNNNNNNNNNNNNNNNNNNNNNNNNNNNNNNNNNNNNNNNNNNNNNNNNNNNNNNNNNNNNNNNNNNNNNNNNNNNNNNNNNNNNNNNNNNNNNNNNNNNNNNNNNNNNNNNNNNNNNNNNNNNNNNNNNNNNNNNNNNNNNNNNNNNNNNNNNNNNNNNNNNNNNNNNNNNNNNNNNNNNNNNNNNNNNNNNNNNNNNNNNNNNNNNNNNNNNNNNNNNNNNNNNNNNNNNNNNNNNNNNNNNNNNNNNNNNNNNNNNNNNNNNNNNNNNNNNNNNNNNNNNNNNNNNNNNNNNNNNNNNNNNNNNNNNNNNNNNNNNNNNNNNNNNNNNNNNNNNNNNNNNNNNNNNNNNNNNNNNNNNNNNNNNNNNNNNNNNNNNNNNNNNNNNNNNNNNNNNNNNNNNNNNNNNNNNNNNNNNNNNNNNNNNNNNNNNNNNNNNNNNNNNNNNNNNNNNNNNNNNNNNNNNNNNNNNNNNNNNNNNNNNNNNNNNNNNNNNNNNNNNNNNNNNNNNNNNNNNNNNNNNNNNNNNNNNNNNNNNNNNNNNNNNNNNNNNNNNNNNNNNNNNNNNNNNNNNNNNNNNNNNNNNNNNNNNNNNNNNNNNNNNNNNNNNNNNNNNNNNNNNNNNNNNNNNNNNNNNNNNNNNNNNNNNNNNNNNNNNNNNNNNNNNNNNNNNNNNNNNNNNNNNNNNNNNNNNNNNNNNNNNNNNNNNNNNNNNNNNNAAGTCTTGCTGAGTATTAGTTGCTCAGGCTTTGGCTCTTTATGTTTTTGTGTGCAGGTAGTCTTTGCTTCCGCTGACTTTAGTGGAGCTTCCAGCAGGTCCACATCGCAGGCTTCGATGTGCGACCTTTGTATTGTCGAGAATATTAGTTGTTTCTTGTATCTTCAGACTTCCGCTGTGTACTCACGTGTTTTAATCTATTAAAGACTTGTTGTAATAACTTAATTATGCAAAATTTGTGTTCTTTATGTGTTGTACTGTTGTGTCTGTTCATCAGCACGTACCGACTCGATCCTGCGAATAGCGGAAACGCTATAGGCACATAACTCGACTGTATCAAACGCTTAATCAACGAGTACCAAAACCCAACCTATATAGCTACAAGTAGAGATGAAACAGTGACATTCGAAAATTCAAATCAGCTCGAAACCTGAAACGAGTCGAATGTTGAGAGTTTGCCCTCTCCTCGGAAACTAACTCGTGCATGGCACATAAATTGTTTGGAGGCCATGGACGTGGACGGATCGATCAAGCGGTGGCCAGGTTGAGTTCGGGCAGCGGCGCCATGCTGGAGTGAGTGGTAGCCGAACACGGTGCCAAAGTTGTCGTGCACCTCCTTCCCGGCGAGCTCCATCGCGGGCCACCTGCATCGTCGTACCCGCAACCGCAACGACGTGCGCCACCCGCAGCGCCAGCCCGATCGGCGCCGTCCTCGCCGGGCTCCGTCCCCGTGGGCACCACCGACCACAGGAAGCACCCCTGGCCTCACCACGCCGGGGATCCGGCTCCCCCATCCCACCTCGCTCCCACACCTCGACGCCTTCGCCGGTCGCCCGCGCCAGCCTGATCCGGATCCCAGTTGCCCTTCAAGCGCGACTCTCCCTCCGGGCACGACACCGCGGCTCCACCTCGCCGGGTTGATGACGAAGAAGGCGCGCCGCATGCGTCGCCGCCGTGACGCAGAGGAGGCCGCCGGCGGGGGGCGACGCNNNNNNNNNNNNNNNNNNNNNNNNNNNNNNNNNNNNNNNNNNNNNNNNNNNNNNNNNNNNNNNNNNNNNNNNNNNNNNNNNNNNNNNNNNNNCCCCGCTGACTGCGCCGCCGCCGTGACGCAGAGGAGGCCGCCGGCGGGGAGCGACGCGAAGCAGTGGTCCGGCGGGGCGCCGGCGGCGAGGACCTCCAACCGGCCGTTGCGGAAGCGGGCGAGCGCGTCGGGGTCCGCGCCTGCCGGGCGGCGAAGGCGGCGTCGCGGAGGACGAAGTCGCGCCACGACTTGCAGACGGAGGCGCACCGCGTCAGGGAACCGCACGGGAGGCGGGGGAGGACGTTGTCGAACAAGATGTCGTCGGGGAGATGCGCCGGCGACCTCTGCAACTTTAGCTCATCGCCTGCGCCCATCATGCTAGCTTGTCGATATGATCGACGACGGAAACTCTCTACTCCTTGCTCTCTGAGATATAATAAGGCCCGGCAACATTAATTGTTGTATTCTCTTCATAACTCGTTTCCGTCACGTCCTTTGTTTCTAGTTTCCGTCCGTGTACTACCATTATTCTACTTCGCCCCTTCTACCAAAACGTACGAATCAAATTAACAGCCGGATCAGAACATGATTAGTTTCAATTAACATAGTTTTGACAATTGTTTAGTTTATATATTTTTCATGTTGTAACCTAACAAAGCTGTTGAAGTTTGTCAGAATCTTACACTACACCAGAGCACGGAAATACCGACCCACAATCATTCTCAAGTTGTCGTAAGTTAGTTATCAGTAGGTACTTCTTGTTTTGCAAAAAATTGTCATCTAGCCTCGGAGGCGGTCGGTCCTTAATCTCTGATTTACATGTCAAACTATGGGTCGGGAAACCTATGTCGAACTATATGTCTGCTTGTAAGTCTATAAGACACGGGTAAGACACGGGTATTATGGACTACTCCTTCATCCATCCTACACTAGGGAGAACCCTCGGCAAAGCCTACACTCGGCAAAGAAACTCCCATGGTGTAGTTTGATCGGGAAAGGGCCCTTTGCCGAGAACATTTTTCGAGCACTCGGCAAACACTTTAGGGGGCTCCCCGGCAACCGCAACAACGAATTCTTGCGGTCGACGAAACCTGCCTTAGACTAGCTTCCAATCAAGAAGCAGAGCGGAGCGCTTCGCGCGGGGCGCGCTAGCTGCAGGCGAGGGACCCCACCGGCCCCAACGAGTTTCAAAGTTTTAAAAAATGTATAGCTTCCAAACCAAATTTCCGAATTGCAAACTGGTTGCACCATTGCGCTCCTTGCGACGGAATCTTCGAAACTCGATCCCACTTCATTATATTCTGAATTTTTTTAAAAATAAAAGAAATTAACGAATCAAAAAATTGGAGCAACAAATAAAAAATTTAGACAACAAACGAAAATGTTGTAGCAACAAAAGAAAAAAGTTGGACAAAACAAATAAAAAAATTGGACAACTAAATAGAAATGTTGGGGCGTCAATTCAAAAAGTTACACGAATAAACGGAAAAGTTGTTGAAACAAATCAAAAAGTTGGCCGACCAAAGGAAAAAGTTACATCAAATTTTCTATTGTCGATTTTTTGTGTCGAAACACACTCAAGTGAGGTCTAGTTTCGAATATCTCGTCACGAGAAATACGATGATGAAATCACGGTTTACGCTCTACGGATTTTTTTTAGTTGAAACTATTAATGATGTCAGCGCAGACCCCACTCTACAACCAAGCACCCCGCATGCTGCGTTCTCTACCGACATCCTGTCTTTTCCAAAAAATAACACTGACCGCAACAACTAAACCTAGCGGTGACCGCAGACCAGATAAACCCGGCCTTAGGGGGTTTTCCGTAGTGCTACACCAAACGTTTTATACGTAGGAACCGTTTTACTAAAGAAGCCGTTCTTCCAGAGGAGCTAGAGCCGGAGCCAGTTTTCCTATATGTATGTCAAATTGTGATTTGAGCAACAAAGAGTGACATTTTTATGAAGTTTAGGTTGAATCCCTCTCCTCTCCCGACATTTTAGTTCAACCAATGCTTGATGTTTTAACCATTCCTTCTACGAAAATAGTGTCATACATCAGTTTTGTTGTTGAGGACTTGGTGGTCCATCCGGTAGATGTATCACCGTTACTTCACAAAAACGTAGGTAGCTGAATTTTAGCCTGGTAACTTGCTTTATAACCAGCTTTAGAAACCGGCCTGAAGGCTATCCTTATGTATTTTGAAAAATTGTCTGGAATAACCATTAATTCCCATAAGAGTGCGAACGAACATAGAGGAATGACAGATCCATGAAATTGCACACATTTGCCCCTAGGTGTCTGTCCTCTGAAATATTTAAGGTGTTCCGCTTCACTACGTAGAAGAGAGGATGTACAGCCGTTGGTTGACAAAATTTTGAAACACATAGCTGGCTGGAGAGGCAGGCTCGCTAGCTGTTTATCTCCTATCTTTTATAAATTTCCGAAATGGGGAATTCATATGACCAACACACAGATGGCGAGCAATGTATAGTAATAACAATCAGAAGTTCCGGCCACTTGGTAAATTGGCCTAGGTACAGTCTGTGTTAAGCAGGAATATTGGCATACCATATCCGAGACATCTGAACATTTGTCAATGTGATGGTACAAATTAATTAACTCTGCTAGCTAGCGTTAATTAATGAACAAGCCAAAAACAGAAGAAAAATTAGGTAAACAAATGGTCCCTATCACCAAATAATTAGTTGAATCGGCGGCGGTAGTAGCTAGGCACTCGGTCCAACCGGCTCGTCGTAGTCCTTCCACTCCAACCTCTCCCCGTTCTTGATCCTCCTCATCCTCTCCATGACCCCGTCCATGGCGATGTCGAACGCGTCCTCCATCTCTCCCTGGAACGCCGCCGTGGGGTCCTTGTACACGAACCTCGGCATGATTCTGATCACCTCCTCCCGCATCTCCGCCACCTGCTCGTCGGTGTAGCGGCTCAGCGCCTCCTCCACTTGCACCTTCCCGCTCACCACGTCGTCGAGGTCGAACAGCACGTAGTACCTGACCCGCCGCCGGCGATCGTCCGGGTGGTCGTGCCACCACCGGTACTGCAGCCCGCAGGGTGGAGAAGCGGGGGAAGAAGACGGGGATGCAGCCGGCTACGACGGCGTCGATGGCGGAGCGGCGCGTGAACGAGTCCCCGGGCGGCTGCAGGCAGAACCGCGCGGAGGCGAAGAGGTTGACGAGCTTCTCCGCCGTGCGGCAGGAGATGGCGCCCTCCAGGCCGTTCGCCGCCGCCTCGCTGCACAGGTCGAACACCTTCTCGCGGATGGGGAGCTTCCCCTTGGCACGCTGCGCGCCGGCGAACGCCATGAGCAGCGGCCGCGGGGCTCGGCGCGCGAGGGCCCGCCCAGNNNNNNNNNNNNNNNNNNNNNNNNNNNNNNNNNNNNNNNNNNNNNNNNNNNNNNNNNNNNNNNNNNNNNNNNNNNNNNNNNNNNNNNNNNNNNNNNNNNNNNNNNNNNNNNNNNNNNNNNNNNNNNNNNNNNNNNNNNNNNNNNNNNNNNNNNNNNNNNNNNNNNNNNNNNNNNNNNNNNNNNNNNNNNNNNNNNNNNNNNNNNNNNNNNNNNNNNNNNNNNNNNNNNNNNNNNNNNNNNNNNNNNNNNNNNNNNNNNNNNNNNNNNNNNNNNNNNNNNNNNNNNNNNNNNNNNNNNNNNNNNNNNNNNNNNNNNNNNNNGGAAGTAGCTCGGGTACGGCACCGCCAGGTCGCGCGGGTGCGGGACGCTGGACTCCACGGTCAGCGCCGTCATGTTGCGCGACTCCGGCTGGAGGAGGAAGCTGTTGCCACACCACCCGTCGGGGCCGCCCGGCTCGCGCCGGAACGGCCACATCGTTCCGCCGGTNNNNNNNNNNNNNNNNNNNNNNNNNNNNNNNNNNNNNNNNNNNNNNNNNNNNNNNNNNNNNNNNNNNNNNNNNNNNNNNNNNNNNNNNNNNNNNNNNATGGGTCGCCGGTGAGGCATTCGTATCGACGCATCCGGTGGTGGAACATCACCTCCAGCGCGAACTGGTCCCTGCTGTACCTGCAATTAACGGTACGTACGTCGACTCCTTGATATATATGCATCTTTTCTAATCACAAACGTAACTCCATTTTAACAACCATGCGTTAATTGATCGATCGATGAGCAATCATGCATGCATCAACGTACAATAACTAGCTTAATATATATATANNNNNNNNNNNNNNNNNNNNNNNNNNNNNNNNNNNNNNNNNNNNNNNNNNNNNNNNNNNNNNNNNNNNNNNNNNNNNNNNNNNNNNNNNNNNNNNNNNNNNNNNNNNNNNNNNNNNNNNNNNNNNNNNNNNNNNNNNNNNNNNNNNNNNNNNNNNNNNNNNNNNNNNNNNNNNNNNNNNNNNNNNNNNNNNNNNNNNNNNNNNNNNNNNNNNNNNNNNNNNNNNNNNNNNNNNNNNNNNNNNNNNNNNNNNNNNNNNNNNNNNNNNNNNNNNNNNNNNNNNNNNNNNNNNNNNNNNNNNNNNNNNNNNNNNNNNNNNNNNNNNNNNNNNNNNNNNNNNNNNNNNNNNNNNNNNNNNNNNNNNNNNNNNNNNNNNNNNNNNNNNNNNNNNNNNNNNNNNNNNNNNNNNNNNNNNNNNNNNNNNNNNNNNNNNNNNNNNNNNNNNNNNNNNNNNNNNNNNNNNNNNNNNNNNNNNNNNNNNNNNNNNNNNNNNNNNNNNNNNNNNNNNNNNNNNNNNNNNNNNNNNNNNNNNNNNNNNNNNNNNNNNNNNNNNNNNNNNNNNNNNNNNNNNNNNNNNNNNNNNNNNNNNNNNNNNNNNNNNNNNNNNNNNNNNNNNNNNNNNNNNNNNNNNNNNCCCGCCGTTGACGGTGATGGCACACCAGCTCCAAATGTCGTCGAACAACGGCGAGCCCTCCACGCAGTCCTTTAGCATGTTGAACCGCTCCGGCACGTCCACCATGTACACGTACCGTCCCTCGCATGATGGCTGCCGCCGCCGGTCCTCAACTTCCGCTGCAGGTGGAGGGAGGAAGCTGGTCGCGTCGAGGGCACCGCCCGTGGAGGAGGATAACGGCGAGAGGTGGAAGATCAACTCCAAGGTGAGGACCCATGAGCACACCATGAGGACGTACTTCTTGGGGTAGTGAGCTTCCATGGCTGGATAGATCGATTAGTGGTACGTAGCTGCCTCCGTCCACAGCAGCTCGATCGATCTGGGGCTTTCTCTAATTAGTAGCTAGGAATCTGACAATGGAGGGTTAAACCTTTGACCTTGAGATAATAACAACTTAATTTACTACGTGCTGGGGCTTTGTTTATTAATATATATATATATATATATCGGTACCTTATGCAACCTTTGACCGTGGAAACAATAAATATTGGTGCTTAGGGTAACTTAAGATGGTAAGACCAAAAACTAGTGTGCATCATGTGTGTAGAAACTTTGGGCAATACAACAAGTTATGTAGTAGTATATGTTTCTTTTATTGCTTTCGAAAAAAAAATGTTTCTTTTATTGTGCTTCTGAATGGTAAGCAGGCACTACTAGATAGCTAGTTTGGATCCATCTGAACTTCACCTTGGGTTCATTAATGGTGCCTTTTTTGTGTGTAACTTAATTTTTCTCTCGATTTGTGTGTTGCTTAATTTGGGGTTCTCTATATGACCACTTGTGTGGAGCATGTTGACATGGACCAGCTAGATTACTTATACCAGTATATGTCACATCAAGTGGTGATGAGGTAGGTAAACATGATCAGTACTCCCTCGGACCTTGAAATCTCGACGTTGAAGACATATCGAAACTAAAAAAACACTAACCATACCAAAGGAGTAACGTGTTTACGTAAGTGTCGGGTATTTTGTCAACTTAGAATTCTAGATGCATTGTGACTTGCTTCCCTGTATTTGTGATAACGAAAATGCTTCACGTACAGCCGCTGGATGATTGGACCTGATTATTGATAATCGCTCTTGATGAGCTGGTCGCACGGCCGGGTCGGAAGACCCAGTCGTGTGTATAGCAGCGTTCTTGTGATAAGTCCTACCTCTGCAACCCCCCCCCCCCACCCCCTACAAAACCGGTATATTNNNNNNNNNNNNNNNNNNNNNNNNNNNNNNNNNNNNNNNNNNNNNNNNNNNNNNNNNNNNNNNNNNNNNNNNNNNNNNNNNNNNNNNNNNNNNNNNNNNNNNNNNNNNNNNNNNNNNNNNNNNNNNNNNNNNNNNNNNNNNNNNNNNNNNNNNNNNNNNNNNNNNNNNNNNNNNNNNNNNNNNNNNNNNNNNNNNNNNNNNNNNNNNNNNNNNNNNNNNNNNNNNNNNNNNNNNNNNNNNNNNNNNNNNNNNNNNNNNNNNNNNNNNNNNNNNNNNNNNNNNNNNNNNNNNNNNNNNNNNNNNNNNNNNNNNNNNNNNNNNNNNNNNNNNNNNNNNNNNNNNNNNNNNNNNNNNNNAAGACCTCCCTCTGCAGCCCCCCCCCCCTCACCCCCTACAAAACCGGTATATTCTAAACATCACCTACACCTACGCATGGGGTATATTGAAAGAAGTAGGAGCTACTCAGAATGAGGGTTAAAAAAAAACTCTATCTCCGTTTAATTCCTAATGAATCAACGGTTGATACACAGGTGGGGTACAATCTAGCAGGACAAAGGTATATTTTTCGCAACTAGTTCTTTATTGGTTTGGATTCGTCATAGAAGACCACATTAGTTGTAGTGTAGTAATATTCTTGTCCGTCATTAAATGGAGGTAACACCATTGCTTGATTTTTATGGCTCCGCCACTGCATAGAGCACTTGTTATTAAAATTGTTATTTGAGTCTATTGTAAAAAAATATTTTTTAATATGTAGGTCAAATTGTGCTTTGACTATCAACTCTGATGTTTTGTGAAGTAGACTGAATAAAGTATTCGTGTCATATACTCGGTCGTTCTCTCCACCGCTTCGTCTCCAACCCGCTCTCTCCCGTGAACAGCCCAACCTCGCTGCCTCCGCCAGATCACATCCGGACCCCTCTCTCCCTGCCTCTGGCGACCTCGCCGTCGGCCGGCGAGTGGAGAGGAGCCACCCTGCAGTAAAATAAAATTGTTTAGGTTGTTATTTTCAGTTGGTTTGTAGTTGGGCTCGTCCCGCATGTTTCTTTTTCATTTCATATTATTTTGGGCGGTCGACGGTCGTCATGGTCTTTCTACGTGGTGTCGGCGAGCTCGACGGTGATGACGGTCGCCCGATGCTCCTCTTCGATAAAACAAGCTCCTTCCGGCAGATCCACGCCCAAGGGCGCTGGATTTGTGAAGACGGTGACCCGATCTGGCCCCCACGCCCCCCCAGTCTTCCATCCCCTTCACCGGCGTTCAACAACTTCGCTGGTCCTATGTCGCCAGATCCGCGCCTTCTAGTGCGGGTACGGCTGGGGTGCGACCAGATCCTCCATTGCCGCGCATCGACCTCCCCCTCGTCGGCCTCCGCATCGCTCCACCCTGGCGTCCAAGGGCTTCACCGAGGGTGAATTCATTGAGAATGGCGCTTCGCCGCCTTCCTTTACTGCAGGTCCACCTCTCCTTGACGCTATCCCCTCCGCCAGGCCGACGTGCTCCCCTCGGCTGGCTCGCGGAGCACCCAATTGGTCGCCGTCGTCCATCCATGGAGTTGGAGCAGTAGATCCTCTTATAGGGTTTCATGTAAAGGTGAAAGCGAGAGTTGAGGAGATGATGATGATGCATCATTCCTTTTTTTTCTTGGTCAAACAGAAAAATGTTTGAATCATCCCTTGTATACGAAAATGGTGTCATGCAGCAGCTTAATTTGTTGCAGTATAATCCAAAGCATATATTAATCGGAGATCTCCATGCAACAACAGTATATTATGAAGAAATTGAAACCATGTCGCCTCAATTATGAAGTCGCGCGAAATACTGATCTGCAAACCTGCAGCACAAGAGTGTACCATGGAGCATTGGAGCTAGCGAGCCATATACCGATCGACGTGGACGCATCGTCAAGCGGTGGCGCGGCCAAGTTGAGTTGGGGCAGCGGCGCCATGCCGGAGTGGTACCCGAACACGATGCCGAAGTCGTCGTGCACCTCCTTCCCGGCAAGCTCCACCCGTCGCGCCGGCGCAGCAGCTCGCGTCGCCACACCTGCACCAACGACCTCGCGCGCCGCCCGCAGCGCCAGCCCGACGGCCTCGTCGCCGCCGGGGTCCATCCCANNNNNNNNNNNNNNNNNNNNNNNNNNNNNNNNNNNNNNNNNNNNNNNNNNNNNNNNNNNNNNNNNNNNNNNNNNNNNNNNNNGCGACGTCGTGAGCCAGTGCAGGCGGCCGCCCGCGTACGCCGGCGGCTTCGTGGCGCTGAGCTCCTCGTACGGCGCCATCCGCAAGCTTCCACTGCGACCCGCGCCACCCGGACCCGGCCGCCGCCGACGAGANNNNNNNNNNNNNNNNNNNNNNNNNNNNNNNNNNNNNNNNNNNNNNNNNNNNNNNNNNNNNNNNNNNNNNNNNNNNNTGGCCGGGCCGCCCGGGTCCAGCGCCGCCAGGCAGAGGAGGCCGCTCGTGCACGAGCAGAGCTCCAGCCTGTGCCCGGCGGCGACGGGCAGGAACGAGAGGCGTGCGTCCCGCGGCGGCGAGGACCTGGAAGACGCCGTCGTGGAAGCGCGCGAGGCCGGCGGAGATCGGCGAGGGGCTCTGCGCCTGCATCGCGGCGAAGGTGGCGTCGCTGCGGAGGATGAGGTCGCGCCAGGACTTGCAGACGGCGGCGAGGCGCGCCAGCGTCCGGAACCACTACCTATGTAGGCGGAGAGAGTGTATTGCATAGCTAGATTGAAACCCTATTTAGTGGGTGAAAAATACCACTACCTACACTTGAGTCATCTTCAAAGTCATAGATACTTTTAAGGTTCATTTTCTTGGTGAACCATAGATACTTTACCAGCCAAGAAAAAGGATACTTCAAGTTACAGTGTTCTGGTCTTTGGGCGGACCTACCTTTCTTGCCTCCACCCCCCCCCCCCCCCCCCCAAAATGAAGTCTTCTGGATTCCAATGTTTTAGTGACTTAACAGAGTTGAATCATATGAAAGAATAACCTTGCCACGTGCCTACATCAATCGCAAATTCAGAAAGGCCCAACCAATTTGTAACAACCTCCTTTGGAGCGGTAGCACATATATCGTGTATTATAAGCAGCAAGAGACAAGAGTCGACTACAACACATCCTTTAATTTCCCCAAGAAAGAACGCCAAAATTAAACTGCACAGTACAATGCATCTTTCCTCAGGTATCCCATATCATGACGACGGCAATGACCGCGACGCCAAGAACGACGGCGAGCCACCTGCCCACCGGCGTGTCGACAGCAAGCGGCCTCCGGGGCTTGTAGCCCTTGGAGAGGAGGTGGTTGATGGAGACGTAGATGAAGATGCCCGTGGCGAGGCCCATGGAGATGGCAAAGATCCAGTCGGCCACCCGGCCCTGCGTGGTGGCGTCGATGACGATGCCGATGCCGACACCGATGGGGCTGGAGATGGCGAAGGCGAAGGCGTAGGCGAAACACGAGANNNNNNNNNNNNNNNNNNNNNNNNNNNNNNNNNNNNNNNNNNNNNNNNNNNNNNNNNNNNNNNNNNNNNNNNNNNNNNNNNNNNNNNNNNNNNNNNNNNNNNNNNNNNNNNNNNNNNNNNNNNNNNNNNNNNNNNNNNNNNNNNNNNNNNNNNNNNNNNNNNNNNNNNNNNNNNNNNNNNNNNNNNNNNNNNNNNNNNNNNNNNNNNNNNNNNNNNNNNNNNNNNNNNNNNNNNNNNNNNNNNNNNNNNNNNNNNNNNNNNNNNNNNNNNNNNNNNNNNNNNNNNNNNNNNNNNNNNNNNNNNNNNNNNNNNNNNNNNNNNNNNNNNNNNNNNNNNNNNNNNNNNNNNNNNNNNNNNNNNNNNNNNNNNNNNNNNNNNNNNNNNNNNNNNNNNNNNNNNNNNNNNNNNNNNNNNNNNNNNNNNNNNNNNNNNNNNNNNNNNNNNNNNNNNNNNNNNNNNNNNNNNNNNNNNNNNNNNNNNNNNNNNNNNNNNNNNNNNNNNNNNNNNNNNNNNNNNNNNNNNNNNNNNNNNNNNNNNNNNNNNNNNNNNNNNNNNNNNNNNNNNNNNNNNNNNNNNNNNNNNNNNNNNNNNNNNNNNNNNNNNNNNNNNNNNNNNNNNNNNNNNNNNNNNNNNNNNNNNNNNNNNNNNNNNNNNNNNNNNNNNNNNNNNNNNNNNNNNNNNNNNNNNNNNNNNNNNNNNNNNNNNNNNNNNNNNNNNNNNNNNNNNNNNNNNNNNNNNNNNNNNNNNNNNNNNNNNNNNNNNNNNNNNNNNNNNNNNNNNNNNNNNNNNNNNNNNNNNNNNNNNNNNNNNNNNNNNNNNNNNNNNNNNNNNNNNNNNNNNNNNNNNNNNNNNNNNNNNNNNNNNNNNNNNNNNNNNNNNNNNNNNNNNNNNNNNNNNNNNNNNNNNNNNNNNNNNNNNNNNNNNNNNNNNNNNNNNNNNNNNNNNNNNNNNNNNNNNNNNNNNNNNNNNNNNNNNNNNNNNNNNNNNNNNNNNNNNNNNNNNNNNNNNNNNNNNCTTGGTCTCTGCAAGTTCGTTATTCAGTTTTGAAAGCAACGGAAGTGTAAGTAATAAATCATGTAAAATTTTCACGTGTCAACTTGCGTCCAGATGCTGTACGGTCCAAAGCTAGAAATAAGCACGGATTTGTTCTGTTGCATGCAGTGGGTTCTGAAATGCTGATTGAAGCCACGGTGGTGCTAGGAAGGAGCACTTTAAATGTGTAGAGAACGGCAACGATGTGATCTTGACAGGTAGCAAGATCAGTAAGTTAGTTAGGTTCTTAAGGACGCGTTAGGCATGAATGCACAGAGCTTCTGGATGGGCATATAAATCCAAGGCTTGCTTTTCTTTTGAAATTTTCTGAATCGAAGACTGGAACAGGTAAAAGTATGCATGACAATGATAGAACTGAAACGTTGCGTGAAGTTCAGAGCATCGGATAAGGTAGCCTAAAAAACGACACTGTTTGGTAGAAAGATCTGCAGATTATTCCGGCTGTACTGAATCTTTCTGCTGCTTTCGCTTTGACGAAACAGCGGGATACTAACGCAGCCTGGACAAAACGCAAGGAAAGATCCCACTTGGTCTTTTGTCACAACCATGATCACTATCGCAAGAAGCTAGCTGAGAACCGACGAATGTTCAGTTCAGTTCTTCAGTCTACTGAACTTTGCAATCAAGTCACAATTCCACACATATCATTTTATACCAATCGTGGAGAACCAACTATTCTAGTCAGACTAAACTAAGCTCGGAACCTTCACAGGTGACATGATAGAGACGTCTTCTAATAATCAAGAAATCAAGCAGGAGGAGACGAGCAATGCAAGGAAGCATCCATATATATCACTATCAAGTTTAGCCTTACCGGCGACTCCGATGGCGATGGCTTCGNNNNNNNNNNNNNNNNNNNNNNNNNNNNNNNNNNNATTATACGAAGTTATACGAATATATATCACTATCAAGTTTAGCCTTACCGGCGACTCCGATGGCGATGCCTTCGAAGACGGAGTGGAAGCAGAGGGCGGCTATGAGCAGCACGCTGTCGCCGAGGGTGCTGGCGTTGCGCAGCATGGACGCGACGTTGTGATCGGCCGACGGCCCATTGTGCGCCTCCTGTAGGTGAGCACAAATAATCAAGCAATGATAAGAAACTGAATTCAGAAATAACTAAACATGGTCTGACTTGGTCACATGACAAATTCATTCAGGAACTGAACTTACAGCTGGCTGCGGCCCCGAGCTGTTGCCATTTGTGTTGCTCACCTTGCCCTCCTCCAAACCTATTGGAAATTACCAAATGGAATGGATCGTTCAATTATGGGAATCCTAAGCATCAAATCAATACAGCTCATGGCGTATCCAAATTCAGCAAGGAGGAGATGTGACGATCAATTGACAGTAGGCAGGCCAGAGAGCCTTCCGTGATAACGACTGGACTATTGAAACTTTTCCGTGTATAGACGACAATGATCTCATGCTCATGTTTCCATATTATTGGAAACTATCAGAGGACCTACGCGTTTCTATCCATCCAGTCAAACAAAACTAGGCAGTAAAAATCTCTCATCGTGTCGATCATAACCCAAGAACAGCAGGACTCAAGCATACTGCAAAAACCTGAACTCAGTTTCTCATGGAAACAGAGTATCAGTACGGTAGCACTACTACTGCATTCCCTCCCGTAACCCAAGAAACAGGACTCCTCTGGCATACTGCAAAAACCTGAACTCGGTTTCCCATGGAAACAGAGGATCGGTGGCACTACTACTGCATTGCCTCCCCTAAACTGCAGCAAAGGCCATTGCTTACTTCAATTTCCCGAGGCCAAGATTCAGGAGTGGTAAAACTGTGACTGAAAGAAACGAGTAAATTTGTTGTACCCCAACTGAATGAGGCCAGAGTCCCCACCAATCCGAACAACCTGTGGCCAGCAACAGGAAAAAGCTGGTACTACTAGTGCAGTTAACCCAACCTCCCACCGGCCACCTTGATTGATTCACACCGGTACACCGCCCAGCTCACCCGCCGCTGTCAAAACACAGACCAAAGAAGACGAAGACACGTGACCGTGTCAGGCGCGCGCTCACCTGCTGCGCTATTAACGCCGGCGCCGTGGCGGCCCCCGCCGCGGGAGACGACGTGCGAGATGACGCAGTCGGCGAGCATGGTGACGACGTATCCCGCGCAGGCGAGCATGAAGGCGTAGGGGTACCCGGAGTCCGGGAGCAGGTCCCCGAAGGTCTCGTCGGCGTCGCTGAGGAAGTGCATGAGCGCGGTGCCGAGGAAGACGCCGCCGGCGAACTGCGTGCCGAGCGCCAGGAACGCCTCGTTCCACCGCATGAAGTAGGGCGACACGCCCCCCGCCAGCGTGCCCGCGAACACCACAGCCAGGCACCACAGCTTCGCCGCCACCAACCCCCGCGCGCGCAGGTTTGGCTCCGCGTCGGAGTCCGAGTCGGACGAGCCGCCGTGCGCCCACGCGGTGGTCGCANNNNNNNNNNNNNNNNNNNNNNNNNNNNNNNNNNNNNNNNNNNNNNNNNNNNNNNNNNNNNNNNNNNNNNNNNNNNNNNNNNNNNNNNNNNNNNNNNNNNNNNNNNNNNNNNNNNNNNNNNNNNNNNNNNNNNNNNNNNNNNNNNNNNNNNNNNNNNNNNNNNNNNNNNNNNNNNNNNNNNNNNNNNNNNNNNNNNNNNNNNNNNNNNNNAGTCGAGCATGAAGGCGTAGGGGTACCCGGATTCCGGGAGCAGGTCCCCGAACGTCTCGTCGGCGTCGCTGAGGAAGTGCATGAGCGCGGTGCCGAGGAAGACGCCGCCGGCGAACTGCGTGCCGAGCGCCAGGAACGCCTCGTTCCACCGCATGAAGTAGGGCGACACGCCCCCCGCCAGCGTGCCCGCGAACACCACAGCCAGGCACCACAGCTTCGCCGCCACCAACCCCCGCGCGCGCAGGTTTGGCTCCGCGTCGGAGTCCGAGTCGGACGAGCCGCCGTGCGCCCACGCGGTGGTCGCCCACGCGGTGGTCGCCCACGCGGCGAGGGTGAGGCCAAGGAGCAGGAGGCGGTGGGCGGCCATGGCGCGCGGCGGGGATGGAGGATGGACACGCGCGGTGGTGGAAATGAAATGGAAAGGCGGAGGAGTTCCGGAGACGGGGCGGGTTTAATTGGGAGGTTTGGATGAGGCTGACGCGTGGGGCCGGTCGTTGACGATGGTTGGTCGCCGTGTGTTTGGAGTACTTACTTTGTACTGCGGCTGGGATGGGAGGGGGGCAGTAGTACAGACAGGGACGGGGTTTGGAAAGTCCGTGACGGCCGGAGTGTCTGTCTCTCGGGCTGCTCCGACGACGACGGTGATTTCATCTCAACGGGAACAAGCCTGGATCTATCTATCCCATCATCCCTGATTGAACGCTGCTGCACGGTGGCTGCGCCATTAACCGGCACGTCCTTCCACGTCCACTCCATTACGACAGCCATACCGGCCGGGTTGTTCTTCGCAACTCAGCCGGGAGACCTACGACGGGCTAGTGCAATTTGCATCGCTTCAATTCATTGATAGGCGCGTTTCATTTTGATTGACGATTATATTGCTGTGCTTAGGCCCCGTGATTTGGACGATCCCGGCGTCTGAGTTGATTAGCTGCTTGTTTTTCTTTCCTAATTCGCAAGAAAAATCAACCGGAGGCTCGCACAGGAAGCAAATCTGACAGATGCAAAGTAGACGAATATAGCTGGTAGAATTTATTTACTCACTATAATGTCACAAACATATAATGGCATGCCGGCTCATCAGGAAACAAATAAATAAGGAATCATGCATGTATTGGCTCATCAATCTCTGTTTGGTTGAGTTGTGCAAGCAAGCATATTAAAAGTGCGTGTTGCGTGCCTTTTCCGGCCATCCACCAGGGACTATTAACAAACAATGCTTACTTATATTCTTTTCCATATATCTGATGAACAGAACTGACACATTGCGTCAGCCGTCAGACGACGACGACGACAACGAAACGAGGACAGCGACCAAGCAGCAGTAGGTGTGAACGATGCACGGCTCTTTGGCCCGAGGACTACGTTAGGCCTGCATGCTCGCTTTGCTTAATTATCAACAACTACCCACGCTACCGCCCTCTCCTGTTTGACTGTTTCCGTTCATCCTATGCTTTGCTTATCTAAAAAAAATTCTTATCCCACTTCCACCGCAAAAAAAAAAAAAAAAATCTTATCCCACTTTTTCGAATGAAAAAGTTTTTTTAGTTGCTTATCTACAACTGGGTTGAAATATTGGCCTTTAGATGGGTTGACCGAGCAAAACATATGACGCTAACAAAGGCCGGCCACACACTTTTGACGCTAACAAATCTTGTTCGATGGCCAAGCAAAAAGCTCCGACGCAACATGGAACAATTTGAGGACAAGAGTGGAAAAAAAGATTAGAATACGAAGTCGAACATTTTGAATCGCACAATTTGTAGAAAGAGGCCTGAATAATTTGAACAGAAATTAAGCCTGAACAATTCGAATCAAAAGTGGAAAAAATAAACCCCTTATGGAGCAATCACAACTTTATAACACGAACATAGCATGATGAAATACATATTTTTTATTATGTTCCGTTGTGTATCTTTGATGCTTAATCACAAAAAAGGGTGTATCCTTGATGCTCTCTTAGCACATGAAATATTGTCCAAGTATATTCTATTTTCCATTGGGTTATGGTAGTATTTCAGTAAAAAATTTGTACTGTCTTCACTATTTCATGGATTAAAGAAAAATAGGAATGGAAGAACACTGCTCTAAAACGAAAGGAGAAATTACAAAGGTGGATGCATTTTTCAAAAACAGAAAAAATGATCGCTAGCCAAGAAGTCAAAGAAAATATCTGAACTCAATGAAAATAAAAGAATGGAATAAAATTTAATTTTGAAACAAGTTAAAAATAGAAGTAAACTAGACTCATATAATAATTTGGAATATATAACTCACGTAGATTACAAGCTAAATGTGCCAAAAGGATCTTGTAACTACAAATAAAATTTAGATAAAACAGTGGAAAAGAAAAGGAACTAAGCAGAATGAAAATAGACCCATGATTTTTTTTTTGAAGTGGAAATAGGCCCATAATTTACCTGGACTGTTATATATGGGCCTAAGCGTAACTGAAACAGAATGAAATAGGCCCATAACACAGGTCCGGTGGCACGTAACGTTTGGCGGAGAACCCGCCGCCGCGGCATGAATTCCGGGAGCGACGGGCTCCCGATTCGATCCGTGCTCGTTCGGTTGTTGTCGCCCTAATTCTCCCATTCCTCTCGCTCGCTCATCCCCAAACCAAGCGAAGAGGAGTGGAACCGCAGATCTGTTGGCCGCCGCCGCCGCAGGTCCAATCCGGTGGCGATGGGGACCGAGGAAGATATGGCTGGTTTGCCCGAACCGGTGAAGGAGAGTTTGCGCGCCATCTTTGCCAACGGGCGGCGCTTGCATAAGCTCCAGATCGAGGACCCCGCTGCTTTTGAACTCGAGATGCAGAAACTGGAGATGGAGAGTTCCGACGAGGAGCCTGATGAGGAATTTTATCGGCAGCAGAAAAAGGAGTGGGAGGAGTATCCCGCACGTATGAAGGCTCTGTTTCCCGATGAGGAGGCCGCCGCCCTGGTGGATCATGCCCATGACGAGGCCAAGGACTCGGTAGCTGACCTCCCTGAGGAGGCCATCTCTGCGAGGCCTAGCACCCCTCAGGATGCCGCCTTCACGAGGGCTGACGCTGGTGGTCCCCAGTGCGGCGGCCGCAAAGGAGAAGCCGTCGATCGCCCTGACGAACAGGTGAAGAGGCAACGCGTGTTGGCTTCGCCTGAAATTGCCCCACCGTTCGATAGGTCTGCGTTCAAGTTCTGAGCTCTCTCGTCAGTTAATTAATTTTGTCTCTATAATATCTTTAGTTGCTTTGTGATTAGCTGATGTGTTGTTGGTGCTATACAGTATAATCCTTTCGGCCTGTGGCATTGCAGTTTGGGAATTTAGGAAATTAGAAGTTTCAGAATGTATGCTAGTTCTCTTTTGTTTTGACTGGTTGGGGTGTTGATCGATTGGTGAGGAAAAAGATGGTTTGTGTTCTCCTGCCACGATTGTTTTGACTGTGTGTGGTGTTGCTTTGCTTGTTTCCTTGCTTAGTTTTCACCTGTTGTTTTGCAAACCTACATGGGATTTTCTAGGGATTGAACTCCAGTTTCGTTTGCTTCAAGCACATGTTACCTCACAAGATCTAGTAATATAGACTATTTGTGTACTTACGTTTTTAAGGACTGGAAGTTTTTACAGTAACTTAAAAGAATTAACTACAGTGACCTGTATCTTTGGTTTTGTGGATAGATAAACAGTTAAATATTGAGTATACTCAAAACCAATGTCTAGTTTTTATCTGAGGTTCGGTTCATACACTTTTATTTGGTCAGATTTGTTGTGTGAGAATGTATTACAAATGTGTTAAAGTGGTTATCTAGGTTTTTGTGAGCTTAGCTACTCAGTGTTGCTATTTATGCTTCATATAGGTTATTGAATCTGTATCTGTGAAGGGTATGGATACTTCAAAGAGTTCTATTTGTTGGTATCTTGATAATTTTTCATGTCCTCATAGTCGGCCCCCAGAAGTGAAGTGGGCTGAGGCGATGGACGAGATTTACATCACCGTACAATTACCTGATGCAAAGGATACTATAGTGAAGTGGGCTCAGGCGATTGACAAGGTTTACATCACTGTACAATTACCTGATGCAATAGATACTACTGTCAATTTGGAACCTGAAGGTACTTTCACAATCCGTGGCAACGATATTGGGGCTGATGGGCGCTTGTATCACCTCAATCTTGATCTGAATGACAAAGTAAACGTTGAGGTAATGCATTGTCTGGATATACTTAATTAGTTTTCATTCTTGTTGAAAGGCTGCATTGTCTTGTGGATAGACTTAGTTTTAATTCTTGTTGAAAGGTTGCCTTACTTACCCAGAGTTTCTTCGTTTTGATTTAACAGGCTAGCAAAACAAGTGTAGGGTTCAGATCCATAGTCTGTATTGCCGAAAAAGGCGAGGCAAAATGGTGGAAGAAGCTTGTTCGAGATGACCAGAAGGTACCTCGCTCCATGAATAATGATGACGGGGACAAATAGGTGAATGAAGATATCCACGGTACAATAGATGTATTTTCCTCTATTTTAATTTTGATACTAGTGGAAGTCAAATATCGTGATATGCAGGAATATTCTTTGCACTTTGCTAGACGTTTGTTGGAAAGGGTGTGGTTTGGAACTTGTAGATTCATCAAATGAAGATGTTTGTGTGATGTGGACAGACACTAAACTAGCAGCTGAATAAACTTTGTTGAAAGTTGAGGACCAAGTGACAATTGCAAATTAATATTCTTTTAAAGTTTTTTTGTTTCTTGTTTGTTAAATGATATATCATGGTTTGCACAATACACAGTTTCATTTTATGTTTCAGTACTACTTATTCTATAGTAGAAGTTTCAATAATTGACTAATTTATTTCACAAGATTTTATATTTTCATGACCGGATAGAGTTTGTGGAGTGTAGTGGGGAATTGTGACTCATTGAAAAAGTTAGAATAAGGATAGCTTCTTATATGCTTATACAAAATTAACTCTATTCAATATTCTCACAATTTTTGTACTTTTTGCTCCTTTACTAGAAGAAGCGATGAAACCCGAAGCTATTCAGAAGGTTGAGGAGGCCGAAAAGACAAGAGGCAAAACCCAATTTTTAAACGACTTGTAATGGCTGAAGGCATGCTACGTGATCCTTTGTTCAATTGTGACTAGCAAGTTTTGGCGCGCTCCGCTGCGCCCGGTAGATATAGTACCATTTTTTTATTGTGATCCAATGCAAAGGTTCACACGAAGGCCATTTCAAAAGAAAAAAAAAACAGAGAGGGGTTCACATGAAGGTTTGCATGCCATGTTGGTCGCTTGTTTGATTCCTGTACGGTGGCAAAGGCCTTGTTCTCGGTTGGTTTGGAACGCGAATACACAAAGCTAGAGAGCTTTTATGCAAATTTGCTTGGACCAAGACATGGACGCACCATGTCCACATCTTTCTCAGCCTAACTTGGTGCTCTCGGCCTCCTCCCTTCTTCATTCACTTCACCACTCTTGGTCATGAACCTTCTTTGGGCCTGATTGGTTGGTTGTATTTGGACTAACCAGGCTCTAGGGATGCAAATATGCACTGATTGGTTGACTGAATCTGAGATAGACAGTCCGGTAGCATGCAAGTGCCAATTGATTGTGACTCTGGGAATGCAAAAAGCATAGCTGAGTCTGGCTCGCCAGGAACGGAAACGAATCTCGTTTCTGCTCAGCCTGGCTCGCTTGCTGCTGGCTAGCGGTGGTCTGGCCCCTTACATGCAAGCCTGGCTTCGGTCAGAAGCAAACCAATCGTGCCCTTCATATCTCTCTCACCGCCTCCTATGGATGCAACCGGTCCCAACAATCTCTCGACATCCGGCAGCCCGAAATAGCAAAATCGAAAGCCTATGTCCCTCTCTCGTCCCCTGTTTCCTTCCTCTAGCTGTTCCTCTTTCTGCACCATAGAGTTCAGTTTGTTTATTTTAACAATATAGCTCATCTTGCCGCTGCAATGGAGTAGGACGCCGGTGGAGGAGAGGGAGAGGGGTCCCGTGCAGCGTGGGGCTGCTGCTCTAGAGGGTGTTCATGCAGCAACGCAGGGCTCGCGAGGAGGCGGCAGTGCGAGCGGTAAGCTCGACGGCTTGGTGGAGCAGAGCAGCGCAAGTGGAACGGCAATACGACGGCTGGAGGTCAGTGGTAATTTTCTCTTAGATTGATTTCTTCCTCGCTCTACATCCTTCCATCGCGCATGAGGCAGCAAAATGAACCAGGCAGCTTCAAAATGGTGGTCGGGCACGAGCTCGCCTCCGGCAGAGGAGCACATGGTGAAAGCCCCACAATAGCCACCAACGTATGGTAGTGACGTGGTGGCCGGAGCACGTCGGTAAAAACGCCCACAGCGTCGGAAGTCTCACCGCTATTCAGCACTTTACTATGAGTTCCACAACTACTTTTCTACATGGGAGATGAGCGTTTCTATGGTAGTTTTAGGACAATTTGCTTAGTTTTGGCAATTTGTTCTATAATGTCTAATTAAAAGTCAAATTAGAGGTCAAGGGCAAGCTCATAAAAACCACTCTCTATAGCTAGGTTCTCACTCCTAGGTTCTGAGGGGCTCAGACTGACTCCAACAAGGAGGAGATAGGCAAATTGATGTTTGCCTCTCAAGTACAGTGTTCTGCATGTGATTATCGCTCCAACAGCTACTGCATTTTCGTCACGAATCCTCTCTCTCTCTCTCTCTCTCTCTCTCTCTCTCTCTTGTAGCTCTCCCTGCGCCCCGGCTGACGACGGCAAGTGCGCGTTCCGGCCAAACGGAGGGAGAAGAGGGAAAACAAAGGGGTGCACAAGCATCATCTCATTGACGCGGAGCTCTGGACATGTGTGTCGACGACGGGGAGGCCCAGAAGAGGGAGCTTGACAGATGGGCGGCGACGACGGTTAATCGGCGGCGGGGAGAAGACCAAATCAGGCGATATAAGCTGCAGATTCGGAGGGAGATGTTGTCTGAGAGGTGTGCGAGCTTGATGCACACTTTGAGGTGCTAGGAATCGAGACGAGGCTCACCGGGTGGGGCGAAGTCGACGACGGAAGCGGCGACGGTAACCCGGTGGCCATGTCAATGAAAAGCTTTGATTCCTCAGCTAGGCGAGCAGAGGAAGAAAGGGAGCAGTGACACGAGCTTCAGCTTATCCAAGCGCAGCTTCCCGCGTAATGGCTCTCGCGGCAAGGCACTGACGTGGCTGAAAAGCGACCCCGTGTCACAGCACCGCCACGCCCGACATGCCACAGGGGTCTGTCGCCGCGCGGTGCGCGCTGATGTTGCGTTGTCCGTGTCGGCAGCGTCGCCCTACAGCGCGAGCTCGCCGCGTCGCCCTATCGCGCGCGCGAGCCCGCTGGGCGTCGATCTACGTAGCTACATGCGCCCGCCACGCTCTGATGTCTGCGTCCTCGTCGCGTCGCCCTGCAGCGCGAGCTCGCCACGCTCTGTCGTTTGCGTCCTCATCGCGTCGTCGTGCGCACAAGGCAGGCCGCCCCCTGCCGTGCCCATTGCTTCGTCGTGTCCTGCCCCTGTCACGTTGCGTACAACCGAGGAGCTCGGCGGCTACCTGACGGGAGGGACCTGGGAGCGCCGCCCTGGTCCGCAGCAAGTTCGGCATGACGTCGCGCCGCCTCCACCGTGGCCGCATCGCTCCGCTCGCCTCCCGTCGCGCACGCACACAAATGCATCGGAGGAGAGAGGGATGGCATTTTTCCATTGCCTCTCTCCTCCGAGCCATTTTTCCTCTTGGGTTGCCTCCTCTGTTGGAGCTCCTACGGTAGCTGGTGGAGAGCCAAAAATGAATTTGCCTCCCCATATAGCTCACCTGTTGAAGTCAGTCTCATGTGCTTTTGTTTGACTATTTACAGATGCATATTTTTAGCTGTGGTAACTCCTATAGTATTGTCGCGTGTTAATTGTTCTTTGTTACAACAAATGCTTGATTTAACTTAGGTAAAGTAATATGTAGGATATTGCTTCATTTTTCTAATGAAATCTATGTATCTTGGGTTATTTATGTCCAATTAACTAATGTTTTACTTAGTTTGACTCTTAATAAAAATGAAATGACAAAATAAAATAAGTAAAACCCCAGAATATTATTCCAATCTCTGCAATGAATAATACATGTAACTATTTCTTATTACATCCATTAAAGAAAGAAAAAGAAAACATTCCTACTCTCAACTTCCAATCCCTAAACACATGTAACACTGAATGCTTAAAGTTGTTCTAGCCTCCGAAGTCATAAAAACCAAATTGAAGGGATCTTAAGCTCAAAAGCTCCTTCCACCCAGATCCTGGAAATGACGAGAAGAACATGAGTGCCAAAACCACATGCTCATTTAACTCCAACGCTTTAAGGGAAATAATATGAGTCGTTGCTTTCTCAGCAATCCTACGTTTATTTTATCCCTCCATCGTTTCCTAAACCCATTCTATTGTTACCTTAATGTGGTAATTCACTGCATGAATAACTCGTGTTGTTCTGTTATGACCGGCATAAGTATATGATATACACGATATAGTGTAATGTACTGTATATTTTAGGTCACTATGGAAGTTCAAAATTTTAGAGCACCCTAATGCTGGTTCGATTCTTTCTGCCCCTGGGGATAGTTCGGTTCGCATGCTAATGATGGTTCGGTTCCTGCAGTGCTGCGTTCGTTATAACCGAATATAAGACAGAAAAGAAAGAGATTTGCTGCCGTGCGACCGGGAAAGATTGGCCCACATGGACGGCGAGCTACGCAATTAAGAATTGGTTATTCCCTCGTTCGAACCGAGTTGAACATTCGAACGACGGAAACTTTTACCTATAGTTCGACGTCTGCAGTGTACAAATCGAACCCTGCTCACTGGCTTCTCTCCTCACGGTTCGGTTTCACCTCTCATTGTTGATGCTCTTAAGTTACATGTTGAAAAATCAGTAAAAAAAAATACTTGGAATGTGTTGTAACAACTTAGAACAGTATTTTTTTTTTGTAAATATGCTTTCTTCAGTTTGGCACGCAACGTCAAAGAACAGAAGAAAATTGTACAGGAGATCTAACGGTTATATGGAACATACAAAATCGAGATAAAAGAAAACAGGGGAAATAATAAAAAGGGCAGGGGTAAAACGGAGAAGACCACCCACAGATTCCTGCTTTGTTTTGTTCTCCCCTGCCTCGCCGCTCGCCAAATCGCTGCTGCTGCATCCCCCACCTCAAGCCGCATCCGCAGCCGCCTACCTCTGCGCGGATTCCAAAGCCGGCAGCCGCCCGCCCGTACCTCCTCCTCCGCCTCGCTGCCTCCGGAACCTTTCAAGAAGCAGGCTTCGGGCTCTCACGCCCAGCCGCGCCCGGAGTTCGATGTCCGTTTGCACGCGATGGATCCGCACCGCCTGACCGGCCCCGCCGCGACCGACGCCGACGATGCCGACGACTGGGGTAAAGTTGCGAAACACCTGCCCACGTTTTGTTCTCTGGTTCTCGTTAGGGTTTCGCATCTTAATGGGTGAATAAATGCTATGCCTGTGATTTAGTTTCGTCCGCGTTCGTAATATCCCTGGGCGAATCTAAGAACCATCCTTGAAAATGAAGTGAGATTAGCACGCTTATTTGCTAATGCTAGCATCTGCGGCAGTCATTGGATACTCATAGGTTAACAATCGGCGTGATTTAGCTCAGAAACAGTTGTTGTAACTAATAATTTTATTTTGGGCACCTCTACATAGTGAGAATTTCCCTGCCCATGACAACTTTATGGGGGATGTAGAAAGGACATAGTGACCTTGCTGGCAAATTTGACTGGGAGCTTTATGATTCTTGTTTCCTTTGAACCAAGGAACTTAATAATTCTTAATGAATAAAGTTCGCTGCAATCAGGTATACTTCAAATATTGGCTGCGCCACCCCCCCCCAAAAGCCCCACCCGCAGCCGCCTACCTCTGCGCGGATTCCAAAGCCGGCAGCCGCCCGCCCGTACCTCCTCCTCCGCCTCGCTGCCTCCGGAACCTTTCAAGAAGCAGGCTTCGGGCTCTCACGCCCAGCCGCGCCCGGAGTTCGATGTCCGTTTGCACGCGATGGATCCGCACCGCCTGACCGGCCCCGCCGCGACCGACGCCGACGATGCCGACGACTGGGGTAAAGTTGCGAAACACCTGCCCACGTTTTGTTCTCTGGTTCTCGTTAGGGTTTCGCATCTTAATGGGTGAATAAATGCTATGCCTGTGATTTAGTTTCGTCCGCGTTCGTAATATCCCTGGGCGAATCTAAGAACCATCCTTGAAAATGAAGTGAGATTAGCACGCTTATTTGCTAATGCTAGCATCTGCGGCAGTCATTGGATACTCATAGGTTAACAATCGGCGTGATTTAGCTCAGAAACAGTTGTTGTAACTAATAATTTTATTTTGGGCACCTCTACATAGTGAGAATTTCCCTGCCCATGACAACTTTATGGGGGATGTAGAAAGGACATAGTGACCTTGCTGGCAAATTTGACTGGGAGCTTTATGATTCTTGTTTCCTTTGAACCAAGGAACTTAATAATTCTTAATGAATAAAGTTCGCTGCAATCAGGTATACTTCAAATATTCCTTTTATTGTATTTTCAATGAGACAATAGGTTCCGTAGACCAATTATCTGCTCTCCTAGGTTACATTGTACTTTATTTCTTCAAATAATAATTCATTGATTGACACCTGAATGACGTCAATCTCTGTCATTATAAGATTTTTTTTATGTTTGTGCTTAACTGCTATGCGCCATGCGTTGGACAAATGGGATGACACAGATCTGAACAGTTTTCCATATCGGCCAATAAATATGGTGAACGAACCTCTTGTAGGCTCCTTGAGGTTTCGACATAAATTACTACTTTATTGATTGCTTTGTACATCCCGTAGTTTGTTATGGCACAGAAACTTGTTGGTGCTTGATTTTTTGAGATATTTTTGTTTATTTTTCTTGCATAATTGCGAATGAACAATTCATATTAGTGCTTTGTGATCTAATTGAAAGTACTAGATTGAGTGGTGTAAATAGGATAACGAAATGGAATAACATCAGGAGCTACACGCAAGACATCAATATCCCTAGAATTTCTCTGTCACCCTGTGTAGCTATATGCTTATACCATAGCATGTCAAAAGAAAGAACGAAAATGATGATGTTCCTGTAAGGACCATAGTTTTTAAGGAGGTGTGGCGCTCTGAGGCGATCAGGGTGAGACTGCTAAGCCTAGGCGACAAGGTGACACTATAGTCTTAAGGCGTCCCCTTGTAACTGTAGGGACGCCTAAGCTACGCCTTGAAAACTTAGTGTAGCACCAAGTTTTGTTGTTGTGGCCAATGTATTTAAGCTAAGGTAATAGTCATCACGAAAGCGCTCACTGAATCACTGGTGCTGAACTAAAAATCAAACAAAGCTAGAACAGAATGGTGCACACATTACGGTCAATGATAATACATAGTGGTCTCACATAGATCATTTGTAATTTCTTGTATGAGCAATTGAGCATGTTAGATTGCACACCTCAAACTTAGAATTTGATGTTACATTTCGATATGTTACTTGACTGCAGTGCTAATGTGCAATCCTTGATAAATTTTGACGCATATCGTAGTTGGGCTTGCCTTTTGGGCTTGGATGGTGGACTTTTATATATGAGTCATAGAAATGACATGCCATCTAGACCCTGTGTTAGTAAACACAAATTGATGTGCTAGGCCATCAAGTTGAATATAGGCAATAGGAGTCCCGTGTCATGGTCGGTATCCAGTATCAAAGGAGGCGCAGGAGGG
