# Supplementary material for: Population genomics uncovers loci for trait improvement in the indigenous African cereal tef (Eragrostis tef)
Source: Commun Biol. 2025 May 26;8:807. doi: 10.1038/s42003-025-08206-5 (PMC12106829; doi:10.1038/s42003-025-08206-5)
Supplement: Supplementary file 12 — Reporting Summary [file 42003_2025_8206_MOESM12_ESM.pdf]

nature portfolio

Corresponding author(s): Oluwaseyi Shorinola, Solomon Chanyalew, Cristobal Uuay

Last updated by author(s): Feb 21, 2025

Reporting Summary

Nature Portfolio wishes to improve the reproducibility of the work that we publish. This form provides structure for consistency and transparency in reporting. For further information on Nature Portfolio policies, see our Editorial Policies and the Editorial Policy Checklist.

Statistics

For all statistical analyses, confirm that the following items are present in the figure legend, table legend, main text, or Methods section.

n/a	Confirmed	
☐	☒	The exact sample size (*n*) for each experimental group/condition, given as a discrete number and unit of measurement
☒	☐	A statement on whether measurements were taken from distinct samples or whether the same sample was measured repeatedly
☐	☒	The statistical test(s) used AND whether they are one- or two-sided<br>*Only common tests should be described solely by name; describe more complex techniques in the Methods section.*
☒	☐	A description of all covariates tested
☐	☒	A description of any assumptions or corrections, such as tests of normality and adjustment for multiple comparisons
☐	☒	A full description of the statistical parameters including central tendency (e.g. means) or other basic estimates (e.g. regression coefficient) AND variation (e.g. standard deviation) or associated estimates of uncertainty (e.g. confidence intervals)
☐	☒	For null hypothesis testing, the test statistic (e.g. *F*, *t*, *r*) with confidence intervals, effect sizes, degrees of freedom and *P* value noted<br>*Give P values as exact values whenever suitable.*
☒	☐	For Bayesian analysis, information on the choice of priors and Markov chain Monte Carlo settings
☒	☐	For hierarchical and complex designs, identification of the appropriate level for tests and full reporting of outcomes
☒	☐	Estimates of effect sizes (e.g. Cohen's *d*, Pearson's *r*), indicating how they were calculated

Our web collection on statistics for biologists contains articles on many of the points above.

Software and code

Policy information about availability of computer code

Data collection	Data collection did not involve the use of software or code.
Data analysis	We used open source and custom software for all the analyses described in the manuscript.<br><br>Open source software used included: fastp, Bowtie2 (v2.4.1), SAMtools (v1.18), BCFtools (v1.18), R(v4.1.3), VCFtools (0.1.15) , Plink (v1.90b4.6), vcf2phylip (v2.9), IQ Tree 2, ggtree (v3.10.1), ggtreeExtra (v1.12.0), ADMIXTURE v(1.3.0), SNPRelate (v1.29.0), Pophelper (v2.3.1), Plotly (v4.10.1), MetaboAnalyst 6.0, lme4 (v1.1.32), SeqinR (v4.2-36), GAPIT (v3), TASSEL (v5.2.54).<br><br>We deposited or used available scripts for kmer-based analyses. This included previously published scripts for making kmer matrix and for kGWAS available from: https://github.com/wheatgenetics/owwc/tree/master/kGWAS; with additional guidance provided at https://github.com/quirozcj/kmerGWAS_descriptions. Scripts for the shared k-mer state rates are available at https://github.com/Uauy-Lab/tef_kGWAS_2024.

For manuscripts utilizing custom algorithms or software that are central to the research but not yet described in published literature, software must be made available to editors and reviewers. We strongly encourage code deposition in a community repository (e.g. GitHub). See the Nature Portfolio guidelines for submitting code & software for further information.

Data

Policy information about availability of data

All manuscripts must include a data availability statement. This statement should provide the following information, where applicable:

- Accession codes, unique identifiers, or web links for publicly available datasets
- A description of any restrictions on data availability
- For clinical datasets or third party data, please ensure that the statement adheres to our policy

Data are deposited in relevant repositories. Sequencing data is available via NCBI SRA under BioProject ID PRJNA1150514. Raw phenotypic data can be found in the Supplementary Tables. Raw metabolomic data and VCF files are available at Zenodo (https://doi.org/10.5281/zenodo.13837319)

Research involving human participants, their data, or biological material

Policy information about studies with human participants or human data. See also policy information about sex, gender (identity/presentation), and sexual orientation and race, ethnicity and racism.

Reporting on sex and gender	Not Applicable as the research does not involve human participants, their data or biological materials
Reporting on race, ethnicity, or other socially relevant groupings	Not Applicable as the research does not involve human participants, their data or biological materials
Population characteristics	Not Applicable as the research does not involve human participants, their data or biological materials
Recruitment	Not Applicable as the research does not involve human participants, their data or biological materials
Ethics oversight	Not Applicable as the research does not involve human participants, their data or biological materials

Note that full information on the approval of the study protocol must also be provided in the manuscript.

Field-specific reporting

Please select the one below that is the best fit for your research. If you are not sure, read the appropriate sections before making your selection.

☐ Life sciences ☐ Behavioural & social sciences ☒ Ecological, evolutionary & environmental sciences

For a reference copy of the document with all sections, see nature.com/documents/nr-reporting-summary-flat.pdf

Ecological, evolutionary & environmental sciences study design

All studies must disclose on these points even when the disclosure is negative.

Study description	We phenotyped and resequenced a collection of tef (Eragrostis tef) accessions from Ethiopia, and conducted genome-wide association analyses to identify loci underlying important agronomic, grain size and metabolome traits. The accessions were phenotyped in three field trials using an augmented block design. In each trial, each accession was sown in 1 m rows spaced 50 cm apart.
Research sample	In this study, we performed experiments on tef (Eragrostis tef) which is a semi-tropical PACMAD grass within the Chloridoideae subfamily. Our study focuses on a core collection of tef accessions maintained by the Ethiopian Institute of Agricultural Research (EIAR, Ethiopia). This collection comprises 225 tef accessions that sufficiently captures the phenotypic and genetic diversity of a wider collection of 2,715 tef accessions as described in our study.
Sampling strategy	We did not perform sample-size calculations. Instead these sample sizes were pre-determined by the number of germplasms present in the target population of our study.<br>However, the final number of accessions used in specific analyses varied due to unforeseen circumstances. Some samples failed during the phenotyping and/or sequencing stages, and some were excluded due to genetic redundancy, as detailed in the manuscript. Figure 2 provides a breakdown of the number of accessions used for each experiment and analysis. For the phenotypic and metabolome analyses, we used data from ~700 samples derived from 224 accessions. This dataset was sufficiently normally distributed to satisfy the assumptions of the statistical test (mostly t-test and linear mixed modelling) used for hypothesis testing. For GWAS analyses, we used best linear unbiased estimates for 141 accessions, after accounting for genetic redundancy between accessions. While this sample number is limited for detecting small effect loci, samples from 60 - 150 have been used for GWAS across different cereal crops to detect QTL with large effects (Soumya et al., 2021, Scientific Report; Shorinola et al., 2022, G3).
Data collection	Agronomic data from field trials was collected by authors from the Ethiopian Institute of Agricultural Research (EIAR, Ethiopia) using data recording field books and translated to electronic records. Grain size and metabolome measurements were recorded automatically by the instrument used for measurement as described in the Methods section.

Timing and spatial scale

Agronomic data for all the study samples were collected across the three trial locations between July - November 2020. Grain size data was collected for all samples harvested from the field trials between October - December 2021. Metabolite extracts were obtained from the grain samples immediately after grain size measurement and were stored in the freezer in preparation for metabolome profiling. Flow Infusion Electrospray High-resolution Mass Spectrometry (FIE-HRMS) measurement was done for all metabolite samples between June and July 2022.

Data exclusions

We excluded a few samples for a subset of the analyses in this study. Two accessions were excluded from the phylogenetic clustering analysis as they were found to have high genetic heterozygosity (22.7% and 24.9% heterozygous sites, versus an average of 1.5% (standard deviation (SD) = 0.7%). The seed packets for these accessions were likely contaminated. Furthermore, following phylogenetic clustering of the tef accessions into 150 genetically distinct accession groups or singlets, we excluded nine of these accession groups due to the heterogeneity of the seed colour within the groups. While these genetically redundant groups show phylogenetics and kmer profile similarities (>96% shared kmer), they likely still retain some residual heterozygosity that could account for phenotypic heterogeneity within the groups.

Reproducibility

The accessions used in this study were phenotyped three times in locations representing different agro-ecological zones in Ethiopia. Observations across these locations were consistent for qualitative traits (Supplementary Figure 4). For environmentally-sensitive quantitative traits, we performed statistical modelling to account for variation between locations and obtained a robust and representative estimate for genotypic effects. The multilocational phenotyping and statistical modelling approaches that we employed together contribute to the robustness and reproducibility of our study.

Randomization

We used an augmented block design for each of the three field trials conducted. Most accessions were sown once randomly across different blocks and five control accessions were replicated four times within each block. We used a linear mixed modelling approach to account for block effects within each trial location and location effects across trials. In addition, following phylogenetic analyses, we identified accessions that were genetically redundant. We subsequently updated the field trial design to reflect the treatment of redundant accessions as combined redundancy groups. That is, accessions in each redundancy group were treated as replications to derive a common genotypic effect.

Blinding

Blinding is not relevant for this study as there is little or no sample or researcher bias that could affect the result obtained in this study. First, our study samples are plants and are not vulnerable to bias that could impact the outcome of the study. Second, each of the analyses described in the study were performed by different researchers most of whom have no prior knowledge of the genetic composition of the study samples.

Did the study involve field work? ☒ Yes ☐ No

Field work, collection and transport

Field conditions

The field work was carried out in three experimental stations across three different locations in Ethiopia: Alem Tena, Debris Zeit and Chefe Donsa. Alem Tena has a mean rainfall of 689 mm, the max daily temperature of 29.5 oC and minimum daily temperature of 15.3 oC. Debre Zeit has a mean rainfall of 984 mm, the max daily temperature of 26.8 oC and minimum daily temperature of 11.4 oC. Chefe Donsa has a mean rainfall of 1020 mm, the max daily temperature of 20 oC and minimum daily temperature of 8oC.

Location

Alem Tena is located in latitude 8o 20' N and longitude 38o 57' E. Debre Zeit is located in latitude 8o 44’ N and longitude 38o 58’E Chefe Donsa is located in latitude 8o 57' N and longitude 39o 16’E.

Access & import/export

All of the field trials were conducted in dedicated experimental stations that are routinely used for agricultural research by EIAR. EIAR has a government mandate to conduct agricultural research in Ethiopia. We did not export any viable plant material out of Ethiopia. We only exported DNA material and metabolite extracts from samples. Prior to export, we obtained approval from the Ethiopian Biodiversity Institute who manages access rights for tef germplasm in Ethiopia.

Disturbance

The field work was conducted in dedicated experimental field stations on an agricultural crop. There was no additional disturbance to people or the environment.

Reporting for specific materials, systems and methods

We require information from authors about some types of materials, experimental systems and methods used in many studies. Here, indicate whether each material, system or method listed is relevant to your study. If you are not sure if a list item applies to your research, read the appropriate section before selecting a response.

Materials & experimental systems

n/a	Involved in the study
☒	☐ Antibodies
☒	☐ Eukaryotic cell lines
☒	☐ Palaeontology and archaeology
☒	☐ Animals and other organisms
☒	☐ Clinical data
☒	☐ Dual use research of concern
☐	☒ Plants

Methods

n/a	Involved in the study
☒	☐ ChIP-seq
☒	☐ Flow cytometry
☒	☐ MRI-based neuroimaging

Dual use research of concern

Policy information about dual use research of concern

Hazards

Could the accidental, deliberate or reckless misuse of agents or technologies generated in the work, or the application of information presented in the manuscript, pose a threat to:

No	Yes
☒	☐ Public health
☒	☐ National security
☒	☐ Crops and/or livestock
☒	☐ Ecosystems
☒	☐ Any other significant area

Experiments of concern

Does the work involve any of these experiments of concern:

No	Yes
☒	☐ Demonstrate how to render a vaccine ineffective
☒	☐ Confer resistance to therapeutically useful antibiotics or antiviral agents
☒	☐ Enhance the virulence of a pathogen or render a nonpathogen virulent
☒	☐ Increase transmissibility of a pathogen
☒	☐ Alter the host range of a pathogen
☒	☐ Enable evasion of diagnostic/detection modalities
☒	☐ Enable the weaponization of a biological agent or toxin
☒	☐ Any other potentially harmful combination of experiments and agents

Plants

Seed stocks	All the seed stocks for field phenotyping used were obtained from the Ethiopian Institute of Agricultural Research. The accession numbers of the seed stock are presented in Supplementary Table 7.
Novel plant genotypes	We did not generate any novel plant genotypes in this study. We used a natural population of tef accessions in this study.
Authentication	We sequenced all the tef accessions and used this data to identify accessions that likely have mixed seed stock (unusually high heterozygosity) or are genetically redundant. Only distinct, clean, and non-redundant accession groups were used for GWAS analyses.
